# Synthesis of chiral α-trifluoromethyl alcohols and ethers via enantioselective Hiyama cross-couplings of bisfunctionalized electrophiles

**DOI:** 10.1038/s41467-018-05946-3

**Published:** 2018-09-03

**Authors:** Andrii Varenikov, Mark Gandelman

**Affiliations:** Schulich Faculty of Chemistry, Technion-Israel Institute of Technology, Technion City, 3200002 Haifa, Israel

## Abstract

Methods for synthesis of chiral organic compounds bearing trifluoromethyl-substituted stereocenters are of great interest for agrochemical and pharmaceutical labs and industries in their search for new bioactive materials. We report on employment of bisfunctionalized electrophiles, bearing both a trifluoromethyl and a functional group as direct substituents of the reactive center, in cross-coupling reactions. We exemplify this concept in the asymmetric synthesis of enantioenriched α-trifluoromethyl- and perfluoroalkyl-containing benzylic and allylic ethers and alcohols by nickel-catalyzed stereoconvergent Hiyama cross-coupling reaction. Substrate electrophiles are conveniently prepared in few steps from trifluoroacetic acid. The method represents a conceptually different approach to chiral CF_3_-substituted alcohols and ethers and allows for a rapid catalytic preparation of a wide range of these valuable compounds in high yields and enantioselectivity.

## Abstract (web summary)

Compounds containing stereocenters bearing -CF_3_ groups, especially in proximity of heteroatoms, are of great interest for drug and agrochemicals development. Here, the authors report a nickel-catalyzed Hiyama cross-coupling methodology for the asymmetric synthesis of valuable alpha-trifluoromethyl alcohols and ethers.

## Introduction

Fluorine-containing compounds have attracted significant attention over recent decades, since incorporation of the fluorine atom can profoundly affect its molecular behavior^1^. Fluorine incorporation is a key tool used in the pharmaceutical and agrichemical industries to alter activity, bioavailability, and metabolic stability of the organic compound^2, 3^. Among other fluorinated functionalities, the trifluoromethyl group is widely employed in existing drugs and drug candidates^4–6^. Therefore, methods of CF_3_-group introduction, especially with concomitant construction of the stereodefined trifluoromethyl-substituted stereogenic centers, are of great importance^7–12^. Although the development of synthetic routes for the preparation of materials possessing these asymmetric carbon centers is a vibrant field, an attractive approach for their enantioselective creation by means of the cross-coupling reactions remains mainly unexplored. The sole reported example demonstrates a catalytic synthesis of enantioenriched CF_3_-substituted alkanes by Negishi cross-coupling reaction^13, 14^. This omission is rather remarkable, given that cross-coupling transformations proved to be highly useful and diverse tool for the asymmetric catalytic synthesis of compounds bearing related fluorine-substituted stereogenic centers^15, 16^.

**Fig. 1:**
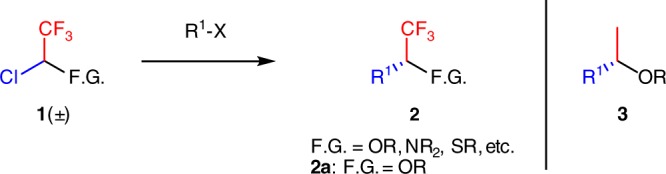
Proposed pathway towards formation of α-CF_3_-substituted functionalized compounds. Electrophiles bearing –CF_3_ group in proximity to heteroatom can serve as attractive starting materials for the preparation of enantioenriched trifluoromethylated ethers, amines, thioethers, etc

We envisioned that the utilization of electrophiles of type **1** (Fig. 1) bearing both a CF_3_ and a functional group at the reaction center in cross-coupling reactions would represent a desirable means to augment molecular complexity and facilitate the construction of synthetic libraries of α-CF_3_ substituted alcohols, ethers, amines, sulfides, etc. Moreover, employing a racemic mixture of the building block **1** in an asymmetric stereoconvergent catalytic transformation would provide important compounds **2** in enantioenriched manner.

**Fig. 2:**
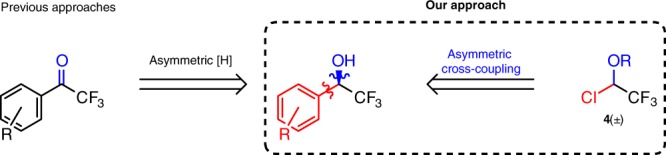
Approaches towards chiral α-trifluoromethyl alcohols. Asymmetric hydrogenation represents a major way to prepare enantiospecific α-CF_3_ benzyl alcohols. Our approach is based on asymmetric cross-coupling reaction of building block 4

Initiating an active program in this direction we have primarily focused our efforts to examine the feasibility of this approach with regard to the asymmetric synthesis of trifluoromethyl-substituted benzyl (and alkyl) alcohols and ethers (Figs. 1, 2a). These compounds are extremely attractive building blocks since such motifs are found in a large number of biologically active compounds^17–19^. Moreover, substructure **3** (derivatives of phenethyl alcohol or its ethers) appears in a variety of bioactive compounds and existing pharmaceuticals (for example, commercial drugs, containing fragment 3; see Crizotinib, Aprepitant, Vestipitant, Fosaprepitant). Therefore, our approach, if successful, would render practical enantioselective synthesis of trifluoromethylated analogs of these materials.

It should be noted that all current synthetic methods for the preparation of highly enantioenriched α-CF_3_-substituted alcohols (**2a**, R = H) rely on enantioselective reduction of corresponding aryltrifluoromethyl ketones, utilizing chiral iridium or rhodium catalysts or chiral boranes (Fig. 2)^20–22^. An alternative approach by addition of trifluoromethyltrimethylsilane (TMSCF_3_) to aldehydes is known; however, this method is reported to provide moderate enantioselectivity^7, 23^.

In this paper we reveal a totally different synthetic approach for the preparation of these useful compounds in high yields and enantioselectivity by means of cross-coupling chemistry. Thus, we report a nickel-catalyzed asymmetric stereoconvergent Hiyama cross-coupling reaction for the synthesis of enantioenriched α-trifluoromethyl alcohols and ethers (aryl, vinyl, and alkyl). The approach can be extended to the preparation of chiral perfluoroalkyl alcohols. Substrates for this transformation are conveniently prepared in a few steps from trifluoroacetic acid (or its anhydride), as an economical source of the trifluoromethyl group (**4**, see Fig. 2). To the best of our knowledge this is the first example of an employment of electrophiles bearing both a trifluoromethyl and a functional group as direct substituents of the reactive center. Such electrophiles represent an attractive entry for cross-coupling transformations for the rapid increase of molecular complexity and asymmetric preparation of a wide class of organic compounds bearing CF_3_-substituted stereogenic centers.

## Results

### Optimization of reaction conditions

For proof of concept, we explored compounds **4a** and **4b** (for the preparation, see Supplementary note 1) in an enantioselective Hiyama cross-coupling reaction. After comprehensive investigation of the reaction conditions, we were pleased to find that α-chloro-α-trifluoromehyl ethers **4** proved eminently suitable electrophiles in Hiyama cross-coupling reaction. Under conditions, revealed by our screening, **4a** provided the desired ether **5a** in excellent yield and enantioselectivity (93% yield and 95% ee) after 40 h (Table 1, entry 1). Notably, the reaction employing **4b** is completed after 16 h with no compromise in yield and ee (96 and 97% correspondingly). Therefore, further reaction conditions analysis was performed with substrate **4b** (entry 2).

**Table 1:** Optimization of the reaction conditions


Entry^a^	Variation	Reaction time (h)	Yield^b^	ee
1	**4a** as a starting	30	93%	95%
2	**4b** as a starting	16	96%	97%
3	No NiCl_2_·glyme	16	0%	—
4	No ligand **7a**	16	2%	—
5	5% NiCl_2_·glyme, 6% ligand	16	73%	75%
6	10 ^o^C instead of RT	60	7%	—
7	40 ^o^C instead of RT	16	15%	—
8	THF instead of DMA	16	97%	91%
9	Dioxane instead of DMA	60	73%	79%
10	Toluene instead of DMA	60	65%	26%
11	Ligand **7b** instead of **7a**	60	85%	21%
12	Ligand **7c** instead of **7a**	60	20%	<5%
13	Ligand **7d** instead of **7a**	16	89%	74%
14	Ligand **7e** instead of **7a**	60	2%	—
15	Ligand **7f** instead of **7a**	60	29%	<5%
16	Ligand **7g** instead of **7a**	20	94%	96%
17	No light	40	94% (52%^c^)	96%
18	White LED	20	96% (81%^c^)	97%
19	0.15 eq. H_2_O	16	90%	94%
20	Under air in closed vial	60	4%	—

^a^Reactions were performed with **4b** as a starting material on 0.06 mmol scale using 1.3 eqiv. PhSi(OMe)_3_, 2.5 eqiv. tetrabutylammonium triphenyldifluorosilicate (TBAT), 10% NiCl_2_·glyme, 11% ligand **7a** in *N*,*N*-dimethylacetamide (DMA), at room temperature with blue LED light irradiation

^b^NMR yields vs. internal standard

^c^After 16 h

We have found that the methylene bisoxazoline ligand **7a** in combination with NiCl_2_·glyme was an excellent and general catalyst for this process. Interestingly, structurally similar ligands **7b** and **7c** gave significantly poorer results in terms of yields and enantioselectivity (entries 11 and 12). A catalytic system based on ligand **7g** was as efficient as that of **7a**, requiring, however, longer reaction time (entry 16). Pyridine-based oxazoline ligands **7e** and **7f** proved to be inefficient in this transformation (entries 14 and 15). Variation in temperature has a profound effect on the reaction outcome: cooling the reaction to 10 ^o^C drastically decreases the reaction rate (entry 6), while heating up to 40 ^o^C causes conversion of trimethoxy(phenyl) silane to phenyl tetrafluorosilicate, which is not active as a nucleophile under our reaction conditions (entry 7). While the reaction could proceed in darkness, irradiation with a white or blue LED lamp significantly accelerated the process (compare entry 17 with 2 and 18). Although the precise mechanism of this photoinduction is to be clarified, we presume that it facilitates an oxidative addition of electrophile **4** to an excited Ni(I) catalytic species which likely takes place by radical mechanism. Induction of radical type oxidative addition step by light excited transition metal complex was previously reported^24^. Small amounts of water could be tolerated with no significant deterioration of the reaction outcome (entry 19); however, the reaction should be performed under anoxic conditions (entry 20).

### Substrate scope

**Fig. 3:**
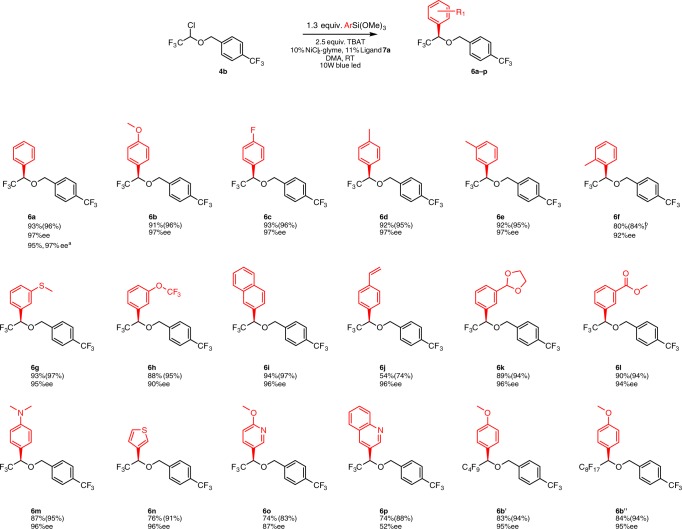
Examples of the Hiyama cross-coupling reaction with aryl silanes. Various aryls and heterocycles can be introduced. Isolated yield is reported (^19^F NMR yield is given in brackets). Reaction conditions: **4b** (1 equiv.), (hetero)aryltrimethoxy silane (1.3 equiv), TBAT (2.5 equiv.), NiCl_2_·glyme (0.1 equiv.), Ligand **7a** (0.11 equiv.), DMA, room temperature, 10 W blue led. ^a^Reaction on 2 g scale of **4b**; ^b^reaction time 48 h

Having optimized conditions in hand, we subjected **4b** to the reaction with different aryl silanes as nucleophiles (Fig. 3). Gratifyingly, various electron-rich and electron-poor aryl trimethoxy silanes are suitable partners for this reaction, providing the corresponding cross-coupling products in excellent yields and enantioselectivity. Moreover, *ortho*-, *meta*- and *para*-methyl substituted aryl silanes are amenable to this process (compounds **6d**, **6e**, and **6f**). *Ortho*-substituted aryl silanes require significantly longer reaction times (48 h), probably due to slower transmetallation to nickel catalyst, while the yield and ee of the product are only slightly diminished (entry **6f**). Interestingly, a styrenyl group could also be incorporated by this transformation; however, the ether was isolated in comparatively diminished yield, likely due to the partial oligomerization of the styrenyl residue (entry **6j**). Notably, heterocyclic silanes can be employed as nucleophiles in this reaction. The corresponding ethers were obtained in good yields and ee (**6n**−**p**). This method is scalable: cross-coupling of **2g** of substrate **4a** with PhSi(OMe)_3_ furnishes ether **6a** in 95% yield and 97% ee.

Although we focused our efforts on the asymmetric catalytic synthesis of trifluoromethyl-substituted benzyl alcohols and ethers, perfluoroalkyl motifs such as −C_4_F_9_ and −C_8_F_17_ could be successfully introduced (entries **6b′** and **6b″**) without any modification of the reaction conditions. As such, the current methodology gives access to higher perfluorinated homologs of compound **6**.

**Fig. 4:**
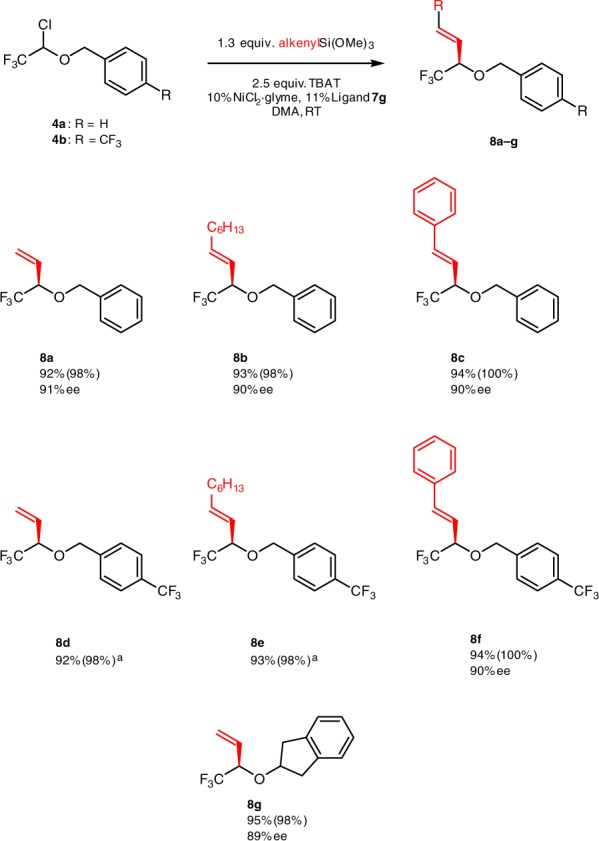
Selected examples of the cross-coupling reaction with alkenyl silanes. Isolated yield is reported (^19^F NMR yield is given in brackets). Reaction conditions: electrophile (1 equiv.), alkenyl trimethoxy silane (1.3 equiv), TBAT (2.5 equiv.), NiCl_2_·glyme (0.1 equiv.), Ligand **7g** (0.11 equiv.), DMA, room temperature. ^a^Enantiomers are not separated by HPLC

The reaction is not limited to aryl silanes. We have found that alkenyl trimethoxy silanes are highly efficient nucleophiles, which furnish α-trifluoromethylallyl ethers **8** in practically quantitative yield and high enantioselectivity (Fig. 4). In this case, ligand **7g** was used as it provided superior enatnioselectivity compared to ligand **7a**. For example, compound **8a** was obtained in 65 and 90% ee with ligands **7a** and **7g**, correspondingly. While light irradiation also has a positive effect on the rate of the reaction with alkenyl silanes, it is not necessarily required here, as the reaction is complete in ca. 4 h. Notably, incorporation of an alkenyl motif allows for further versatile synthetic modification of the resulting products.

**Fig. 5:**
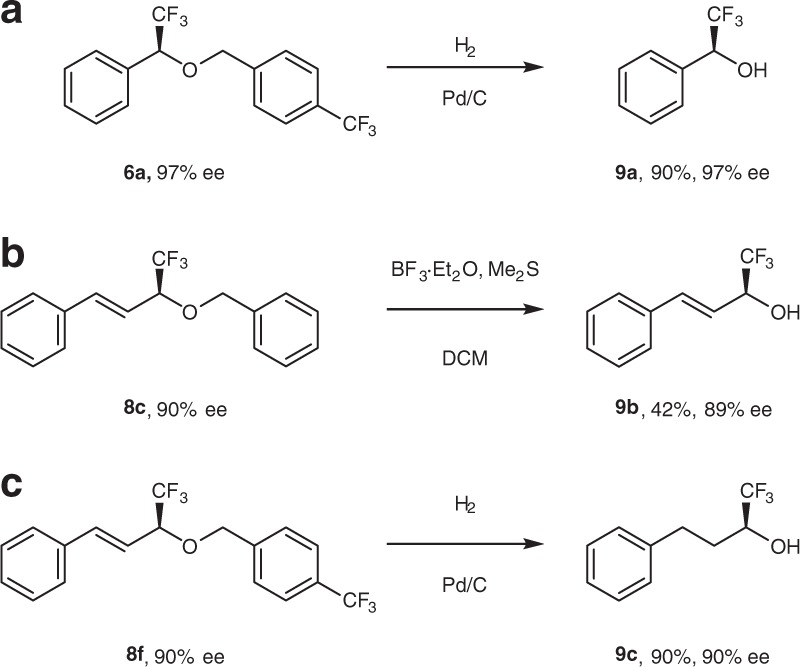
Preparation of (α-trifluoromethyl) aryl, vinyl, and alkyl alcohols by deprotection (or reductive deprotection) of corresponding ethers. **a** Deprotection of benzylic ether **6a** by palladium-on-carbon-catalyzed hydrogenation. **b** Deprotection of **8c** with BF_3_ etherate and dimethylsulphide to give allylic alcohol **9b**. **c** One-pot reductive deprotection of **8f** with hydrogen and palladium on carbon to give alkyl alcohol **9c**

Since chiral trifluoromethylbenzyl alcohols are valuable building blocks for organic synthesis, we explored a deprotection of ethers **6** and **8** in order to examine a feasibility of preparation of these useful compounds. Notably, unprotected α-trifluoromethylbenzyl alcohols were prepared by simple hydrogenolysis of the corresponding ether in high yield and without loss of the initial ee (Fig. 5a; exemplified on ether **6a**). The protective benzyl group in alkenyl ethers **8a−c** was removed upon their treatment with BF_3_/Me_2_S to result in corresponding *allyl* alcohols in moderate yield and high ee (Fig. 5b, exemplified on ether **8c**). Interestingly, when allyl ether **8f** was subjected to the reductive deprotection conditions (H_2_, Pd/C), saturated (α-trifluoromethyl) *alkyl* alcohol **9c** was smoothly obtained in 90% yield and with no deterioration of ee (Fig. 5c). As such, this reaction provides an entry to the preparation of (α-CF_3_)-alkyl-substituted alcohols in addition to their aryl and alkenyl counterparts.

After exploring the scope of nucleophiles, we have studied various α-chloroethers as electrophiles in our cross-coupling reaction. Gratifyingly, the method is not limited to 4-(trifluoromethyl)benzyl substituted ether **4b**. Precursors bearing various alkoxy (RO-) groups, which contain ether (**4c**), ester (**4d**), protected amine (**4e**), benzyl (**4a**) or even an unfunctionalized alkyl moiety (**4f**) proved to be highly efficient in this cross-coupling transformation (Table 2). As such, the functionalized (trifluoromethyl)benzyl ethers **11** can be directly prepared in high yield and enantioselectivity from the corresponding substrates **4**. While the reaction works with primary and secondary ethers, our attempts to use tertiary counterparts gave negligible conversion with traces of the target product. Having initiated the current work with R = benzyl group (**4a**) as a convenient handle for studies in methodology development, and having demonstrated diverse functionalization of the perfluoroalkyl-bearing part of the ether, we are in a position to conclude that the other half of the ether moiety could be freely modified without loss of reaction performance. This means the previously discussed deprotection to the alcohol to permit further functionalization to the corresponding ether is superfluous, although it remains a viable route where the benzyl group is desired for the orthogonal protection in multistep synthesis or for further preparation of free alcohol.

**Table 2:** Cross-coupling of various ethers

^a^Isolated yield is reported (^19^F NMR yield is given in brackets). Reaction conditions: electrophile (1 equiv.), 4-methoxyphenyltrimethoxy silane (1.3 equiv), TBAT (2.5 equiv.), NiCl_2_·glyme (0.1 equiv.), ligand **7a** (0.11 equiv.) DMA, room temperature, 10 W blue LED

Interestingly, reaction times for various ethers could significantly vary. While **6b** could be synthesized in less than 16 h with light irradiation or in 40 h in dark, reaction with **4a** achieves full conversion in 30 h (60 h in the dark), but for alkyl-substituted analogs reaction times are 72 h (without light irradiation the reaction fails to reach full conversion). Presumably, an oxidative addition is the rate-limiting step and the lower the LUMO of the electrophile, the faster SET from the catalyst occurs, hence a faster reaction is observed. This hypothesis is congruent with observed reaction rates, while the reaction mechanism is likely to be similar to the previously reported for Ni-catalyzed stereoconvergent cross-coupling processes^25–27^.

## Discussion

We have developed a catalytic stereoconvergent approach to the synthesis of enantioenriched α-trifluoromethyl- and perfluoroalkyl-containing benzylic and allylic ethers and alcohols. Our method is based on the utilization of a bisfunctionalized electrophile, bearing both a trifluoromethyl and a functional group as direct substituents of the reactive center, in cross-coupling reactions. Significant functional group compatibility, high yields and enantioselectivity of the reaction allows usage of this method in the late-stage synthesis for the preparation of the complex systems. The concept of the employment of such bisfunctionalized building blocks in cross-coupling reactions opens a door to the rapid preparation of numerous chiral functional organic compounds bearing trifluoromethyl (or perfluoroalkyl)-substituted stereogenic center. Utilization of this concept for the asymmetric catalytic synthesis of α-trifluoromethylthiols and amines, among others, is under studies in our labs.

## Methods

### General procedure for the cross-coupling reactions

In the glovebox, in 20 ml glass vial, mixture of NiCl_2_·glyme (22 mg, 0.1 mmol, 0.1 equiv) and ligand **7a** (50.5 mg, 0.11 mmol, 0.11 equiv) in dry DMA (5 ml) were stirred for 1 h. The obtained solution of catalyst was diluted with DMA (5 ml), then TBAT (1.35 g, 2.5 mmol, 2.5 equiv.) and trimethoxy(aryl) silane (1.3 mmol, 1.3 equiv.) were added to the vial, followed by the solution of the electrophile (1 mmol, 1 equiv.) in 5 ml of DMA. The vial was tightly closed with PVC tape and stirred outside of the glovebox with additional light irradiation (household white-light 10 W LED lamp or blue-light 10 W LED lamp with *λ*_em_≈460 nm) for 16 h. After completion of the reaction, a solution was poured into 15 ml 0.5 M NaOH solution and stirred for additional 10 min. The obtained mixture was diluted with 45 ml of water and extracted with ether (3 × 20 ml). Combined organic fractions were washed with 10 ml of water, 10 ml of brine and dried over Na_2_SO_4_. The residue after solvent evaporation was subjected to column chromatography (silica gel, 230–400 mesh, hexane/DCM or hexane/EtOAc) to give the product.

### Data availability

The authors declare that the data supporting the findings of this study are available within the article and its Supplementary Information file. For the experimental procedures, NMR, and HPLC analysis of the compounds in this article, see Supplementary Methods in the Supplementary Information file. The X-ray crystallographic coordinates for structure reported in this article have been deposited at the Cambridge Crystallographic Data Centre (**11f**: CCDC **1854431**). These data could be obtained free of charge from The Cambridge Crystallographic Data Centre via www.ccdc.cam.ac.uk/data_request/cif.

## Electronic supplementary material

Supplementary Information
