# Supplementary material for: Synthesis of chiral α-trifluoromethyl alcohols and ethers via enantioselective Hiyama cross-couplings of bisfunctionalized electrophiles
Source: Nat Commun. 2018 Sep 3;9:3566. doi: 10.1038/s41467-018-05946-3 (PMC6120892; doi:10.1038/s41467-018-05946-3)
Supplement: Supplementary file 1 — Supplementary Information [file 41467_2018_5946_MOESM1_ESM.pdf]

Synthesis of chiral α-trifluoromethyl alcohols and ethers via enantioselective Hiyama cross-couplings of bisfunctionalized electrophiles.

Varenikov et al.

Supplementary methods

General information. All reactions were generally performed in glove box or in dried glassware under an atmosphere of dry N_2. Reaction mixtures were stirred magnetically unless otherwise indicated and monitored either by ^{19}F NMR spectroscopy of the reaction mixture or thin layer chromatography (TLC) on Merck precoated glass-backed silica gel 60 F-254 0.25 mm plates with visualization by fluorescence quenching at 254 nm. TLC plates were stained using potassium permanganate. Chromatography purification of products (flash column chromatography) was performed on silica gel 60 (70-230 mesh or 240-400 mesh Merck) using a forced flow of eluent at 0.3-0.5 bar. Concentration of reaction product solutions and chromatography fractions under reduced pressure was performed by rotary evaporation at 35-45 $^{\circ}C$ at the appropriate pressure and then at room temperature, c.a. 10 mmHg (vacuum pump) unless otherwise indicated. All chemicals, including dry solvents were purchased from Aldrich, Fluka, Acros, TCI, FluoroChem or Alfa Aesar and used as such unless stated otherwise. Yields given refer to chromatographically purified compounds unless otherwise demonstrated. Anhydrous DMA was purchased from Aldrich and stored over 4Å molecular sieves. TBAT was recrystallized prior to use.[1] Ligand was synthesized according to known procedures. Phenyl-, 4-methoxyphenyl-, p-tolyl- and 4-vinylphenyl trimethoxysilanes were obtained from commercial sources. ^{1}H NMR spectra were recorded on Bruker 600 MHz, 500 MHz, 400 MHz and 300 MHz spectrometer. ^{13}C NMR spectra were recorded on Bruker 125 MHz, 100 MHz and 75 MHz spectrometer. ^{19}F NMR spectra were recorded on Bruker 188 MHz. ^{1}H NMR chemical shifts are reported in parts per million (δ) downfield from tetramethylsilane (the peak of residual $CHCl_3$ in $CDCl_3$ at 7.26 ppm as reference). ^{13}C NMR chemical shifts are reported in parts per million (δ) downfield from tetramethylsilane (the central peak of $CDCl_3$ at 77.16 ppm as reference). ^{29}Si NMR chemical shifts are reported in parts per million (δ) downfield from tetramethylsilane (tetramethylsilane peak at 0 ppm as reference). ^{19}F NMR chemical shifts are reported in parts per million (δ) (C_6F_6 peak at -161.9 ppm as reference). All ^{13}C spectra are proton decoupling. NMR coupling constants (J) are reported in Hertz (Hz), and splitting patterns are indicated as follows: bs – broad singlet; s – singlet; d – doublet; dd – doublet of doublet; ddd – doublet of doublet of doublet; dt – doublet of triplet; t – triplet; tt – triplet of triplets; q – quartet; m – multiplet, dm – doublet of multiplets. High resolution mass spectrometric measurements (HRMS) were performed by the Waters LCT Premier and Bruker Maxis Impact with APCI solid probe. Enantiomeric excess was determined by HPLC analysis on Shimadzu HPLC (LC-20AT pump; SPD-M20A diode array detector; DGU-$20A_5$ degasser; SIL-20A auto sampler;. Short path distillation was performed using Buchi Glass Oven B-585 Kugelrohr.

Preparation of substrates

Reaction conditions for the following transformations are not optimized.

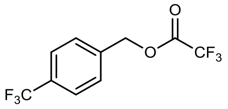

4-(trifluoromethyl)benzyl trifluoroacetate(**16a**).

To the solution of 3.52 g (20 mmol) 4-(trifluoromethyl)benzyl alcohol in 50 ml of dry dichloromethane was slowly added 4.4 g (2.95 ml, 21 mmol) of trifluoroacetic anhydride and stirred for 30 min. After evaporation of solvent, 4-(trifluoromethyl)benzyl trifluoroacetate was obtained as colorless liquid in quantitative yield (5.4 g).

^{1}H NMR (300 MHz, $CDCl_3$): δ 7.68 (d, J^{H-H}= 8.1 Hz, 2H), 7.52 (d, J^{H-H}= 8.1 Hz, 2H), 5.41 (s, 2H). **^{13}C NMR (75 MHz, $CDCl_3$):** δ 157.4 (q, J^{C-F}= 43.1 Hz), 137.2, 131.6(q, J^{C-F}= 33 Hz), 128.7, 126.1 (q, J^{C-F}= 3.8 Hz), 123.9(q, J^{C-F}= 271 Hz), 114.6(q, J^{C-F}= 285 Hz), 68.5. **^{19}F NMR (188 MHz, $CDCl_3$):** δ -63.0 (s, 3F), -75.0 (s, 3F). **R_f** = 0.41 (15% DCM/hexane).

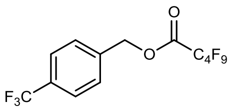

4-(trifluoromethyl)benzyl perfluoropentanoate(**16b**).

To the solution of perfluoropentanoic acid(3.96 g, 2.3 ml, 15 mmol), oxalyl chloride(1.4 ml, 16.5 mmol) in 50 ml of dry DCM was added dropwise dry DMF(0.25 ml, 3 mmol). Reaction was stirred until gas evolution has ceased (ca. 3h), then for additional 3h, and treated with solution of (4-trifluoromethyl)benzyl alcohol (2.3 ml 16.5 mmol) in 20 ml of DCM. After 1h solvent was evaporated and residue was subjected to column chromatography (eluent: 10% DCM in Hexane) giving the title compound in 71% yield (4.5g).

^{1}H NMR (300 MHz, $CDCl_3$): δ 7.67 (d, J^{H-H}= 8.1 Hz, 2H), 7.51 (d, J^{H-H}= 8.1 Hz, 2H), 5.43 (s, 2H). **^{13}C NMR (75 MHz, $CDCl_3$):** 158.2 (t, J^{C-F}= 30 Hz), 137.1, 131.6(q, J^{C-F}= 33 Hz), 128.7, 126.1 (q, J^{C-F}= 3.8 Hz), 123.9(q, J^{C-F}= 272 Hz) *Signals for carbons of the perfluoroalkyl group were not observed.* **^{19}F NMR (188 MHz, $CDCl_3$):** δ -63.0 (s, 3F), -81.0 (t, J^{F-F}= 9.5 Hz, 3F), -118.7(m, 2F), -123.6(m, 2F), -126.2(m, 2F). **HRMS** (APCI, -MS) calcd. for [M-H] $C_{13}H_5F_{12}O_2$ m/z: 421.0103, found: 421.0169. **R_f** = 0.49 (15% DCM/hexane).

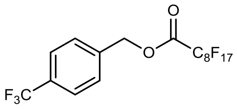

4-(trifluoromethyl)benzyl perfluorononanoate(**16c**).

Mixture of perfluorononanoic acid (2.9 g, 6.3 mmol), 4-trifluoromethylbenzyl alcohol (1.2 g, 0.95 ml, 7 mmol) and toluenesulfonic acid (173 mg, 1 mmol) in toluene (50 ml) was refluxed overnight with Dean-Stark trap. Evaporation of solvent and purification by column chromatography (eluent: 10% DCM in Hexane) gave the title compound in 77% yield (3.1 g).

^{1}H NMR (300 MHz, $CDCl_3$): δ 7.67 (d, J^{H-H}= 8.1 Hz, 2H), 7.51 (d, J^{H-H}= 8.1 Hz, 2H), 5.43 (s, 2H). **^{13}C NMR (75 MHz, $CDCl_3$):** δ 158.3 (t, J^{C-F}= 30 Hz), 137.1, 131.6(q, J^{C-F}= 33 Hz), 128.8, 126.0 (q, J^{C-F}= 3.8 Hz), 123.9(q, J^{C-F}= 272 Hz), 68.9. *Signals for carbons of the perfluoroalkyl group were not observed.* **^{19}F NMR (188 MHz, $CDCl_3$):** δ -63.1 (s, 3F), -81.0 (t, J^{F-F}= 10 Hz, 3F), -118.5(m, 2F), -

122.4 – -121.5(m, 6F), -122.8(m, 2F), -126.3(m, 2F). **HRMS** (APCI, -MS) calcd. for [M-H] $C_{17}H_5F_{20}O_2$ m/z: 620.9976, found: 621.0017. $\mathbf{R_f}$ = 0.50 (15% DCM/hexane).

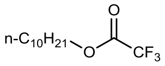

n-decyl trifluoroacetate(**16d**).

The title compound was synthesized similarly to **16a** using 3.2 g (20 mmol) of n-decanol. Evaporation of solvent gave *n*-decyl trifluoroacetate in quantitative yield (5.4g).

Spectroscopic data is similar to previously reported.[2]

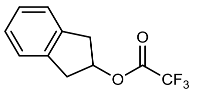

2-indanyl trifluoroacetate(**16e**).

The title compound was synthesized similarly to **16a** using 1.34 g (10 mmol) of 2-indanol. Evaporation of solvent gave 2-indanyl trifluoroacetate in quantitative yield (2.3 g).

^{1}H NMR (400 MHz, CDCl$_3$): v 7.08-7.35(m, 4H), 5.71(m, 1H), 3.41(dd, J^{H-H}= 17.3 Hz, 6.5 Hz, 2H), 3.41(dd, J^{H-H}= 17.3 Hz, 2.9 Hz, 2H). **^{13}C NMR (100 MHz, CDCl$_3$):** δ 157.6(q, J^{C-F}= 42.3 Hz), 139.3, 127.4, 124.8, 114.6(q, J^{C-F}= 286 Hz). **^{19}F NMR (188 MHz, CDCl$_3$):** δ -75.3.

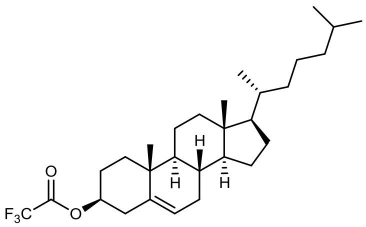

Cholesteryl trifluoroacetate(**16f**).

The title compound was synthesized similarly to **16a** using 1.93 g (5 mmol) of cholesterol. Evaporation of solvent gave 2-cholesteryl trifluoroacetate in quantitative yield (2.4 g).

Spectroscopic data is similar to previously reported.[3]

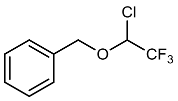

1-chloro-1-benzyloxy-2,2,2-trifluoroethane(**4a**).

The title compound was synthesized by combination of two literature modified processes.[4, 5] A solution of 4-(trifluoromethyl)benzyl trifluoroacetate (5.4 g, 20 mmol) in diethyl ether (20 mL) was added slowly at -78 °C to a suspension of $LiAlH_4$ (266 mg, 7 mmol) in diethyl ether (30 mL). The reaction mixture was stirred at this temperature for 3 h and then treated slowly with cooled H_2SO_4 (1M, 20 mL). The upper layer was separated and the aqueous phase was extracted with diethyl ether (2×20 ml). Combined organic phases were dried over Na_2SO_4, solvent evaporated. The residue was dissolved in 100 ml of dry dichloromethane, combined with triphenyl phosphite (7.4 g, 6.3 ml, 24 mmol) and cooled to 0°C with ice bath. To the obtained vigorously stirred solution in small portions was added NCS was added N-Chlorosuccinimide (4 g, 30 mmol) in small portions (Caution! Very exothermic!). Reaction mixture was removed from ice bath and stirred for 5 minutes. To the reaction mixture was added ~50 g of silica, solvent evaporated. Obtained mixture was subjected to column chromatography (eluent: hexane) giving 1-chloro-1-(4-trifluoromethyl)benzyloxy)-2,2,2-trifluoroethane as colorless liquid in 65% yield (2.63 g).

^{1}H NMR (300 MHz, $CDCl_3$): δ 7.36-7.48 (m, 5H), 5.58(q, J^{H-F} = 4.2 Hz, 1H), 5.02 (d, J^{H-H}= 11.9 Hz, 1H), 4.70 (d, J^{H-H}= 11.9 Hz, 1H). **^{13}C NMR (75 MHz, $CDCl_3$):** δ 134, 129.2, 129, 128.8, 121(q, J^{C-F}= 279 Hz), 87(q, J^{C-F}= 38.6 Hz), 72. **^{19}F NMR (188 MHz, $CDCl_3$):** δ -79.6 (d, J^{F-H} = 4.1 Hz, 3F). **R_f** = 0.20 (Hexane). **HRMS** (ESI, MS+) calcd. for [M+H] $C_9H_9ClF_3O$, m/z: 225.0294, found: 225.0297.

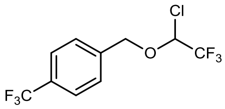

1-chloro-1-(4-trifluoromethyl)benzyloxy)-2,2,2-trifluoroethane (**4b**).

The title compound was synthesized similarly to **4a** using 4.9 g (18 mmol) of 4-(trifluoromethyl)benzyl trifluoroacetate (**16a**). After purification by column chromatography (eluent: hexane), 1-chloro-1-benzyloxy-2,2,2-trifluoroethane was obtained as colorless liquid in 71% yield (3.12 g)

^{1}H NMR (300 MHz, $CDCl_3$): δ 7.67 (d, J^{H-H}= 8 Hz, 2H), 7.5 (d, J^{H-H}= 8 Hz, 2H), 5.62 (q, J^{H-F} = 4.1 Hz, 1H), 5.06 (d, J^{H-H}= 12.3 Hz, 1H), 4.76 (d, J^{H-H}= 12.3 Hz, 1H). **^{13}C NMR (75 MHz, $CDCl_3$):** δ 138.3, 131.3(q, J^{C-F}= 33 Hz), 128.5, 126 (q, J^{C-F}= 3.8 Hz), 124(q, J^{C-F}= 271 Hz), 120.9(q, J^{C-F}= 278 Hz), 87.4(q, J^{C-F}= 39 Hz), 71.3. **^{19}F NMR (188 MHz, $CDCl_3$):** δ -62.9 (s, 3F), -79.6 (d, J^{F-H} = 4.1 Hz 3F). **R_f** = 0.24 (Hexane). **HRMS** (ESI, MS+) calcd. for [M+H] $C_{10}H_8ClF_6O$, m/z: 293.0168, found: 293.0162.

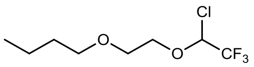

1-chloro-1-(2-(butoxy)ethoxy)-2,2,2-trifluoroethane(**4c**).

The mixture of 2-butoxy ethanol (1.18 g, 10 mmol), trifluoroacetaldehyde ethyl hemiacetal(1.92 g, 12 mmol) and 5 Å molecular sieves(10 g) in THF (20 ml) was stirred at the room temperature until no 2-butoxy ethanol was observed by ^{1}H NMR (*ca*. 5 days). Then molecular sieves were filtered off, solvent evaporated. The residue was dissolved in DCM (50 ml), combined with triphenyl phosphite (4.65 g, 4 ml, 15 mmol) and cooled to 0C with ice bath. To obtained vigorously stirred solution in small portions was added NCS (2.7 g, 20 mmol) in small portions (Caution! Very exothermic!). Reaction mixture was removed from ice bath and stirred for 5 minutes. To the reaction mixture was added ~50 g of silica, solvent evaporated. Obtained mixture was subjected to column chromatography (eluent: 1% to 3% EtOAc in hexane), giving 1-chloro-1-(2-(butoxy)ethoxy)-2,2,2-trifluoroethane as colorless liquid in 71% yield (2.5 g).

^{1}H NMR (300 MHz, $CDCl_3$): 5.88 (q, J^{H-F} = 4.25 Hz, 1H), 3.94-4.05 (m, 1H), 3.94-4.05 (m, 1H), 3.82-3.95(m, 1H), 3.65-3.71 (m, 2H), 3.74(t, J^{H-H}=6.6 Hz, 2H), 1.55(m, 2H), 1.35(m, 2H), 0.92(t, J^{H-H}= 7.3 Hz). **^{13}C NMR (75 MHz, $CDCl_3$):** δ 121(q, J^{C-F}= 280 Hz), 89.1(q, J^{C-F}= 39 Hz), 71.3, 71.5, 70.1, 69.5, 31.8, 19.3, 14.0. **^{19}F NMR (188 MHz, $CDCl_3$):** -79.9 (d, J^{F-H} = 4.3 Hz, 3F). **R_f** = 0.2 (10% DCM/Hexane). **HRMS** (ESI, MS+) calcd. for [M+Na] $C_8H_{14}ClF_3O_2Na$, m/z: 257.0567, found: 257.0532.

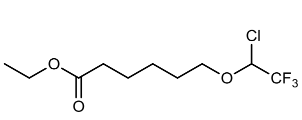

Ethyl 6-(1-chloro-2,2,2-trifluoroethoxy)hexanoate (**4d**).

The title compound was synthesized similarly to **4c**, using ethyl (6-hydroxy)hexanoate (1.6 g, 10 mmol). After column chromatography (eluent: 0.5%to 3% EtOAc in

hexane), Ethyl 6-(1-chloro-2,2,2-trifluoroethoxy)hexanoate was obtained as colorless liquid in 82% (2.25 g).

^{1}H NMR (300 MHz, CDCl$_3$): 5.62 (q, J^{H-F} = 4.1 Hz, 1H), 4.13 (q, J^{H-H} = 7.2 Hz, 2H), 3.96 (dt, J^{H-H}= 9.2 Hz, 6.4 Hz, 1H), 3.6 (dt, J^{H-H}= 9.2 Hz, 6.4 Hz, 1H), 2.31, (t, J^{H-H}=7.3 Hz, 2H) 1.59-1.76 (m, 4H), 1.36-1.49(m, 2H), 1.25 (t, J^{H-H}=7.1 Hz, 3H). **^{13}C NMR (75 MHz, CDCl$_3$):** δ 173.6, 120.9(q, J^{C-F}= 279 Hz), 88.9(q, J^{C-F}= 39 Hz), 71.4, 60.4, 34.2, 28.5, 25.4, 24.6, 14.4. **^{19}F NMR (188 MHz, CDCl$_3$):** -79.9 (d, J^{F-H} = 4.3 Hz, 3F). **R$_f$** = 0.2 (30% DCM/Hexane). **HRMS** (ESI, MS+) calcd. for [M+H] $C_{10}H_{17}ClF_3O_3$, m/z: 277.0834, found: 277.0818.

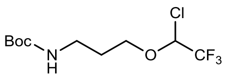

1-chloro-1-(3-(N-Bocamino)propoxy)-2,2,2-trifluoroethane(**4e**).

The title compound was synthesized similarly to **4d**, using 3-(N-Bocamino)propanol (1.75 g, 10 mmol). After column chromatography (eluent: 5% to 12% EtOAc in hexane), 1-chloro-1-(3-(N-Bocamino)propoxy)-2,2,2-trifluoroethane was obtained as colorless oil in 54% (1.57 g).

^{1}H NMR (300 MHz, CDCl$_3$): 5.64 (q, J^{H-F} = 4.1Hz, 1H), 4.64 (bs, 1H), 3.03 (dt, J^{H-H}= 9.6 Hz, 6.1 Hz, 1H), 3.69 (dt, J^{H-H}= 9.2 Hz, 6.4 Hz, 1H), 3.13-3.33 (m, 2H), 1.81-1.93 (m, 2H), 1.36-1.49 (m, 2H), 1.44 (s, 9H). **^{13}C NMR (75 MHz, CDCl$_3$):** δ 159.1, 120.9 (q, J^{C-F}= 279 Hz), 88.5 (q, J^{C-F}= 38.5 Hz), 69.4, 37.6, 29.3, 28.5. **^{19}F NMR (188 MHz, CDCl$_3$):** -79.8 (d, J^{F-H} = 4.1 Hz 3F). **R$_f$** = 0.18 (10% EtOAc in Hexane). **HRMS** (ESI, MS+) calcd for [M+H] $C_{10}H_{18}ClF_3NO_3$, m/z: 292.0922. found: 292.0996 .

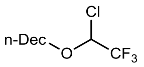

1-chloro-1-decyloxy-2,2,2-trifluoroethane(**4f**).

The title compound was synthesized similarly to **4a** using 4.08 g (18 mmol) *n*-decyl trifluoroacetate. After purification by column chromatography (eluent: hexane), 1-chloro-1-benzyloxy-2,2,2-trifluoroethane was obtained as colorless liquid in 76% yield (4.4 g).

^{1}H NMR (300 MHz, CDCl$_3$): 5.62(q, J^{H-F} = 4.1 Hz, 1H), 3.96 (dt, J^{H-H}= 9.3 Hz, 6.7 Hz, 1H), 3.61 (dt, J^{H-H}= 9.3 Hz, 6.4 Hz, 1H), 1.67 (m, 2H), 1.16-1.42 (m, 14H), 0.88 (t, J^{H-H}= 6.5 Hz, 3H). **^{13}C NMR (75 MHz, CDCl$_3$):** δ 120.9 (q, J^{C-F}= 279 Hz), 88.9 (q, J^{C-F}= 39.1 Hz), 71.9, 32, 29.65, 29.6, 29.4, 29.3, 28.7, 25.8, 22.8, 14.3. **^{19}F NMR (188 MHz, CDCl$_3$):** -79.9 (d, J^{F-H} = 4.1 Hz, 3F). **R$_f$** = 0.61 (Hexane). **HRMS** (ESI, -MS) calcd for [M-H] $C_{12}H_{23}ClF_3O$, m/z: 274.1311, found: 274.1318.

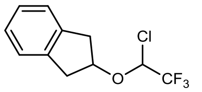

1-chloro-1-(2-indanoxy)-2,2,2-trifluoroethane(**4g**).

The title compound was synthesized similarly to **4a** using 2.3 g (10 mmol) of 4-(trifluoromethyl)benzyl trifluoroacetate (**16e**). After purification by column chromatography (eluent: hexane), 1-chloro-1-(2-indanoxy)-2,2,2-trifluoroethane was obtained as colorless liquid in 65% yield (1.52 g).

^{1}H NMR (400 MHz, CDCl$_3$): δ 7.16-7.26 (m, 4H), 5.76 (q, J^{H-F}= 4.3 Hz, 1H), 4.82 (tt, J^{H-H}= 6.6 Hz, 4.5 Hz, 1H), 3.33 (dd, J^{H-H}= 16.6 Hz, 6.7 Hz, 1H), 3.27 (dd, J^{H-H}= 16.3 Hz, 6.7 Hz, 1H), 3.2(dd, J^{H-H}= 16.6

Hz, 4.3 Hz, 1H), 3.06 (dd, J^{H-H}= 16.4 Hz, 4.3 Hz, 1H) . **^{13}C NMR (100 MHz, $CDCl_3$):** δ 140, 139.1, 127.3, 127.1, 124.81, 124.77, 120.9 (q, J^{C-F}= 280 Hz), 87.7 (q, J^{C-F}= 39 Hz), 82, 39.6, 38.5. **^{19}F NMR (188 MHz, $CDCl_3$):** δ -79.9 (d, J^{F-H}= 4.3 Hz 3F). **R_f** = 0.31 (Hexane). **HRMS** (ES MS-) calcd. for [M-H] $C_{11}H_{10}ClF_3O$, m/z: 249.0294, found: 249.0290.

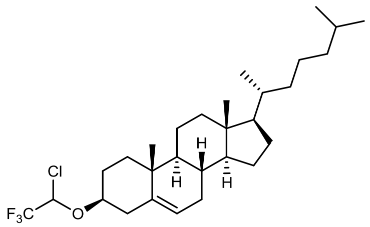

1-chloro-1-(cholesteryloxy)-2,2,2-trifluoroethane(mixture of diastereomers) (**4h**).

The title compound was synthesized similarly to **4a** using 4 g of (5 mmol) cholesteryl trifluoroacetate (**16f**). After purification by column chromatography (eluent: hexane), 1-chloro-1-benzyloxy-2,2,2-trifluoroethane was obtained as white solid in 70% yield (850 mg).

^{1}H NMR (500 MHz, $CDCl_3$): δ 5.75 (m, 1H), 5.41 (m, 1H), 3.70 (m, 1H), 2.25-2.45 (m, 1H), 1.87-2.06 (m, 4H), 1.79-1.87 (m, 1H), 1.43-1.73 (m, 7H), 1.21-1.41 (m, 4H), 1.02-1.2 (m, 7H), 1.01 (s, 3H), 0.935-1.00 (m, 2H), 0.91 (d, J^{H-H}= 6.6 Hz, 3H), 0.87 (d, J^{H-H}= 6.6 Hz, 3H), 0.86 (d, J^{H-H}= 6.6 Hz, 3H), 0.68 (s, 3H). **^{13}C NMR (125 MHz, $CDCl_3$):** δ 139.5, 139.2, 123.3, 123.2, 121.05(q, J^{C-F}= 280 Hz), 121.03(q, J^{C-F}= 280 Hz), 86.85(q, J^{C-F}= 38 Hz), 86.79(q, J^{C-F}= 38 Hz), 81, 80.9, 56.84, 56.83, 56.27, 50.20, 50.15, 42.5, 39.8, 39.7, 38.9, 37.6, 37.1, 35.84, 36.83, 36.76, 36.3, 36, 32.07, 32.04, 31.96, 28.6, 28.4, 28.2, 27, 24.4, 24, 23, 22.7, 21.21, 21.19, 19.4, 18.9, 12. **^{19}F NMR (188 MHz, $CDCl_3$):** δ -80.0 (d, J^{F-H} = 4.3 Hz, 3F). **R_f** = 0.49 (Hexane). **HRMS** (ES MS+) calcd. for [M+H] $C_{29}H_{47}ClF_3O$, m/z: 503.3268, found: 503.3215.

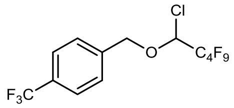

1-chloro-1-(4-trifluoromethyl)benzyloxy-1*H*-perfluoropentane (**4i**).

The title compound was synthesized similarly to **4b** using 4.2 g (10 mmol) of 4-(trifluoromethyl)benzyl perfluoropentanoate (**16b**). After purification by column chromatography (eluent: hexane), 1-chloro-1-benzyloxy-2,2,2-trifluoroethane was obtained as colorless liquid in 61% yield (2.7 g).

^{1}H NMR (400 MHz, $CDCl_3$): 7.58 (d, J^{H-H}= 8.2 Hz, 2H), 7.39 (d, J^{H-H}= 8.2 Hz, 2H), 5.78 (dm, J^{H-F} = 13.6 Hz, 1H), 4.99 (d, J^{H-H}= 11.9 Hz, 1H), 4.66 (d, J^{H-H}= 11.9 Hz, 1H). **^{13}C NMR (100 MHz, $CDCl_3$):** δ 137.9, 131.3(q, J^{C-F}= 32.5 Hz), 125.8, 126 (q, J^{C-F}= 3.8 Hz), 124(q, J^{C-F}= 272 Hz), 88.9 (dd, J^{C-F}= 34.8 Hz, 23.6 Hz), 71.7. *Signals for carbons of the perfluoroalkyl group were not observed.* **^{19}F NMR (188 MHz, $CDCl_3$):** -62.9(s, 3F), -80.9(t, J^{F-F} = 9.6 Hz, 3F), -116.3(dm, J^{F-F}= 283 Hz, 1F(α-F)), -120.9(dm, J^{F-F}= 300 Hz, 1F(γ-F)), -123.4(dm, J^{F-F}= 300 Hz, 1F(γ-F)), -125.1 (dm, J^{F-F}= 291 Hz, 1F(β-F)), -125.8(dm, J^{F-F}= 285.1 Hz, 1F(α-F)), -127.8(dm, J^{F-F}= 291 Hz, 1F(β-F)). **R_f** = 0.33 (Hexane). **HRMS** (APCI, -MS) calcd. for [M-H] $C_{13}H_6ClF_{12}O$, m/z: 440.9916, found: 442.0021.

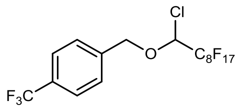

1-chloro-1-(4-trifluoromethyl)benzyloxy-1*H*-perfluorononane (**4j**).

The title compound was synthesized similarly to **4a** using 3.0 g (4.9 mmol) 4-(trifluoromethyl)benzyl perfluorononanoate (**16c**). After purification by

column chromatography (eluent: hexane), 1-chloro-1-benzyloxy-2,2,2-trifluoroethane was obtained as colorless liquid in 58% yield (1.8 g).

^{1}H NMR (300 MHz, $CDCl_3$): 7.66 (d, J^{H-H}= 8.1 Hz, 2H), 7.47 (d, J^{H-H}= 8.2 Hz, 2H), 5.86 (dm, J^{H-F} = 13.9 Hz, 1H), 5.07 (d, J^{H-H}= 12 Hz, 1H), 4.75 (d, J^{H-H}= 12 Hz, 1H). **^{13}C NMR (151 MHz, $CDCl_3$):** δ 137.9 (q, J^{C-F}= 1.2 Hz), 132.8 (q, J^{C-F}= 32.6 Hz), 128.6, 125.9 (q, J^{C-F}= 3.9 Hz), 124.0 (q, J^{C-F}= 272 Hz), 88.9 (dd, J^{C-F}= 35 Hz, 24 Hz), 71.7. *Signals for carbons of the perfluoroalkyl group were not observed.* **^{19}F NMR (188 MHz, $CDCl_3$):** -63.0 (s, 3F), -80.9 (t, J^{F-F} = 9.8 Hz, 3F), -116.3 (dm, J^{F-F} = 284 Hz, 1F), -120 – -124(m, 10F), -125.4(dm, J^{F-F} = 284 Hz, 1F), -125.7(dm, J^{F-F} = 297 Hz, 1F), -126.7 (dm, J^{F-F} = 297 Hz, 1F). **R_f** = 0.34 (Hexane). **HRMS** (APCI, MS-) calcd. for $C_{17}H_6ClF_{20}O$ [M-H], m/z: 641.9872, found: 641.9856.

Preparation of trimethoxy(aryl)silanes

Procedure **A**: a procedure was adapted from the literature as follow: 20 ml (~1 M) of a solution of aryl Grignard reagent in THF (prepared from 20 mmol (1 eq.) of the corresponding arylbromide and 530 mg, 22 mmol (1.1 eq.) of magnesium turnings by heating at 50°C for 3h.) was dropwise added to the ice-cold solution of 6 ml (2 eq.) tetramethylorthosilicate in hexanes (80 ml). The resulting mixture was stirred for 20h at room temperature. The obtained suspension was filtered through sintered glass filter, solvent removed under reduced pressure and the residue was purified by bulb-to-bulb distillation.

Procedure **B:** a procedure was adapted from literature[6] as follows: In a glovebox, a Schlenk tube was sequentially charged with $[Rh(cod)(MeCN)_2]BF_4$ (44 mg, 0.14 mmol, 2 mol%), DMF(7 ml), (hetero)aryl bromide (or iodide, 7 mmol, 1 eq.), tetrabutylammonium iodide (in case of aryl bromide is used, 2.6 g, 1 eq.), triethylamine (3 ml, 21 mmol, 3 eq.) and trimethoxysilane (1.35 ml, 10.5 mmol, 1.5 eq.). The mixture was heated at 80 °C for 3 h. Upon completion, volatiles and solvent were removed under reduced pressure; residue was dissolved in small amount of dichloromethane (*c.a.* 5 ml) and 100 ml of diethyl ether was added. Obtained mixture was filtered, concentrated under reduced pressure and subjected to bulb-to-bulb distillation.

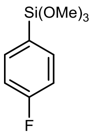

Trimethoxy(4-fluorophenyl)silane(**17a**): following a procedure **A**, 2.2 ml(20 mmol) of 1-bromo-4-fluorobenzene was used for the preparation of Grignard reagent. Product was obtained as colorless liquid, 1.51 g (31% yield).

Spectroscopic data corresponds to the previously reported.[7]

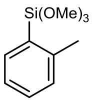

Trimethoxy(2-methylphenyl)silane(**17b**): following a procedure **A**, 2.4 ml (20 mmol) of 2-bromotoluene was used for the preparation of Grignard reagent. Product was obtained as colorless liquid, 1.53 g (36% yield).

Spectroscopic data corresponds to the previously reported.[8]

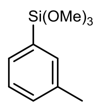

Trimethoxy(3-methylphenyl)silane(**17c**): following a procedure **A**, 2.4 ml (20 mmol) of 3-bromotoluene was used for the preparation of Grignard reagent. Product was obtained as a colorless liquid, 1.6 g (38% yield).

^{1}H NMR (600 MHz, $CDCl_3$): δ 7.47 (m, 1H), 7.44 (m, 1H), 7.26-7.31(m, 2H), 3.63(s, 9H), 2.37(s, 3H). **^{13}C NMR (125 MHz, $CDCl_3$):** δ 137.6, 135.4, 131.9, 131.6, 129.3, 128.1, 51.0, 21.6. **^{29}Si NMR (79 MHz, $CDCl_3$):** δ -54.2.

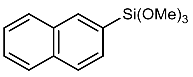

Trimethoxy(2-naphtyl)silane(**17d**): following a procedure **A**, 4.14 g (20 mmol) of 2-bromonaphthalene was used for the preparation of Grignard reagent. Product was obtained as colorless liquid, 1.84 g (35% yield).

^{1}H NMR (300 MHz, $CDCl_3$): δ 8.21 (s, 1H), 7.71-7.93 (m, 3H), 7.69(m, 1H), 7.46-7.57(m, 2H), 3.62(s, 9H), 2.49(s, 3H). **^{13}C NMR (100 MHz, $CDCl_3$):** δ 138.5, 132.8, 131.4, 130.4, 129.0, 51.0, 16.0. **^{29}Si NMR (79 MHz, $CDCl_3$):** δ -55.1.

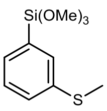

Trimethoxy(3-thiomethoxyphenyl)silane(**17e**): following a procedure **A**, 2.7 ml (20 mmol) of 3-bromothioanisole was used for the preparation of Grignard reagent. Product was obtained as colorless liquid, 1.58 g (33% yield).

^{1}H NMR (300 MHz, $CDCl_3$): δ 7.53 (m, 1H), 7.37-7.44 (m, 1H), 7.27-7.37(m, 2H), 3.67(m, 9H). **^{13}C NMR (100 MHz, $CDCl_3$):** δ 136.5, 134.6, 133.0, 130.3, 128.6, 127.9, 127.5, 127.1, 126.9, 126.2, 51.1. **^{29}Si NMR (79 MHz, $CDCl_3$):** δ -54.1.

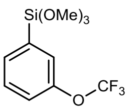

Trimethoxy(3-trifluoromethoxyphenyl)silane(**17f**): following a procedure **A**, 3 ml (20 mmol) of 3-(trilfluoromethoxy)bromobenzene was used for the preparation of Grignard reagent. Product was obtained as colorless liquid, 1.35 g (24% yield).

^{1}H NMR (300 MHz, $CDCl_3$): δ 7.56 (dm, J^{H-H}= 7.3 Hz, 1H), 7.48 (m, 1H), 7.43 (dd, J^{H-H}= 8.1 Hz, 7.3 Hz, 1H), 7.3 (dm, J^{H-H}= 8.1 Hz, 1H), 3.63(s, 9H). **^{13}C NMR (100 MHz, $CDCl_3$):** δ 149.3(q, J^{C-F}= 1 Hz), 133.2, 132.6, 129.8, 127.1, 123.3, 120.6(q, J^{C-F}= 257 Hz). **^{29}Si NMR (79 MHz, $CDCl_3$):** δ -56.5. **^{19}F NMR (376 MHz, $CDCl_3$):** δ -57.9 (s, 3F).

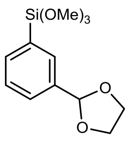

Trimethoxy(3-(1,3-dioxolan-2-yl)phenyl) silane(**17g**): following a procedure **A**, 4.6 g (20 mmol) of 3-(1,3-dioxolan-2-yl)bromobenzene was used for the preparation of Grignard reagent. Product was obtained as colorless liquid (2.2 g, 41% yield).

^{1}H NMR (400 MHz, $CDCl_3$): δ 7.76(m, 1H), 7.66(dt, J^{H-H}= 7.3 Hz, 1.2Hz, 1H), 7.58(dt, J^{H-H}= 7.8 Hz, 1.5 Hz, 1H), 7.42(dd, J^{H-H}=7.8 Hz, 7.3 Hz, 1H), 5.82(s, 1H), 4.09-4.17(m, 2H), 4.0-4.09(m, 2H), 3.62(s, 9H). **^{13}C NMR (100 MHz, $CDCl_3$):** δ 137.5, 135.8, 133.1, 129.7, 128.9, 128.2, 103.9, 65.5, 51. **^{29}Si NMR (79 MHz, $CDCl_3$):** δ -54.8.

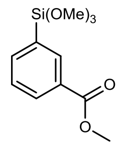

<u>Methyl 3-(trimethoxysilyl)benzoate</u>(**17h**): following a procedure **B**, using 1.5 g of methyl 3-bromobenzoate. Product was obtained as colorless oil (0.99 g, 56% yield).

^{1}H NMR (400 MHz, $CDCl_3$): δ 8.33(m, 1H), 8.12(dt, J^{H-H}= 7.8 Hz, 1.6 Hz, 1H), 7.84(dt, J^{H-H}= 7.3 Hz, 1.1 Hz, 1H), 7.48(m, 1H), 3.92(s, 3H), 3.64(s, 9H). **^{13}C NMR (100 MHz, $CDCl_3$):** δ 167.2, 139.3, 136, 131.9, 130.3, 129.9, 128.2, 55.3, 51.1. **^{29}Si NMR (79 MHz, $CDCl_3$):** δ -55.7.

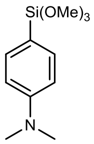

<u>Trimethoxy(4-dimethylaminophenyl) silane</u>(**17i**): following a procedure **B**, using 1.73 g of 4-(dimethylamino)iodobenzene. Product was obtained as white solid (m.p. = 23-27 °C), 423 mg (25% yield).

^{1}H NMR (500 MHz, $CDCl_3$): δ 7.51(d, J^{H-H}= 8.6 Hz, 2H), 6.73(d, J^{H-H}= 8.6 Hz, 2H), 3.60(s, 9H), 2.98(s, 6H). **^{13}C NMR (125 MHz, $CDCl_3$):** δ 152.1, 136.2, 131.9, 111.8, 50.9, 40.15. **^{29}Si NMR (99 MHz, $CDCl_3$):** δ -51.7.

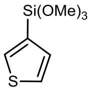

<u>Trimethoxy(3-thienyl) silane</u>(**17j**): following a procedure **A**, Grignard reagent was prepared by bromine-magnesium exchange of 3-bromothiophene (0.94 ml, 1.63 g, 10 mmol) with *i*PrMgBr·LiCl[9]. Product was obtained as colorless oil (702 mg, 34% yield).

^{1}H NMR (400 MHz, $CDCl_3$): δ 7.75(dd, J^{H-H}= 2.6 Hz, 1.1 Hz, 1H), 7.43(dd, J^{H-H}= 4.8 Hz, 2.6 Hz, 1H), 7.28 (dd, J^{H-H}= 4.8 Hz, 2.6 Hz, 1H), 3.62 (s, 9H). **^{13}C NMR (100 MHz, $CDCl_3$):** δ 136, 131.8, 130.1, 126.2, 50.9. **^{29}Si NMR (79 MHz, $CDCl_3$):** δ -56.5.

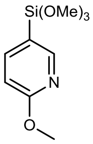

<u>Trimethoxy(2-methoxypyrid-5-yl) silane</u>(**17k**): following a procedure **B**, using 1.32 g of 5-bromo-2-methoxypyridine. Product was obtained as colorless oil (762 mg, 48% yield).

^{1}H NMR (400 MHz, $CDCl_3$): δ 8.39(m, 1H), 7.78(dd, J^{H-H}= 8.3 Hz, 1.9 Hz, 1H), 6.77(dd, J^{H-H}= 8.3 Hz, 0.7Hz, 1H), 3.69(s, 3H), 3.62(s, 9H). **^{13}C NMR (100 MHz, $CDCl_3$):** δ 165.8, 153.7, 144.7, 116.5, 111.1, 53.5, 51. **^{29}Si NMR (79 MHz, $CDCl_3$):** δ -54.2.

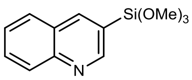

<u>Trimethoxy(3-quinolinyl) silane</u>(**17l**): following a procedure **B**, using 1.46 g of 3-bromoquinoline. Product was obtained as yellowish oil (793 mg, 45% yield).

^{1}H NMR (400 MHz, $CDCl_3$): δ 9.07(d, J^{H-H}= 1.7 Hz, 1H), 8.49(m, 1H), 8.12(d, J^{H-H}= 8.4 Hz, 1H), 7.85(ddd, J^{H-H}= 8.4 Hz, 6.9 Hz, 1.5 Hz, 1H), 7.57(m, 1H), 3.69(s, 3H), 3.62(s, 9H). **^{13}C NMR (100 MHz, $CDCl_3$):** δ 154.2, 149.1, 144.8, 130.7, 129.6, 128.3, 127.7, 126.8, 122.8, 51.1. **^{29}Si NMR (79 MHz, $CDCl_3$):** δ -55.8.

Preparation of trimethoxy(alkenyl) silanes

(2-phenylvinyl)- and (1-octenylvinyl)tirmethoxysilanes were synthesized by hydrosilylation reaction of phenylacatylene and octyne-1 respectively[10] followed by further methanolysis of corresponding (vinyl)trichlorosilanes[11] by known procedures.

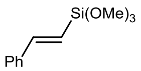

<u>(*E*)-2-phenyl)vinyltrimethoxysilane</u>(**17m**): 2.2 ml (2.04g, 20 mmol) of phenylacatylene was used. Short path distillation afforded the title compound as a colorless liquid in 2.06 g (9.2 mmol, 46% yield after two steps). Spectroscopic data corresponds to the previously reported.

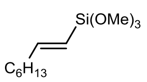

<u>(*E*)-trimethoxy(oct-1-en-1-yl)silane</u>(**17n**): 3 ml (2.2 g, 20 mmol) of octyne-1 used. Short path distillation afforded the title compound as a colorless liquid in 2.4 g (10.4 mmol, 52% yield after two steps). Note: some amount of (*E*)-trimethoxy(oct-1-en-2-yl)silane was obtained as a byproduct during hydrosilylation reaction, which does not interfere the reaction.

^{1}H NMR (400 MHz, $CDCl_3$): δ 6.45 (dd, $J^{H\text{-}H}$= 18.8 Hz, 6.4 Hz, 1H), 5.38 (dd, $J^{H\text{-}H}$= 1.4 Hz, 18.8 Hz, 1H), 3.57 (s, 1H), 2.17 (m, 2 H), 1.42(m, 2H), 1.29(m, 6H), 0.88(m, 3H). **^{13}C NMR (100 MHz, $CDCl_3$):** δ 155.1, 117.1, 50.7, 36.8, 31.8, 29.0, 29.3, 22.7, 14.8. **^{29}Si NMR (79 MHz, $CDCl_3$):** δ -53.5.

General Procedure for the Cross-coupling Reaction

In the glovebox, in 20 ml glass vial, mixture of $NiCl_2$·glyme (22 mg, 0.1 mmol) and ligand **7a** (50.5 mg, 0.11 mmol) in dry DMA (5 ml) were stirred for 1h. The obtained solution of catalyst was diluted with DMA (5 ml), then TBAT (1.35 g, 2.5 mmol) and trimethoxy(aryl) silane (1.3 mmol) were added to the vial, followed by the solution of the electrophile **4b** (293.6 mg, 1 mmol) in 5 ml of DMA. The vial was tightly closed with PVC tape and stirred outside of the glovebox with additional light irradiation (household white-light 10W LED lamp or blue-light 10W LED lamp with λ_{em}≈460 nm) for 16h. After completion of the reaction, a solution was poured into 15 ml 0.5M NaOH solution and stirred for additional 10 minutes. The obtained mixture was diluted with 45 ml of water and extracted with ether (3×20 ml). Combined organic fractions were washed with 10 ml of water, 10 ml of brine and dried over Na_2SO_4. The residue after solvent evaporation was subjected to column chromatography (silica gel, 230-400 mesh, hexane/DCM or hexane/EtOAc).

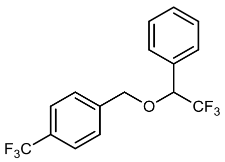

<u>1-phenyl-1-(4-trifluoromethyl)benzyloxy-2,2,2-trifluoroethane</u> (**6a**).

Used 258 mg (243 µl) of trimethoxy(phenyl) silane. Product isolated as colorless oil.

Run 1: 310 mg, 93% yield (96% by ^{19}F NMR), 97% *ee*. **Run 2:** 308 mg, 93% yield (96% by ^{19}F NMR), 97% *ee*.

^{1}H NMR (500 MHz, $CDCl_3$): δ 7.62(d, J^{H-H}= 8.2Hz, 2H), 7.48-7.40(m, 7H), 4.71(d, J^{H-H}= 12.5Hz, 1H), 4.66(q, J^{H-H} = 6.4Hz, 1H), 4.55(d, J^{H-H}= 12.5Hz, 1H). **^{13}C NMR (125 MHz, $CDCl_3$):** δ 140.8, 132.3, 130.4(q, J^{C-F}= 33 Hz), 129.9, 128.9, 128.5, 127.9, 125.7 (q, J^{C-F}= 3.8 Hz), 124.2(q, J^{C-F}= 272 Hz), 123.9(q, J^{C-F}= 281 Hz), 79.2(q, J^{C-F}= 32 Hz), 70.9. **^{19}F NMR (188 MHz, $CDCl_3$):** δ -62.8 (s, 3F), -76.6 (d, J^{F-H} = 6.9 Hz 3F). **R_f** = 0.36 (10% DCM/Hexane). **HRMS**(APCI, -MS) calcd. for $C_{16}H_{11}F_6O$ [M-H] m/z: 333.0714, found: 333.0786. **HPLC**: Daicel CHIRALPAK OJ-H column, 7% IPA/Hexane, flow 0.75ml/min; (4R,5S)-ligand: 17.9 min (major), 36.8 min (minor).

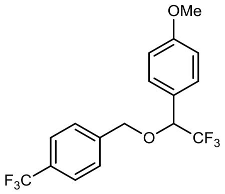

1-(4-methoxyphenyl)-1-(4-trifluoromethyl)benzyloxy-2,2,2-trifluoroethane(**6b**).

Used 297 mg of (268 µl) trimethoxy(4-methoxyphenyl) silane. Product isolated as colorless oil.

Run 1: 332 mg, 91% yield (95% by ^{19}F NMR), 97% *ee*. **Run 2:** 336 mg, 92% yield (94% by ^{19}F NMR), 97% *ee*.

^{1}H NMR (300 MHz, $CDCl_3$): δ 7.62(d, J^{H-H}= 8.3 Hz, 2H), 7.44(d, J^{H-H}= 8.3 Hz, 2H), 7.37(d, J^{H-H}= 8.6 Hz, 2H), 6.95(d, J^{H-H}= 8.6 Hz, 2H), 4.69 (d, J^{H-H}= 12.4 Hz, 1H), 4.61(q, J^{H-H}= 6.6 Hz, 1H), 4.51(d, J^{H-H}= 12.4 Hz, 1H). **^{13}C NMR (75 MHz, $CDCl_3$):** δ 160.9, 141, 130.4(q, J^{C-F}= 33 Hz), 129.8, 127.9, 125.6(q, J^{C-F}= 3.4 Hz), 124.2(q, J^{C-F}= 271 Hz), 124.1, 123.9(q, J^{C-F}= 281 Hz), 114.3, 78.8(q, J^{C-F}= 32 Hz), 70.6, 55.5 **^{19}F NMR (188 MHz, $CDCl_3$):** δ -62.8 (s, 3F), -76.8 (d, J^{F-H} = 6.6 Hz 3F). **R_f** = 0.17 (10% DCM/hexane). **HRMS**(APCI, +MS) calcd. for $C_{17}H_{14}F_6O_2$ [M+] m/z: 364.0893, found: 364.0910. **HPLC**: Daicel CHIRALPAK OJ-H column, 7% IPA/Hexane, flow 0.75ml/min; (4R,5S)-ligand: 17.6 min (major), 33.1 min (minor).

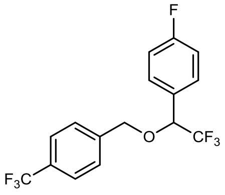

1-(4-fluorophenyl)-1-(4-trifluoromethyl)benzyloxy-2,2,2-trifluoroethane(**6c**).

Used 281 mg of trimethoxy(4-fluorophenyl) silane. Product isolated as colorless oil.

Run 1: 323 mg, 90% yield (95% by ^{19}F NMR), 96% *ee*. **Run 2:** 326 mg, 93% yield (96 by ^{19}F NMR), 96% *ee*.

^{1}H NMR (300 MHz, $CDCl_3$): δ 7.62(d, J^{H-H}= 8.1 Hz, 2H), 7.47-7.40 (m, 4H), 7.12 (m, 2H), 7.24(d, J^{H-H}= 8.1 Hz, 2H), 4.71 (d, J^{H-H}= 12.4 Hz, 1H), 4.66(q, J^{H-H}= 6.2 Hz, 1H), 4.55(d, J^{H-H}= 12.4 Hz, 1H). **^{13}C NMR (75 MHz, $CDCl_3$):** δ 165.2(d, J^{C-F}= 249 Hz), 140.6, 131.2(q, J^{C-F}= 33 Hz), 130.3(d, J^{C-F}= 8 Hz), 128.1, 127.9, 125.7(q, J^{C-F}= 4 Hz), 124.2(q, J^{C-F}= 272 Hz), 123.7(q, J^{C-F}= 281 Hz), 116(d, J^{C-F}= 22 Hz) 78.5(q, J^{C-F}= 32 Hz), 71. **^{19}F NMR (188 MHz, $CDCl_3$):** δ -62.8 (s, 3F), -76.8 (d, J^{F-H} = 6.2 Hz 3F), -111.4(m, 1H). **R_f** = 0.36 (10% DCM/Hexane). **HRMS**(APCI, -MS) calcd. for $C_{16}H_{10}F_7O$ [M-H] m/z: 351.0609, found: 351.0620. **HPLC**: Daicel CHIRALPAK OJ-H column, 7% IPA/Hexane, flow 0.75ml/min; (4R,5S)-ligand: 13.1 min (major), 30 min (minor).

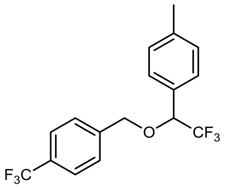

1-(*p*-tolyl)-1-(4-trifluoromethyl)benzyloxy-2,2,2-trifluoroethane (**6d**).

Used 276 mg (264 µl) of trimethoxy(p-tolyl) silane. Product isolated as colorless oil.

Run 1: 324 mg, 93% yield (97% by ^{19}F NMR), 96% *ee*. **Run 2:** 317 mg, 91% yield (95% by ^{19}F NMR), 97% *ee*.

^{1}H NMR (300 MHz, $CDCl_3$): δ 7.61(d, J^{H-H}= 8.1 Hz, 2H), 7.43(d, J^{H-H}= 8.1 Hz, 2H), 7.33(d, J^{H-H}= 8.1 Hz, 2H), 7.24(d, J^{H-H}= 8.1 Hz, 2H), 4.69 (d, J^{H-H}= 12.4 Hz, 1H), 4.63(q, J^{H-H} = 6.5 Hz, 1H), 4.52(d, J^{H-H}= 12.4 Hz, 1H), 2.39 (s, 3H). **^{13}C NMR (75 MHz, $CDCl_3$):** δ 140.9, 140, 130.2, 129.6, 129.2, 128.4(q, J^{C-F}= 31 Hz), 127.8, 125.6, 124.2(q, J^{C-F}= 271 Hz), 123.9(q, J^{C-F}= 281 Hz), 79.1(q, J^{C-F}= 32 Hz), 70.6, 21.4 **^{19}F NMR (188 MHz, $CDCl_3$):** δ -62.8 (s, 3F), -76.7 (d, J^{F-H} = 6.4 Hz, 3F). **R_f** = 0.37 (10% DCM/Hexane). **HRMS**(APCI, -MS) calcd. for $C_{17}H_{13}F_6O$ [M-H] m/z: 347.0871, found: 347.0803. **HPLC**: Daicel CHIRALPAK OJ-H column, 7% IPA/Hexane, flow 0.75ml/min; (4R,5S)-ligand: 9.9 min (major), 25.2 min (minor).

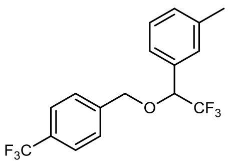

1-(*m*-tolyl)-1-(4-trifluoromethyl)benzyloxy-2,2,2-trifluoroethane (**6e**).

Used 276 mg (264 µl) of trimethoxy(m-tolyl) silane. Product isolated as colorless oil.

Run 1: 317 mg, 91% yield (95% by ^{19}F NMR), 96% *ee*. **Run 2:** 309 mg, 89% yield (94% by ^{19}F NMR), 97% *ee*.

^{1}H NMR (500 MHz, $CDCl_3$): δ 7.65(d, J^{H-H}= 8 Hz, 2H), 7.47(d, J^{H-H}= 8 Hz, 2H), 7.37-7.32(m, 1H), 7.32-7.24(m, 3H), 4.73 (d, J^{H-H}= 12.6 Hz, 1H), 4.66(q, J^{H-H}= 6.3 Hz, 1H), 4.56(d, J^{H-H}=12.6 Hz, 1H), 2.42(s, 3H). **^{13}C NMR (125 MHz, $CDCl_3$):** δ 140.9, 138.7, 132.2, 130.7, 130.4(q, J^{C-F}= 32 Hz), 129.1, 128.8, 127.8, 125.65(q, J^{C-F}= 3.9 Hz), 126, 124.2(q, J^{C-F}= 273 Hz), 123.9(q, J^{C-F}= 281 Hz), 79.2(q, J^{C-F}= 31 Hz), 70.8, 21.5 **^{19}F NMR (188 MHz, $CDCl_3$):** δ -62.8 (s, 3F), -76.5 (d, J^{F-H} = 6.5 Hz 3F). **R_f** = 0.36 (10% DCM/Hexane). **HRMS**(APCI, -MS) calcd. for $C_{17}H_{13}F_6O$ [M-H] m/z: 347.0871, found: 347.0803. **HPLC**: Daicel CHIRALPAK OJ-H column, 7% IPA/Hexane, flow 0.75ml/min; (4R,5S)-ligand: 10.4 min (major), 32.8 min (minor).

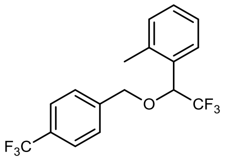

1-(*o*-tolyl)-1-(4-trifluoromethyl)benzyloxy-2,2,2-trifluoroethane (**6f**).

Used 276 mg (264 µl) of trimethoxy(o-tolyl) silane. Note: reaction time 48h. Product isolated as colorless oil.

Run 1: 277 mg, 80% yield (83% by ^{19}F NMR), 92% *ee*. **Run 2:** 275 mg, 79% yield (83% by ^{19}F NMR), 92% *ee*.

^{1}H NMR (300 MHz, $CDCl_3$): δ 7.59-7.65(m, 3H), 7.43(d, J^{H-H}= 8.3 Hz, 2H), 7.27-7.34(m, 2H), 7.32-7.24(m, 2H), 7.18-7.24(m, 1H), 4.98 (q, J^{H-H}= 6.5 Hz, 1H), 4.69(d, J^{H-H} = 12.5 Hz, 1H), 4.50(d, J^{H-H}=12.6 Hz, 1H), 2.28(s, 3H). **^{13}C NMR (125 MHz, $CDCl_3$):** δ 140.9, 137.6, 130.9, 130.6,

130.5(q, J^{C-F}= 32 Hz), 129.6, 128.1, 127.9, 125.7(q, J^{C-F}= 3.8 Hz), 124.3(q, J^{C-F}= 282 Hz), 124.2(q, J^{C-F}= 272 Hz), 75(q, J^{C-F}= 31 Hz), 70.7, 19.4(q, J^{C-F}= 1 Hz) **^{19}F NMR (188 MHz, $CDCl_3$):** δ -62.8 (s, 3F), -76.2 (d, J^{F-H} = 6.4 Hz 3F). **R_f** = 0.36 (10% DCM/Hexane). **HRMS**(APCI, -MS) calcd. for $C_{17}H_{13}F_6O$ [M-H] m/z: 347.0871, found: 347.0803. **HPLC**: Daicel CHIRALPAK OJ-H column, 7% IPA/Hexane, flow 0.75ml/min; (4R,5S)-ligand: 11.2 min (major), 24.9 min (minor).

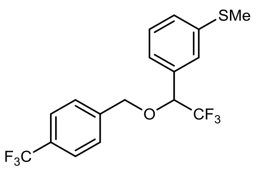

1-(3-thiomethylphenyl)-1-(4-trifluoromethyl)benzyloxy-2,2,2-trifluoroethane(**6g**).

Used 318 mg of trimethoxy(3-thiomethylphenyl) silane. For workup instead of 0.5M NaOH solution, 1M TBAF in THF was used. Product isolated as colorless oil.

Run 1: 348 mg, 92% yield (97% by ^{19}F NMR), 95% *ee*. **Run 2:** 351 mg, 93% yield (97% by ^{19}F NMR), 95% *ee*.

^{1}H NMR (500 MHz, $CDCl_3$): δ 7.64(d, J^{H-H}= 7.9 Hz, 2H), 7.46(d, J^{H-H}= 7.9 Hz, 2H), 7.38-7.31(m, 3H), 7.22(d, J^{H-H}= 7.3 Hz, 1H), 4.73 (d, J^{H-H}= 12.6 Hz, 1H), 4.65(q, J^{H-H} = 6.3 Hz, 1H), 4.56(d, J^{H-H}=12.6 Hz, 1H), 2.51(s, 3H). **^{13}C NMR (125 MHz, $CDCl_3$):** δ 140.7, 149.7, 133, 130.2(q, J^{C-F}= 32.3 Hz), 129.3, 127.9, 127.7, 126.1, 125.7(q, J^{C-F}= 3.4 Hz), 125, 124.2(q, J^{C-F}= 272 Hz), 123.7(q, J^{C-F}= 282 Hz), 79.0(q, J^{C-F}= 32 Hz), 71, 15.7 **^{19}F NMR (188 MHz, $CDCl_3$):** δ -62.8 (s, 3F), -76.5 (d, J^{F-H} = 6.4 Hz 3F). **R_f** = 0.20 (10% DCM/hexane). **HRMS**(APCI, +MS) calcd. for $C_{17}H_{13}F_6OS$ [M+] m/z: 380.0664, found: 380.0723. **HPLC**: Daicel CHIRALPAK OJ-H column, 7% IPA/Hexane, flow 0.75ml/min; (4R,5S)-ligand: 10.9 min (major), 14.5 min (minor).

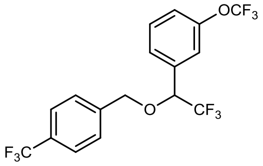

1-(3-trifluoromethoxyphenyl)-1-(4-trifluoromethyl)benzyloxy-2,2,2-trifluoroethane(**6h**).

Used 367 mg of trimethoxy(3-trifluoromethoxyphenyl) silane. Product isolated as colorless oil.

Run 1: 362 mg, 87% yield (96% by ^{19}F NMR), 94% *ee*. **Run 2:** 363 mg, 93% yield (96% by ^{19}F NMR), 94% *ee*.

^{1}H NMR (300 MHz, $CDCl_3$): δ 7.63.(d, J^{H-H}= 8.1 Hz, 2H), 7.24-7.5(m, 6H), 4.74 (d, J^{H-H}= 12.5 Hz, 1H), 4.69(q, J^{H-H} = 6.4 Hz, 1H), 4.59(d, J^{H-H}=12.6 Hz, 1H). **^{13}C NMR (125 MHz, $CDCl_3$):** δ 149.7, 140.4, 134.8, 130.7(q, J^{C-F}= 32.5 Hz), 130.5, 128, 126.8, 125.8(q, J^{C-F}= 3.7 Hz), 124.1(q, J^{C-F}= 272 Hz), 123.5(q, J^{C-F}= 282 Hz), 122.4, 120.9, 120.6(q, J^{C-F}= 257 Hz), 78.5 (q, J^{C-F}= 32 Hz), 71.5 **^{19}F NMR (188 MHz, $CDCl_3$):** δ -58.1 (s, 3F), -62.8 (s, 3F), -76.6 (d, J^{F-H}= 6.4 Hz 3F). **R_f** = 0.41 (10% DCM/hexane). **HRMS**(APCI, +MS) calcd. for $C_{17}H_{12}F_9O_2$ [M+] m/z: 417.0539, found: 417.0532. **HPLC**: Daicel CHIRALPAK OJ-H column, 0.5% IPA/Hexane, flow 0.75ml/min; (4R,5S)-ligand: 11.9 min (major), 20.4 min (minor).

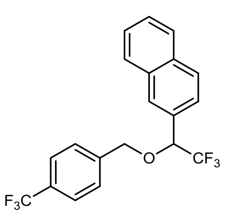

1-(2-naphtyl)-1-(4-trifluoromethyl)benzyloxy-2,2,2-trifluoroethane (**6i**).

Used 323 mg of trimethoxy(2-naphtyl) silane. Product isolated as colorless oil.

Run 1: 365 mg, 95% yield (98% by ^{19}F NMR), 96% *ee*. **Run 2:** 360 mg, 94% yield (97% by ^{19}F NMR), 96% *ee*.

^{1}H NMR (300 MHz, CDCl$_3$): δ 7.84-7.96(m, 4H), 7.51-7.62(m, 5H), 7.37-7.32(m, 1H), 7.45(d, J^{H-H}= 8.1 Hz, 2H), 4.84(q, J^{H-H} = 6.6 Hz, 1H), 4.75(d, J^{H-H}= 12.4 Hz, 1H), 4.58 (d, J^{H-H} = 12.4 Hz, 1H). **^{13}C NMR (151 MHz, CDCl$_3$):** δ 140.8, 134.1, 133.1, 130.5(q, J^{C-F}= 32 Hz), 129.6, 128.9, 128.8, 128.3, 128, 127.9, 127.2, 126.8, 125.7(q, J^{C-F}= 3.8 Hz), 125, 126, 124.2(q, J^{C-F}= 273 Hz), 124(q, J^{C-F}= 281 Hz), 79.3(q, J^{C-F}= 31 Hz), 70.9.**^{19}F NMR (188 MHz, CDCl$_3$):** δ -62.8 (s, 3F), -76.3 (d, J^{F-H} = 6.6 Hz 3F). **R$_f$** = 0.33 (10% DCM/Hexane). **HRMS**(APCI, +MS) calcd. for $C_{20}H_{15}F_6O$ [M+H] m/z: 384.0943, found: 384.0934. **HPLC**: Daicel CHIRALPAK OJ-H column, 7% IPA/Hexane, flow 0.75ml/min; (4R,5S)-ligand: 19.1 min (major), 27.7 min (minor).

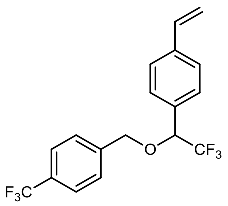

1-(4-vinylphenyl)-1-(4-trifluoromethyl)benzyloxy-2,2,2-trifluoroethane(**6j**).

Used 291 mg (275 μL) of trimethoxy(4-vinylphenyl) silane. Product isolated as colorless oil.

Run 1: 199 mg, 54% yield (73% by ^{19}F NMR), 95% *ee*. **Run 2:** 205 mg, 57% yield (72% by ^{19}F NMR), 96% *ee*.

^{1}H NMR (300 MHz, CDCl$_3$): 7.62(d, 2H, J^{H-H}= 8.2 Hz, 7.50-7.38(m, 6H), 6.75(dd, J^{H-H}= 17.5 Hz, 10.9 Hz, 1H), 5.81(dd, J^{H-H}= 17.5 Hz, 6.9 Hz, 1H), 5.33(d, J^{H-H}= 10.9 Hz, 1H), 4.71(d, J^{H-H}= 12.5 Hz, 1H), 4.66(q, J^{H-F}= 6.6 Hz, 1H), 4.54(d, J^{H-H}= 12.5 Hz, 1H). **^{13}C NMR (75 MHz, CDCl$_3$):** δ 140.8, 139.3, 136.2, 131.6, 130.5(q, J^{C-F}= 33 Hz), 128.7, 127.9, 126.7, 125.7(q, J^{C-F}= 3.8 Hz), 124.2(q, J^{C-F}= 282 Hz), 123.8(q, J^{C-F}= 273 Hz), 115.4, 79(q, J^{C-F}= 32 Hz), 70.9. **^{19}F NMR (188 MHz, CDCl$_3$):** δ -62.8 (s, 3F), -76.6 (d, J^{F-H} = 6.5 Hz 3F). **R$_f$** = 0.35 (10% DCM/Hexane). **HRMS**(APCI, +MS) calcd. for $C_{18}H_{15}F_6O$ [M+H] m/z: 361.1022, found: 361.1015. **HPLC**: Daicel CHIRALPAK OJ-H column, 7% IPA/Hexane, flow 0.75ml/min; (4R,5S)-ligand: 14.0 min (major), 29.9 min (minor).

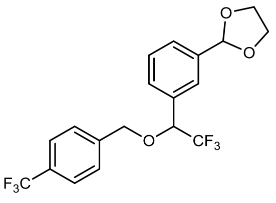

1-(3-(1,3-dioxaolan-2-yl)phenyl)-1-(4-trifluoromethyl)benzyloxy-2,2,2-trifluoroethane(**6k**).

Used 351 mg of trimethoxy(3-(1,3-dioxolan-2-yl)phenyl) silane. Product isolated as white solid.

Run 1: 360 mg, 89% yield (93% by ^{19}F NMR), 95% *ee*. **Run 2:** 362 mg, 89% yield (95% by ^{19}F NMR), 96% *ee*.

^{1}H NMR (500 MHz, CDCl$_3$): δ 7.62(d, J^{H-H}= 8.1 Hz, 2H), 7.54-7.58(m, 2H), 7.45-7.49(m, 2H), 7.43(d, J^{H-H}= 8 Hz, 2H), 5.83, (s, 1H), 4.66-4.72 (m, 2H), 4.54(d, J^{H-H}= 12.4 Hz, 1H), 4.10-4.18(m, 2H), 4.02-4.18(m, 2H). **^{13}C NMR (125 MHz, CDCl$_3$):** δ 140.8, 138.8, 132.5, 130.4(q, J^{C-F}= 32.5 Hz), 129.2, 129.1, 128.2, 127.6, 126.7, 126.7(q, J^{C-F}= 3.8 Hz), 124.2(q, J^{C-F}= 272 Hz), 123.8(q, J^{C-F}= 282 Hz), 103.4, 79.1(q, J^{C-F}= 31.4 Hz), 71.0, 65.5. **^{19}F NMR (188 MHz, CDCl$_3$):** δ -62.7 (s, 3F), -76.4 (d, J^{F-H}= 6.5 Hz, 3F). **R$_f$** = 0.24 (30% DCM/hexane). **HRMS**(APCI, -MS) calcd. for [M-H] $C_{19}H_{15}F_6O_3$ m/z: 405.0931, found: 405.1138. **HPLC**: Daicel CHIRALPAK OJ-H column, 7% IPA/Hexane, flow 0.75ml/min; (4R,5S)-ligand: 12.1 min (major), 13.8 min (minor).

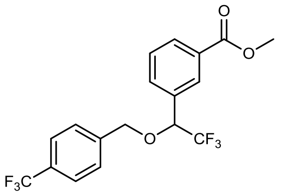

1-(3-(1,3-dioxalen-2-yl)phenyl)-1-(4-trifluoromethyl)benzyloxy-2,2,2-trifluoroethane(**6l**).

Used 333 mg of methyl (3-trimethoxysilyl)benzoate. Product isolated as colorless oil.

Run 1: 352 mg, 90% yield (95% by ^{19}F NMR), 93% *ee*. **Run 2:** 348mg, 89% yield (94% by ^{19}F NMR), 95% *ee*.

^{1}H NMR (500 MHz, CDCl$_3$): δ 8.08-8.14(m, 2H), 7.66 (d, J^{H-H}= 7.7 Hz, 1H), 7.62(d, J^{H-H}= 7.9 Hz, 2H), 7.52(m, 1H), 7.44(d, J^{H-H}= 7.9 Hz, 2H), 4.70-4.76 (m, 2H), 4.58(d, J^{H-H}= 12.6 Hz, 1H), 3.94(s, 3H). **^{13}C NMR (125 MHz, CDCl$_3$):** δ 166.6, 140.5, (q, J^{C-F}= 1.1 Hz), 132.9(q, J^{C-F}= 1.1 Hz), 132.6, 131.1, 131.0, 130.6(q, J^{C-F}= 32.4 Hz), 129.7, 129.2, 127.9, 127.7 (q, J^{C-F}= 3.8 Hz), 124.2(q, J^{C-F}= 272 Hz), 123.7(q, J^{C-F}= 282 Hz), 78.8(q, J^{C-F}= 31.6 Hz), 71.3, 52.5. **^{19}F NMR (188 MHz, CDCl$_3$):** δ -62.8 (s, 3F), -76.5 (d, J^{F-H}= 6.3 Hz, 3F). **R$_f$** = 0.14 (20% DCM/hexane). **HRMS**(APCI, +MS) calcd. for [M+H] $C_{18}H_{15}F_6O_3$ m/z: 393.0920, found: 393.0906. **HPLC**: Daicel CHIRALPAK OD-H column, 5% IPA/Hexane, flow 0.75ml/min; (4R,5S)-ligand: 10.3 min (major), 14.4 min (minor).

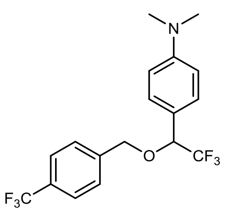

1-(4-(dimethylamino)phenyl)-1-(4-trifluoromethyl)benzyloxy-2,2,2-trifluoroethane(**6m**).

Used 314 mg of trimethoxy(4-(dimethylamino)phenyl) silane. Product isolated as colorless oil.

Run 1: 325 mg, 86% yield (94% by ^{19}F NMR), 96% *ee*. **Run 2:** 333 mg, 88% yield (96% by ^{19}F NMR), 96% *ee*.

^{1}H NMR (500 MHz, CDCl$_3$): δ 7.61(d, J^{H-H}= 8.1 Hz, 2H), 7.44 (d, J^{H-H}= 8.1 Hz, 2H), 7.29(d, J^{H-H}=8.7, 2H), 6.74(d, J^{H-H}= 8.7 Hz, 2H), 4.68, (d, J^{H-H}= 12.5 Hz, 2H), 4.56(q, J^{H-F}= 6.5 Hz, 1H), 4.48(d, J^{H-H}= 12.5 Hz, 1H), 2.996(s, 6H). **^{13}C NMR (125 MHz, CDCl$_3$):** δ 151.5, 141.3(q, J^{C-F}= 1.3 Hz), 130.2(q, J^{C-F}= 32 Hz), 129.5, 127.9, 125.6(q, J^{C-F}= 3.8 Hz), 124.2(q, J^{C-F}= 272 Hz), 124.1(q, J^{C-F}= 281 Hz), 118.9(q, J^{C-F}= 1.1 Hz), 78.9(q, J^{C-F}= 31.7 Hz), 70.1, 40.4. **^{19}F NMR (188 MHz, CDCl$_3$):** δ -62.7 (s, 3F), -76.8 (d, J^{F-H} = 6.5 Hz, 3F). **R$_f$** = 0.2 (20% DCM/hexane). **HRMS**(APCI, +MS) calcd. for [M+H] $C_{18}H_{18}F_6NO$ m/z: 378.1287, found: 378.1269. **HPLC**: Daicel CHIRALPAK OJ-H column, 7% IPA/Hexane, flow 0.75ml/min; (4R,5S)-ligand: 16.5 min (major), 20.4 min (minor).

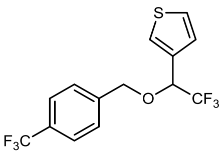

1-(3-thienyl)-1-(4-trifluoromethyl)benzyloxy-2,2,2-trifluoroethane (**6n**).

Used 266 mg of trimethoxy(3-thienyl) silane. Product isolated as colorless oil.

Run 1: 292 mg, 86% yield (92% by ^{19}F NMR), 96% *ee*. **Run 2:** 294 mg, 86% yield (90% by ^{19}F NMR), 96% *ee*.

^{1}H NMR (500 MHz, $CDCl_3$): δ 7.62(d, $J^{H\text{-}H}$= 8.1 Hz, 2H), 7.41-7.46(m, 3H), 7.40(dd, $J^{H\text{-}H}$= 5.1 Hz, 3 Hz, 1H), 7.17(d, $J^{H\text{-}H}$= 5.1 Hz, 1H), 4.8 (q, $J^{H\text{-}F}$= 6.5 Hz, 1H), 4.71(d, $J^{H\text{-}H}$= 12.6 Hz, 1H), 4.57(d, $J^{H\text{-}H}$= 12.6 Hz, 1H). **^{13}C NMR (125 MHz, $CDCl_3$):** δ 140.8, 133.4, 130.5(q, $J^{C\text{-}F}$= 32.4 Hz), 127.9, 127, 126.7, 126.3, 125.7(q, $J^{C\text{-}F}$= 3.8 Hz), 124.2(q, $J^{C\text{-}F}$= 272 Hz), 123.7(q, $J^{C\text{-}F}$= 282 Hz), 73.5(q, $J^{C\text{-}F}$= 31.1 Hz), 70.9. **^{19}F NMR (188 MHz, $CDCl_3$):** δ -62.7 (s, 3F), -76.5 (d, $J^{F\text{-}H}$ = 6.5 Hz, 3F). **R_f** = 0.27 (hexane). **HRMS**(APCI, -MS) calcd. for [M-H] $C_{14}H_9F_6OS$ m/z: 339.0284, found: 339.0287. **HPLC**: Daicel CHIRALPAK OJ-H column, 1% IPA/Hexane, flow 0.75ml/min; (4R,5S)-ligand: 11.0 min (major), 17.3 min (minor).

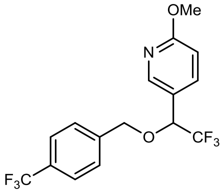

1-(2-methoxypyrid-5-yl)-1-(4-trifluoromethyl)benzyloxy-2,2,2-trifluoroethane(**6o**).

Used 298 mg of trimethoxy(2-methoxypyrid-5-yl) silane. Product isolated as colorless oil.

Run 1: 270 mg, 74% yield (85% by ^{19}F NMR), 86% *ee*. **Run 2:** 265 mg, 73% yield (82% by ^{19}F NMR), 87% *ee*.

^{1}H NMR (400 MHz, $CDCl_3$): δ 8.15(d, $J^{H\text{-}H}$= 2.3 Hz, 1H), 7.7(dd, $J^{H\text{-}H}$= 8.6 Hz, 2.3 Hz, 1H), 7.62(d, $J^{H\text{-}H}$= 8 Hz, 2H), 7.43(d, $J^{H\text{-}H}$= 8 Hz, 2H), 6.82 (d, $J^{H\text{-}H}$= 8.6 Hz), 4.7 (d, $J^{H\text{-}H}$= 12.5 Hz, 1H), 4.63(q, $J^{H\text{-}F}$ = 6.5 Hz, 1H), 4.56(d, $J^{H\text{-}H}$= 12.5 Hz, 1H), 3.97, (s, 3H). **^{13}C NMR (100 MHz, $CDCl_3$):** δ 165.4, 147.5, 140.5, 138.2, 130.6(q, $J^{C\text{-}F}$= 32.6 Hz), 127.9, 125.7(q, $J^{C\text{-}F}$= 3.4 Hz), 124.1(q, $J^{C\text{-}F}$= 272 Hz), 123.8(q, $J^{C\text{-}F}$= 282 Hz), 120.9, 111.7, 76.9(q, $J^{C\text{-}F}$= 32 Hz), 71.0, 53.8. **^{19}F NMR (376 MHz, $CDCl_3$):** δ -62.8 (s, 3F), -76.8 (d, $J^{F\text{-}H}$ = 6.5 Hz, 3F). **R_f** = 0.28 (10% EtOAc/hexane). **HRMS**(APCI, +MS) calcd. for [M+H] $C_{16}H_{14}F_6NO_2$ m/z: 366.0929, found: 366.0952. **HPLC**: Daicel CHIRALPAK OJ-H column, 2% IPA/Hexane, flow 0.75ml/min; (4R,5S)-ligand: 9.9 min (major), 10.9 min (minor).

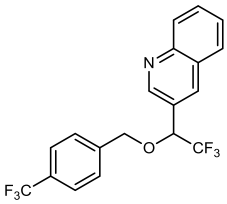

1-(quinolin-3-yl)-1-(4-trifluoromethyl)benzyloxy-2,2,2-trifluoroethane(**6p**).

Used 324 mg of trimethoxy(quinolin-3-yl) silane. Product isolated as slightly yellowish oil.

Run 1: 290 mg, 75% yield (89% by ^{19}F NMR), 49% *ee*. **Run 2:** 283 mg, 73% yield (86% by ^{19}F NMR), 55% *ee*.

^{1}H NMR (500 MHz, CDCl$_3$): δ 8.96(s, 1H), 8.27(s, 1H), 8.17(d, J^{H-H}= 8.47 Hz, 1H), 7.88(d, J^{H-H}= 8.3 Hz, 1H), 7.81(t, 7.1 Hz, 1H), 7.6-7.66 (m, 3H), 7.45(d, J^{H-H}= 7.9 Hz, 2H), 4.91(q, J^{H-F} = 6.5 Hz, 1H), 4.80 (d, J^{H-H}= 12.1 Hz, 1H), 4.67(d, J^{H-H}= 12.1 Hz, 1H). **^{13}C NMR (100 MHz, CDCl$_3$):** δ 149.7, 148.9, 140.2, 136.4, 130.8, 130.7(q, J^{C-F}= 32.6 Hz), 129.6, 128.2, 128.0, 127.6, 127.5, 125.8(q, J^{C-F}= 3.8 Hz), 125.4, 124.1(q, J^{C-F}= 273 Hz), 123.7(q, J^{C-F}= 282 Hz), 77.5(q, J^{C-F}= 32 Hz), 71.6. **^{19}F NMR (376 MHz, CDCl$_3$):** δ -62.8 (s, 3F), -76.3 (d, J^{F-H}= 6.4 Hz, 3F). **R$_f$** = 0.28 (20% EtOAc/hexane). **HRMS**(APCI, +MS) calcd. for [M+H] C19H14F6NO m/z: 386.0980, found: 386.0997. **HPLC**: Daicel CHIRALPAK OD-H column, 7% IPA/Hexane, flow 0.75ml/min; (4R,5S)-ligand: 9.8 min (major), 13.8 min (minor).

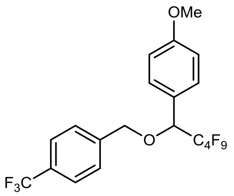

1-(4-methoxyphenyl)-1-(4-trifluoromethyl)benzyloxy-1-*H*-perfluoropentane(**6b'**).

Used 442.6 mg of **4g** and 297 mg (268 μl) trimethoxy(4-methoxyphenyl) silane. Product isolated as colorless oil.

Run 1: 437 mg, 85% yield (94% by ^{19}F NMR), 96% *ee*. **Run 2:** 416 mg, 82% yield (94% by ^{19}F NMR), 93% *ee*.

^{1}H NMR (300 MHz, CDCl$_3$): 7.61(d, J^{H-H}= 8.1 Hz, 2H), 7.34-7.42(m, 4H), 6.96(d, J^{H-H}= 8.7 Hz, 2H), 4.82, (dd, J^{H-F}= 19.2 Hz, 5.2 Hz, 1H), 4.59(d, J^{H-H}= 12.2 Hz, 1H), 4.43(d, J^{H-H}= 12.2 Hz, 1H), 3.86(s, 3H). **^{13}C NMR (151 MHz, CDCl$_3$):** δ 161, 140.6, 130.5, 130.4, (q, J^{C-F}= 32.3 Hz), 127.9, 125.6(q, J^{C-F}= 3.8 Hz), 124.2 (q, J^{C-F}= 271 Hz), 114.23, 78.2(dd, J^{C-F}= 30.9 Hz, 21.3 Hz), 70.3, 55.5. *Signals for carbons of the perfluoroalkyl group were not observed.* **^{19}F NMR (188 MHz, CDCl$_3$):** δ -62.8(s, 3F), -81.1(tt, J^{F-F}= 10.1 Hz, 3Hz, 3F), -116.3(dm, J^{F-F}= 291.1 Hz, 1F(α-F)), -121.3(dm, J^{F-F}= 295.6 Hz, 1F(γ-F)), -123.5(dm, J^{F-F}= 295.6 Hz, 1F(γ-F)), -125.3(dm, J^{F-F}= 289 Hz, 1F(β-F)), -125.7(dm, J^{F-F}= 291.1 Hz, 1F(α-F)), -125.7(dm, J^{F-F}= 289 Hz, 1F(β-F)). **R$_f$** = 0.22 (10% DCM/Hexane). **HRMS**(APCI, +MS) calcd. for $C_{20}H_{15}F_{12}O_2$ [M+] m/z: 514.0797, found: 514.0802. **HPLC**: Daicel CHIRALPAK OD-H column, 1% IPA/Hexane, flow 0.75ml/min; (4R,5S)-ligand: 6.4 min (major), 10.9 min (minor).

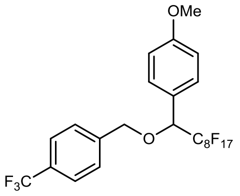

1-(4-methoxyphenyl)-1-(4-trifluoromethyl)benzyloxy-1-*H*-perfluorononane(**6b''**).

Used 642.6 mg of **4f** and 297 mg (268 μl) trimethoxy(4-methoxyphenyl) silane. Product isolated as colorless solid.

Run 1: 583 mg, 83% yield (94% by ^{19}F NMR), 95% *ee*. **Run 2:** 598 mg, 84% yield (94% by ^{19}F NMR), 95% *ee*.

^{1}H NMR (300 MHz, CDCl$_3$): 7.61(d, J^{H-H}= 8 Hz, 2H), 7.38(m, 4H), 7.33(d, J^{H-H}= 8.5 Hz, 2H), 6.97(d, J^{H-H}= 8.8 Hz, 2H), 4.82(dd, J^{H-F}= 19.1 Hz, 4.6 Hz, 1H), 4.60 (d, J^{H-H}= 12.1 Hz, 1H), 4.43(q, J^{H-H}= 12.1 Hz, 1H), 3.85(s, 3H). **^{13}C NMR (151 MHz, CDCl$_3$):** δ 161, 140.6(q, J^{C-F}= 1.5 Hz), 130.5, 130.4, (q, J^{C-F}= 32.3 Hz), 127.9, 125.6(q, J^{C-F}= 3.7 Hz), 124.2 (q, J^{C-F}= 272 Hz), 123, 114.3, 78.3(dd,

J^{C-F}= 30.6 Hz, 20.6 Hz), 70.3, 55.5. *Signals for carbons of the perfluoroalkyl group were not observed.* **^{19}F NMR (188 MHz, $CDCl_3$):** δ -62.8(s, 3F), -80.9 (t, J^{F-F} = 10.2 Hz 3F), -116.1(dm, 288 Hz), -119 - -128(m, 13F). **R_f** = 0.23 (10% DCM/Hexane). **HRMS**(APCI, +MS) calcd. for $C_{24}H_{15}F_{20}O_2$ [M+] m/z: 715.0753, found: 715.0742. **HPLC**: Daicel CHIRALPAK OD-H column, 100% Hexane, flow 1.5ml/min; (4R,5S)-ligand: 8.1 min (major), 20.8 min (minor).

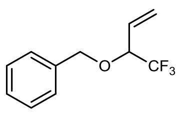

1-vinyl-1-benzyloxy-2,2,2-trifluoroethane(**8a**).

Ligand **7g** (36.5 mg) was used instead of **7a**. Used 185 mg (192 μL) of trimethoxy(vinyl) silane. No additional light irradiation was used. Product isolated as colorless oil.

Run 1: 198 mg, 92% yield (98% by ^{19}F NMR),91% *ee*. **Run 2:** 201 mg, 93% yield (98% by ^{19}F NMR), 91% *ee*.

^{1}H NMR (300 MHz, $CDCl_3$): δ 7.28-7.43(m, 5H), 5.74-5.90(m, 1H), 5.55(s, 1H), 5.51 (d, J^{H-H} = 5.4 Hz, 1H), 4.73(d, J^{H-H} = 12 Hz, 1H), 4.60(d, J^{H-H} = 12 Hz, 1H), 4.13(m, 1H). **^{13}C NMR (151 MHz, $CDCl_3$):** δ 136.8, 129.3(q, J^{C-F}= 1.6 Hz), 128.7, 128.3, 128.1, 124.1(q, J^{C-F}= 282 Hz), 123.2, 77.7(q, J^{C-F}= 31.4 Hz), 71.8. **^{19}F NMR (188 MHz, $CDCl_3$):** -77.0 (d, J^{F-H}= 6.7 Hz 3F). **R_f** = 0.42 (10% DCM/Hexane). **HRMS**(APCI, -MS) calcd. for [M-H] $C_{11}H_{10}F_3O$ m/z: 215.0684, found: 215.0696. **HPLC**: Daicel CHIRALPAK OJ-H column, 3% IPA/Hexane, flow 0.75ml/min; (4R)-ligand: 6.4 min (minor), 6.9 min (major).

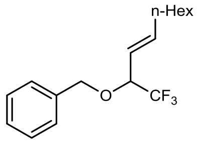

1-(*E*-octenyl-1)-1-benzyloxy-2,2,2-trifluoroethane(**8b**).

Ligand **7g** (36.5 mg) was used instead of **7a**. Used 291 mg of trimethoxy(octenyl) silane. No additional light irradiation was used. Product isolated as colorless oil.

Run 1: 280 mg, 93% yield (98% by ^{19}F NMR), 89% *ee*. **Run 2:** 283 mg, 93% yield (99% by ^{19}F NMR), 91% *ee*.

^{1}H NMR (300 MHz, $CDCl_3$): 7.28-7.42(m, 5H), 5.88(dt, J^{H-H}= 15.5 Hz, 5.8 Hz, 1H), 5.42(dd, J^{H-H}= 15.5 Hz, J^{H-H}= 8.1 Hz, 1H), 4.70(d, J^{H-H}= 12.1 Hz, 1H), 4.54(d, J^{H-H}= 12.1 Hz, 1H), 4.06(m, 1H).**^{13}C NMR (151 MHz, $CDCl_3$):** δ 141, 137.1, 128.6, 128.2, 128, 124.3(q, J^{C-F}= 281 Hz), 120.9(q, J^{C-F}= 1.6 Hz), 77.7(q, J^{C-F}= 31.4 Hz), 71, 32.4, 31.8, 28.8, 28.7, 22.7, 14.2. **^{19}F NMR (188 MHz, $CDCl_3$):** δ -77.1 (d, J^{F-H} = 6.3 Hz 3F). **R_f** = 0.48 (10% DCM/Hexane). **HRMS**(APCI, -MS) calcd. for [M-H] $C_{17}H_{22}F_3O$ m/z: 299.1623, found: 299.1627. **HPLC**: Daicel CHIRALPAK OJ-H column, 1% IPA/Hexane, flow 0.75ml/min; (4R)-ligand: 5.3 min (minor), 5.7 min (major).

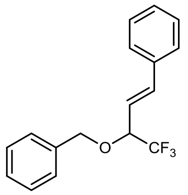

1-(2-phenylethenyl-1)-1-benzyloxy-2,2,2-trifluoroethane(**8c**).

Ligand **7g** (36.5 mg) was used instead of **7a**. Used 291 mg of trimethoxy(styryl) silane. No additional light irradiation was used. Product isolated as colorless oil.

Run 1: 275 mg, 95% yield (100% by ^{19}F NMR), 88% *ee*. **Run 2:** 272 mg, 94% yield (100% by ^{19}F NMR), 92% *ee*.

^{1}H NMR (300 MHz, $CDCl_3$): 7.28-7.50(m, 10H), 6.77(d, J^{H-H}= 16 Hz, 1H), 6.14(dd, J^{H-H}= 16 Hz, J^{H-H}= 7.8 Hz, 1H), 4.78(d, J^{H-H}= 12 Hz, 1H), 4.64(d, J^{H-H}= 12 Hz, 1H), 4.3(m, 1H), 4.12(q, J^{H-H}= 6.7 Hz, 2H), 1.2-1.48(m, 8H), 0.89(t, J^{H-H}=6.8 Hz, 3H). **^{13}C NMR (151 MHz, $CDCl_3$):** δ 138, 136.8, 135.5, 128.95, 128.9, 128.7, 128.3, 128.1, 127.1, 124.2(q, J^{C-F}= 282 Hz), 119.9, 77.6(q, J^{C-F}= 31.4 Hz), 71.6. **^{19}F NMR (188 MHz, $CDCl_3$):** δ -76.7 (d, J^{F-H} = 6.8 Hz 3F). **R_f** = 0.29 (10% DCM/Hexane). **HRMS**(APCI, -MS) calcd for [M-H] $C_{17}H_{14}F_3O$ m/z: 291.1002, found: 291.1000. **HPLC**: Daicel CHIRALPAK OJ-H column, 3% IPA/Hexane, flow 0.75ml/min; (4R)-ligand: 12.3 min (major), 13.6 min (minor).

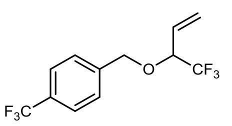

1-vinyl-1-(4-trifluoromethyl)benzyloxy-2,2,2-trifluoroethane(**8d**).

Ligand **7g** (36.5 mg) was used instead of **7a**. Used 185 mg (192 μL) of trimethoxy(vinyl) silane. No additional light irradiation was used. Product isolated as colorless oil.

Run 1: 265 mg, 95% yield (98% by ^{19}F NMR). **Run 2:** 262 mg, 94% yield (99% by ^{19}F NMR).

^{1}H NMR (300 MHz, $CDCl_3$): δ 7.63(d, 2H, J^{H-H}= 8.1 Hz, 7.47(d, J^{H-H}= 8.1 Hz, 2H), 5.75-5.9, (m, 1H), 5.55-5.6(m, 2H), 4.77(d, J^{H-H}= 12.6 Hz, 1H), 4.65 (d, J^{H-H} = 12.6 Hz, 1H), 4.14(m, 1H). **^{13}C NMR (151 MHz, $CDCl_3$):** δ 140.9, 130.4(q, J^{C-F}= 32 Hz), 129.0(q, J^{C-F}= 1.6 Hz), 127.8, 125.7(q, J^{C-F}= 3.8 Hz), 124.2(q, J^{C-F}= 272 Hz), 124.2(q, J^{C-F}= 282 Hz), 123.6, 78.4(q, J^{C-F}= 31.4 Hz), 70.9. **^{19}F NMR (188 MHz, $CDCl_3$):** δ -62.8 (s, 3F), -77.0 (d, J^{F-H} = 6.4 Hz 3F). **R_f** = 0.46 (10% DCM/Hexane). **HRMS**(APCI, +MS) calcd. for $C_{12}H_{11}F_6O$ [M+H] m/z: 283.0552, found: 283.0568. **HPLC**: Enantiomers were not separated.

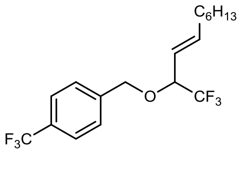

1-(*E*-octenyl-1)-1-(4-trifluoromethyl)benzyloxy-2,2,2-trifluoroethane(**8e**).

Ligand **7g** (36.5 mg) was used instead of **7a**. Used 185 mg (192 μL) of trimethoxy(octenyl) silane. No additional light irradiation was used. Product isolated as colorless oil.

Run 1: 348 mg, 95% yield (100% by ^{19}F NMR). **Run 2:** 340 mg, 93% yield (100% by ^{19}F NMR)

^{1}H NMR (300 MHz, $CDCl_3$): δ 7.62(d, 2H, J^{H-H}= 8.1 Hz, 7.45(d, J^{H-H}= 8.1 Hz, 2H), 5.91(dt, J^{H-H}= 15.5 Hz, J^{H-H}= 6.8 Hz)5.43(dd, J^{H-H}= 15.5 Hz ,J^{H-H}= 8.2 Hz), 4.59(d, J^{H-H}= 12.6 Hz, 1H), 4.07(m, 1H), 2.13(m, 2H), 1.47-1.22(m, 8H), 0.93-0.83(m, 3H). **^{13}C NMR (151 MHz, $CDCl_3$):** δ 141.5, 141.3,

130.3(q, J^{C-F}= 32 Hz), 127.8, 125.6(q, J^{C-F}= 3.8 Hz), 124.2(q, J^{C-F}= 272 Hz), 124.1(q, J^{C-F}= 282 Hz), 120.7(q, J^{C-F}= 1.7 Hz), 78.4(q, J^{C-F}= 31.5 Hz), 70.2, 32.4, 31.7, 28.8, 28.7, 22.7, 14.1. **^{19}F NMR (188 MHz, $CDCl_3$):** δ -62.8 (s, 3F), -77.1 (d, J^{F-H} = 6.5 Hz 3F). **R_f** = 0.52 (10% DCM/Hexane). **HRMS**(APCI, +MS) calcd. for $C_{18}H_{23}F_6O$ [M+H] m/z: 369.1653, found: 368.1660. **HPLC**: Enantiomers were not separated.

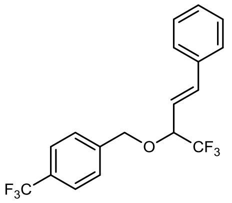

1-(2-phenylethenyl-1)-1-(4-trifluoromethyl)benzyloxy-2,2,2-trifluoroethane(**8f**).

Ligand **7g** (36.5 mg) was used instead of **7a**. Used 291 mg of trimethoxy(styryl) silane. No additional light irradiation was used. Product isolated as colorless oil.

Run 1: 341 mg, 95% yield (100% by ^{19}F NMR), 88% *ee*. **Run 2:** 344 mg, 96% yield (100% by ^{19}F NMR), 93% *ee*.

^{1}H NMR (300 MHz, $CDCl_3$): 7.62(d, J^{H-H}= 8.2 Hz, 2H), 7.52-7.32(m, 7H), 6.78(d, J^{H-H}= 16 Hz, 1H), 6.14(dd, J^{H-H}= 16 Hz, J^{H-H}= 6.9 Hz, 1H), 4.82(d, J^{H-H}= 12.5 Hz, 1H), 4.68(d, J^{H-H}= 12.5 Hz, 1H), 4.31(m, 1H). **^{13}C NMR (151 MHz, $CDCl_3$):** δ 141, 138.4, 135.3, 130.4(q, J^{C-F}= 32 Hz), 129.1, 128.9, 127.9, 127.1, 125.6(q, J^{C-F}= 3.8 Hz), 124.2(q, J^{C-F}= 272 Hz), 124.0(q, J^{C-F}= 282 Hz), 119.5, 78.4(q, J^{C-F}= 31.4 Hz), 70.7. **^{19}F NMR (188 MHz, $CDCl_3$):** δ -62.8 (s, 3F), -76.7 (d, J^{F-H} = 6.3 Hz 3F). **R_f** = 0.32 (10% DCM/Hexane). **HRMS**(APCI, +MS) calcd. for $C_{18}H_{15}F_6O$ [M+H] m/z: 361.1015, found: 361.1021. **HPLC**: Daicel CHIRALPAK OJ-H column, 3% IPA/Hexane, flow 0.75ml/min; (4R)-ligand: 15.4 min (major), 19.0 min (minor).

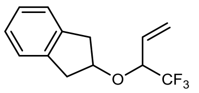

1-vinyl-1-(2-indanoxy)-2,2,2-trifluoroethane(**8g**).

Ligand **7g** (36.5 mg) was used instead of **7a**. Used 185 mg (192 µL) of trimethoxy(vinyl) silane and 251 mg of **4g**. No additional light irradiation was used. Product isolated as colorless oil.

Run 1: 229 mg, 95% yield (98% by ^{19}F NMR), 88% *ee*. **Run 2:** 231 mg, 95% yield (97% by ^{19}F NMR), 89% *ee*.

^{1}H NMR (600 MHz, $CDCl_3$): δ 7.12-7.24(m, 4H), 5.84(ddd, J^{H-H} = 16.9 Hz, 10.3 Hz, 6.4 Hz, 1H), 5.52(d, J^{H-H} = 16.9 Hz, 1H), 5.47(d, J^{H-H}= 10.3 Hz, 1H), 4.55(tt, J^{H-H} = 6.4 Hz, 4.9 Hz, 1H), 4.21(m, 1H), 3.13-3.25(m, 2H), 2.98-3.08(m, 2H). **^{13}C NMR (151 MHz, $CDCl_3$):** δ 140.44, 140.40, 129.93, 129.91, 126.85, 126.84, 124.8, 124(q, J^{C-F}= 280 Hz), 122.1, 80.7, 77.7(q, J^{C-F}= 31 Hz), 39.7, 38.3. **^{19}F NMR (188 MHz, $CDCl_3$):** δ –77.4 (d, J^{F-H} = 6.7 Hz 3F). **R_f** = 0.16 (Hexane). **HRMS**(ES MS+) calcd. for [M+H] $C_{13}H_{13}OF_3$, m/z: 242.0918, found: 242.0953. **HPLC**: Daicel CHIRALPAK OJ-H column, 1% IPA/Hexane, flow 0.75ml/min; (4R)-ligand: 8.1 min (major), 9.7 min (minor).

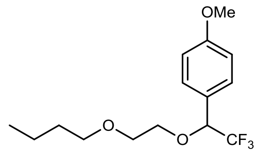

1-(2-(butoxy)ethoxy)-1-(4-methoxyphenyl) -2,2,2-trifluoroethane(**11a**).

Used 234.6mg of **4c** and 297 mg (268 µl) of trimethoxy(4-methoxyphenyl) silane. Product isolated as colorless oil. Note: reaction time 72h.

Run 1: 281 mg, 92% yield (98% by ^{19}F NMR), 93% *ee*. **Run 2:** 282 mg, 92% yield (97% by ^{19}F NMR), 94% *ee*.

^{1}H NMR (300 MHz, CDCl$_3$): 7.33(d, $J^{H\text{-}H}$= 8.5 Hz, 2H), 6.92(d, $J^{H\text{-}H}$= 8.5 Hz, 2H), 4.71(q, $J^{H\text{-}F}$= 6.8 Hz, 1H), 3.82(s, 3H), 3.62- 3.68(m, 1H), 3.55-3.62(m, 1H), 3.42(m, 2H), 1.47-1.60(m, 2H), 1.28-1.43(m, 2H), 0.91(t, $J^{H\text{-}H}$= 7.2 Hz, 3H). **^{13}C NMR (151 MHz, CDCl$_3$):** δ 156.6, 129.7, 125, 124.1(q, $J^{C\text{-}F}$= 281Hz), 114, 79.7(q, $J^{C\text{-}F}$= 30.8Hz), 71.4, 70.4, 69.6, 55.4, 31.9, 19.4, 14. **^{19}F NMR (188 MHz, CDCl$_3$):** δ -77.1 (d, $J^{F\text{-}H}$ = 6.7 Hz 3F). **R$_f$** = 0.29 (40% DCM/Hexane). **HRMS**(ESI, +MS) calcd. for $C_{15}H_{21}F_3O_3Na$ [M+Na] m/z: 329.1335, found: 329.1387. **HPLC**: Daicel CHIRALPAK OD-H column, 0.5% IPA/Hexane, flow 0.75ml/min; (4R,5S)-ligand: 17.4 min (major), 20.0 min (minor).

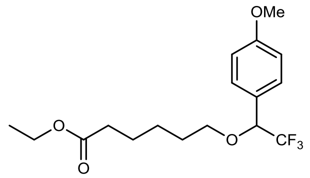

Ethyl 6-(1-(4-methoxyphenyl)-2,2,2-trifluoroethoxy)hexanoate(**11b**).

Used 277.7mg of **4d** and 297 mg (268 µl) of trimethoxy(4-methoxyphenyl) silane. Product isolated as colorless oil. Note: reaction time 72h.

Run 1: 324 mg, 93% yield (97% by ^{19}F NMR), 97% *ee*. **Run 2:** 319 mg, 92% yield (97% by ^{19}F NMR), 96% *ee*.

^{1}H NMR (300 MHz, CDCl$_3$): 7.33(d, $J^{H\text{-}H}$= 8.5 Hz, 2H), 6.92(d, $J^{H\text{-}H}$= 8.5 Hz, 2H), 4.51(q, $J^{H\text{-}F}$= 6.7 Hz, 1H), 4.12(q, $J^{H\text{-}H}$= 7.1 Hz, 2H) 3.82(s, 3H), 3.47(t, $J^{H\text{-}H}$= 6.4 Hz, 2H), 2.28(t, $J^{H\text{-}H}$= 7.5 Hz, 2H), 1.55-1.69(m, 4H), 1.32-1.46(m, 2H), 1.25(t, $J^{H\text{-}H}$= 7.1 Hz). **^{13}C NMR (151 MHz, CDCl$_3$):** δ 173.8, 160.6, 129.5, 125.2, 124.1(q, $J^{C\text{-}F}$= 283 Hz), 114.1, 79.6(q, $J^{C\text{-}F}$= 31 Hz), 70.3, 60.4, 55.4, 34.4, 29.3, 25.6, 24.8, 14.4. **^{19}F NMR (188 MHz, CDCl$_3$):** δ -77.1 (d, $J^{F\text{-}H}$ = 6.7 Hz 3F). **R$_f$** = 0.17 (40% DCM/Hexane). **HRMS**(APCI, +MS) calcd. for [M+H] $C_{17}H_{24}F_3O_4$ m/z: 349.1621, found: 349.1623. **HPLC**: Daicel CHIRALPAK OJ-H column, 1% IPA/Hexane, flow 0.75ml/min; (4R,5S)-ligand: 6.1 min (major), 6.5 min (minor).

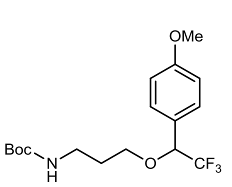

1-chloro-1-(3-(N-Bocamino)propoxy)-2,2,2-trifluoroethane (**11c**).

Used 291.7 mg of **4e** and 297 mg (268 µl) of trimethoxy(4-methoxyphenyl) silane. Product isolated as colorless oil. Note: reaction time 5 days.

Run 1: 277 mg, 77% yield (80% by ^{19}F NMR), 95% *ee*. **Run 2:** 280 mg, 78% yield (82% by ^{19}F NMR), 94% *ee*.

^{1}H NMR (300 MHz, CDCl$_3$): 7.33(d, $J^{H\text{-}H}$= 8.5 Hz, 2H), 6.92(d, $J^{H\text{-}H}$= 8. , 2H), 4.85, (bs, 1H), 4.53(q, $J^{H\text{-}F}$= 6.7 Hz, 1H), 3.82(s, 1H), (m, 1H), 3.54(t, $J^{H\text{-}H}$= 5.8 Hz, 2H), 3.15-3.32(m, 2H), 1.69-

1.87(m, 2H), 1.43(s, 9H). **^{13}C NMR (151 MHz, CDCl$_3$):** δ 160.7, 126.1, 129.6, 124.8, 124.(q, $J^{C\text{-}F}$= 282 Hz), 114.2, 79.8(q, $J^{C\text{-}F}$= 31.3 Hz), 68.7, 55.4, 38.4, 29.8, 28.5. **^{19}F NMR (188 MHz, CDCl$_3$):** δ -77.1 (d, $J^{F\text{-}H}$ = 6.7 Hz 3F). **R$_f$** = 0.15 (10% EtOAc/Hexane). **HRMS**(ESI, +MS) calcd. for $C_{17}H_{24}F_3NO_4Na$ [M+Na] m/z: 386.1550, found: 386.1550. **HPLC**: Daicel CHIRALPAK OJ-H column, 6% IPA/Hexane, flow 0.75ml/min; (4R,5S)-ligand: 20.1 min (major), 26.3 min (minor).

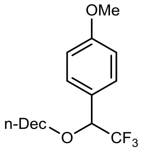

<u>1-chloro-1-decyloxy-2,2,2-trifluoroethane</u>(**11d**).

Used 288.7mg of **4f** and 297 mg (268 μl) of trimethoxy(4-methoxyphenyl) silane. Product isolated as colorless oil. Note: reaction time 72h.

Run 1: 327 mg, 91% yield (97% by ^{19}F NMR), 94% *ee*. **Run 2:** 324 mg, 91% yield (97% by ^{19}F NMR), 95% *ee*.

^{1}H NMR (300 MHz, CDCl$_3$): 7.34(d, $J^{H\text{-}H}$= 8.5 Hz, 2H), 6.92(d, $J^{H\text{-}H}$= 8.5 Hz, 2H), 4.52(q, $J^{H\text{-}F}$= 6.7 Hz, 1H), 3.82(s, 3H), 3.47(t, $J^{H\text{-}H}$= 6.6 Hz, 2H), 1.56-1.66(m, 2H), 1.16-1.98(m, 14H), 0.88(t, $J^{H\text{-}H}$= 6.8 Hz, 3H). **^{13}C NMR (151 MHz, CDCl$_3$):** δ 160.5, 129.6, 125.3, 124.1(q, $J^{C\text{-}F}$= 283 Hz), 114.1, 79.6(q, $J^{C\text{-}F}$= 31 Hz), 70.7, 55.4, 32.0, 29.7, 29.6, 29.5, 26.0, 22.8, 14.2. **^{19}F NMR (188 MHz, CDCl$_3$):** δ -77.1 (d, $J^{F\text{-}H}$ = 6.7 Hz 3F). **R$_f$** = 0.30 (10% DCM/Hexane). **HRMS**(APCI, +MS) calcd. for $C_{19}H_{30}F_3O_2$ [M+H] m/z: 347.2198, found: 347.2200. **HPLC**: Daicel CHIRALPAK AD-H column, 1% IPA/Hexane, flow 0.75ml/min; (4R,5S)-ligand: 6.1 min (major), 6.5 min (minor).

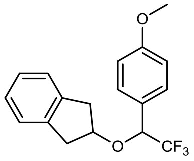

<u>1-(4-methoxyphenyl)-1-(2-indanoxy)-2,2,2-trifluoroethane</u>(**11e**).

Used 297 mg of trimethoxy(4-methoxyphenyl) silane and 251 mg of **4g**. Product isolated as colorless oil. Note: reaction time 72h.

Run 1: 304 mg, 94% yield (99% by ^{19}F NMR), 99% *ee*. **Run 2:** 302 mg, 94% yield (98% by ^{19}F NMR), 98% *ee*.

^{1}H NMR (500 MHz, CDCl$_3$): δ 7.37(d, $J^{H\text{-}H}$ = 8.7 Hz, 2H), 7.20(m, 1H), 7.10-7.18(m, 3H), 6.93(d, $J^{H\text{-}H}$ = 8.7 Hz, 2H), 4.69(q, $J^{H\text{-}F}$ = 6.7 Hz, 1H), 4.43(m, 1H), 3.83(s, 3H), 3.18 (dd, $J^{H\text{-}H}$= 16 Hz, 6.5 Hz, 1H), 3.02-3.12(m, 2H), 2.95(dd, $J^{H\text{-}H}$= 16.2 Hz, 5.2 Hz, 1H). **^{13}C NMR (125 MHz, CDCl$_3$):** δ 160.6, 140.5, 140.3, 129.7, 126.81, 126.78, 126.3, 124.8, 124.7, 124(q, $J^{C\text{-}F}$= 280 Hz), 114.1, 80.2, 78.2(q, $J^{C\text{-}F}$= 32 Hz), 55.4, 39.8, 38.9. **^{19}F NMR (188 MHz, CDCl$_3$):** δ –77.1 (d, $J^{F\text{-}H}$ = 6.7 Hz, 3F). **R$_f$** = 0.24 (20%DCM/hexane). **HRMS**(ES MS+) calcd. for [M+H] $C_{18}H_{18}O_2F_3$, m/z: 323.1259, found: 323.1226. **HPLC**: Daicel CHIRALPAK OJ-H column, 7% IPA/Hexane, flow 0.75ml/min; (4R,5S)-ligand: 14.0 min (minor), 19.2 min (major).

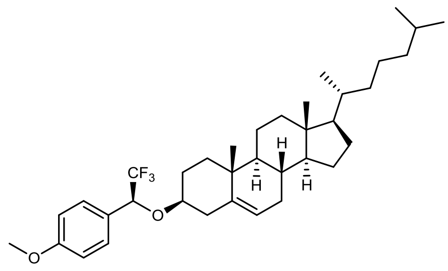

1-(4-methoxyphenyl)-1-(2-cholesteryloxy)-2,2,2-trifluoroethane(**11f**).

Used 297 mg of trimethoxy(4-methoxyphenyl) silane and 503 mg of **4h**. Product isolated as white solid. Note: reaction time 72h.

Run 1: 498 mg, 87% yield (94% by ^{19}F NMR), 95% *dr*. **Run 2:** 506 mg, 88% yield (93% by ^{19}F NMR), 96% *dr*.

(*S*)-ligand: ^{1}H NMR (500 MHz, CDCl$_3$): δ 7.37(d, J^{H-H}= 8.7 Hz, 2H),6.91(d, J^{H-H}= 8.7 Hz, 2H), 5.24(m, 1H), 4.69(q, J^{H-F}= 6.9 Hz, 1H), 3.82(s, 3H), 3.26(m, 1H), 2.30(m, 1H), 2.16(m, 1H), 1.89-2.02(m, 3H), 1.76-1.89(m, 2H), 1.19-1.61(m, 14H), 1-1.18(m, 6H), 0.99(s, 3H), 0.92-0.99(m, 2H), 0.9(d, J^{H-H}= 6.5 Hz, 3H), 0.859(d, J^{H-H}= 6.6 Hz, 3H), 0.855(d, J^{H-H}= 6.6 Hz, 3H), 0.659(s, 3H). **^{13}C NMR (125 MHz, CDCl$_3$):** δ 160.3, 140.5, 129.5, 126, 124.2(q, J^{C-F}= 282 Hz), 122.1, 114, 78.9, 76.6(q, J^{C-F}= 30.5 Hz),56.9, 56.3, 55.4, 50.2, 42.4, 39.9, 39.8, 39.7, 37.1, 36.9, 36.3, 35.9, 32.00, 31.95, 28.3, 28.2, 27.6, 24.4, 23.9, 23.0, 22.7, 21.2, 19.5, 18.6, 12.0. **^{19}F NMR (188 MHz, CDCl$_3$):** δ -77.3 (d, J^{F-H} = 6.8 Hz, 3F). **R$_f$** = 0.16 (hexane). X-ray suitable crystal was obtained by slow cooling of methanol solution: long white needles.

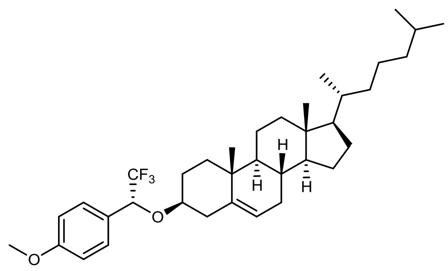

(*R*)-ligand: ^{1}H NMR (500 MHz, CDCl$_3$): δ 7.37(d, J^{H-H}= 8.7 Hz, 2H),6.91(d, J^{H-H}= 8.7 Hz, 2H), 5.35(m, 1H), 4.69(q, J^{H-F}= 6.9 Hz, 1H), 3.82(s, 3H), 3.26(m, 1H), 2.42(m, 1H), 2.31(m, 1H), 1.92-2.02(m, 2H), 1.73-1.86(m, 2H), 1.67-1.74(m, 1H), 1.20-1.6(m, 12H), 1.0-1.18(m, 6H), 0.99(S, 3H), 0.91-0.99(m, 2H), 0.898(d, J^{H-H}= 6.5 Hz, 3H), 0.861(d, J^{H-H}= 6.6 Hz, 3H), 0.856(d, J^{H-H}= 6.6 Hz, 3H), 0.66(s, 3H). **^{13}C NMR (125 MHz, CDCl$_3$):** δ 160.4, 140.4, 129.5, 126, 124.2(q, J^{C-F}= 28 2Hz), 122.3, 114, 78.9, 76.9(q, J^{C-F}= 31.1 Hz),56.9, 56.3, 55.4, 50.2, 44.4, 39.9, 39.7, 38.4, 37.2, 36.9, 36.3, 35.9, 32.05, 31.96, 29.4, 28.4, 28.2, 24.4, 23.9, 23.0, 22.7, 21.2, 19.5, 18.8, 12.0. **^{19}F NMR (188 MHz, CDCl$_3$):** δ -77.3 (d, J^{F-H} = 6.8 Hz, 3F). **R$_f$** = 0.16 (hexane). **HRMS** (APCI, +MS), calcd. for [M+H] $C_{36}H_{54}F_3O_2$ m/z: 575.4070, found: 575.4048. **HPLC**: Phenomenex Lux Cellulose-3 column, 0% IPA/Hexane, flow 0.4 ml/min; (4R,5S)-ligand: 58.3 min (minor), 58.5 min (major).

Deprotection of ethers and absolute configuration determination

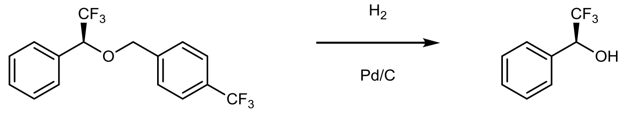

6a, 97%ee **9a**, 90%, 97%ee

Supplementary figure 1. Deprotection of benzylic ether **6a** by palladium-on-carbon-catalyzed hydrogenation.

The mixture 50 mg of **6a** (97% ee) and 10 mg of Pd/C in 1 ml of ethanol was stirred under 3 atm. of hydrogen for one day. After completion of the reaction, the reaction mixture was filtered through celite, washed with ethanol and subjected to preparative TLC (eluent DCM) to give **9a** 24 mg (90% yeid, 97% ee).

Spectroscopic data corresponds to previously reported data.[12] **HPLC**: Daicel CHIRALPAK OJ-H column, 13% IPA/Hexane, flow 0.75ml/min; (4S,5R)-ligand **7a**: 12.7 min (minor), 16.3 min (major). Comparison with commercial sample of (R)-**6a** (12.7 min major, 16.4 minor) gives (S)-configuration of **9a** and **6a**.

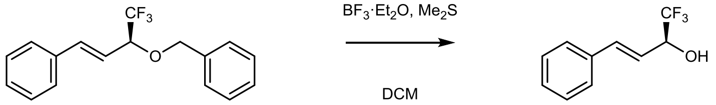

8c, 90% ee **9b**, 42%, 89% ee

Supplementary figure 2. Deprotection of **8c** by means of BF_3 etherate with dimethylsulphide to give allylic alcohol **9b.**

The deprotection was performed by literature procedure, starting from 50 mg of **8c** (90% ee).[13] After preparative TLC **9b** was obtained in 14 mg (42% yield, 89% ee).

Spectroscopic data corresponds to previously reported data.[12] **HPLC**: Daicel CHIRALPAK OJ-H column, 5% IPA/Hexane, flow 1 ml/min; (4R)-ligand **7g**: 16.4 min (major), 20.1 min (minor). Comparison with literature data gives (R)-configuration of **9b** and for **8c**.

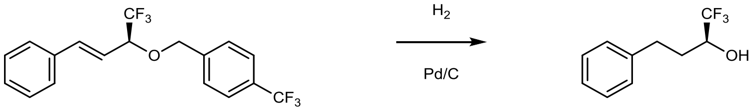

8f, 90% ee **9c**, 90%, 90% ee

Supplementary figure 3. One-pot reductive deprotection of **8f** with hydrogen and palladium on carbon to give alkyl alcohol **9c**.

Reaction was performed similar to the **6d,** using 50 mg of **8f** (90% ee). After preparative TLC **9c** was obtained in 25 mg (90% yield, 90% ee).

Spectroscopic data corresponds to previously reported data.[14] **HPLC**: Daicel Phenomex Lux Cellulose-3 column, 1% IPA/Hexane, flow 1 ml/min; (4R)-ligand **7g**: 37.55 min (major), 39.47 min (minor).

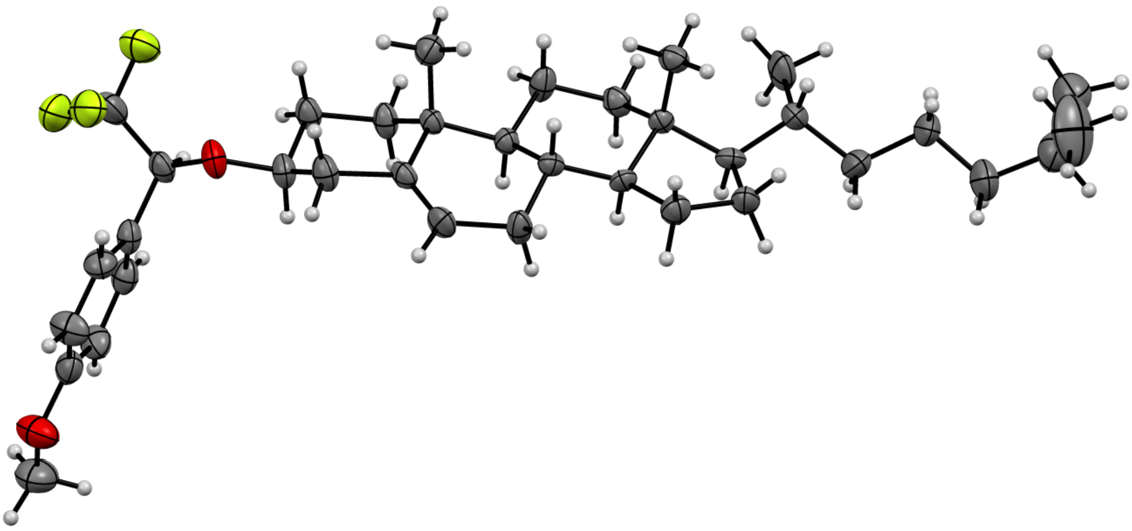

Supplementary figure 4. ORTEP drawing of **11f** with thermal ellipsoids at 50% probability level (CCDC 1854431). The second molecule of the unit cell is omitted for clarity. **11f** was obtained using (4S,5R)-**7a** as a ligand, giving (S)-configuration of newly formed asymmetric center.

Supplementary figures

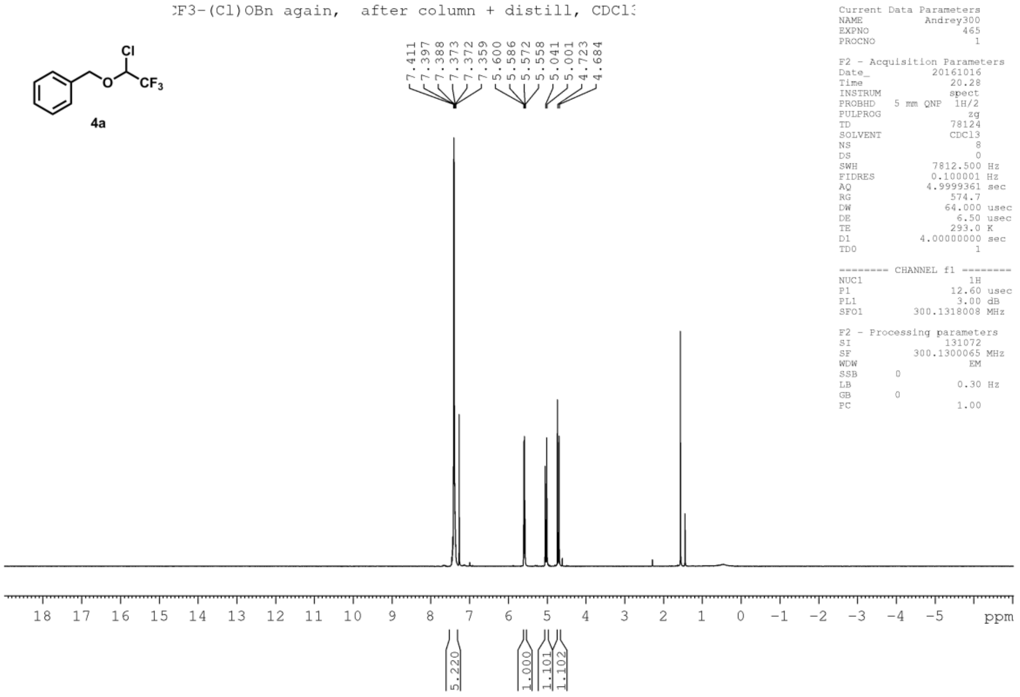

Supplementary figure 5. ^{1}H NMR spectra of **4a** in $CDCl_3$

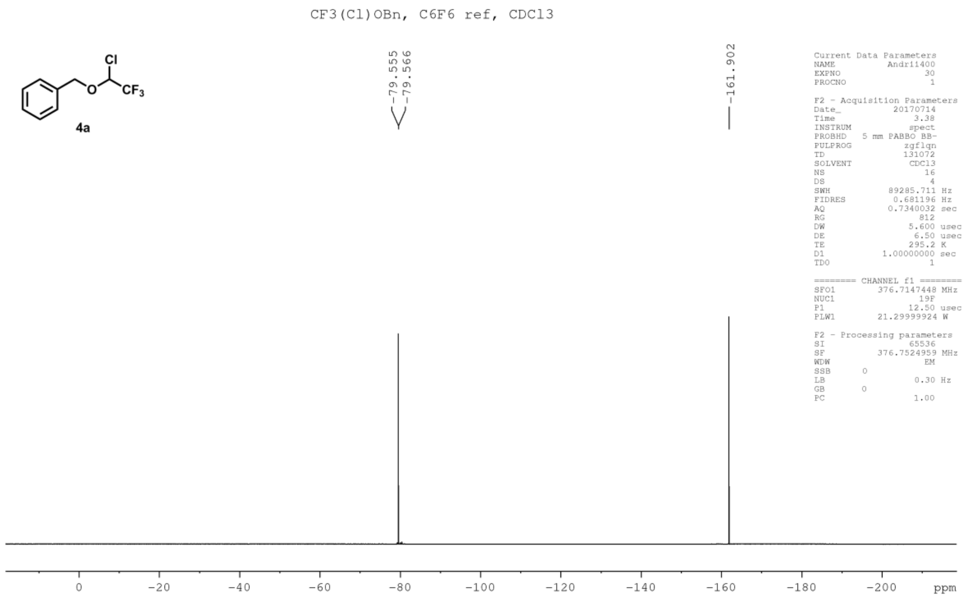

Supplementary figure 6. ^{19}F NMR spectra of **4a** in $CDCl_3$

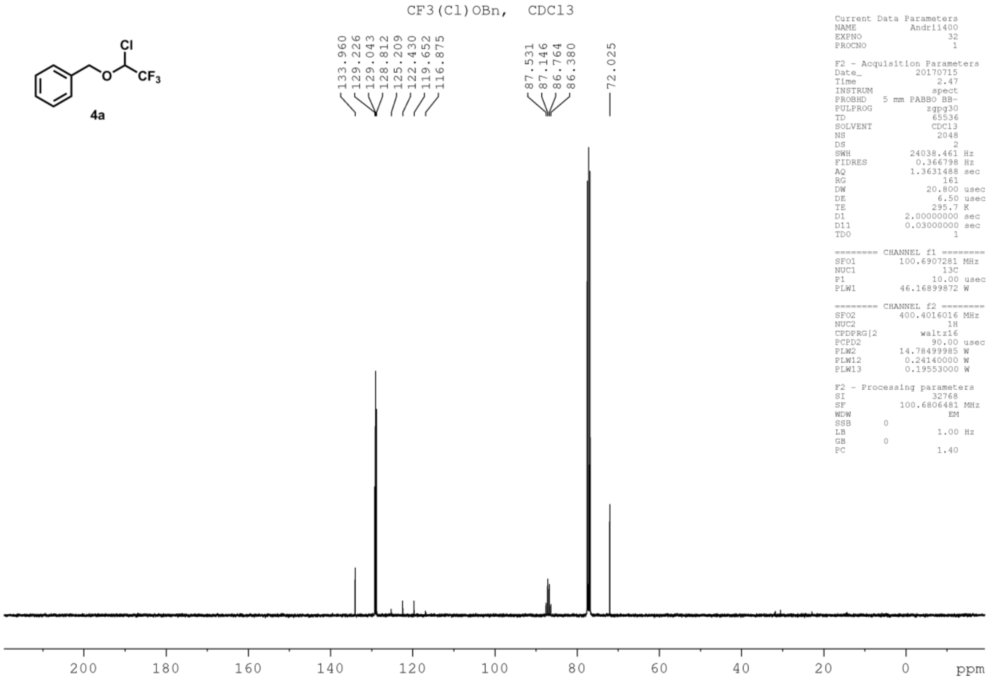

Supplementary figure 7. ^{13}C NMR spectra of **4a** in $CDCl_3$

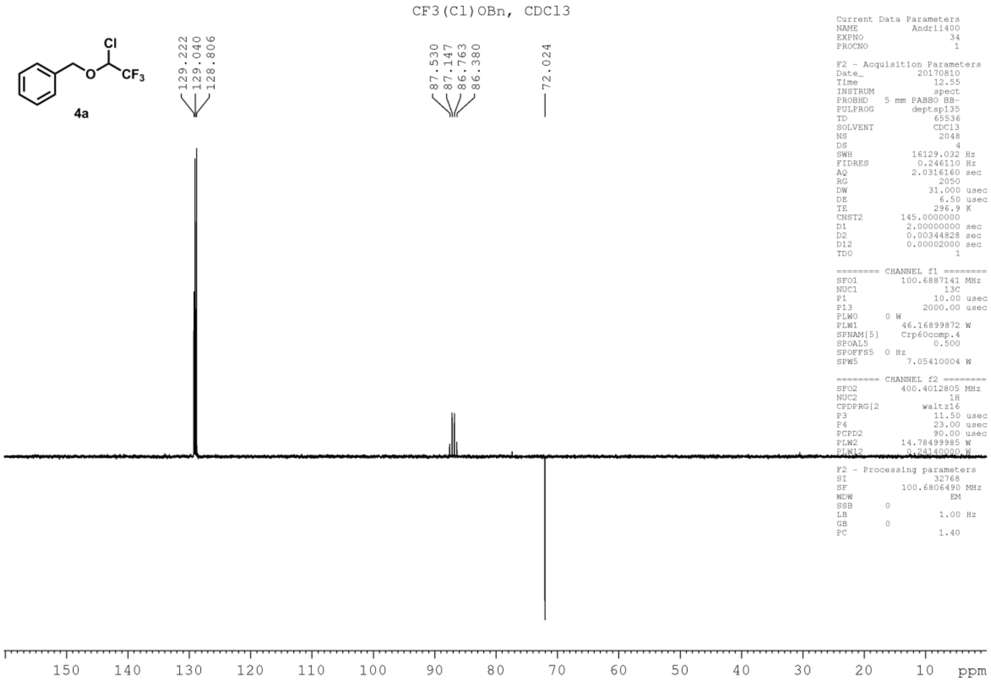

Supplementary figure 8. DEPT-135 NMR spectra of **4a** in $CDCl_3$

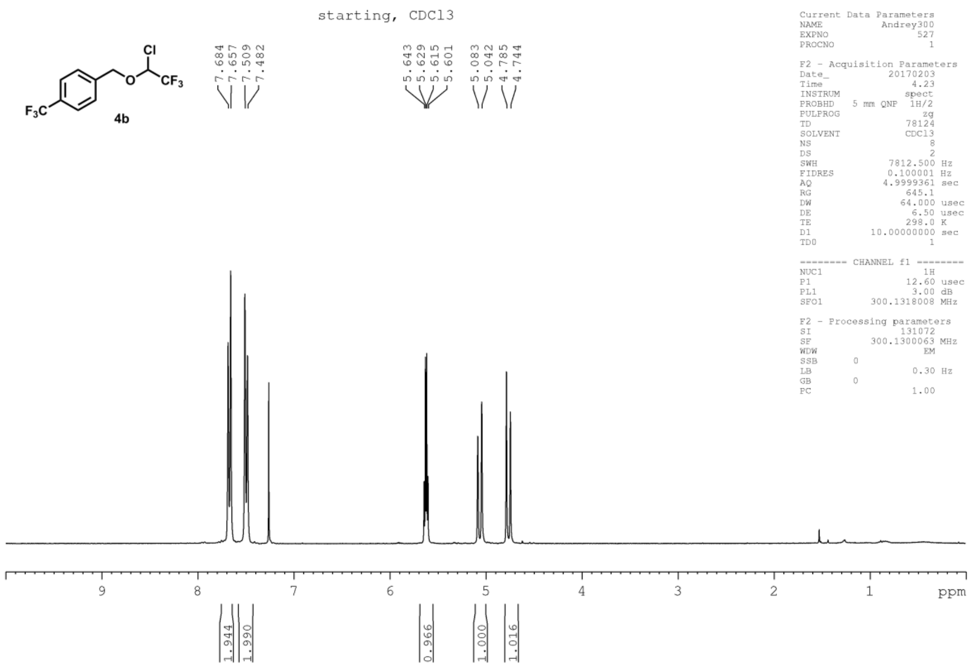

Supplementary figure 9. ^{1}H NMR spectra of **4b** in $CDCl_3$

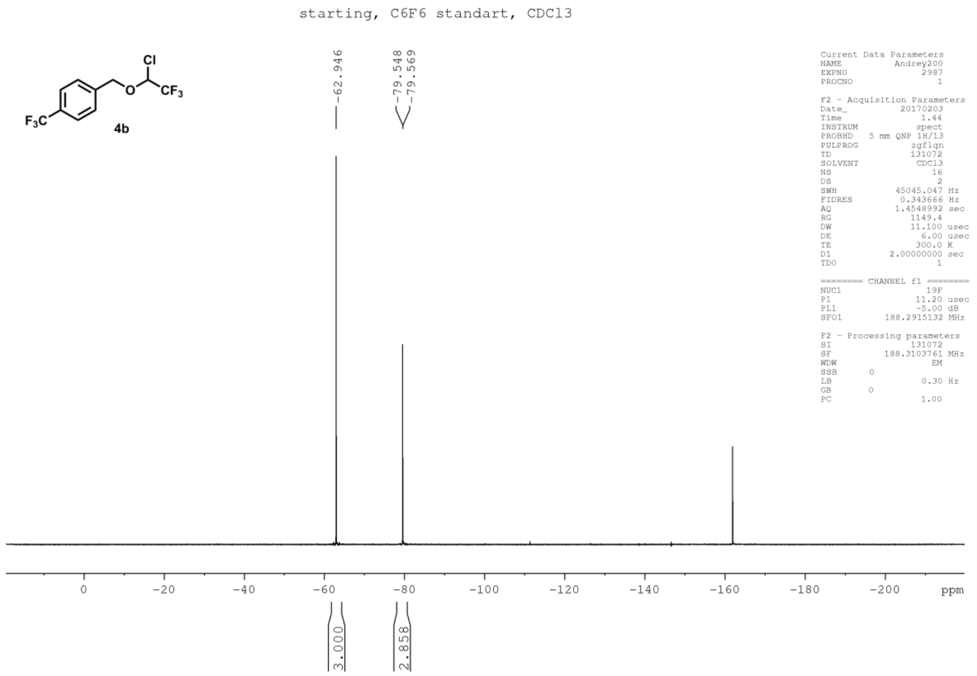

Supplementary figure 10. ^{19}F NMR spectra of **4b** in $CDCl_3$

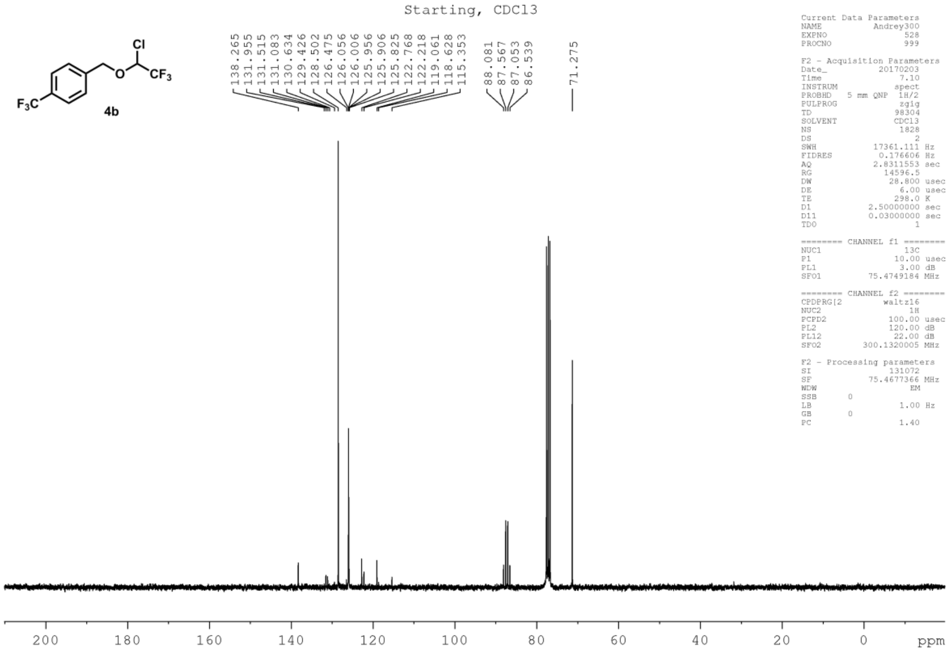

Supplementary figure 11. ^{13}C NMR spectra of **4b** in $CDCl_3$

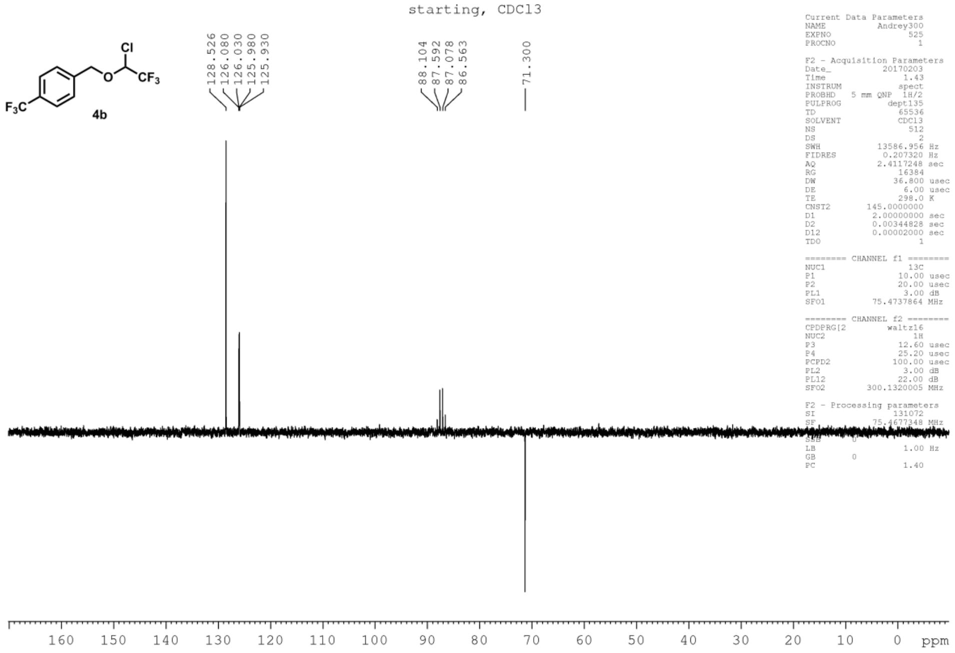

Supplementary figure 12. DEPT-135 NMR spectra of **4b** in $CDCl_3$

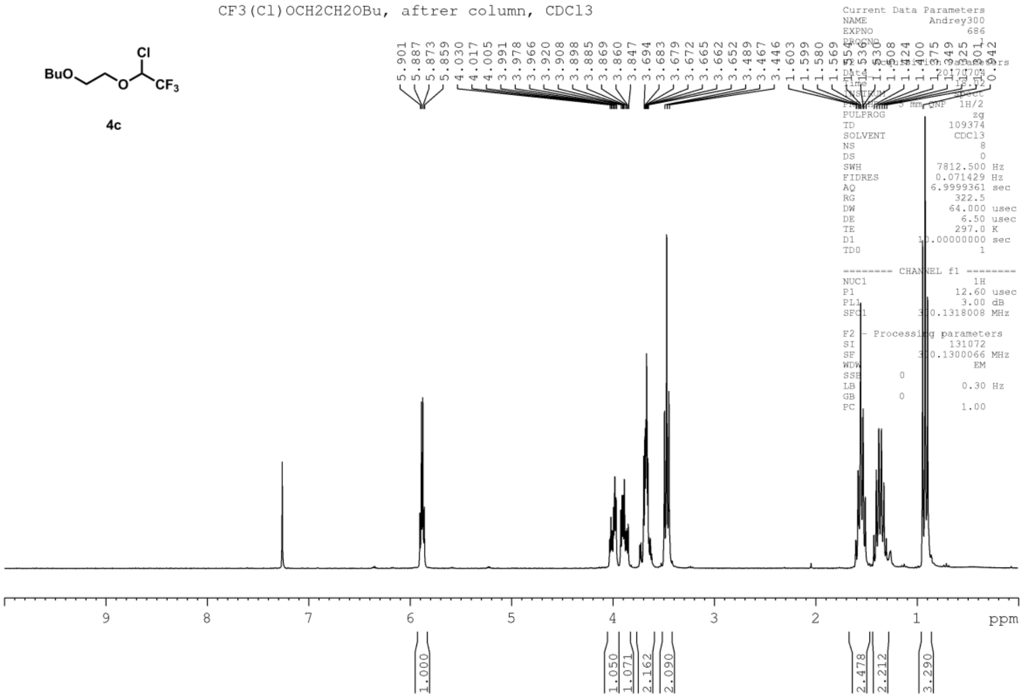

Supplementary figure 13. ^{1}H NMR spectra of **4c** in $CDCl_3$

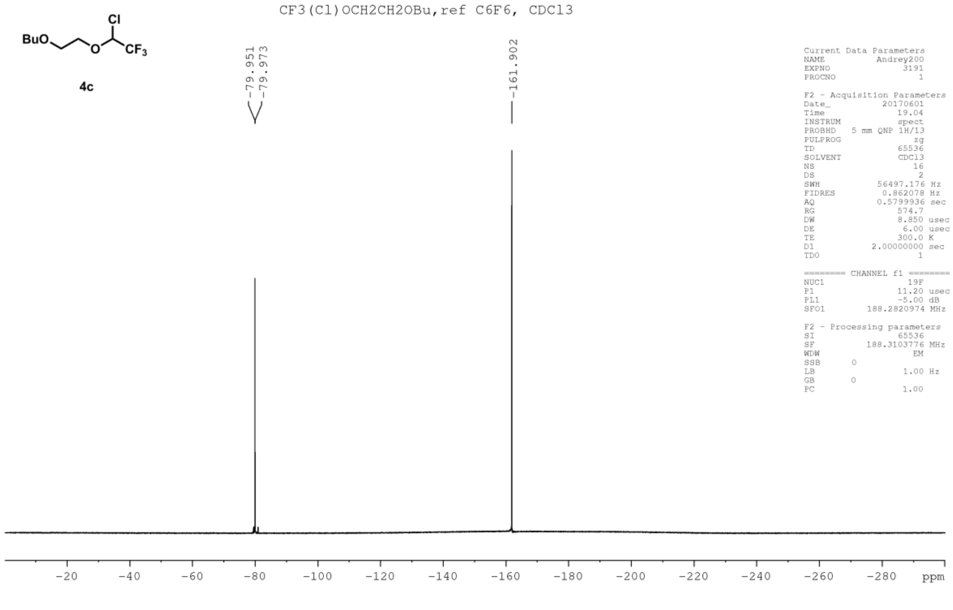

Supplementary figure 14. ^{19}F NMR spectra of **4c** in $CDCl_3$

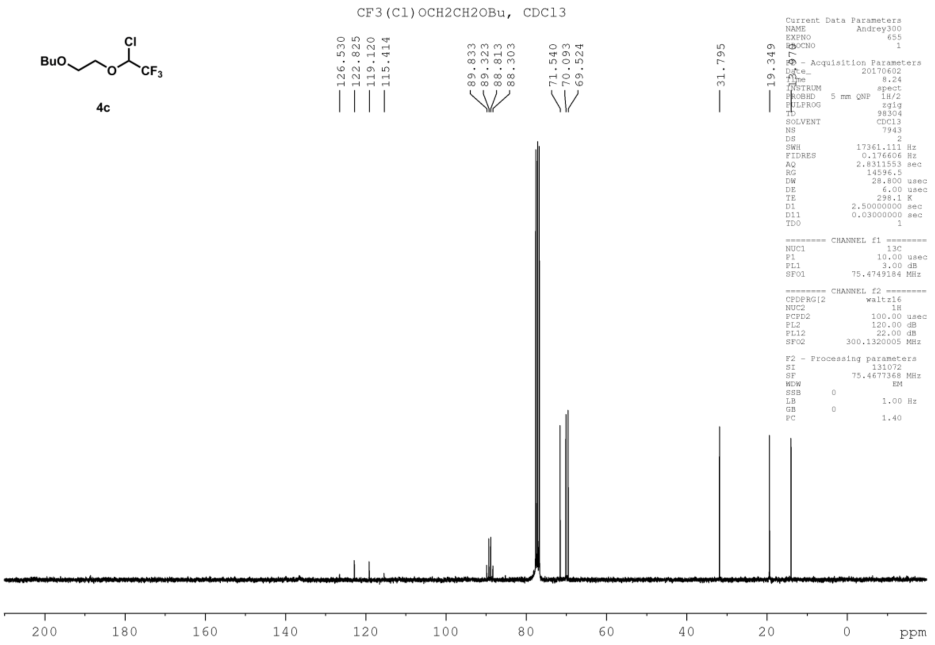

Supplementary figure 15. ^{13}C NMR spectra of **4c** in $CDCl_3$

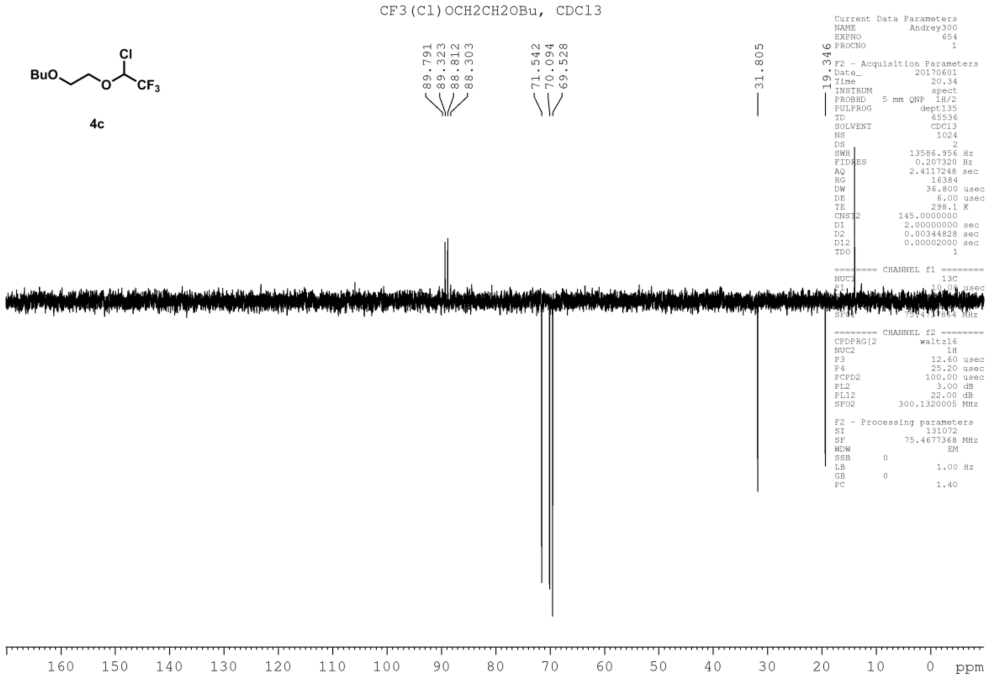

Supplementary figure 16. DEPT-135 NMR spectra of **4c** in $CDCl_3$

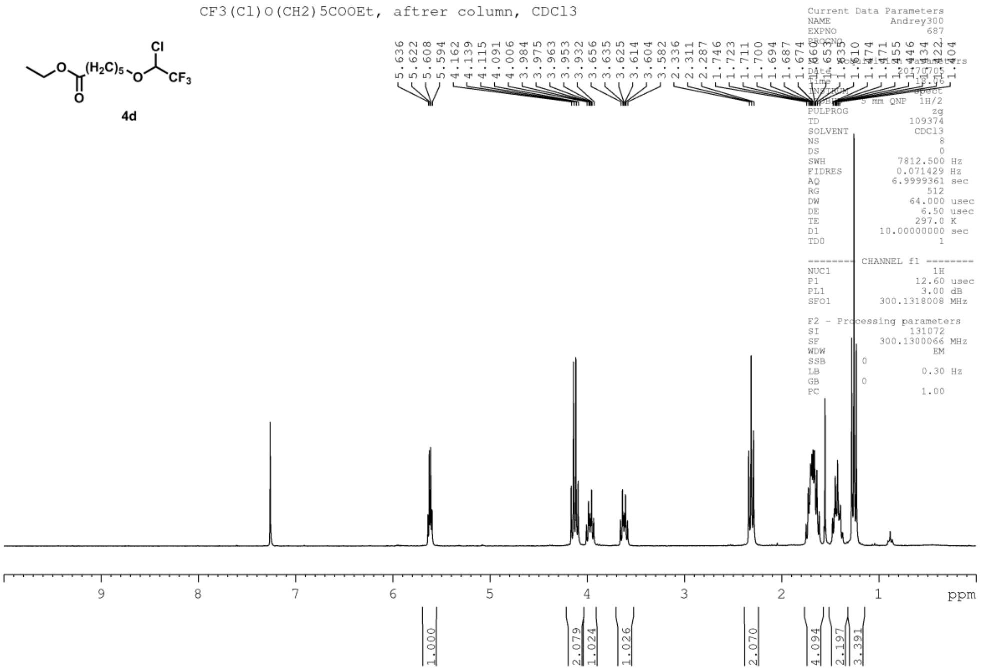

Supplementary figure 17. ^{1}H NMR spectra of **4d** in $CDCl_3$

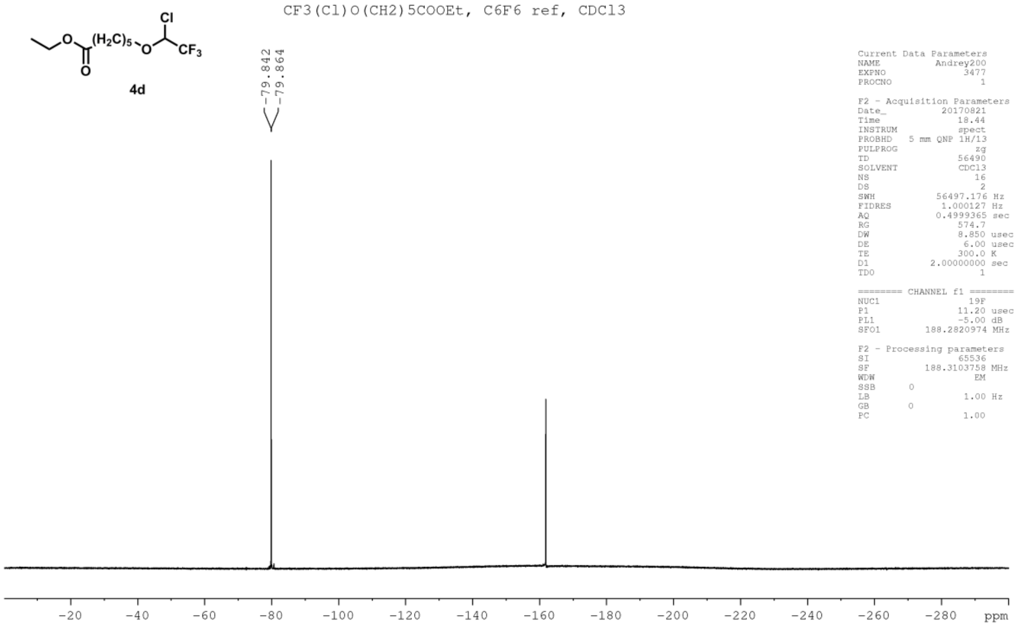

Supplementary figure 18. ^{19}F NMR spectra of **4d** in $CDCl_3$

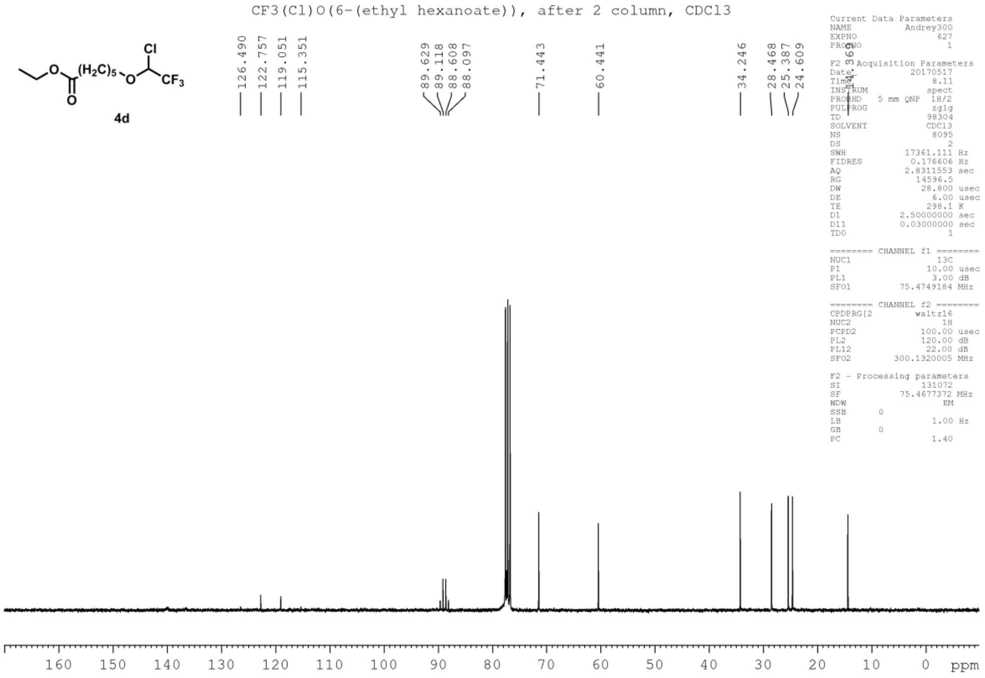

Supplementary figure 19. ^{13}**C** NMR spectra of **4d** in $CDCl_3$

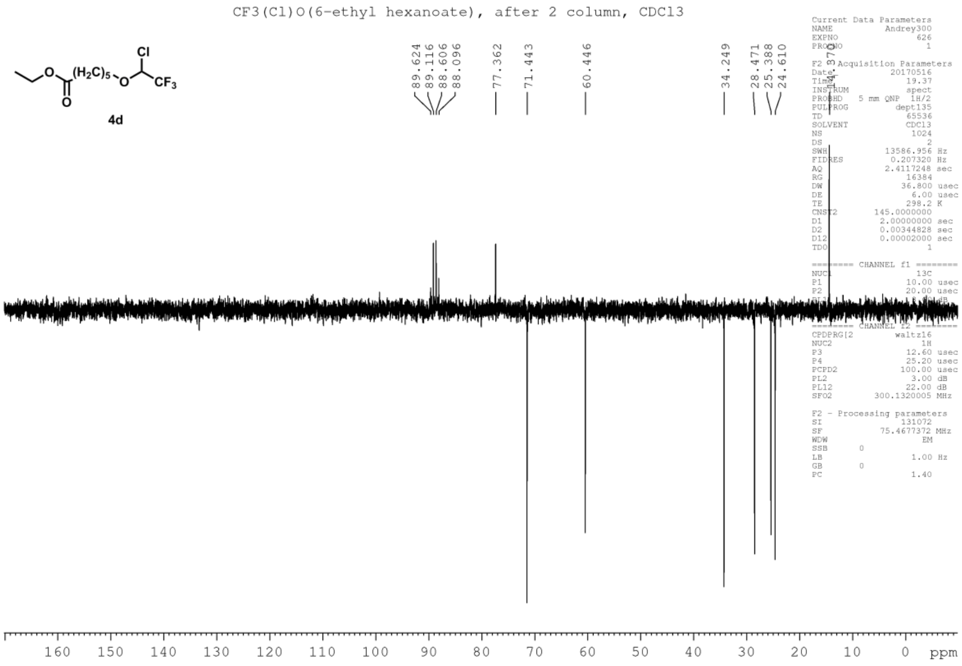

Supplementary figure 20. DEPT-135 NMR spectra of **4d** in $CDCl_3$

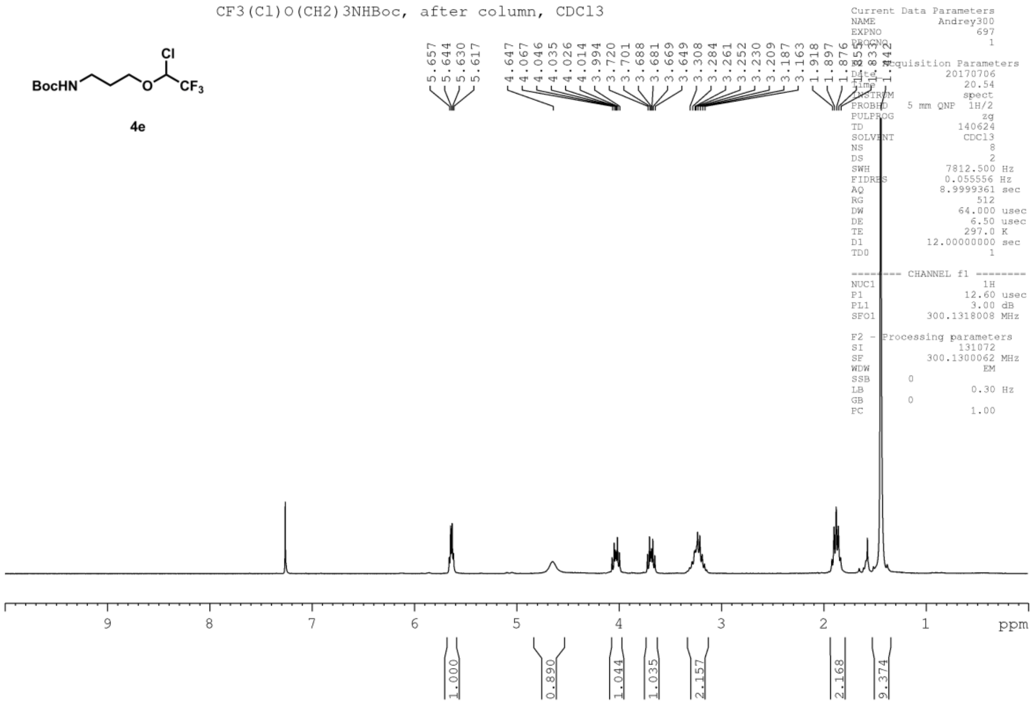

Supplementary figure 21. ^{1}H NMR spectra of **4e** in $CDCl_3$

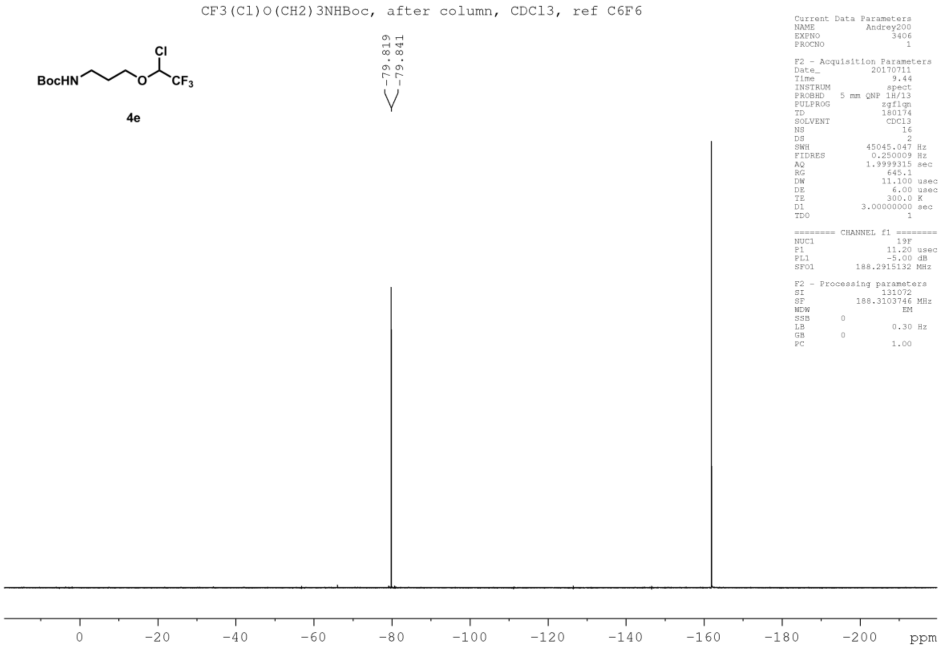

Supplementary figure 22. ^{19}F NMR spectra of **4e** in $CDCl_3$

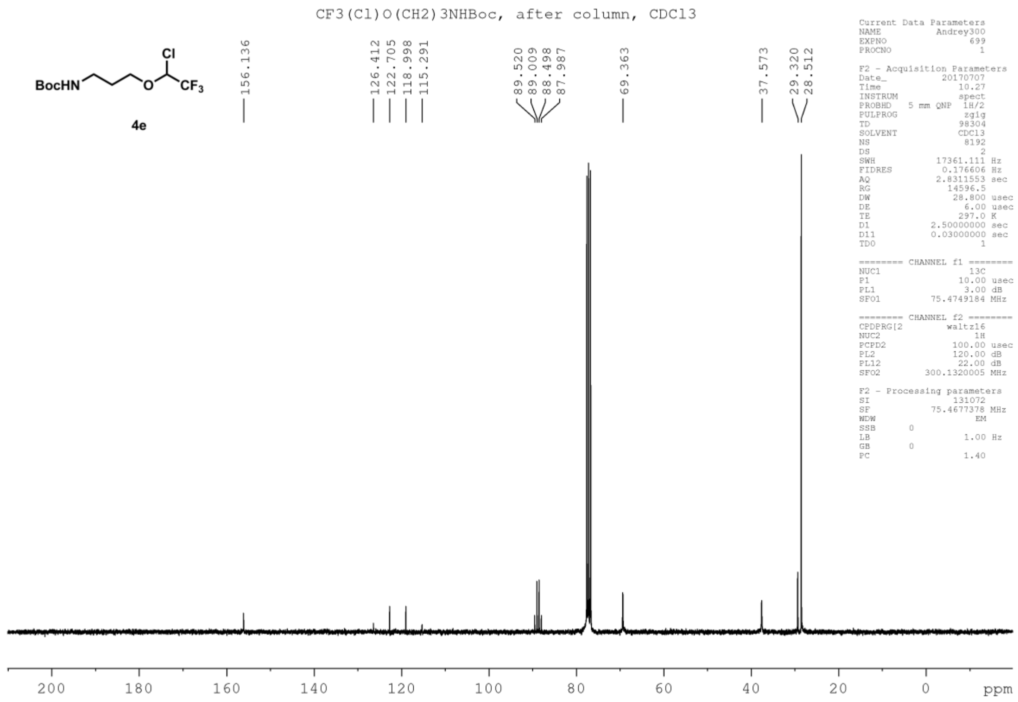

Supplementary figure 23. ^{13}C NMR spectra of **4e** in $CDCl_3$

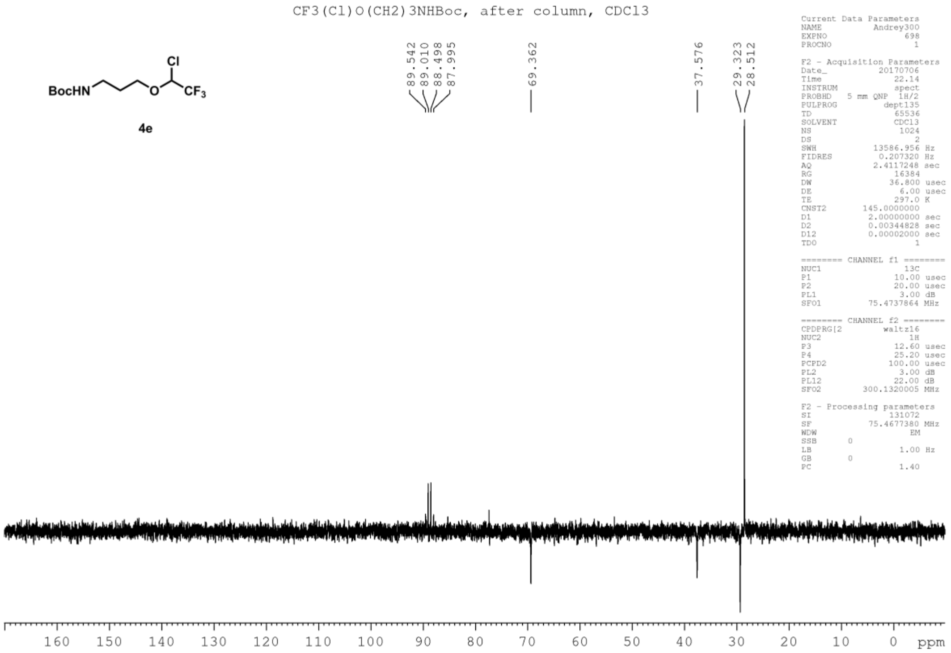

Supplementary figure 24. DEPT-135 NMR spectra of **4e** in $CDCl_3$

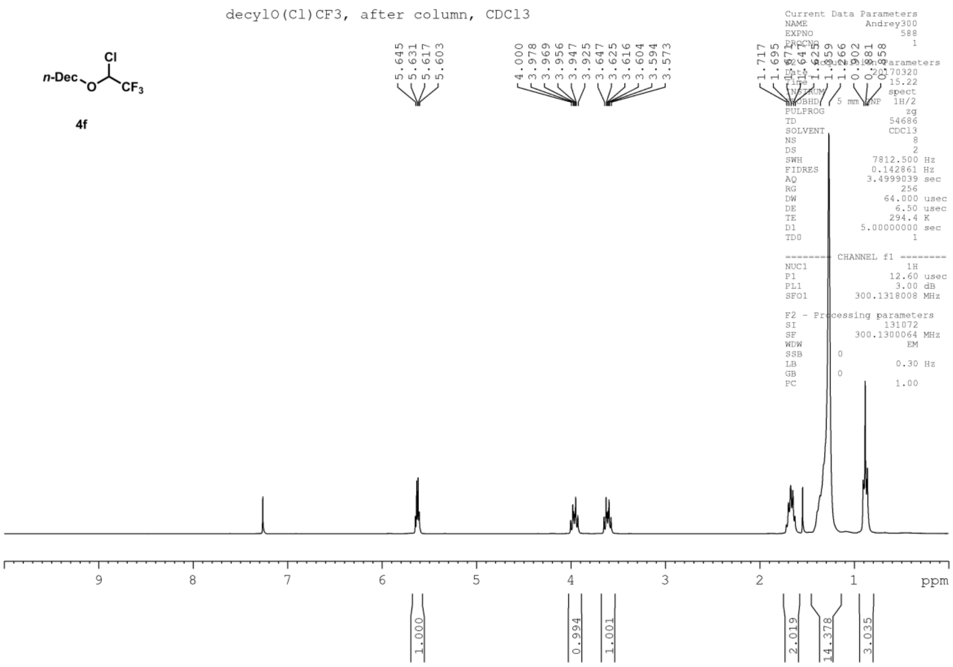

Supplementary figure 25. ^{1}H NMR spectra of **4f** in $CDCl_3$

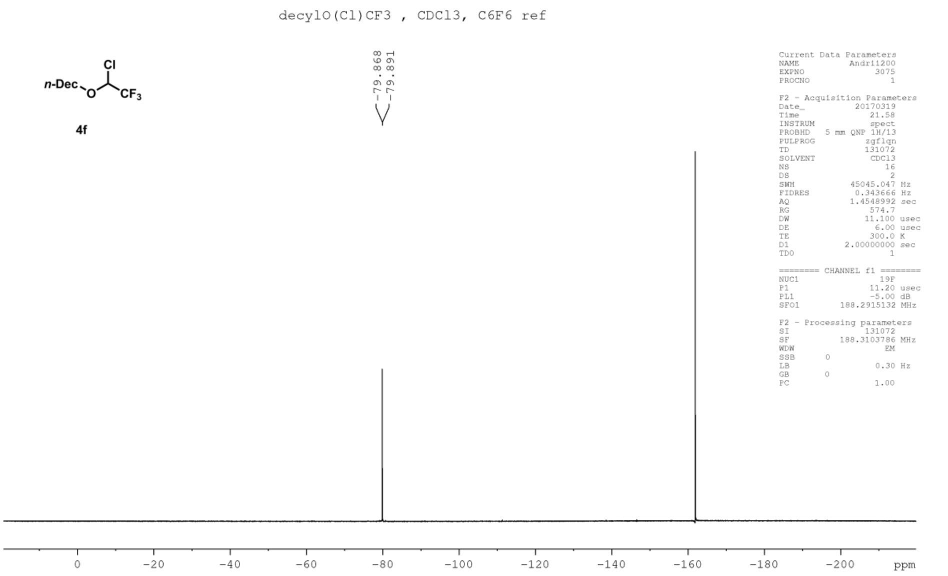

Supplementary figure 26. ^{19}F NMR spectra of **4f** in $CDCl_3$

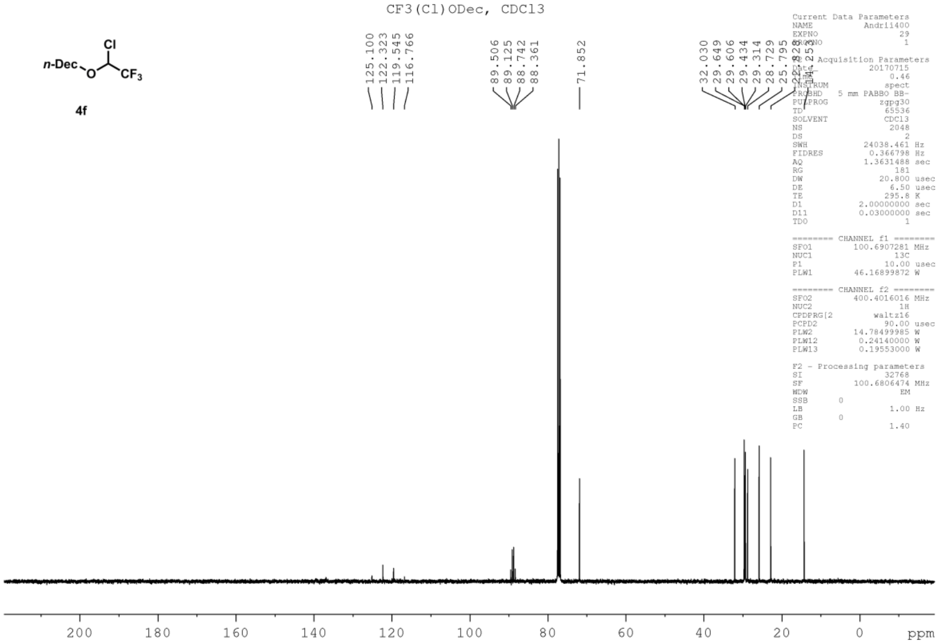

Supplementary figure 27. ^{13}C NMR spectra of **4f** in $CDCl_3$

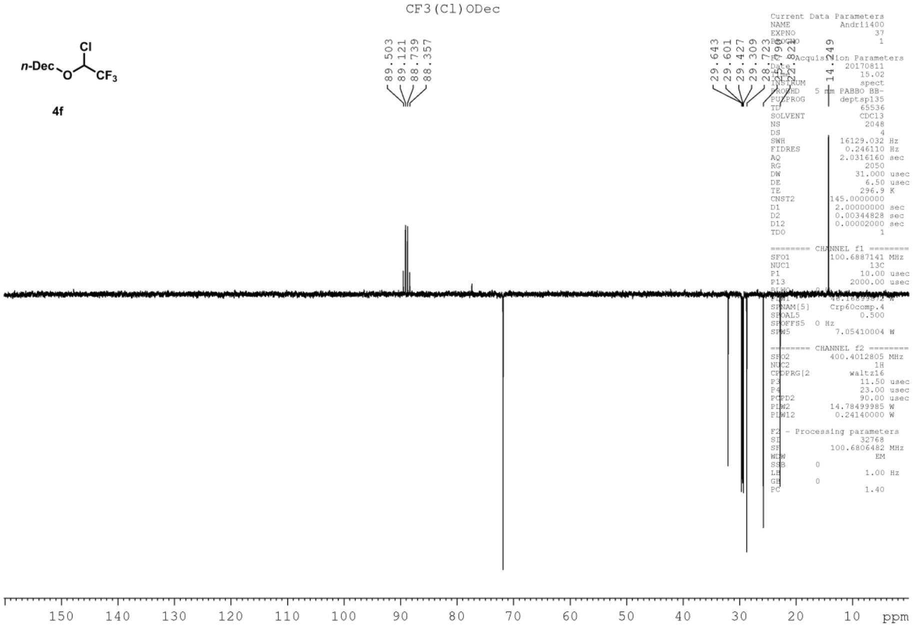

Supplementary figure 28. DEPT-135 NMR spectra of **4f** in $CDCl_3$

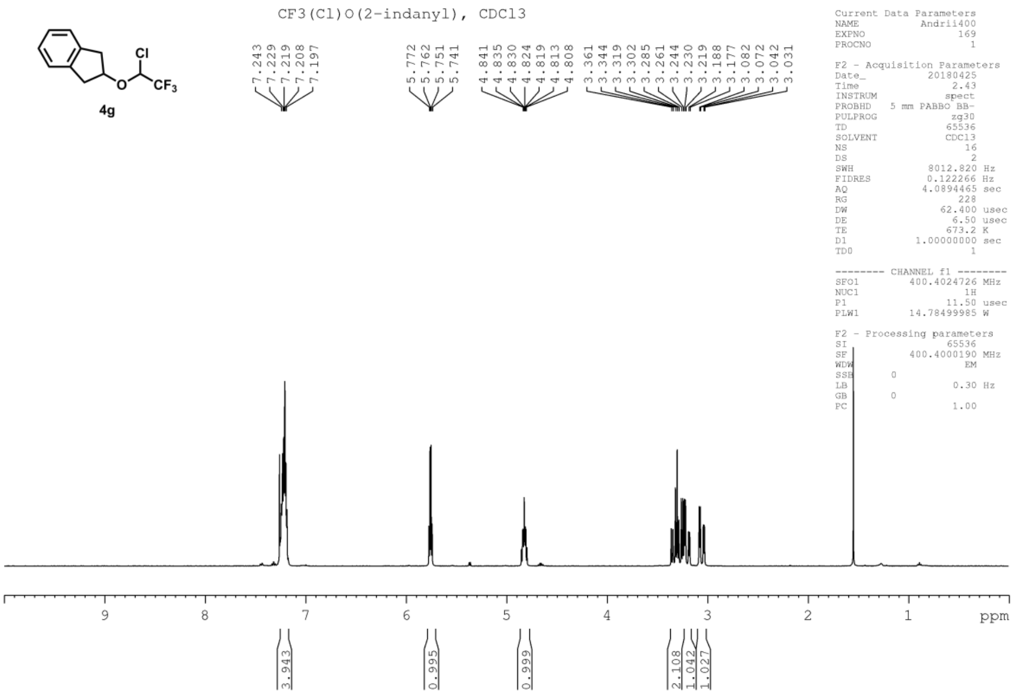

Supplementary figure 29. ^{1}H NMR spectra of **4g** in $CDCl_3$

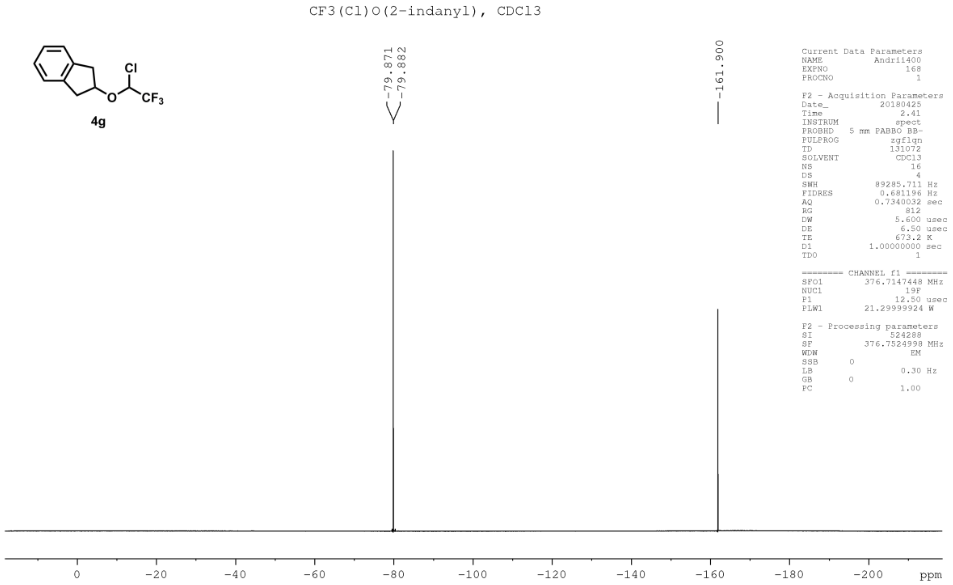

Supplementary figure 30. ^{19}F NMR spectra of **4g** in $CDCl_3$

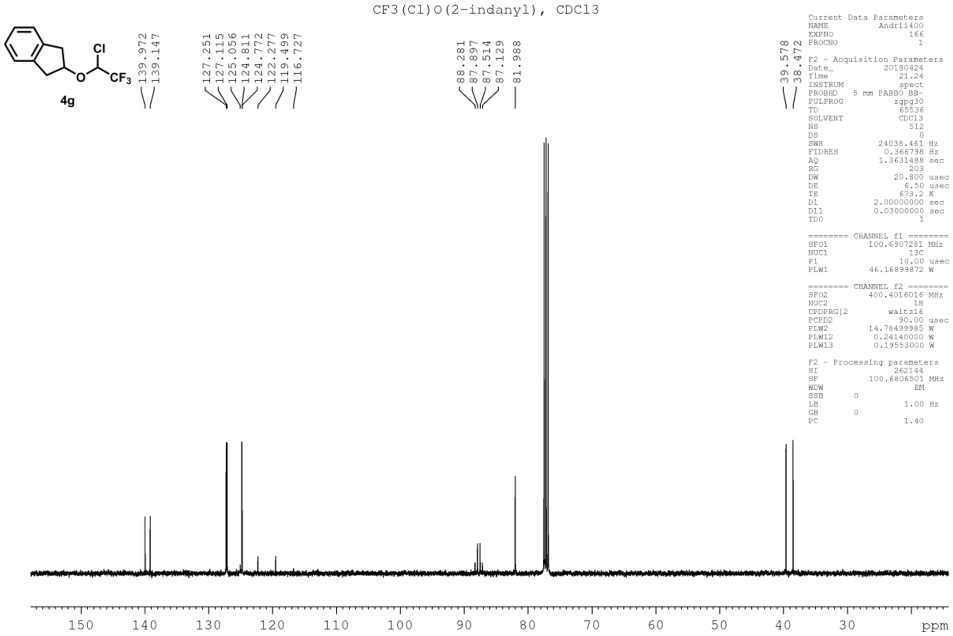

Supplementary figure 31. ^{13}C NMR spectra of **4g** in $CDCl_3$

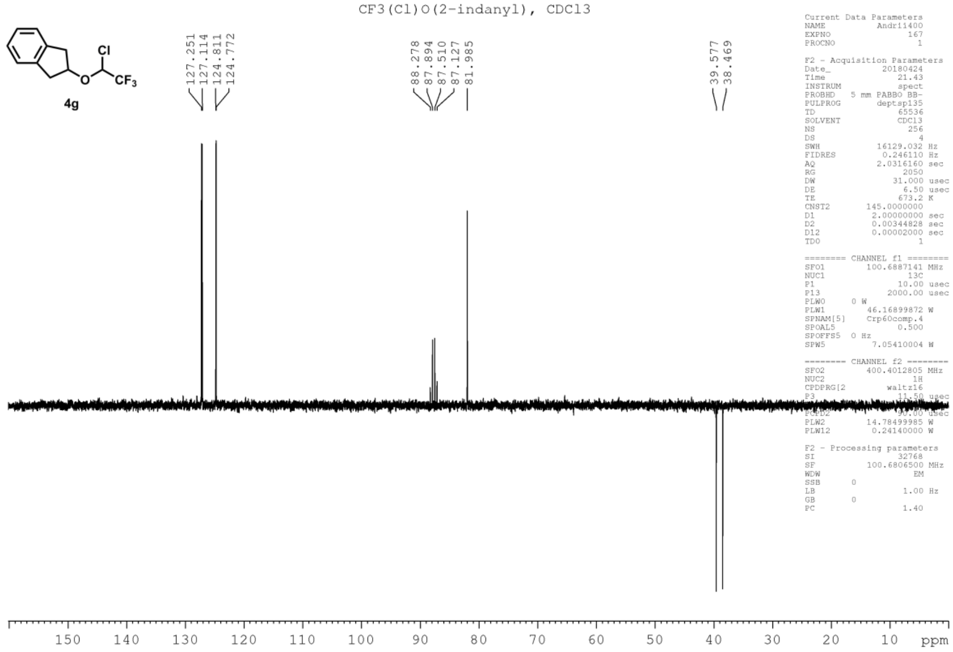

Supplementary figure 32. DEPT-135 NMR spectra of **4g** in $CDCl_3$

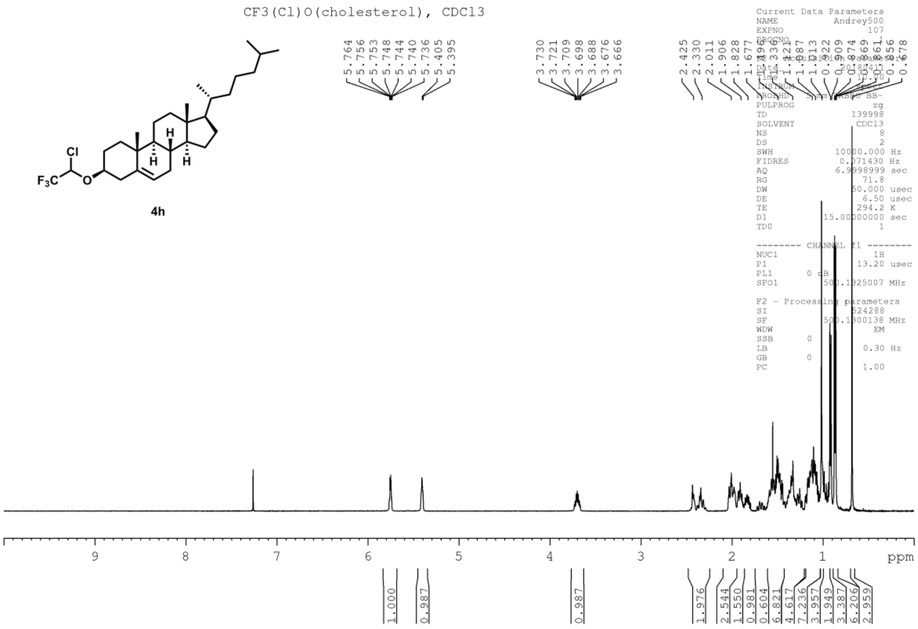

Supplementary figure 33. ^{1}H NMR spectra of **4h** in $CDCl_3$

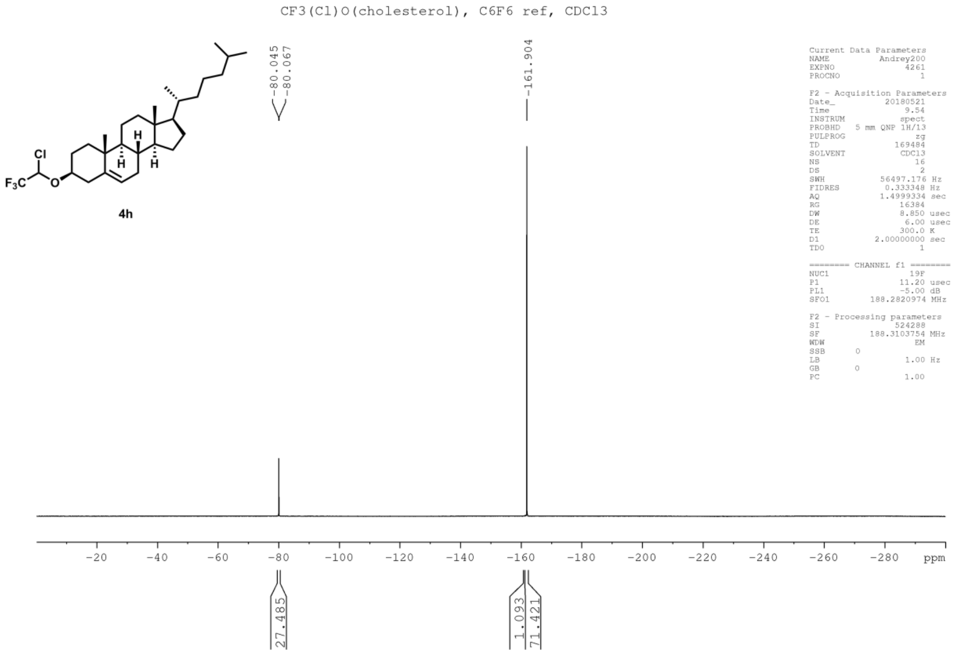

Supplementary figure 34. ^{19}FNMR spectra of **4h** in $CDCl_3$

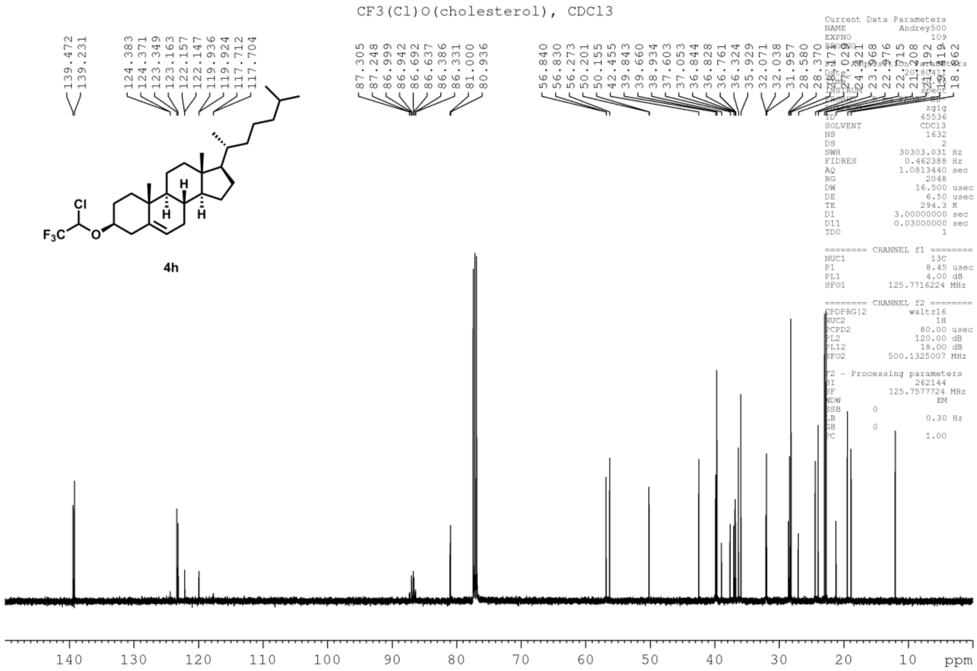

Supplementary figure 35. ^{13}C NMR spectra of **4h** in $CDCl_3$

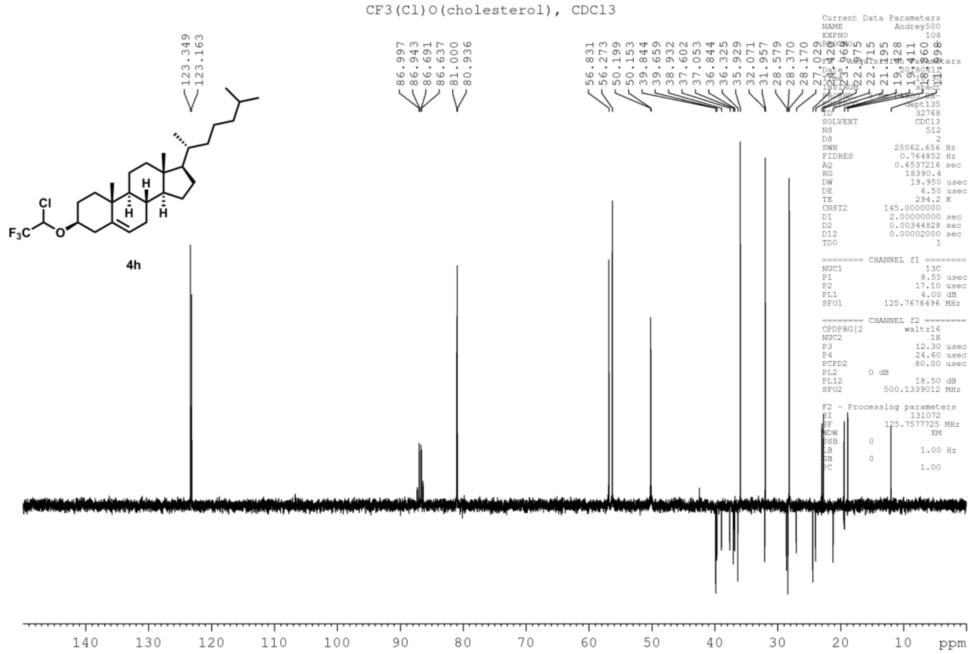

Supplementary figure 26. DEPT-135 NMR spectra of **4h** in $CDCl_3$

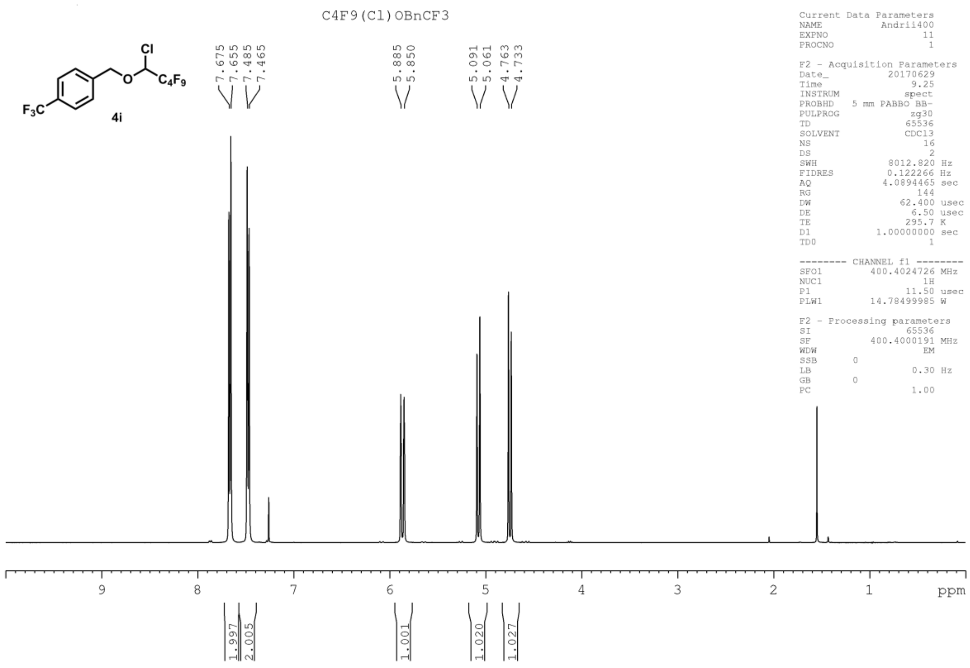

Supplementary figure 37 ^{1}H NMR spectra of **4i** in $CDCl_3$

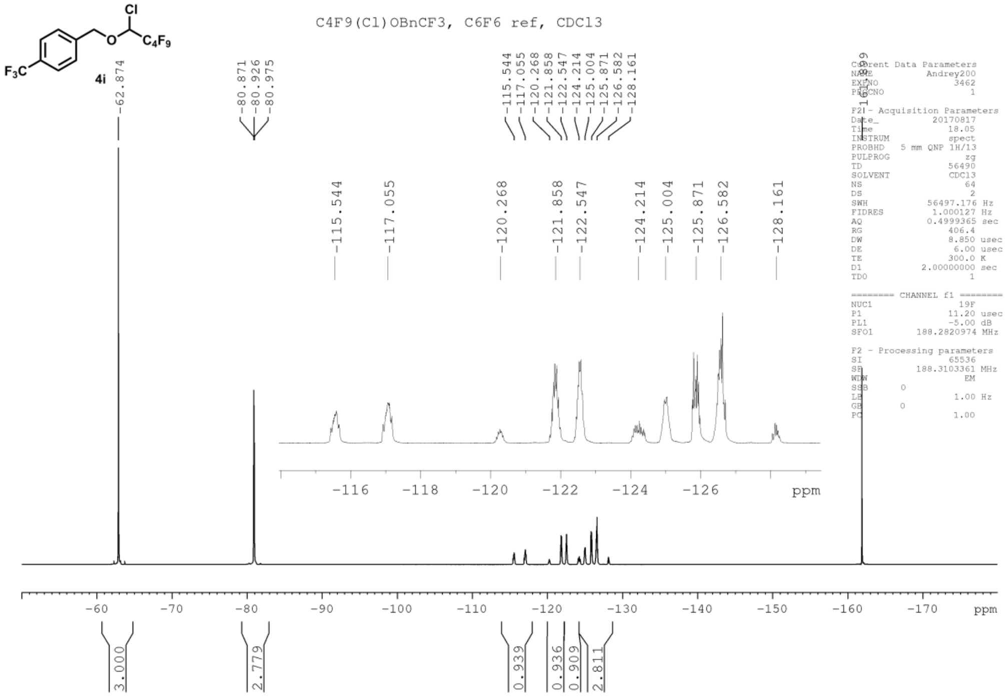

Supplementary figure 38. ^{19}F NMR spectra of **4i** in $CDCl_3$

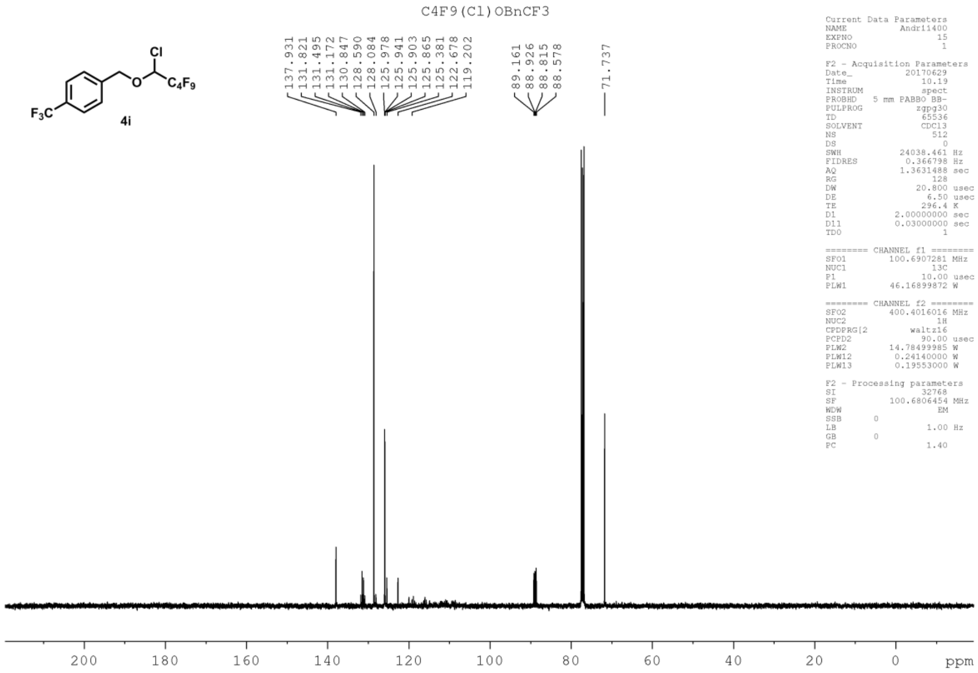

Supplementary figure 39. ^{13}C NMR spectra of **4i** in $CDCl_3$

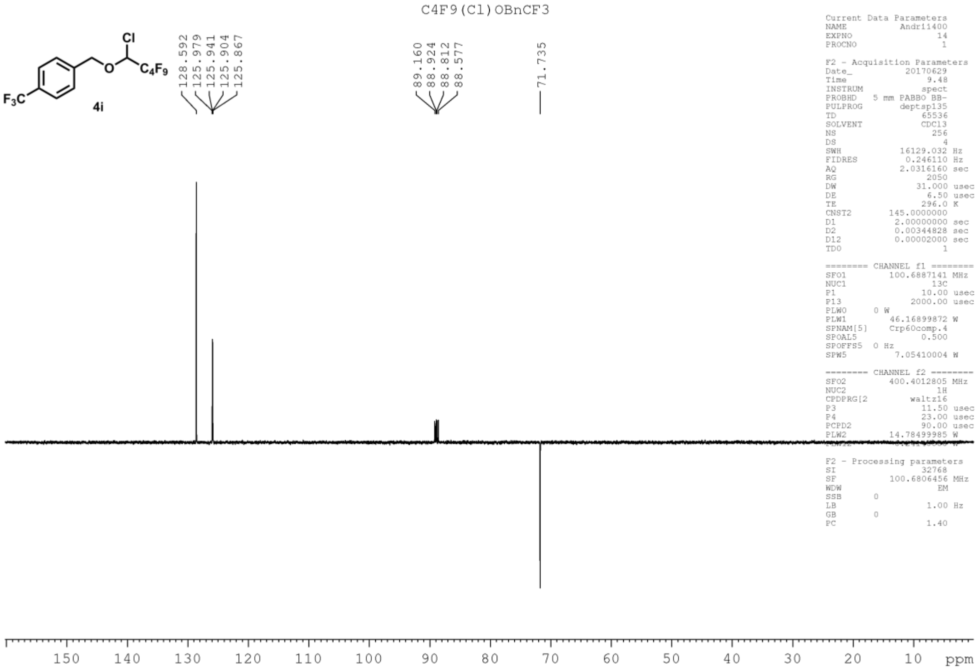

Supplementary figure 40. DEPT-135 NMR spectra of **4i** in $CDCl_3$

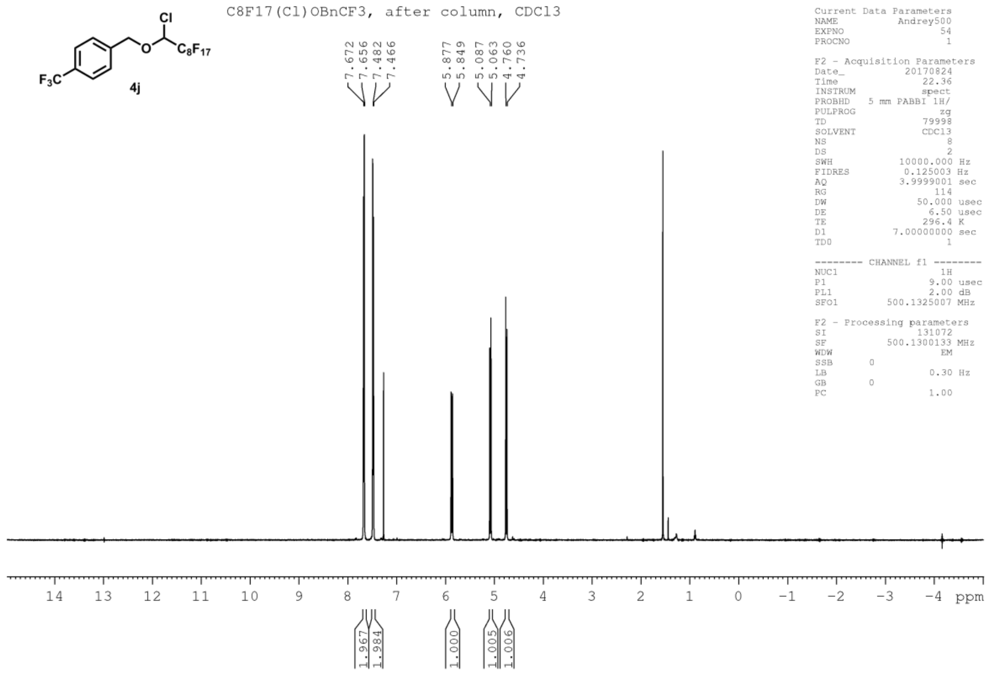

Supplementary figure 41. ^{1}H NMR spectra of **4j** in $CDCl_3$

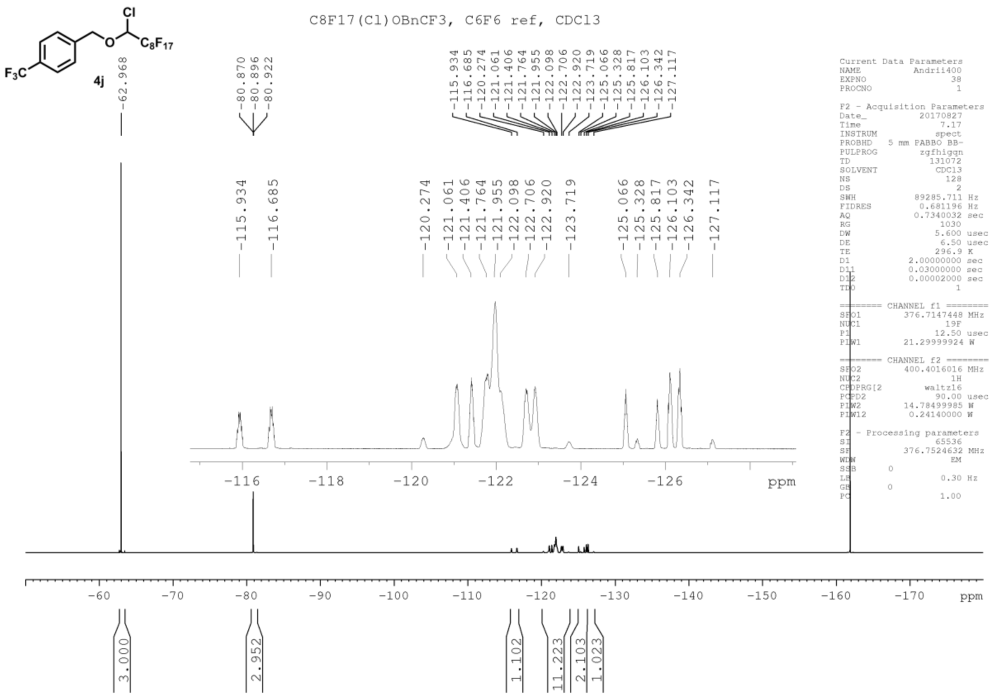

Supplementary figure 42. ^{19}F NMR spectra of **4j** in $CDCl_3$

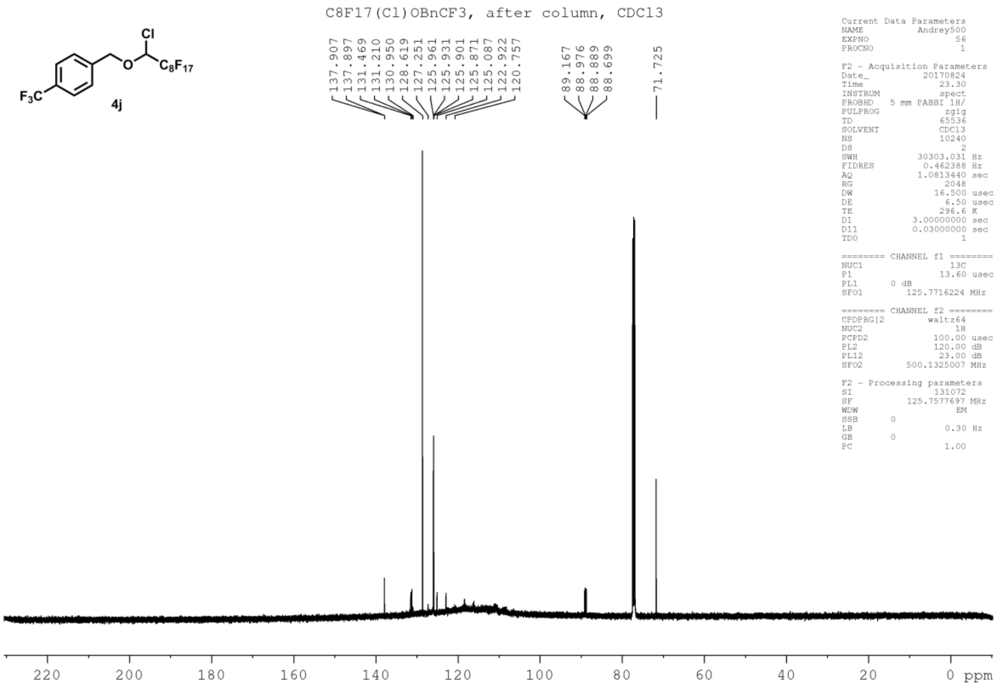

Supplementary figure 43. ^{13}C NMR spectra of **4j** in $CDCl_3$

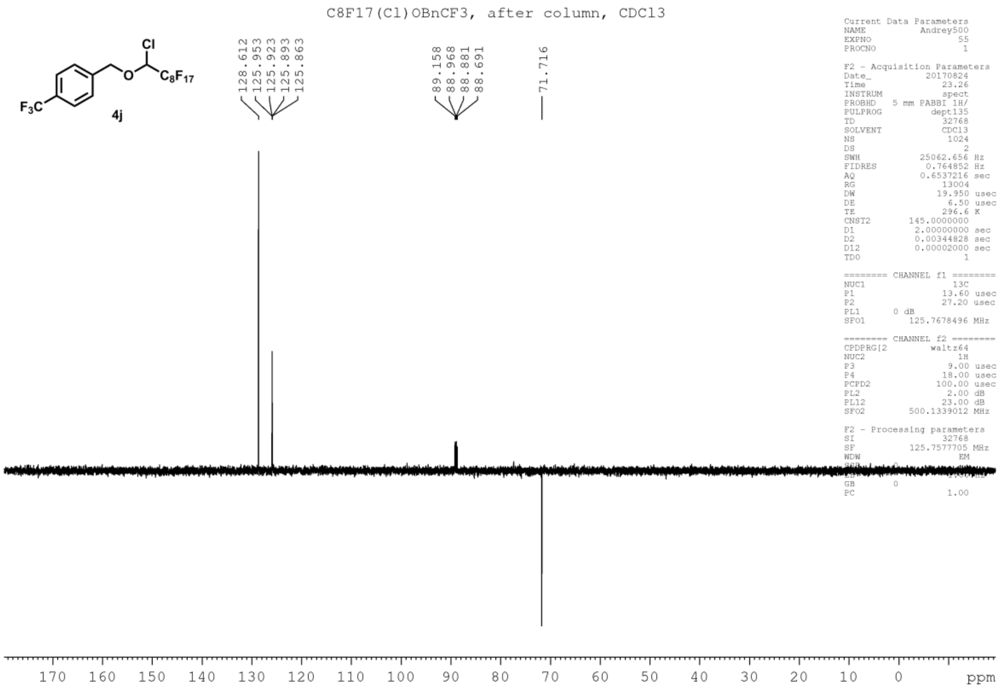

Supplementary figure 44. DEPT-135 NMR spectra of **4j** in $CDCl_3$

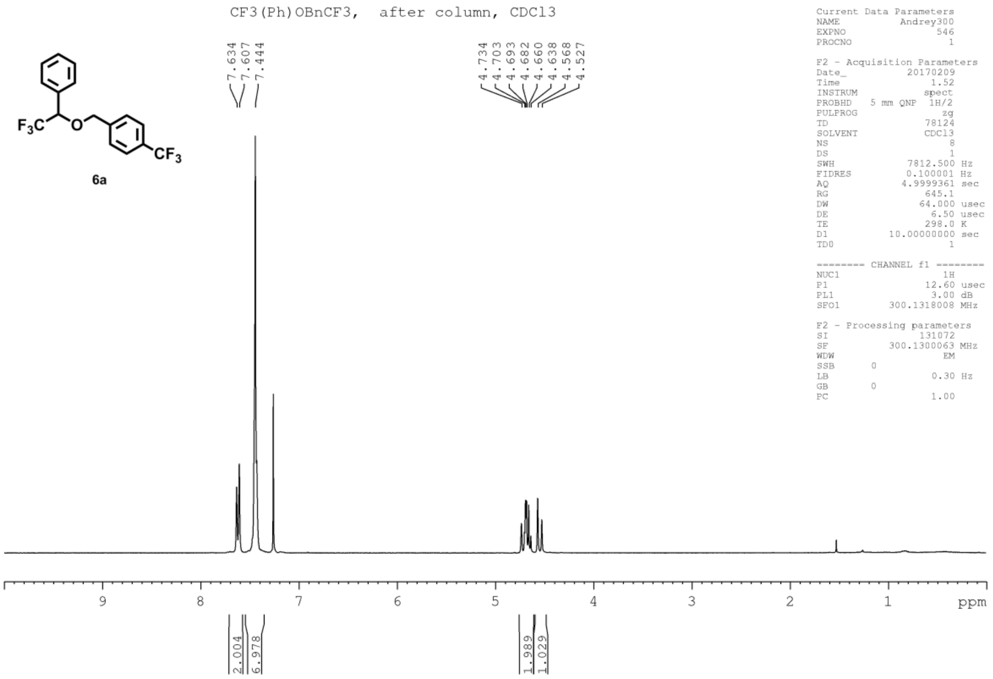

Supplementary figure 45. ^{1}H NMR spectra of **6a** in $CDCl_3$

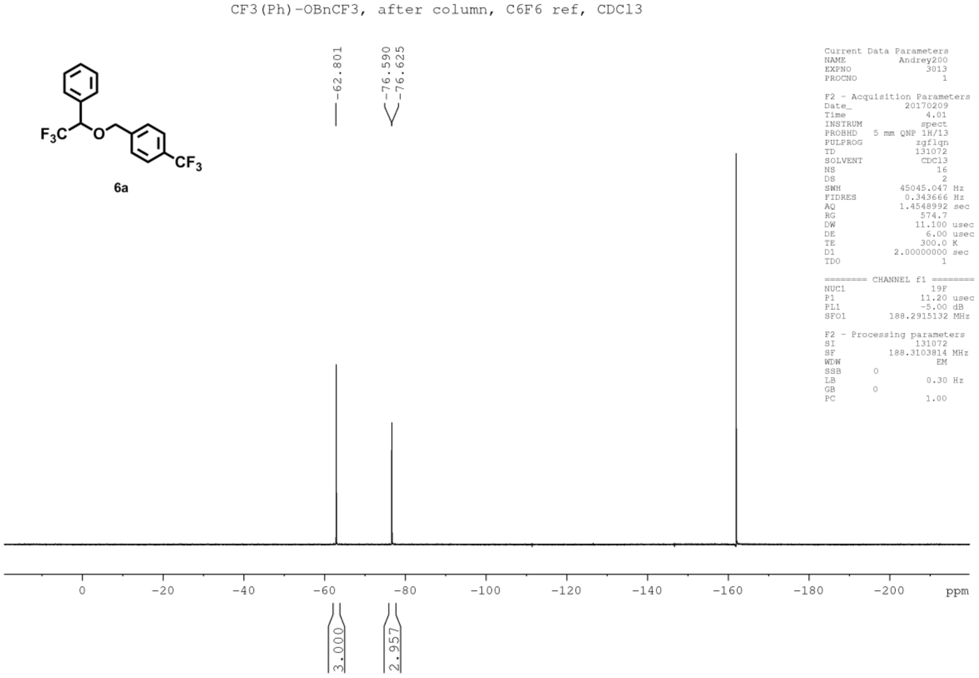

Supplementary figure 46. ^{19}F NMR spectra of **6a** in $CDCl_3$

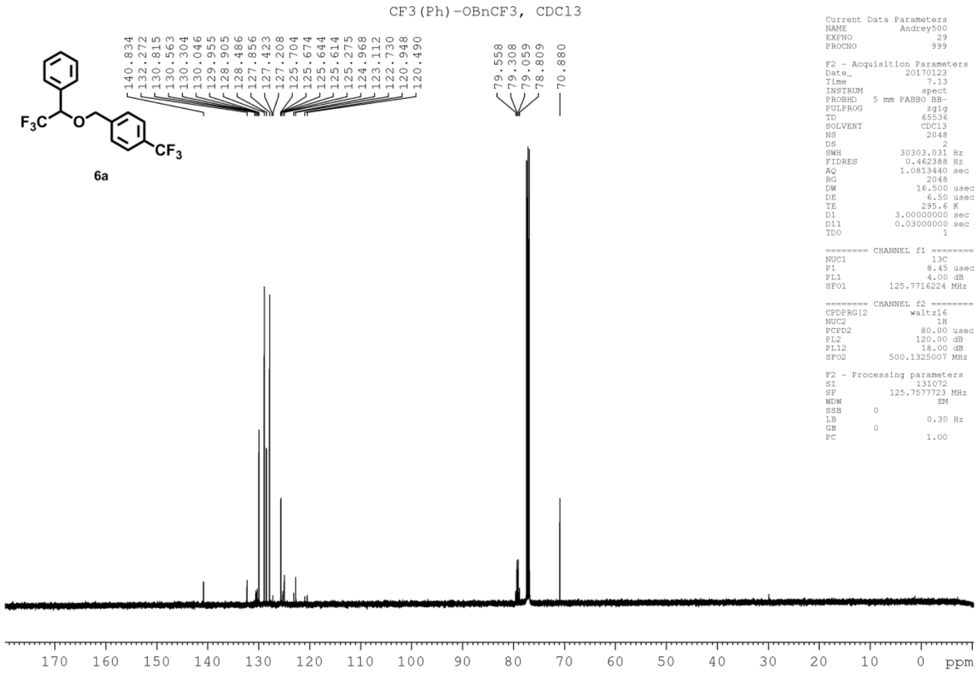

Supplementary figure 47. ^{13}C NMR spectra of **4e** in $CDCl_3$

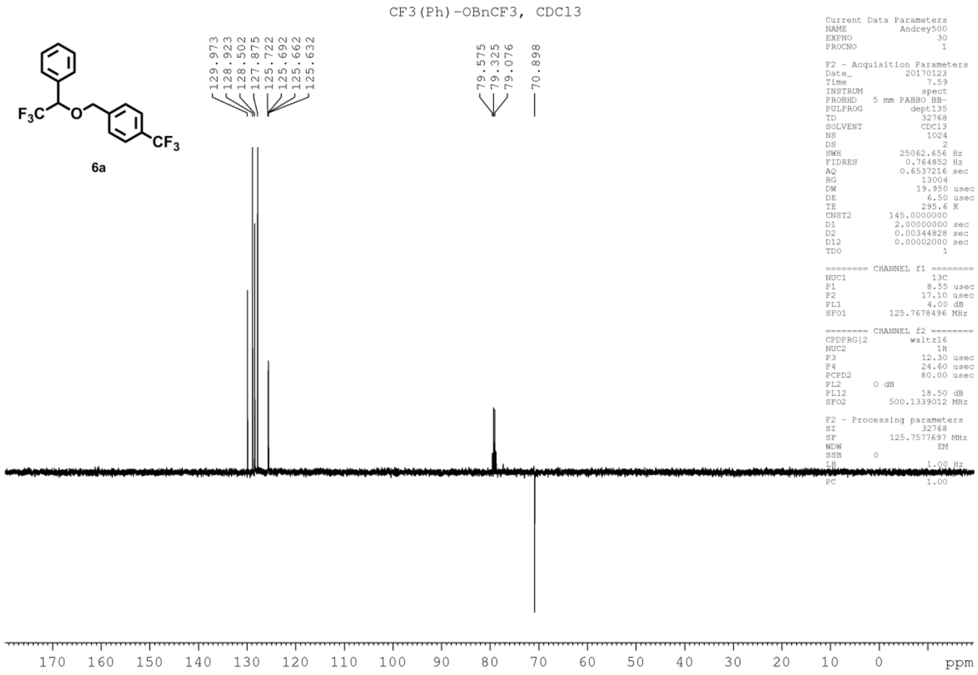

Supplementary figure 48. DEPT-135 NMR spectra of **6a** in $CDCl_3$

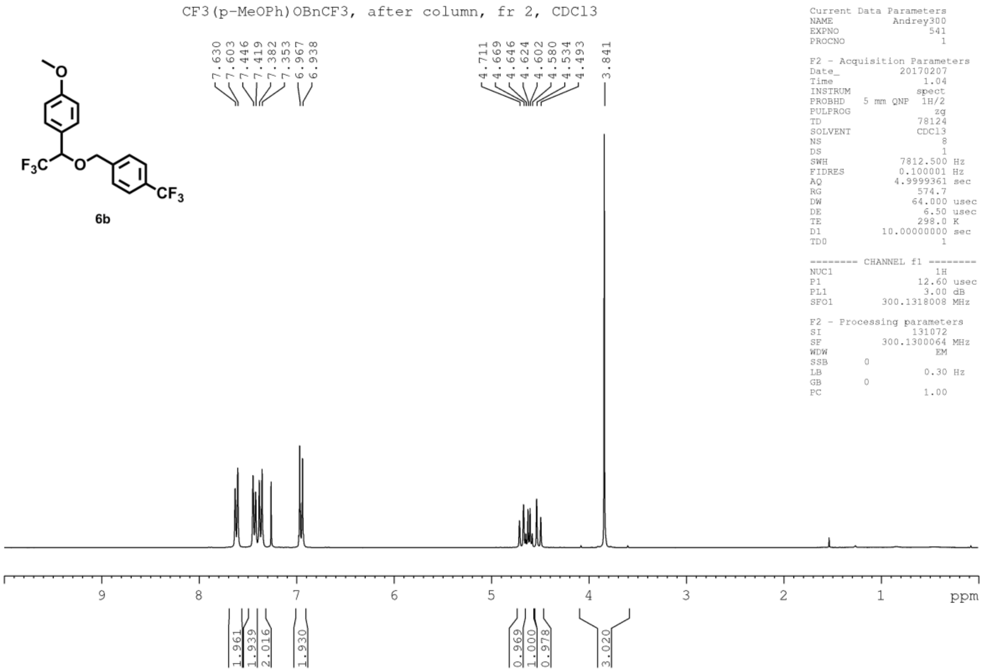

Supplementary figure 49. ^{1}H NMR spectra of **6b** in $CDCl_3$

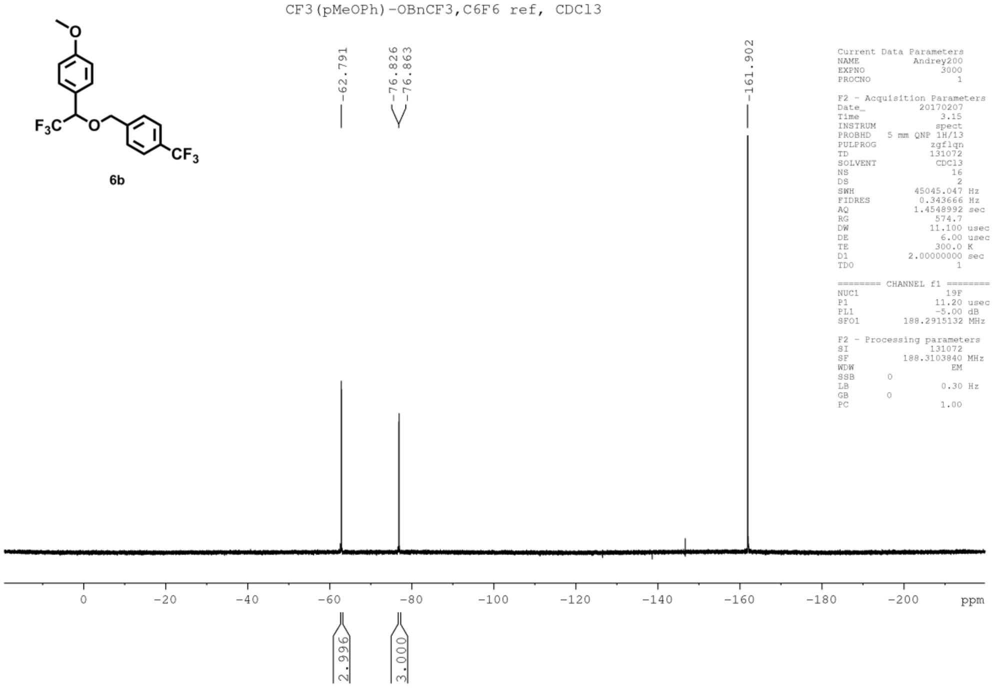

Supplementary figure 50. ^{19}F NMR spectra of **6b** in $CDCl_3$

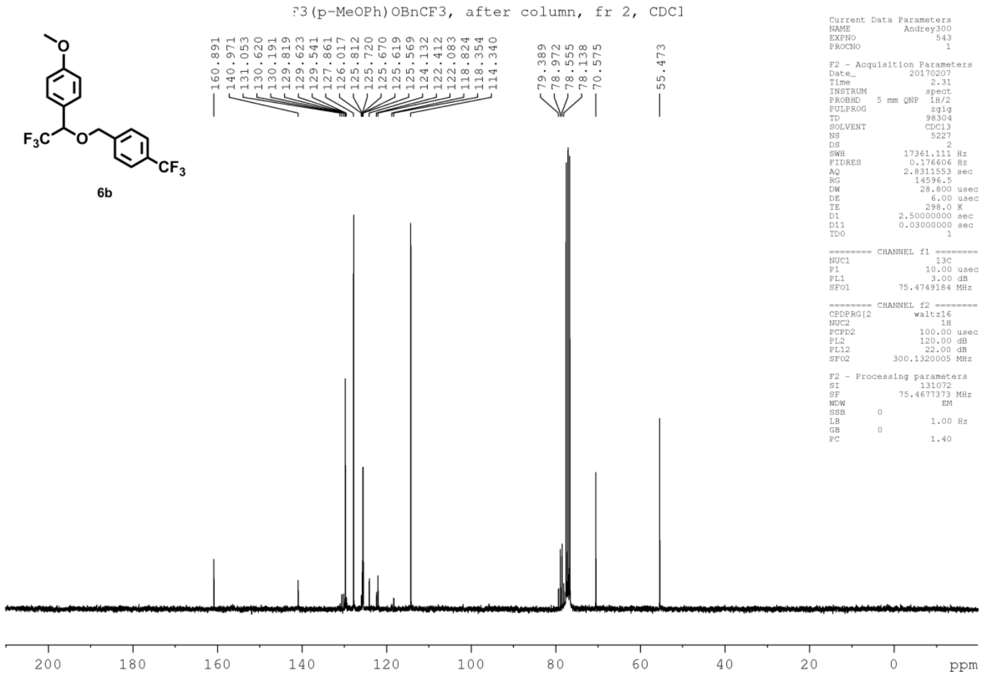

Supplementary figure 51. ^{13}C NMR spectra of **6b** in $CDCl_3$

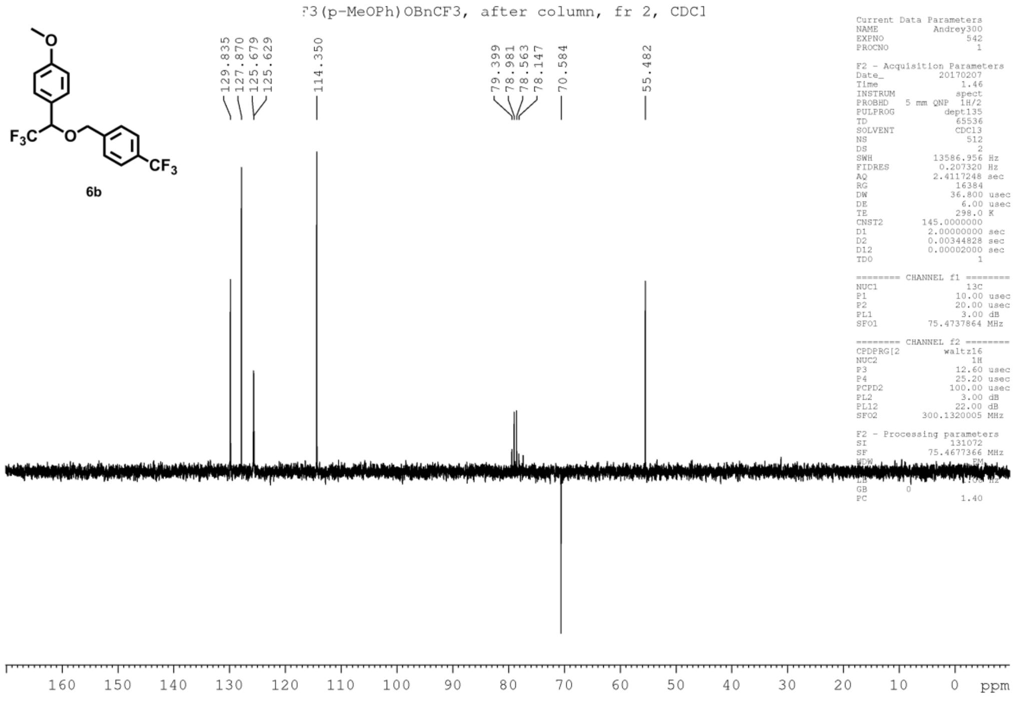

Supplementary figure 52. DEPT-135 NMR spectra of **6b** in $CDCl_3$

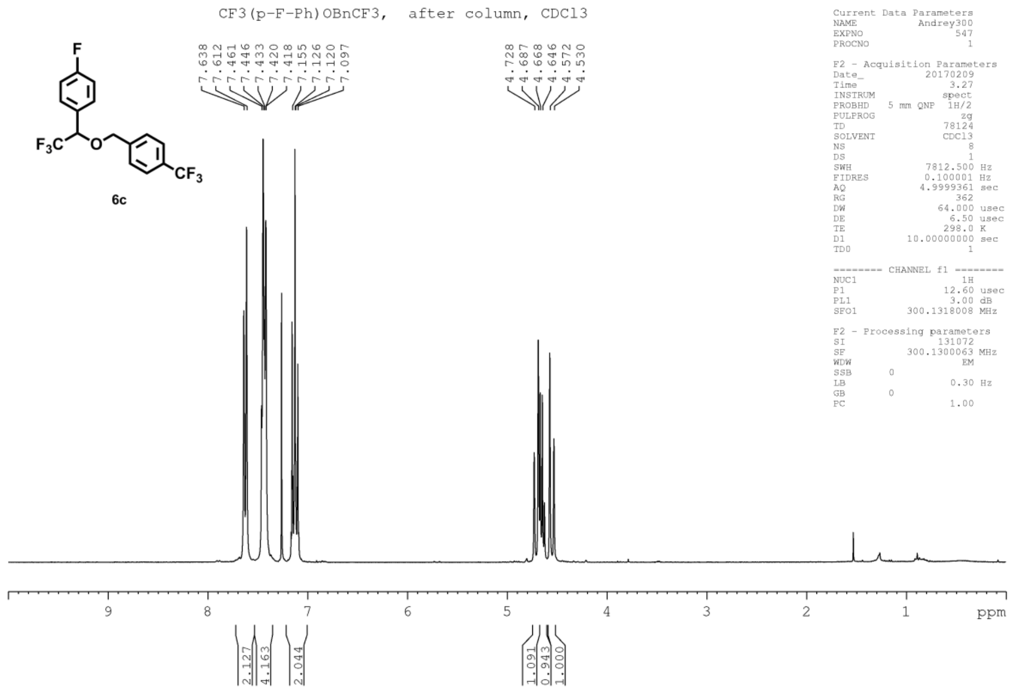

Supplementary figure 53. ^{1}H NMR spectra of **6c** in $CDCl_3$

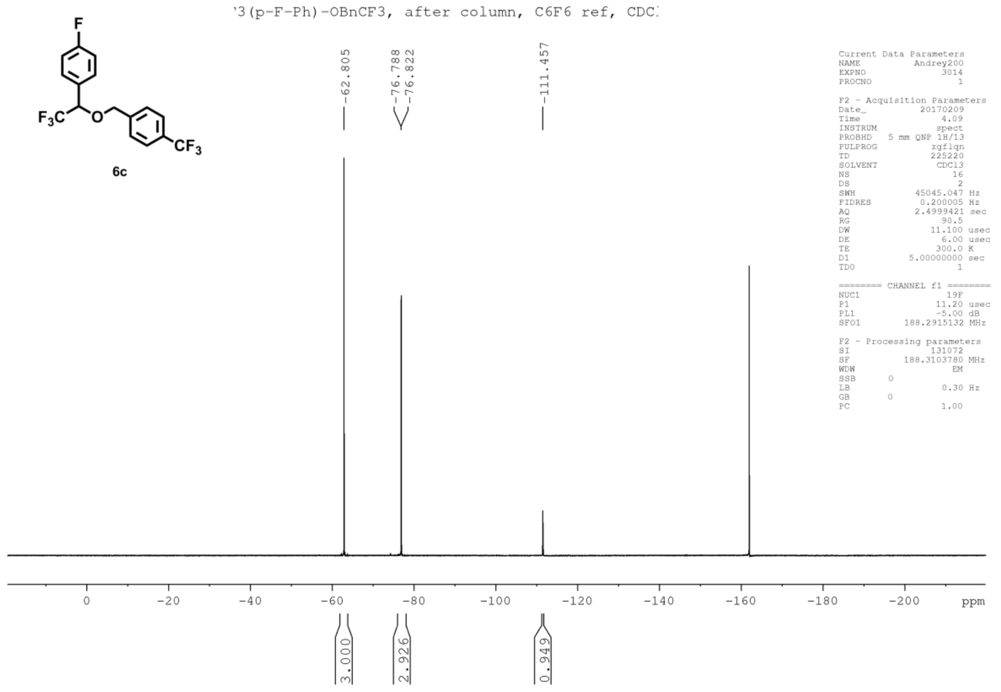

Supplementary figure 54. ^{19}F NMR spectra of **6c** in $CDCl_3$

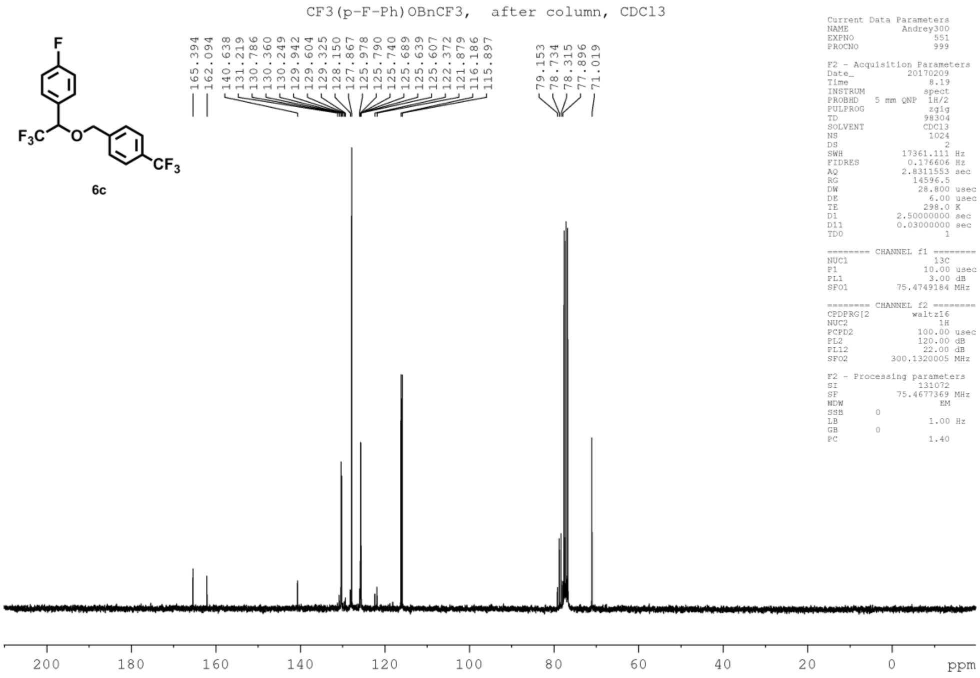

Supplementary figure 55. ^{13}C NMR spectra of **6c** in $CDCl_3$

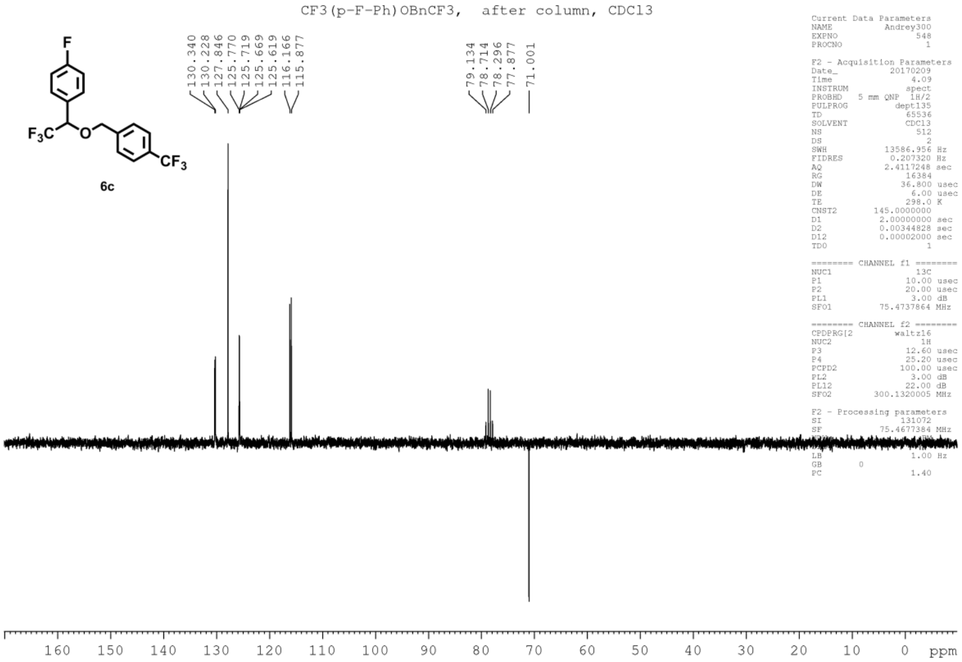

Supplementary figure 56. DEPT-135 NMR spectra of **6c** in $CDCl_3$

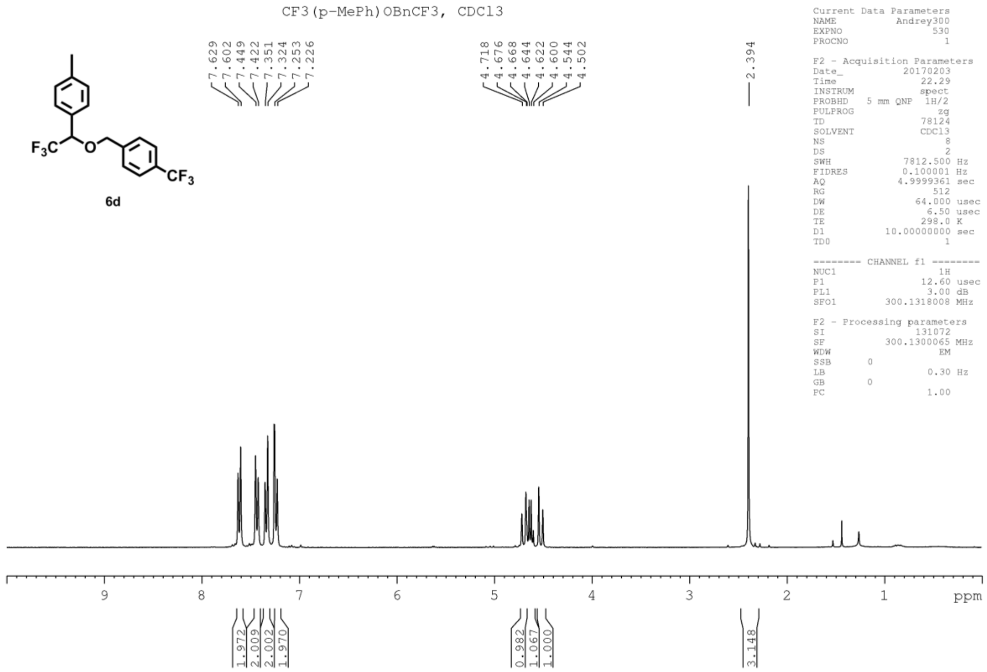

Supplementary figure 57. ^{1}H NMR spectra of **6d** in $CDCl_3$

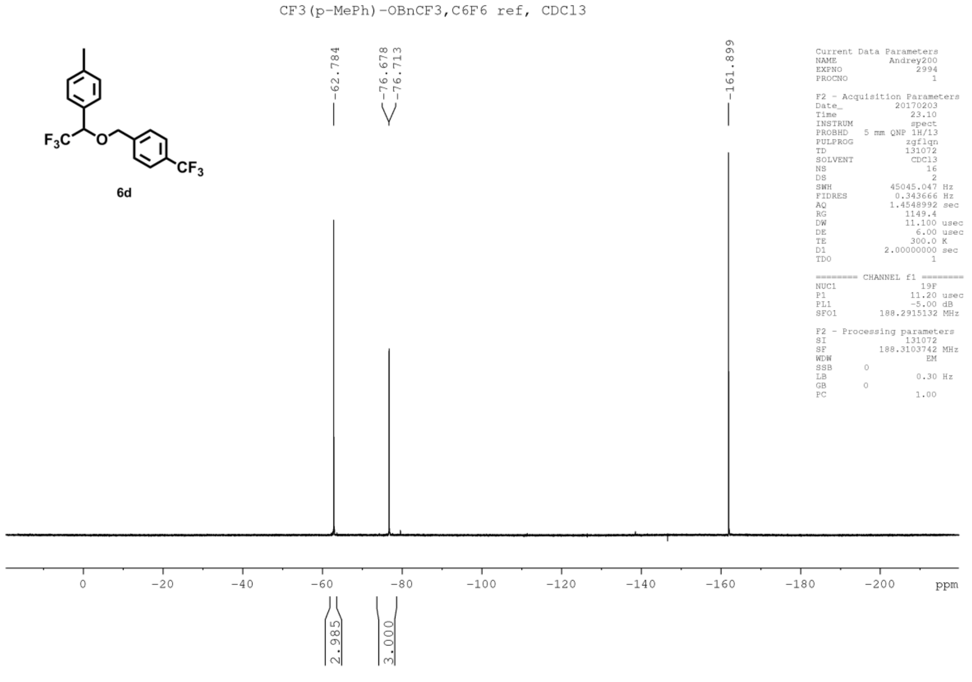

Supplementary figure 58. ^{19}F NMR spectra of **6d** in $CDCl_3$

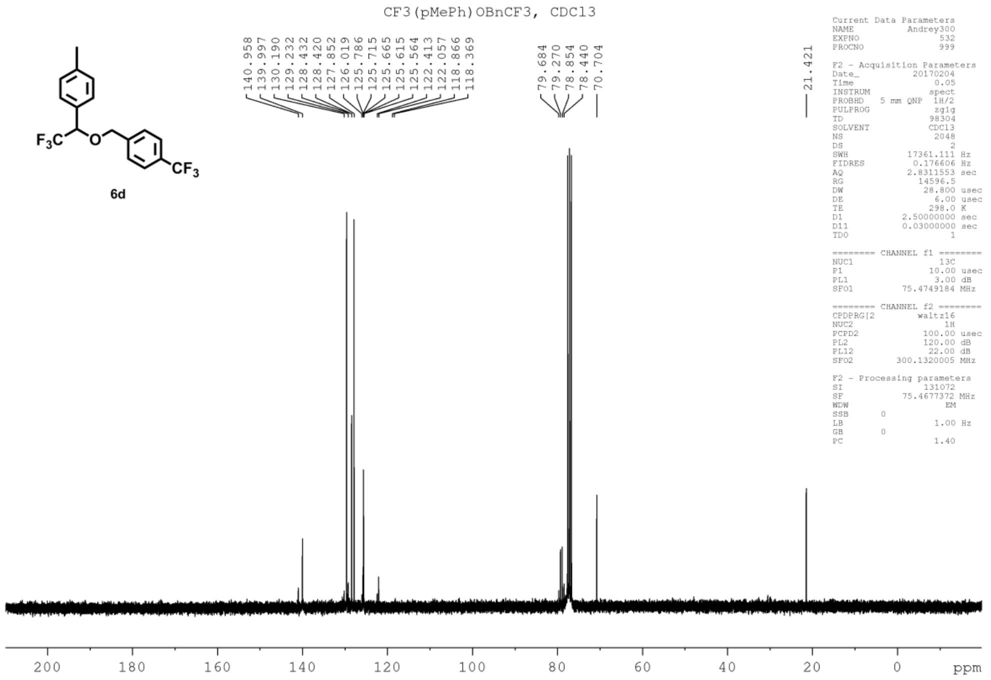

Supplementary figure 59. ^{13}C NMR spectra of **6d** in $CDCl_3$

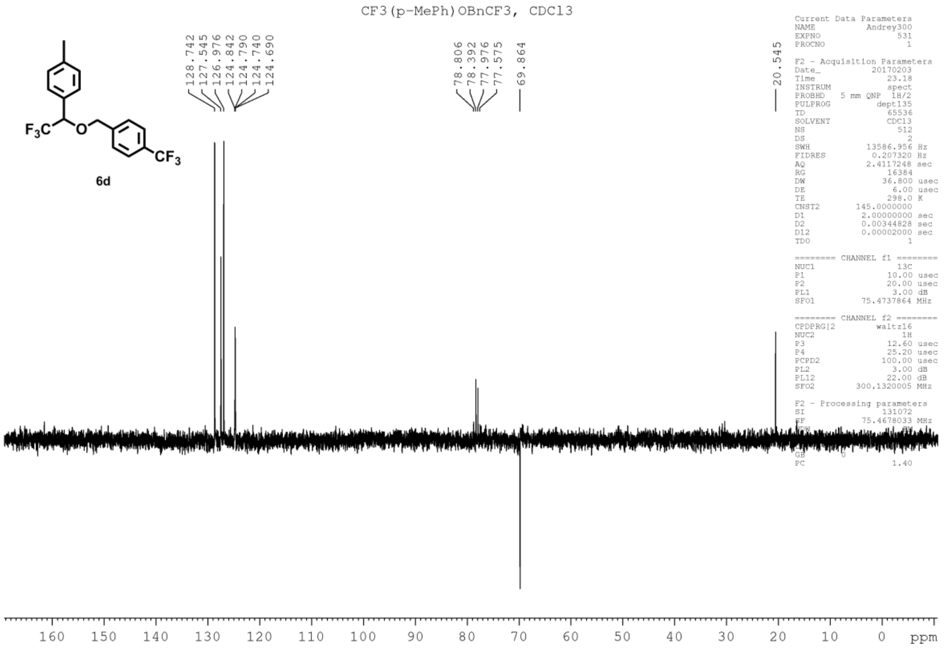

Supplementary figure 60. DEPT-135 NMR spectra of **6d** in $CDCl_3$

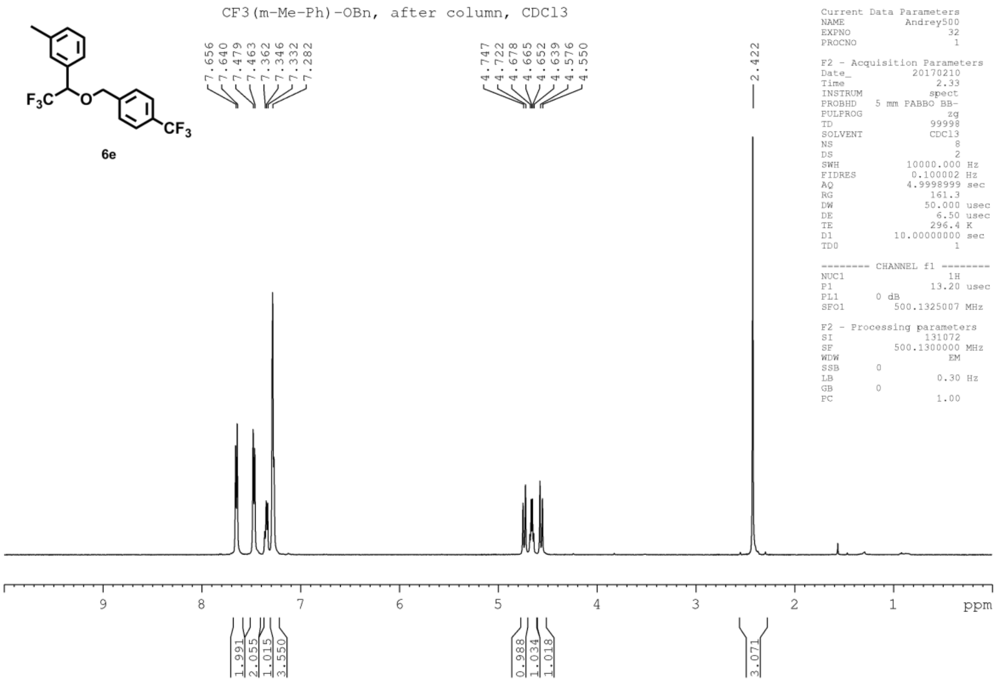

Supplementary figure 61. ^{1}H NMR spectra of **6e** in $CDCl_3$

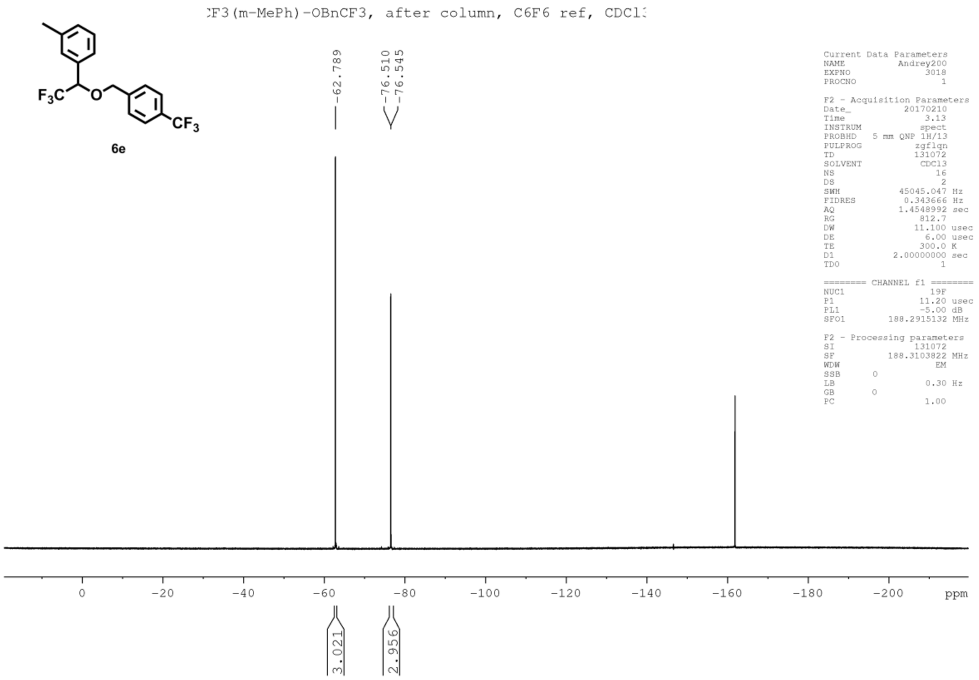

Supplementary figure 62. ^{19}F NMR spectra of **6e** in $CDCl_3$

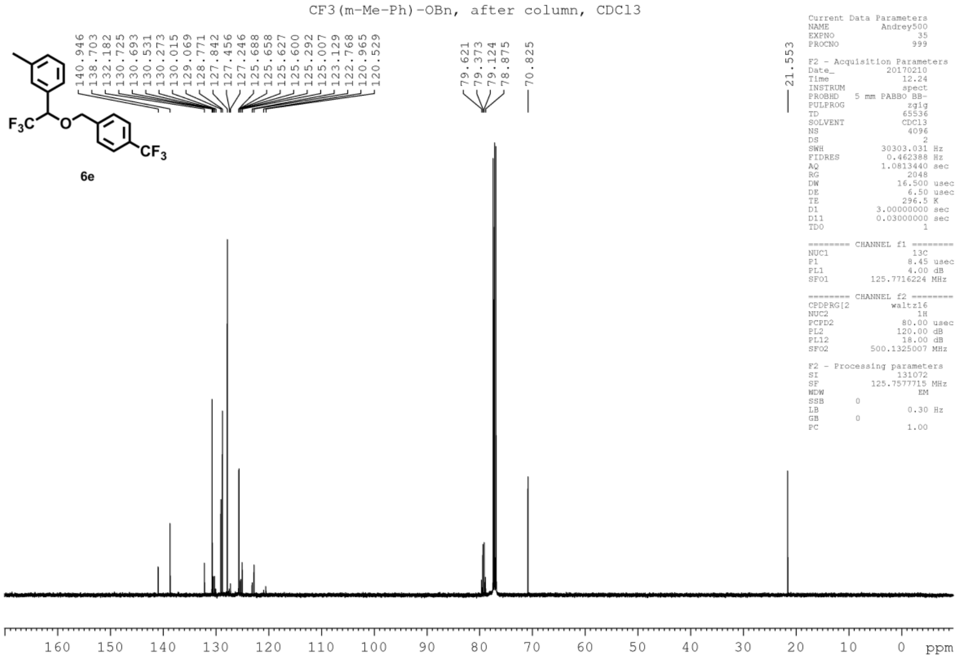

Supplementary figure 63. ^{13}C NMR spectra of **6e** in $CDCl_3$

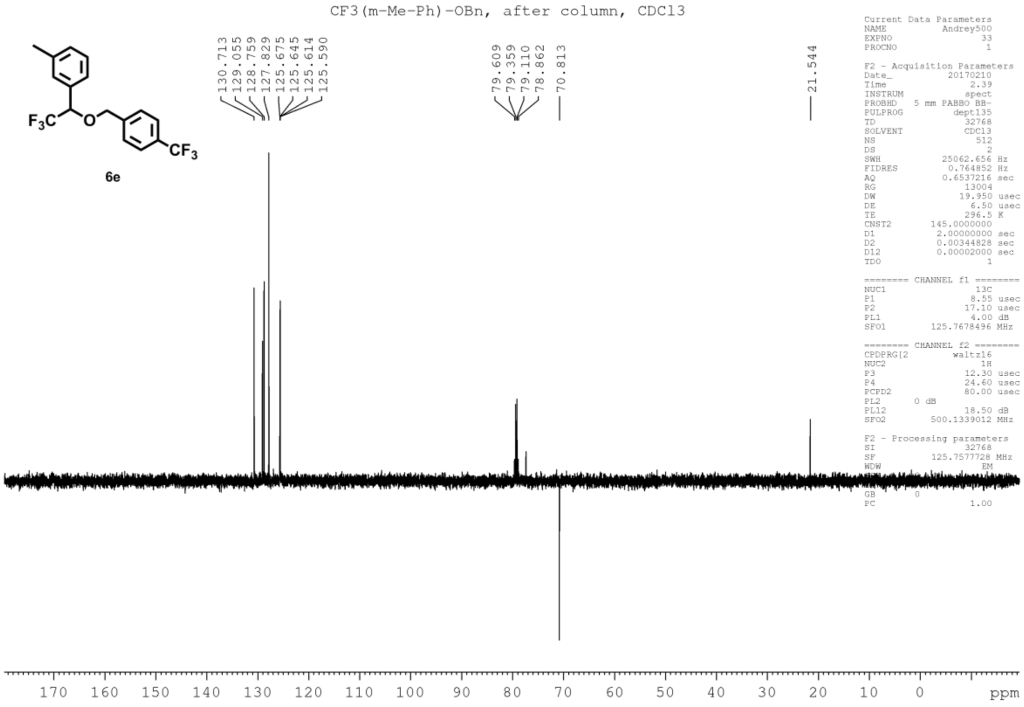

Supplementary figure 64. DEPT-135 NMR spectra of **6e** in $CDCl_3$

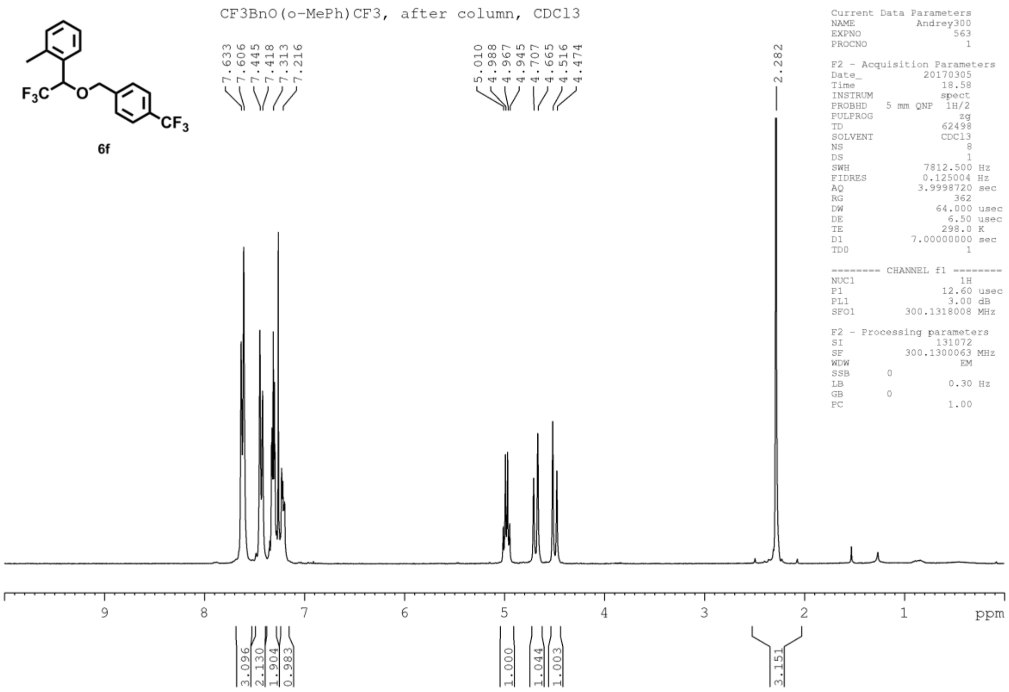

Supplementary figure 65. ^{1}H NMR spectra of **6f** in $CDCl_3$

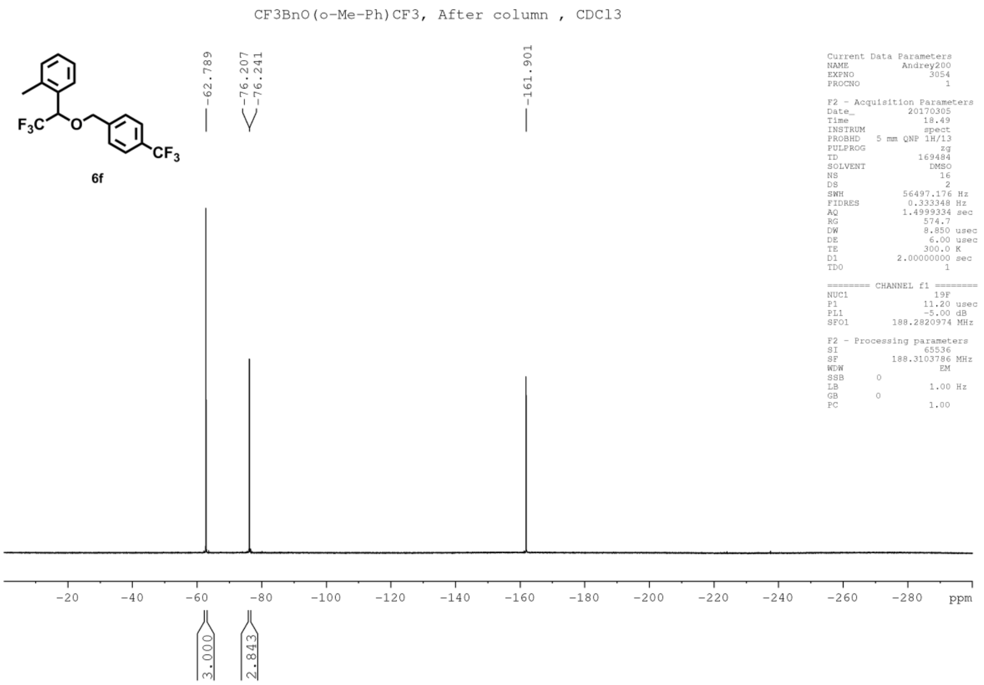

Supplementary figure 66. ^{19}F NMR spectra of **6f** in $CDCl_3$

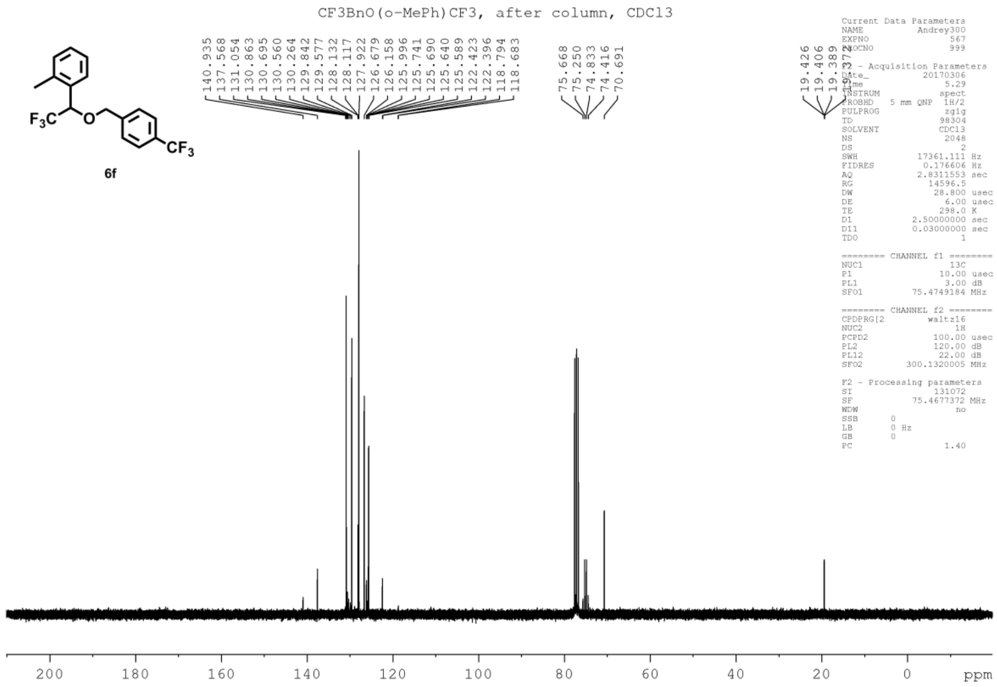

Supplementary figure 67. ^{13}C NMR spectra of **6f** in $CDCl_3$

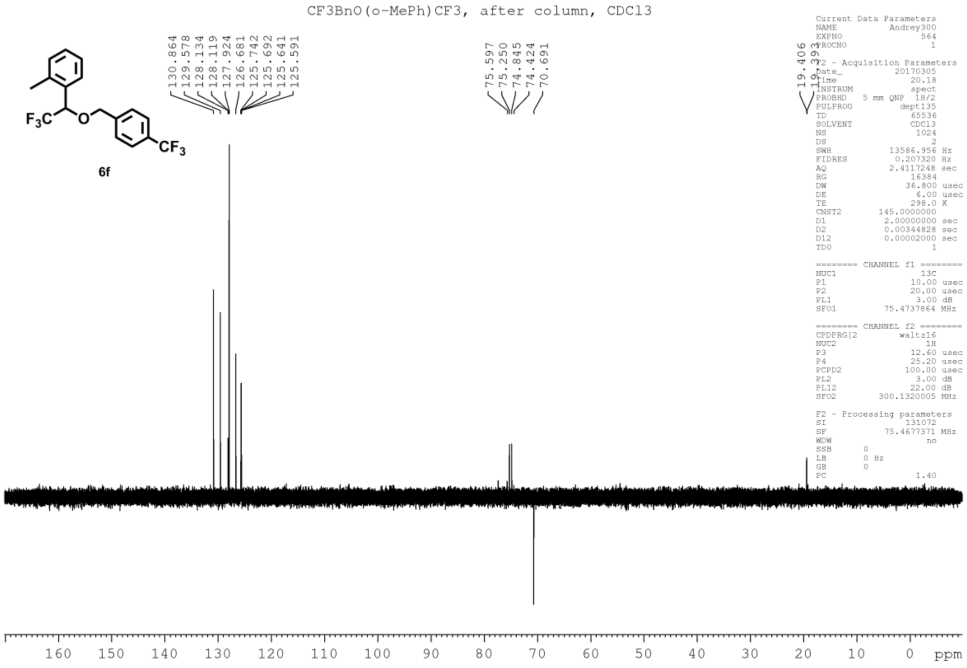

Supplementary figure 68. DEPT-135 NMR spectra of **6f** in $CDCl_3$

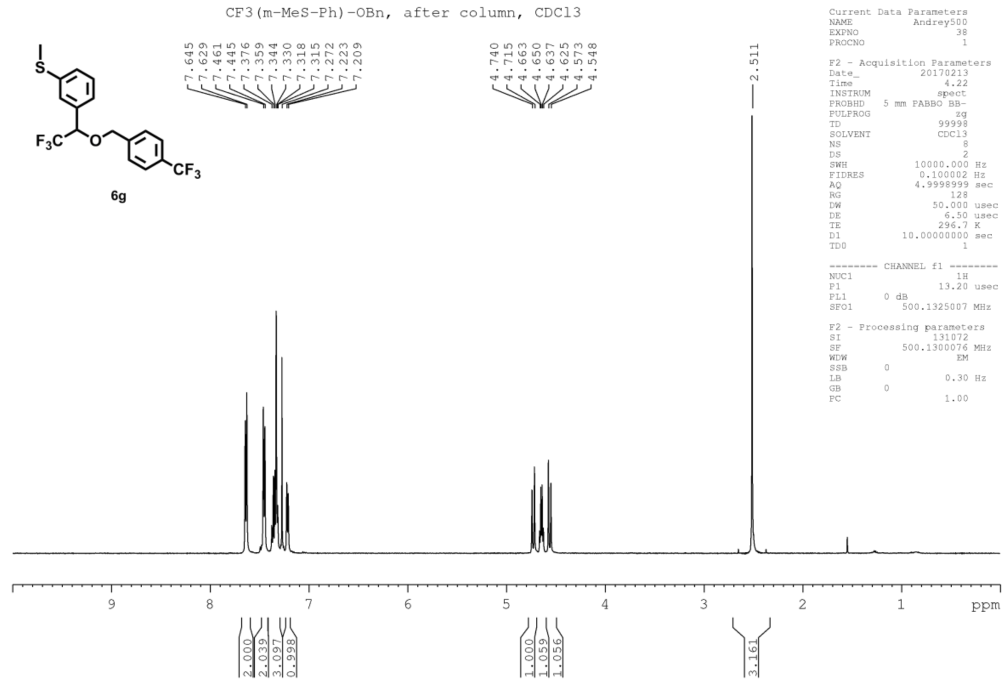

Supplementary figure 69. ^{1}H NMR spectra of **6g** in $CDCl_3$

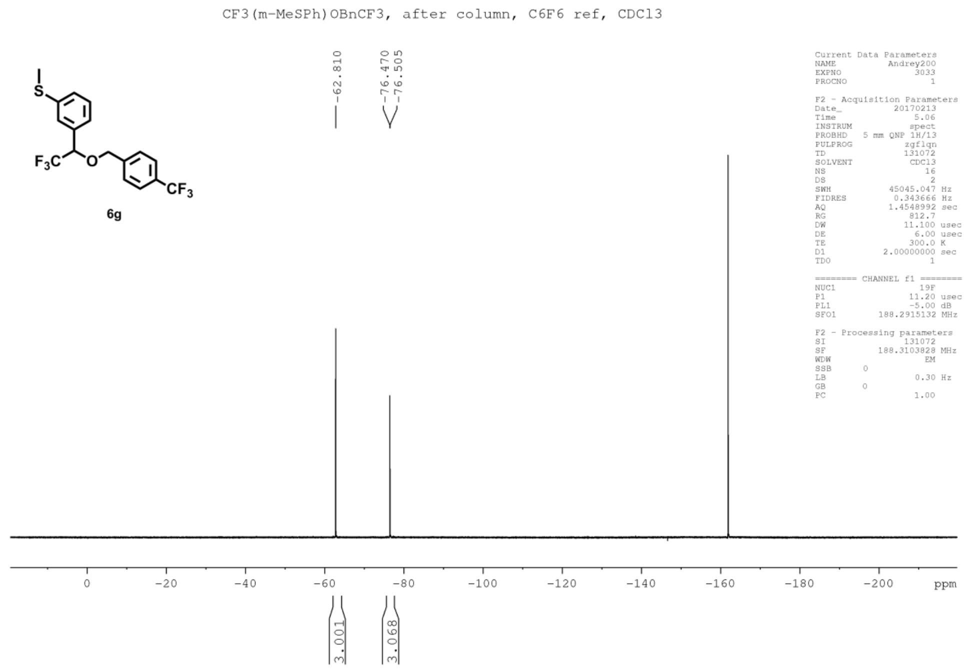

Supplementary figure 70. ^{19}F NMR spectra of **6g** in $CDCl_3$

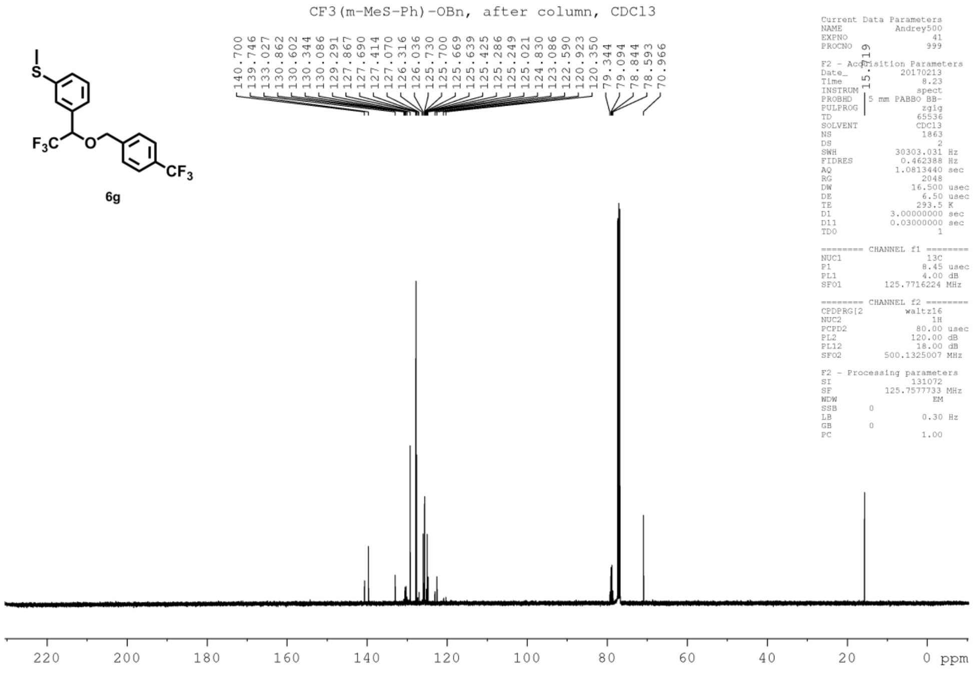

Supplementary figure 71. ^{13}C NMR spectra of **6**g in $CDCl_3$

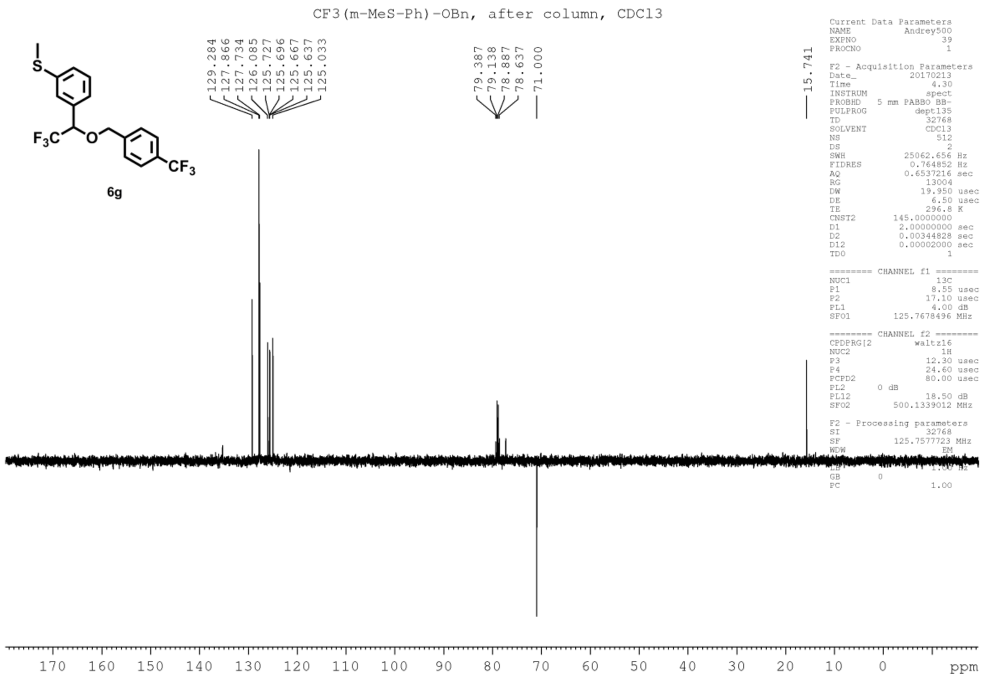

Supplementary figure 72.DEPT-135 NMR spectra of **6g** in $CDCl_3$

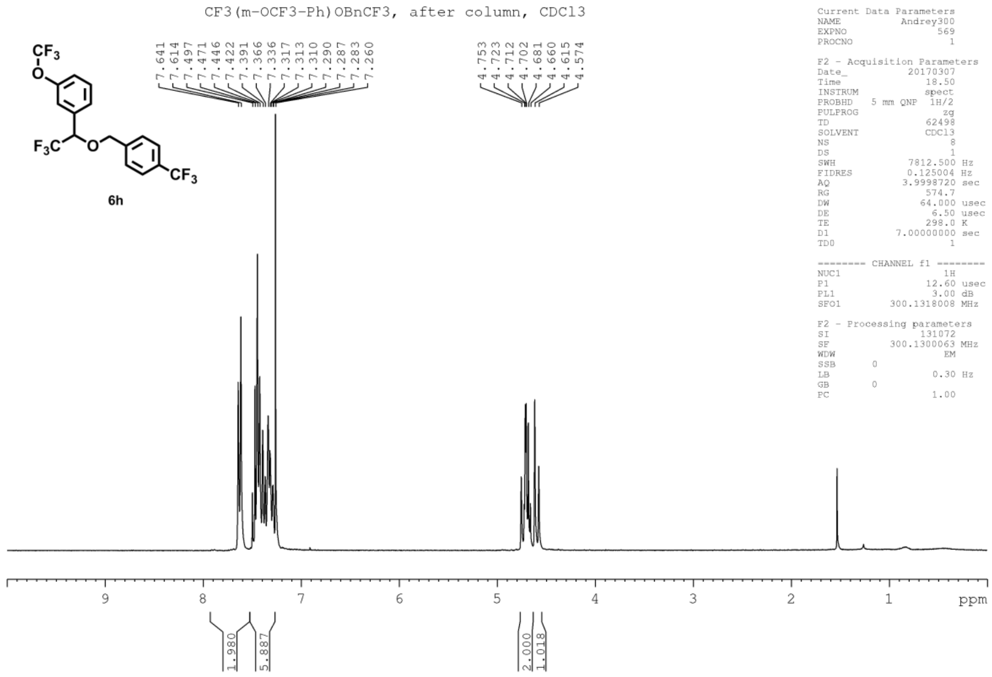

Supplementary figure 73. ^{1}H NMR spectra of **6h** in $CDCl_3$

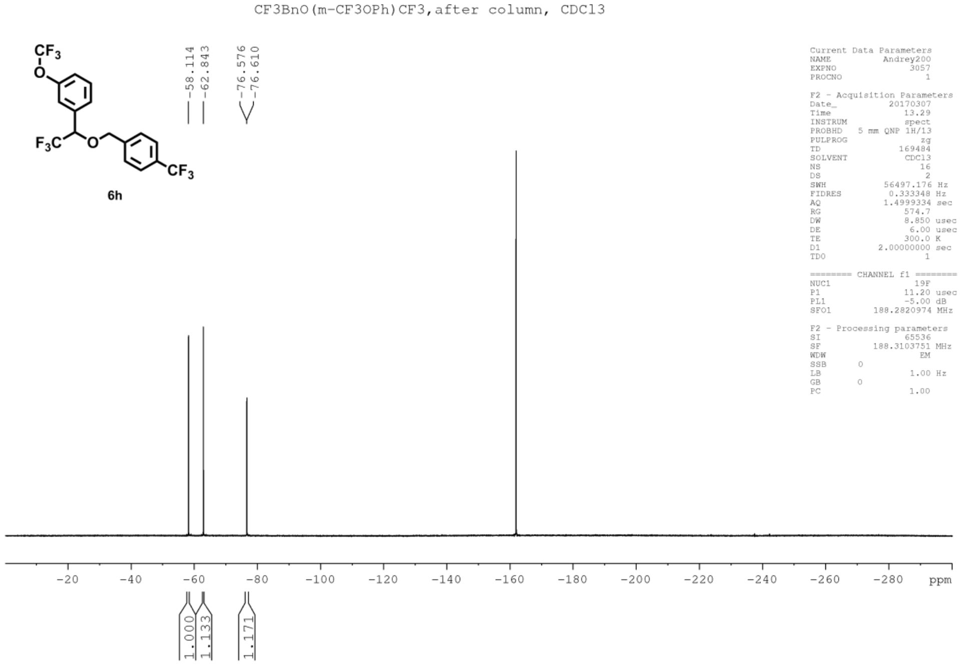

Supplementary figure 74. ^{19}F NMR spectra of **6h** in $CDCl_3$

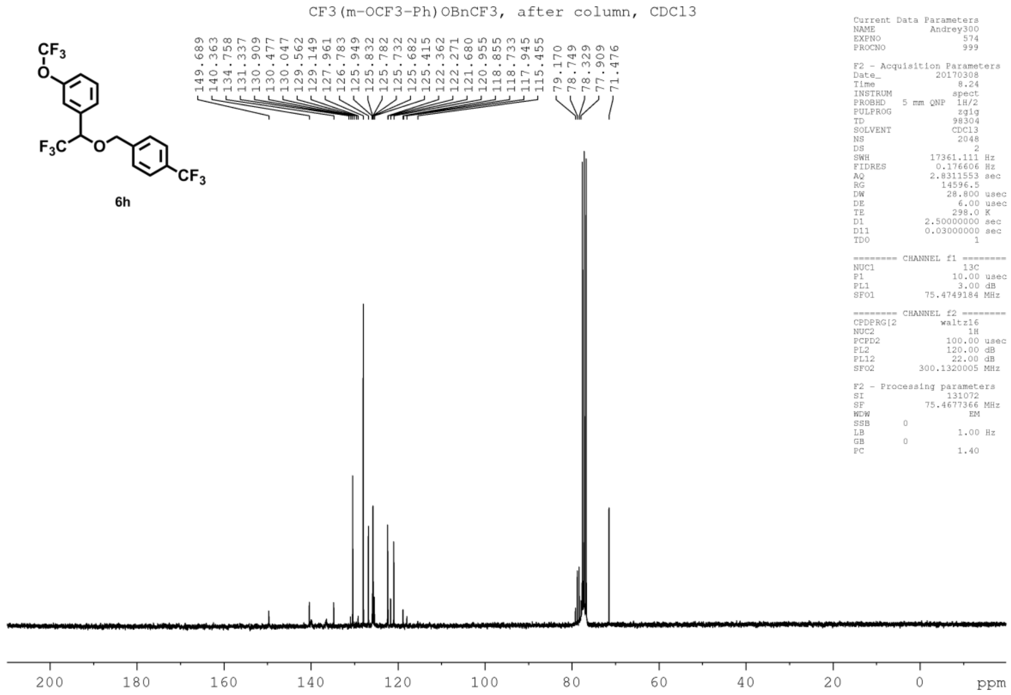

Supplementary figure 75. ^{13}C NMR spectra of **6h** in $CDCl_3$

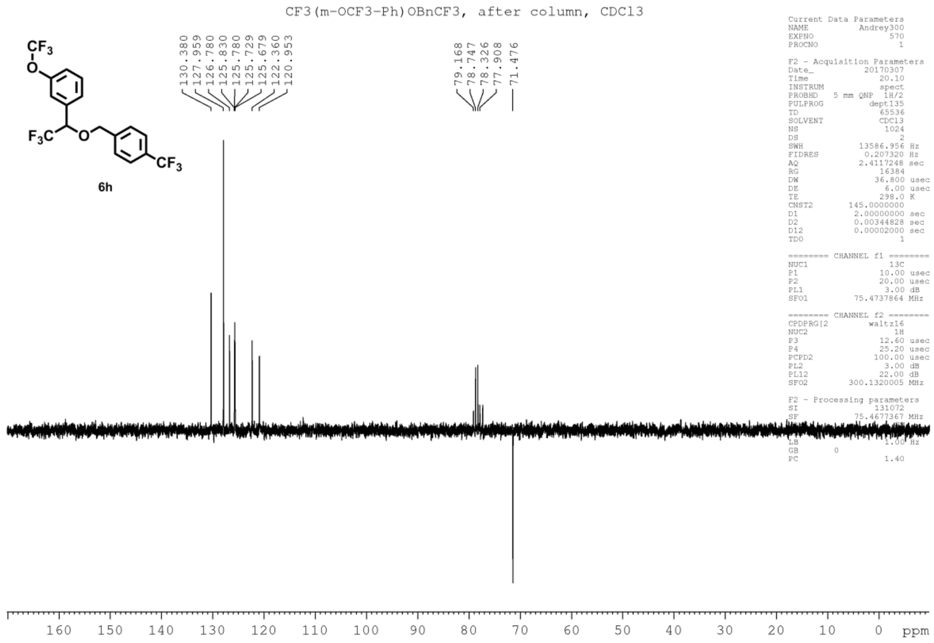

Supplementary figure 76. DEPT-135 NMR spectra of **6h** in $CDCl_3$

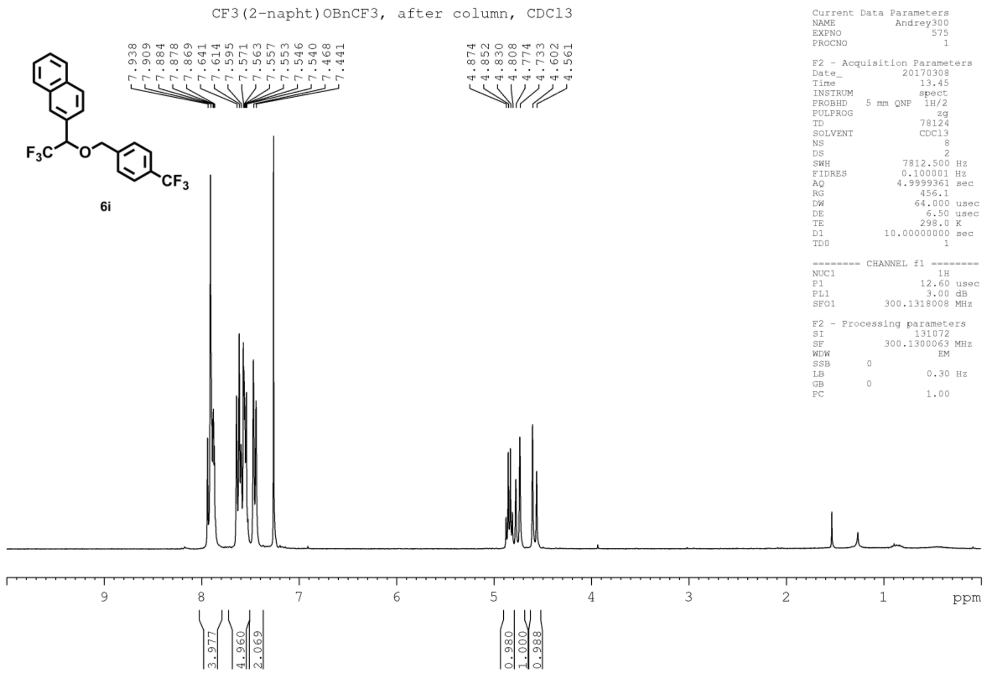

Supplementary figure 77. ^{1}H NMR spectra of **6i** in $CDCl_3$

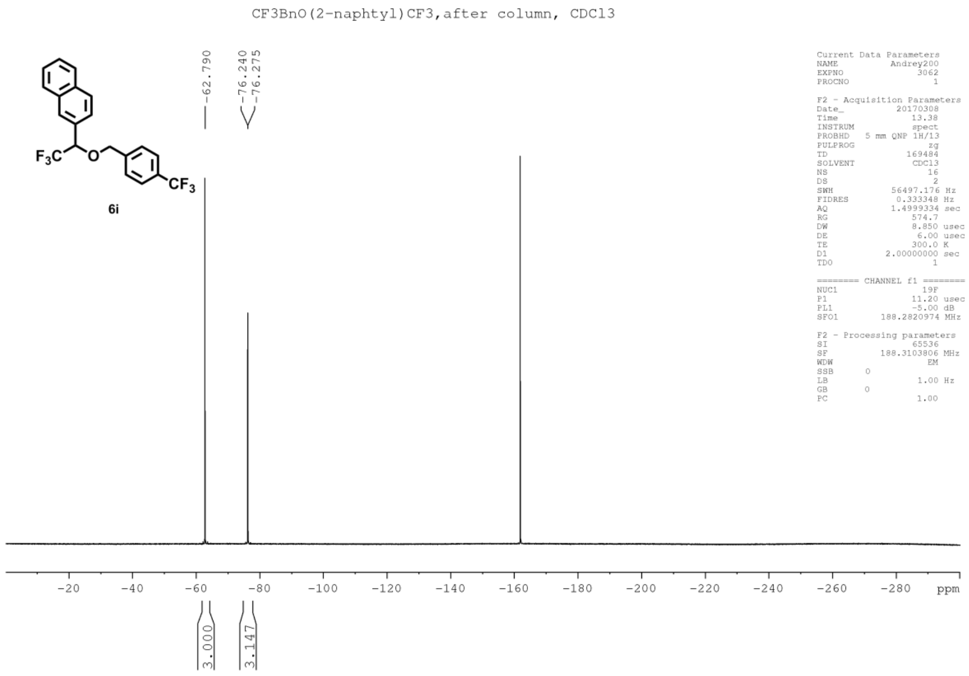

Supplementary figure 78. ^{19}F NMR spectra of **6i** in $CDCl_3$

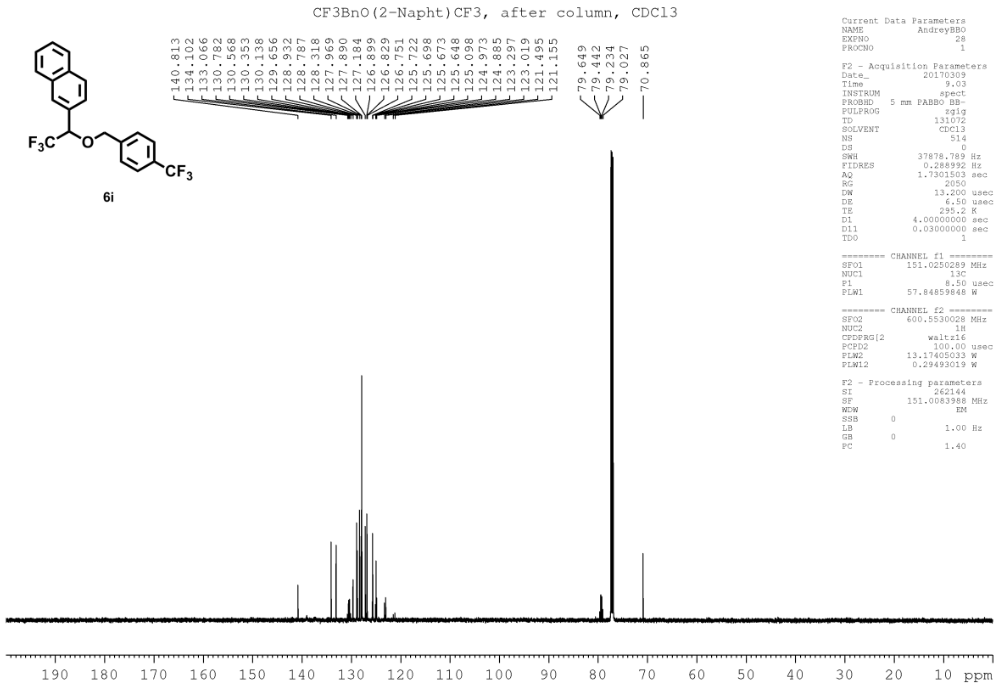

Supplementary figure 79. ^{13}C NMR spectra of **6i** in $CDCl_3$

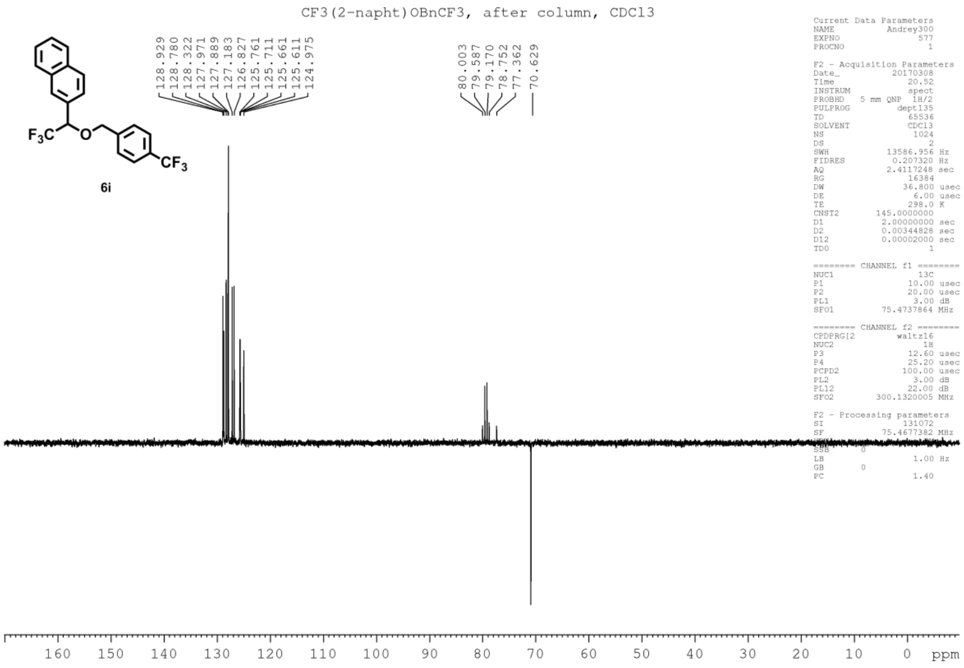

Supplementary figure 80. DEPT-135 NMR spectra of **6i** in $CDCl_3$

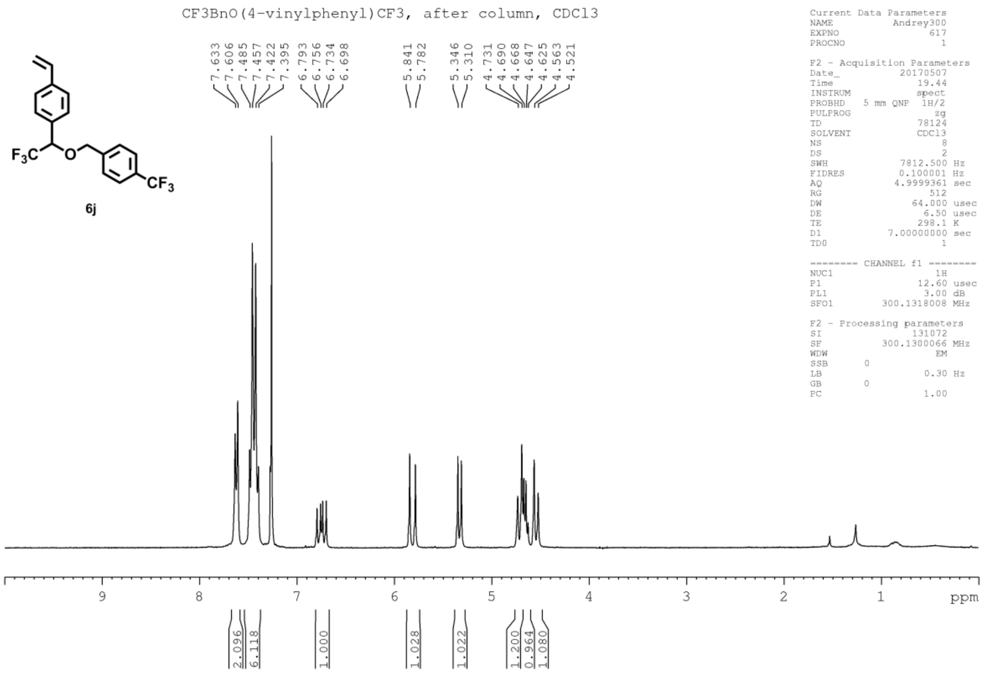

Supplementary figure 81. ^{1}H NMR spectra of **6j** in $CDCl_3$

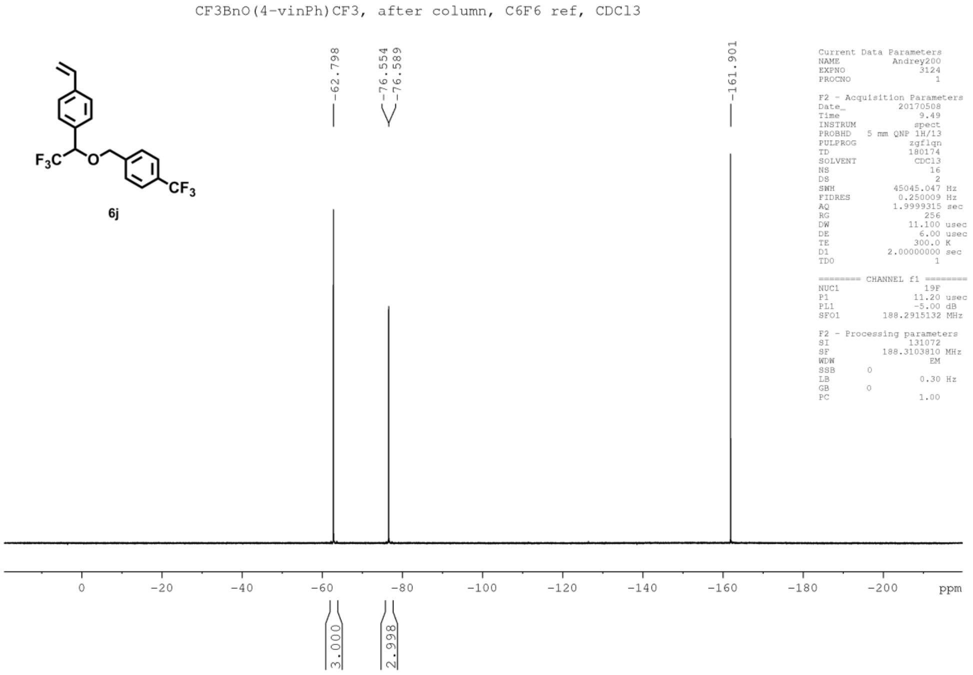

Supplementary figure 82. ^{19}F NMR spectra of **6j** in $CDCl_3$

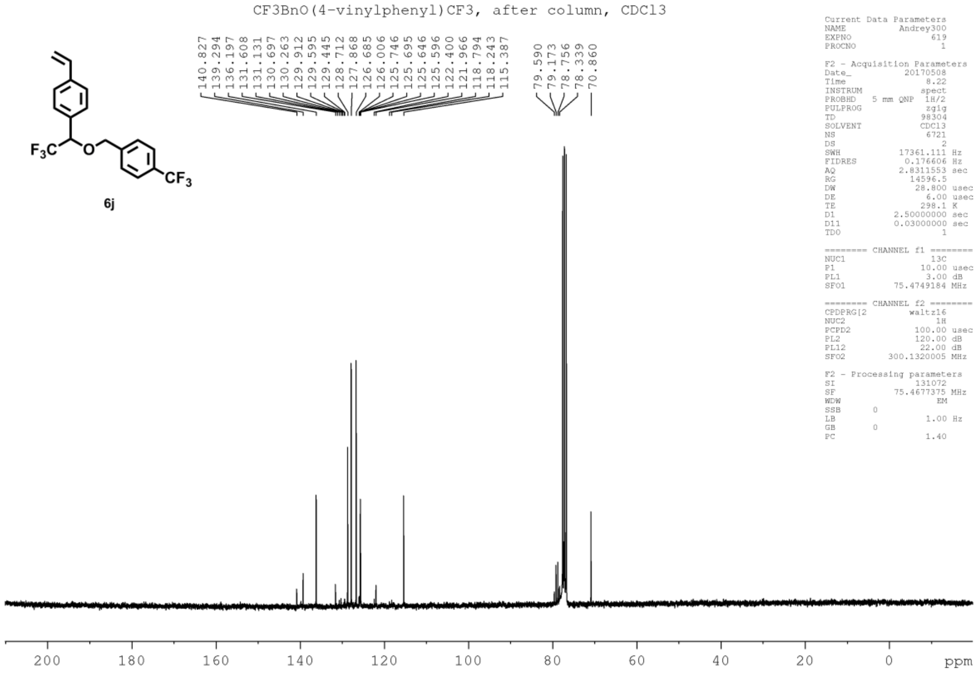

Supplementary figure 83. ^{13}C NMR spectra of **6j** in $CDCl_3$

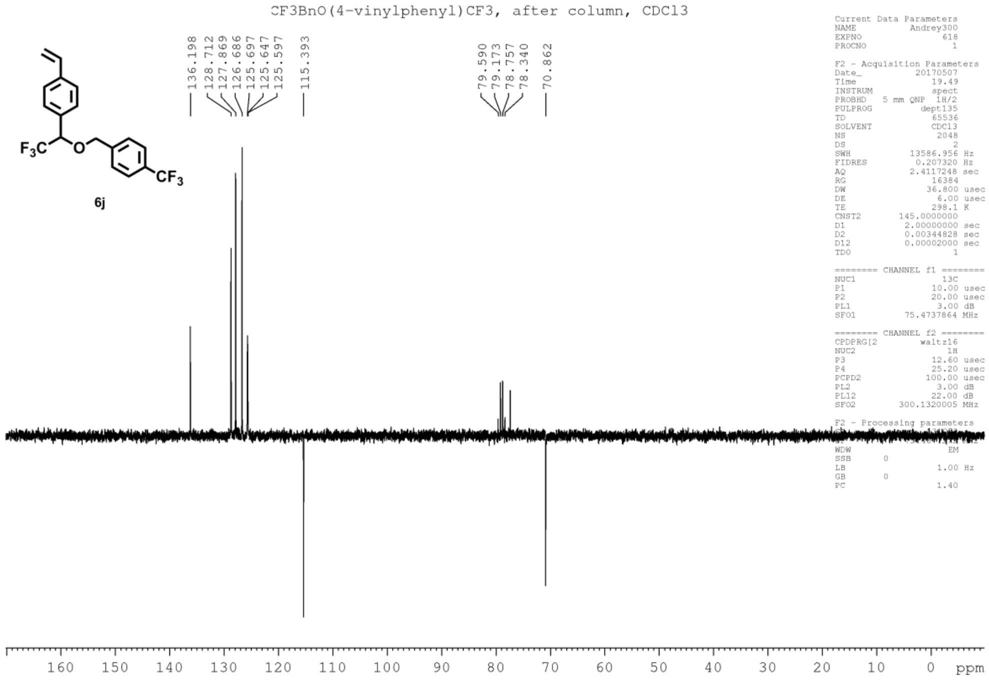

Supplementary figure 84. DEPT-135 NMR spectra of **6j** in $CDCl_3$

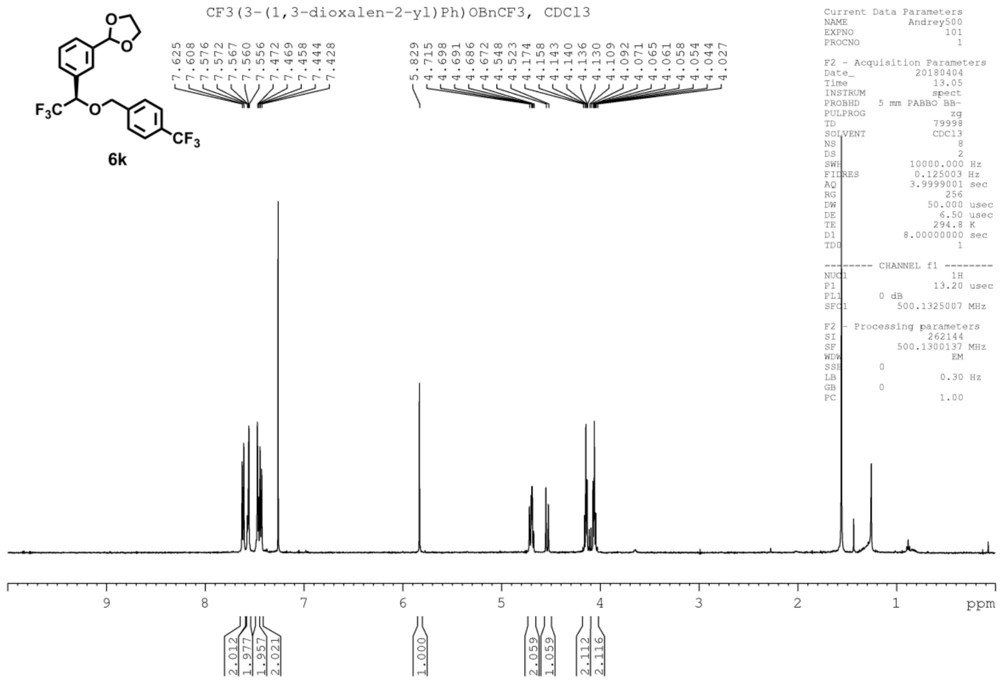

Supplementary figure 85. ^{1}H NMR spectra of **6k** in $CDCl_3$

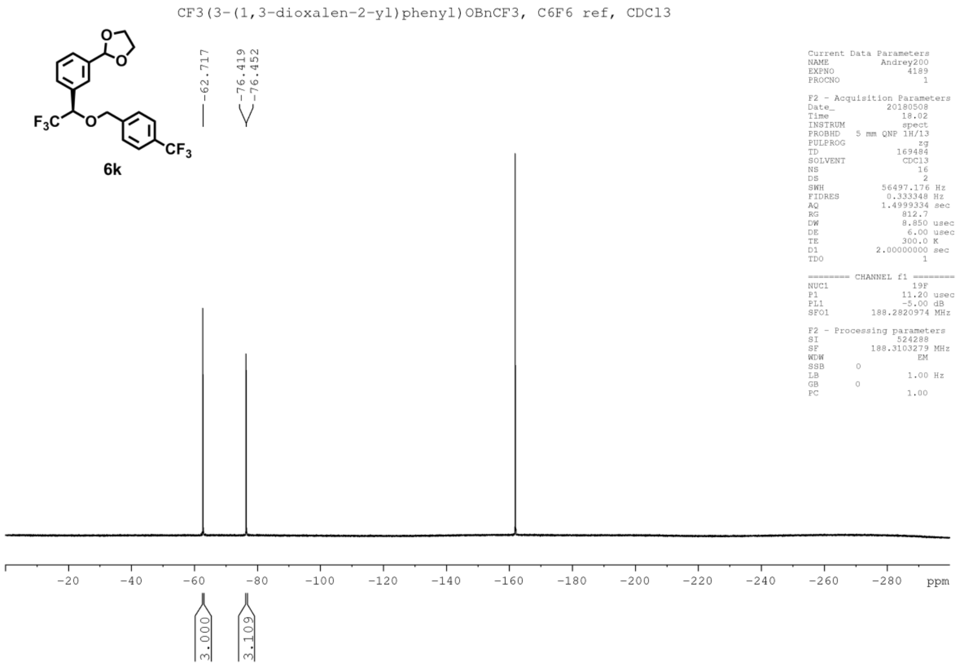

Supplementary figure 86. ^{19}F NMR spectra of **6k** in $CDCl_3$

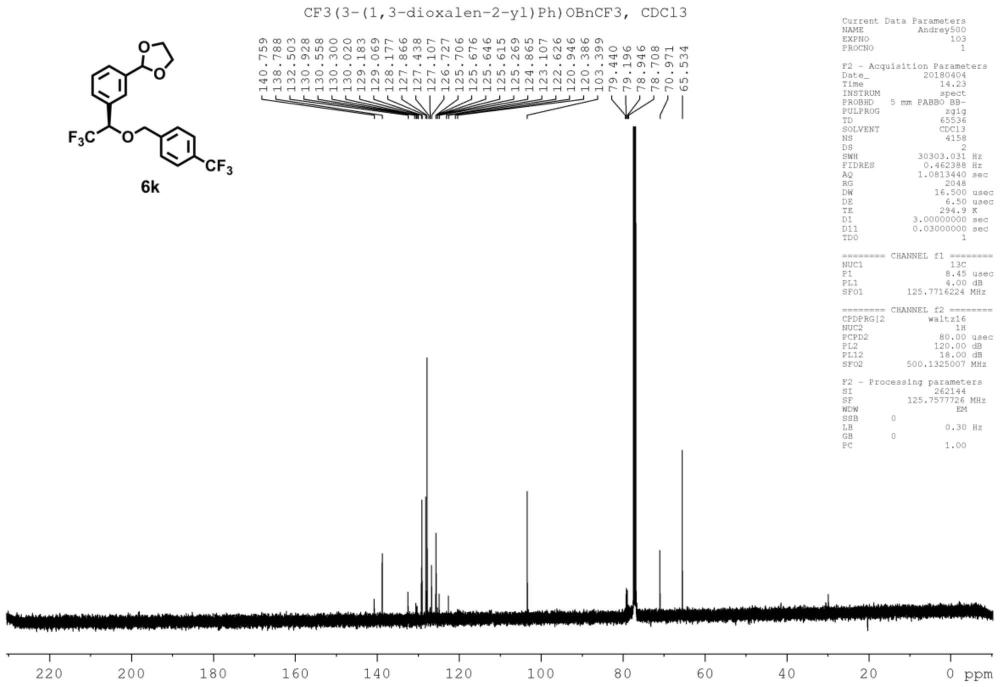

Supplementary figure 87. ^{13}C NMR spectra of **6k** in $CDCl_3$

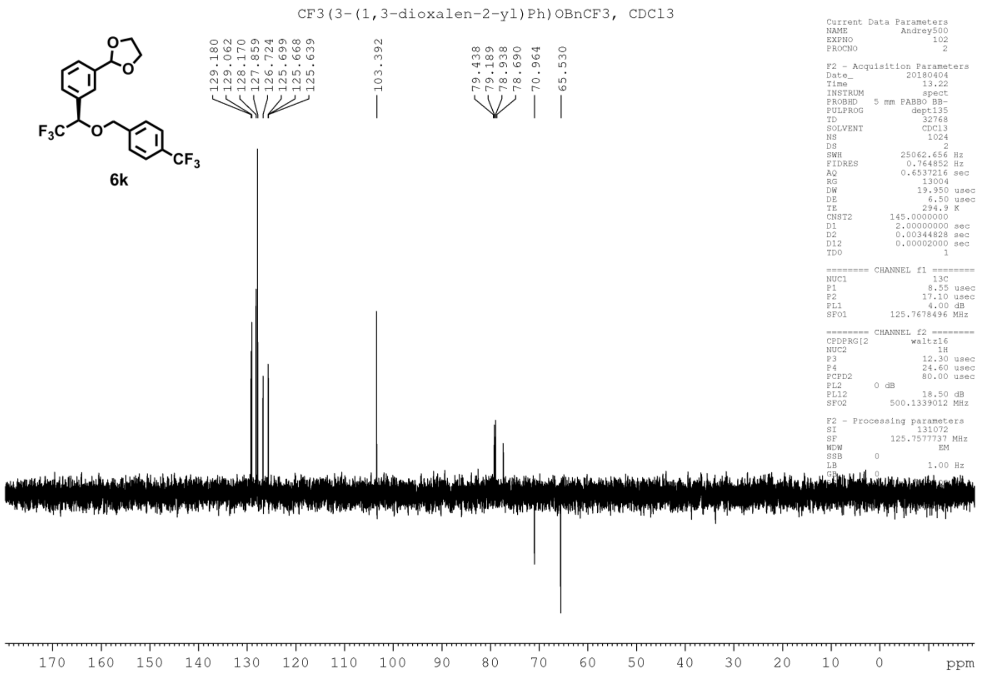

Supplementary figure 88. DPET-135 NMR spectra of **6k** in $CDCl_3$

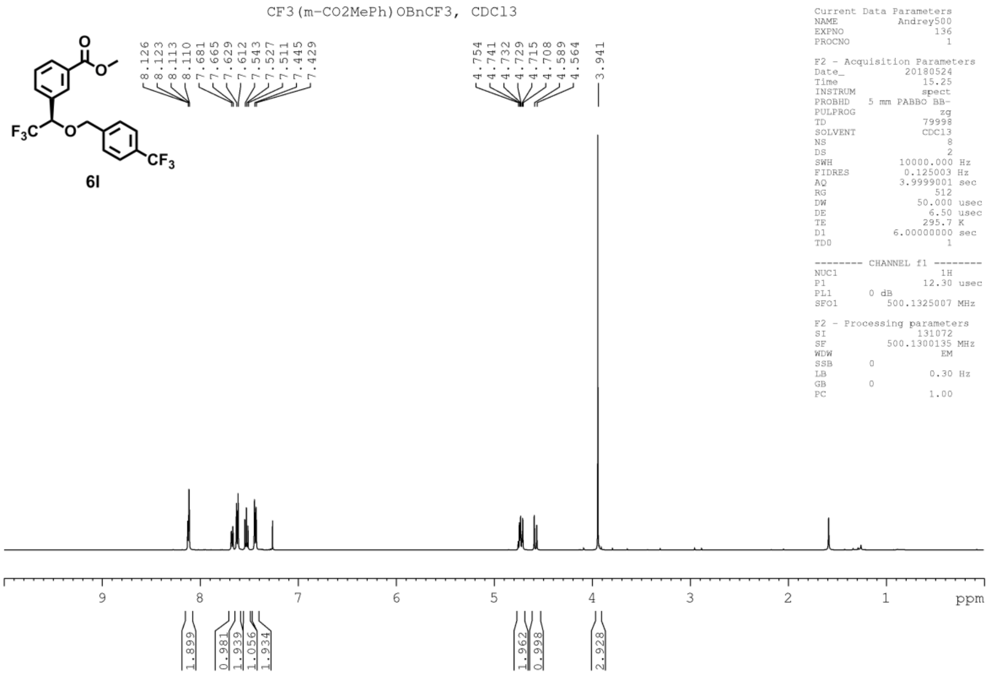

Supplementary figure 89. ^{1}H NMR spectra of **6l** in $CDCl_3$

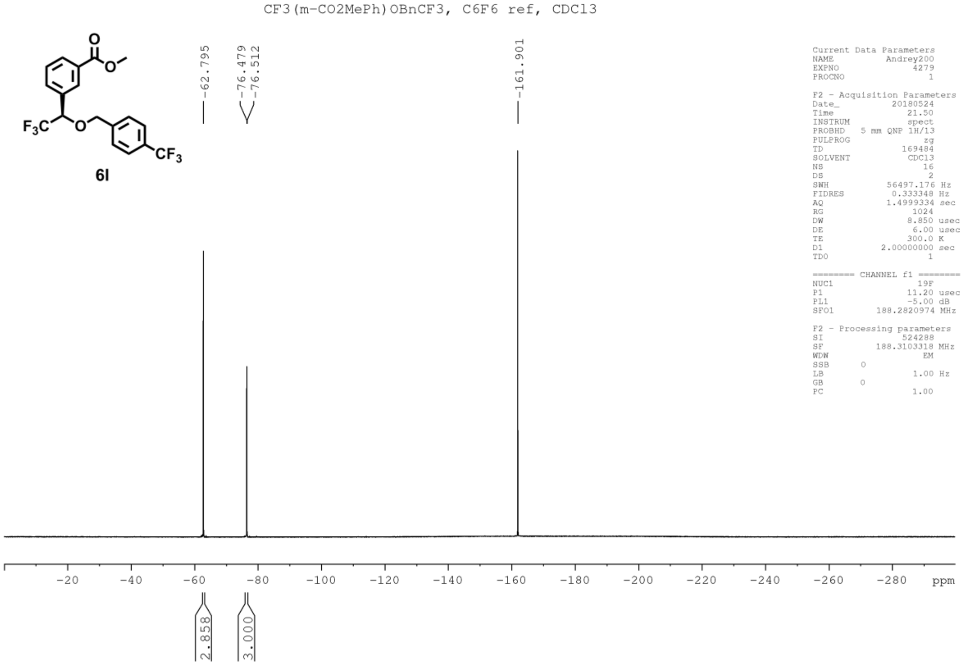

Supplementary figure 90. ^{19}F NMR spectra of **6l** in $CDCl_3$

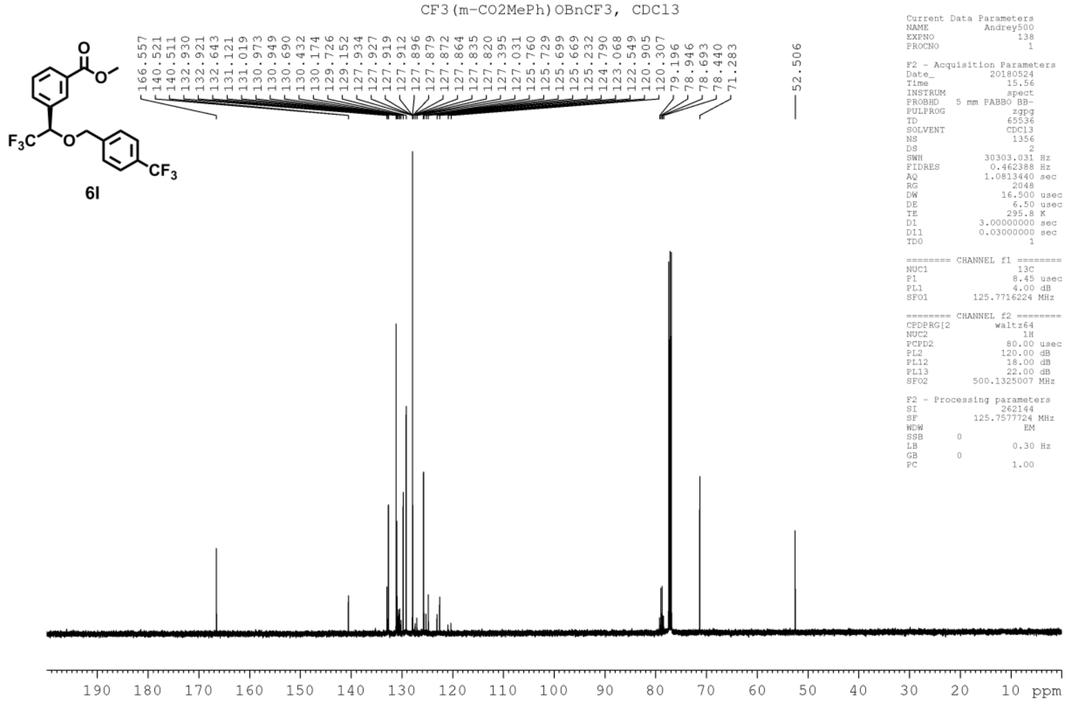

Supplementary figure 91. ^{13}C NMR spectra of **6l** in $CDCl_3$

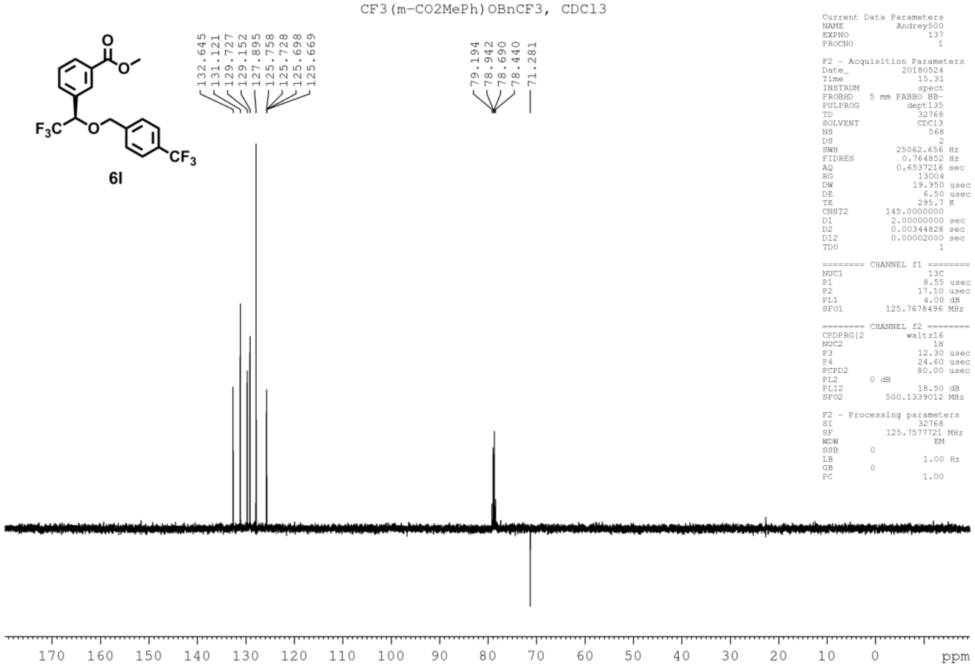

Supplementary figure 92. DEPT-135 NMR spectra of **6l** in $CDCl_3$

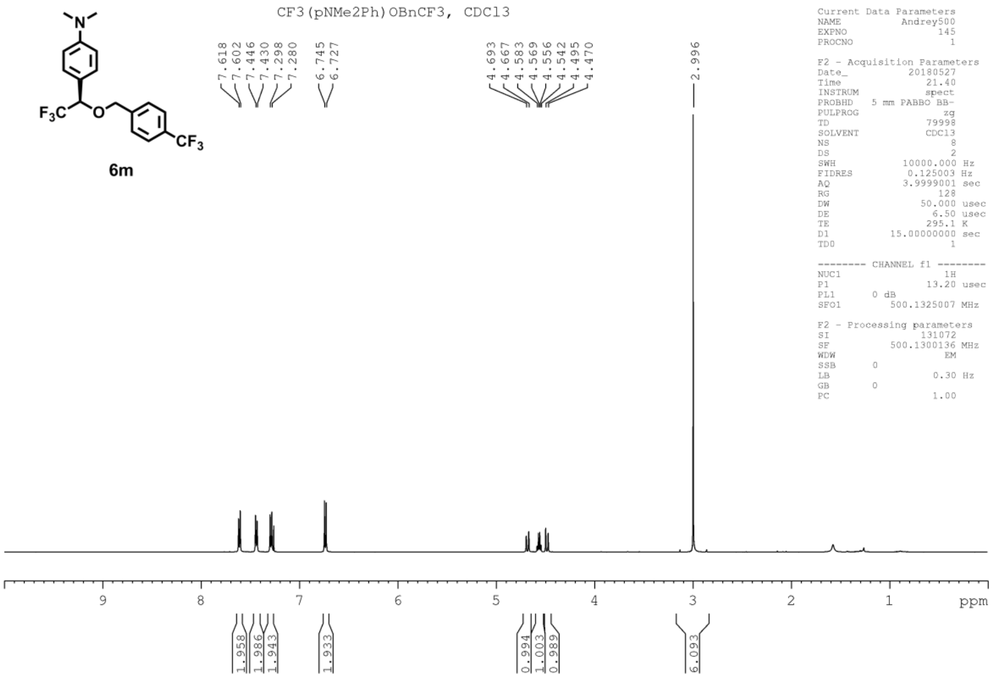

Supplementary figure 93. ^{1}H NMR spectra of **6m** in $CDCl_3$

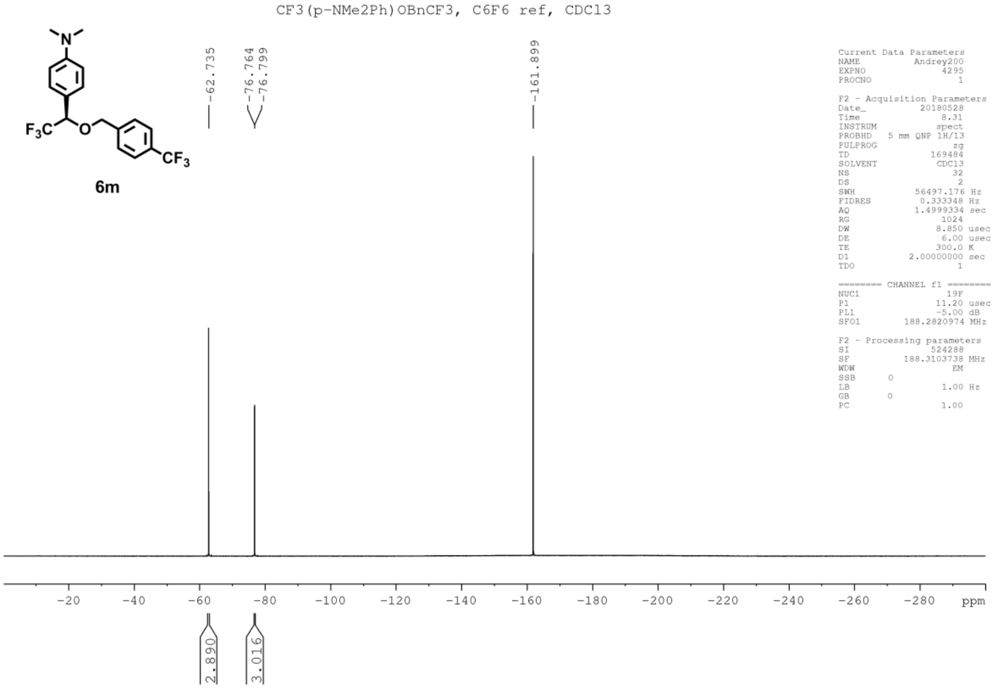

Supplementary figure 94. ^{19}F NMR spectra of **6m** in $CDCl_3$

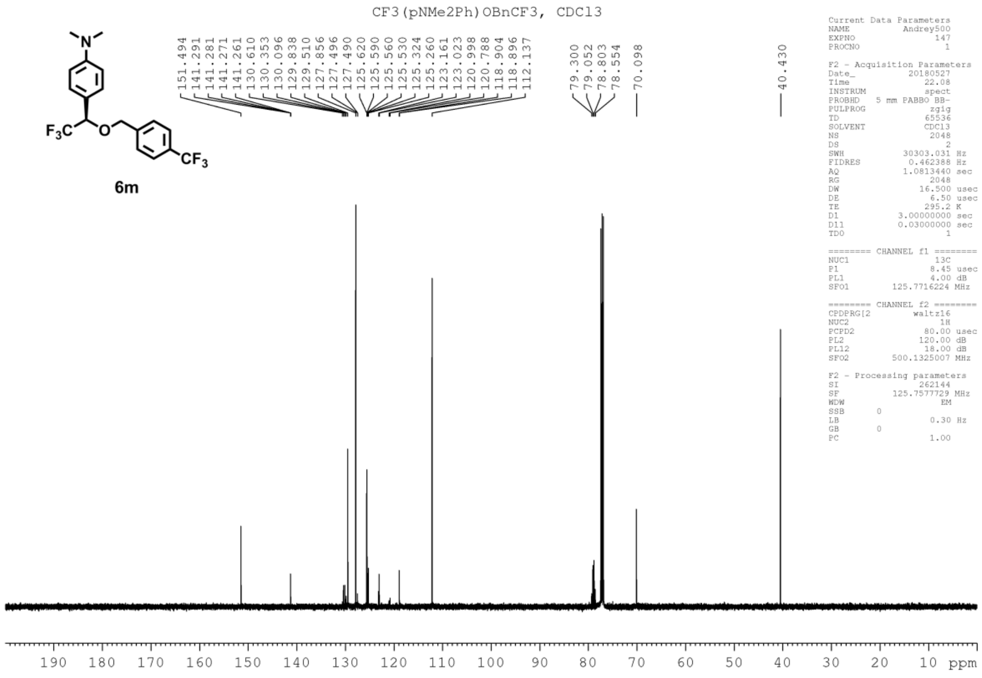

Supplementary figure 95. ^{13}C NMR spectra of **6m** in $CDCl_3$

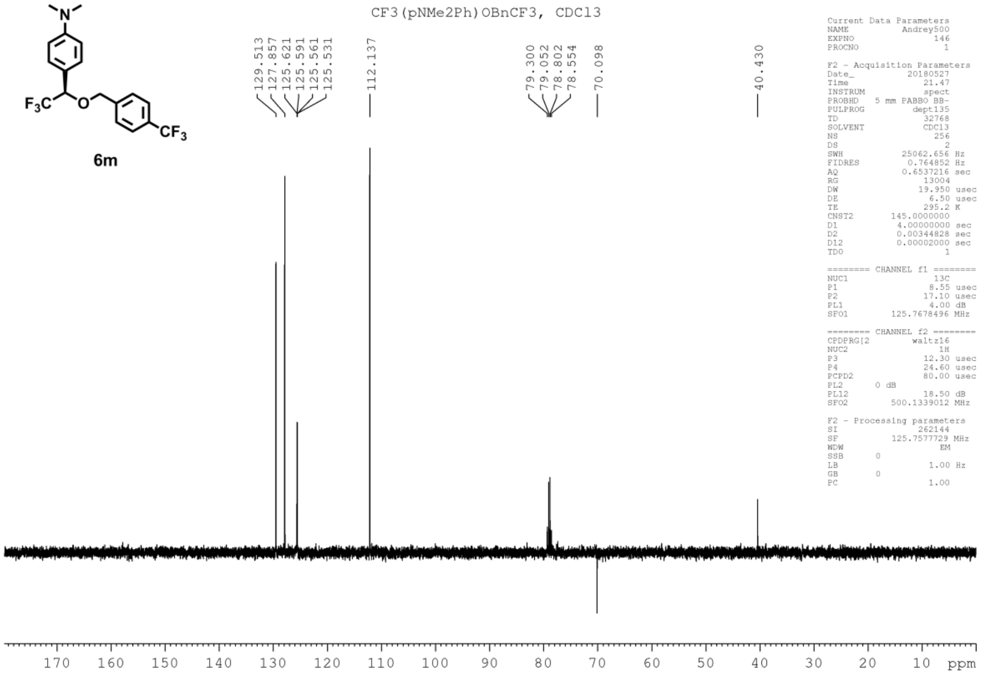

Supplementary figure 96. DEPT-135 NMR spectra of **6m** in $CDCl_3$

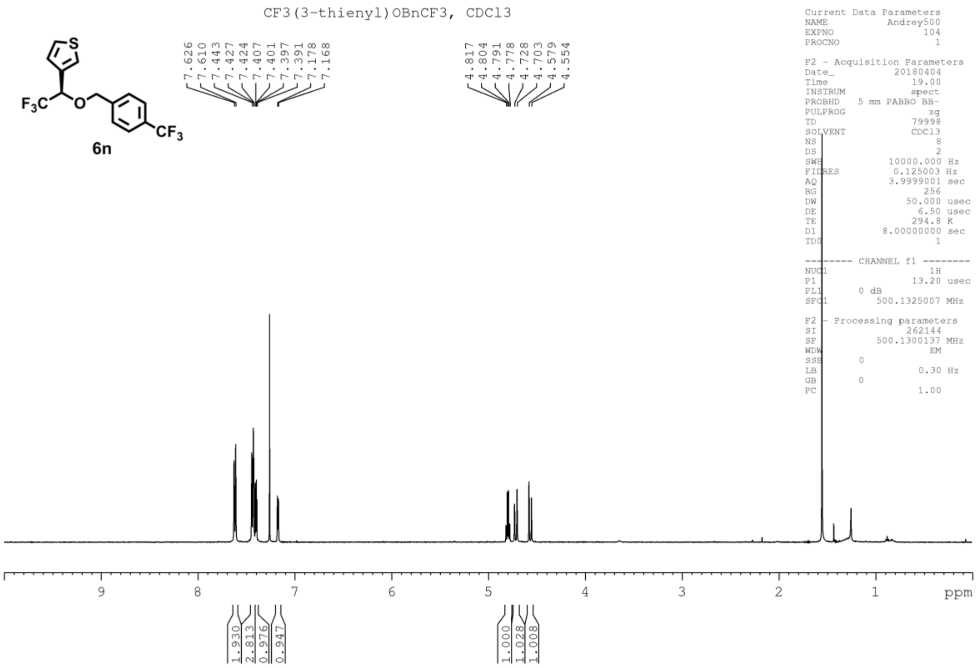

Supplementary figure 97. ^{1}H NMR spectra of **6n** in $CDCl_3$

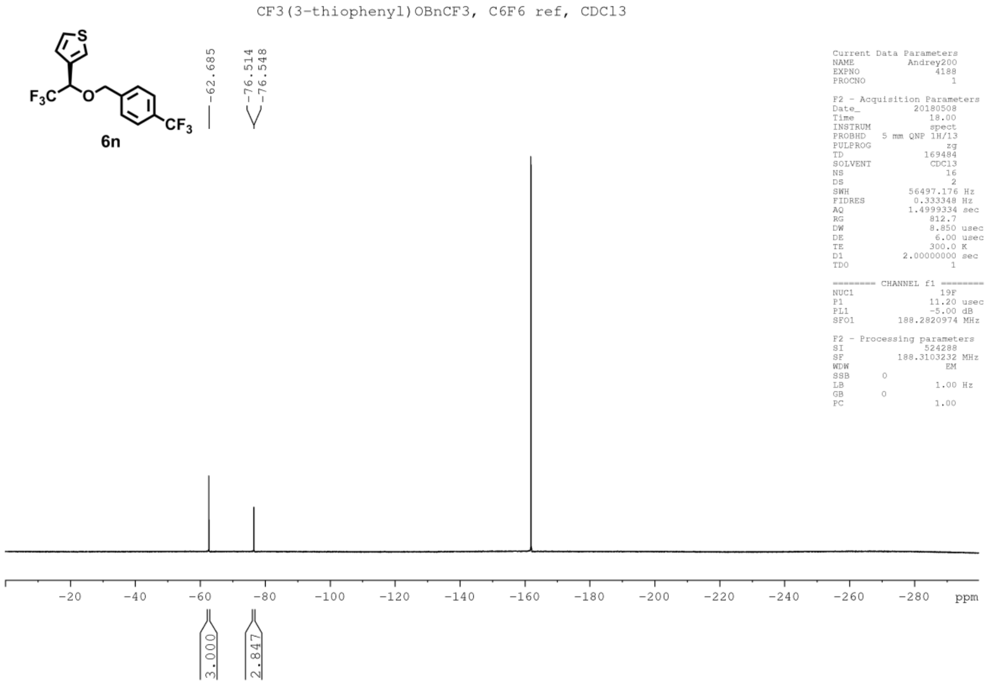

Supplementary figure 98. ^{19}F NMR spectra of **6n** in $CDCl_3$

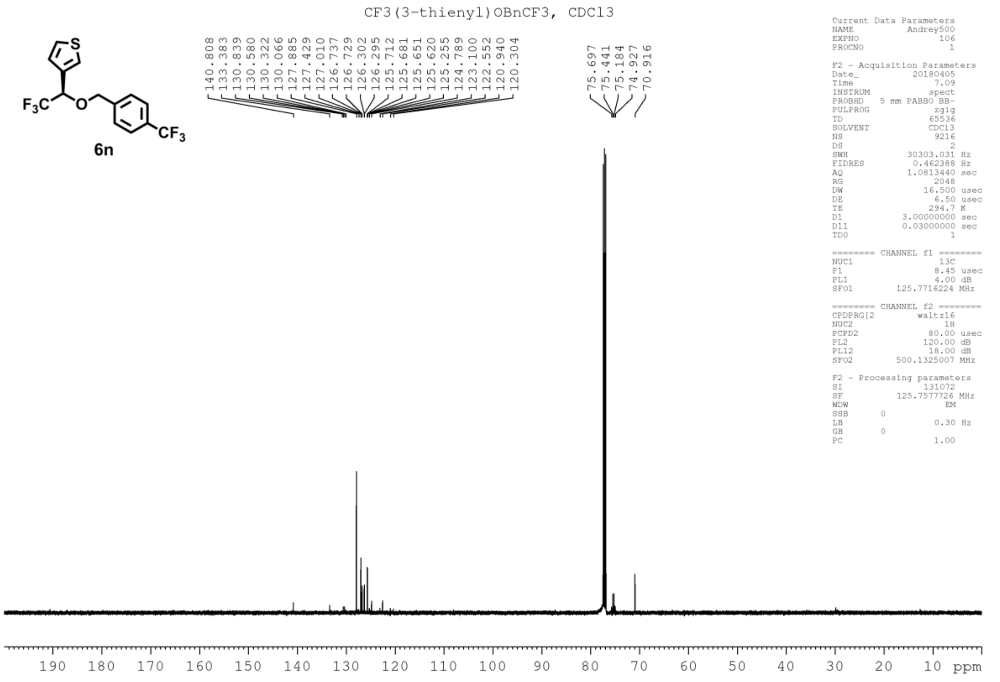

Supplementary figure 99. ^{13}C NMR spectra of **6n** in $CDCl_3$

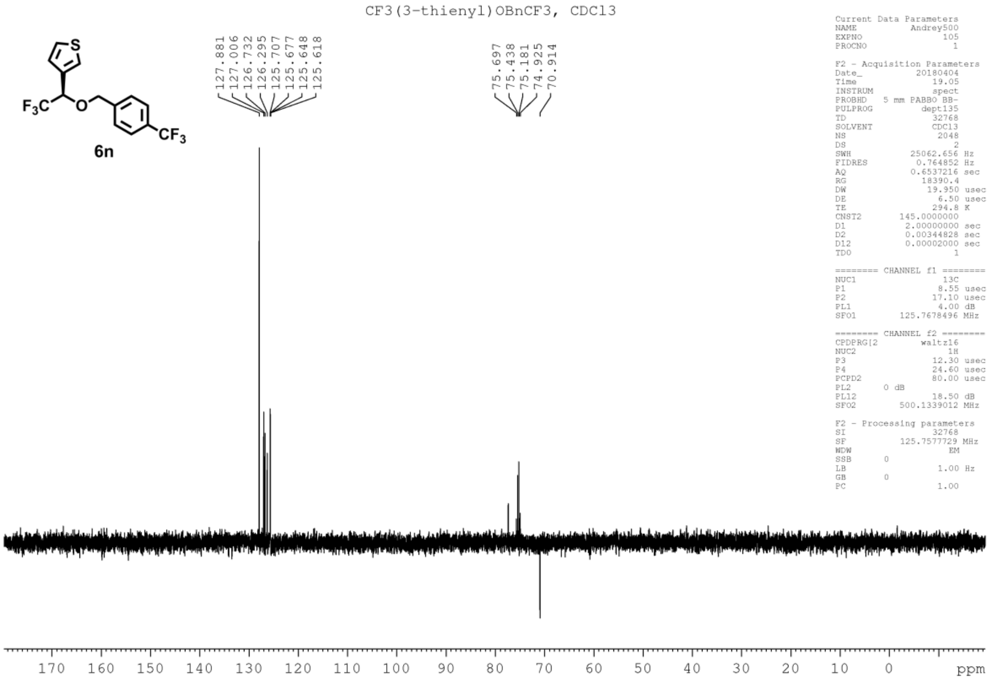

Supplementary figure 100. ^{1}H NMR spectra of **6n** in $CDCl_3$

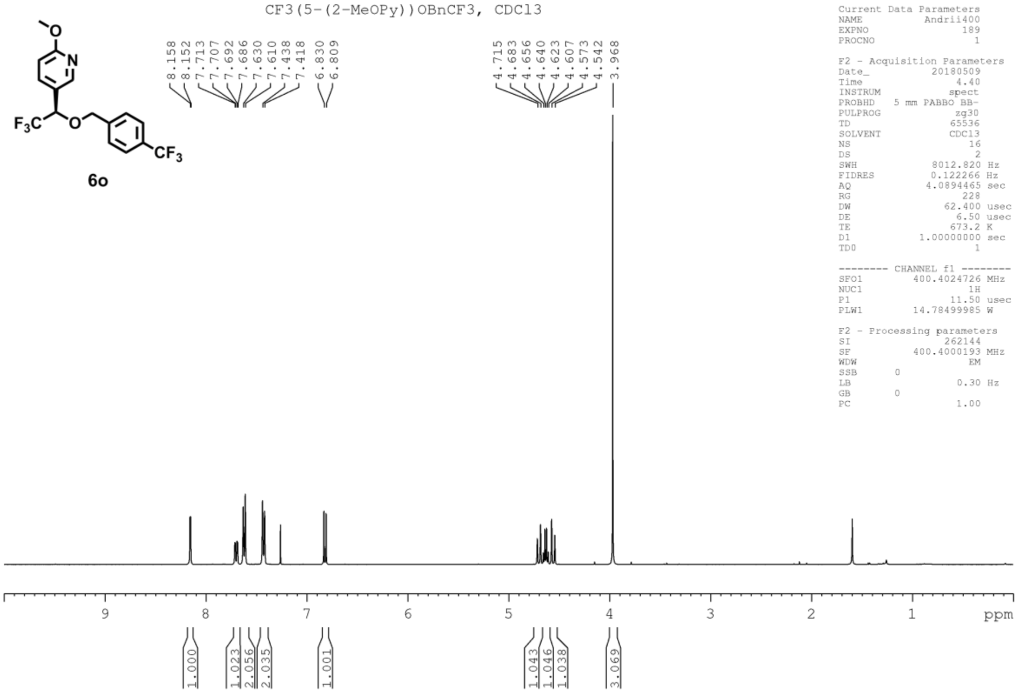

Supplementary figure 101. ^{1}H NMR spectra of **6o** in $CDCl_3$

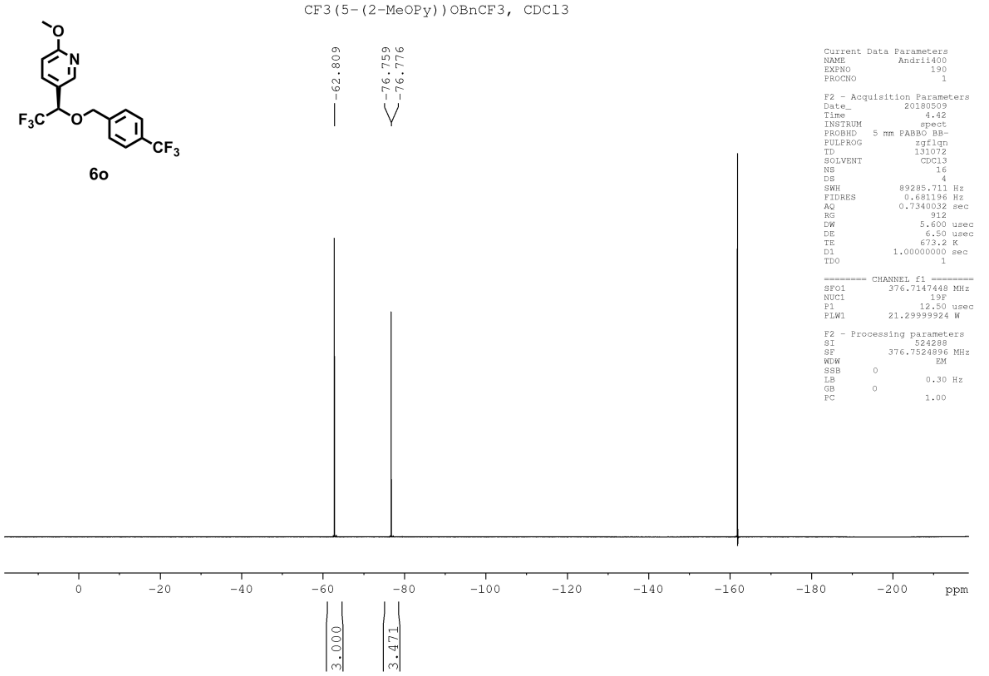

Supplementary figure 102. ^{19}F NMR spectra of **6o** in $CDCl_3$

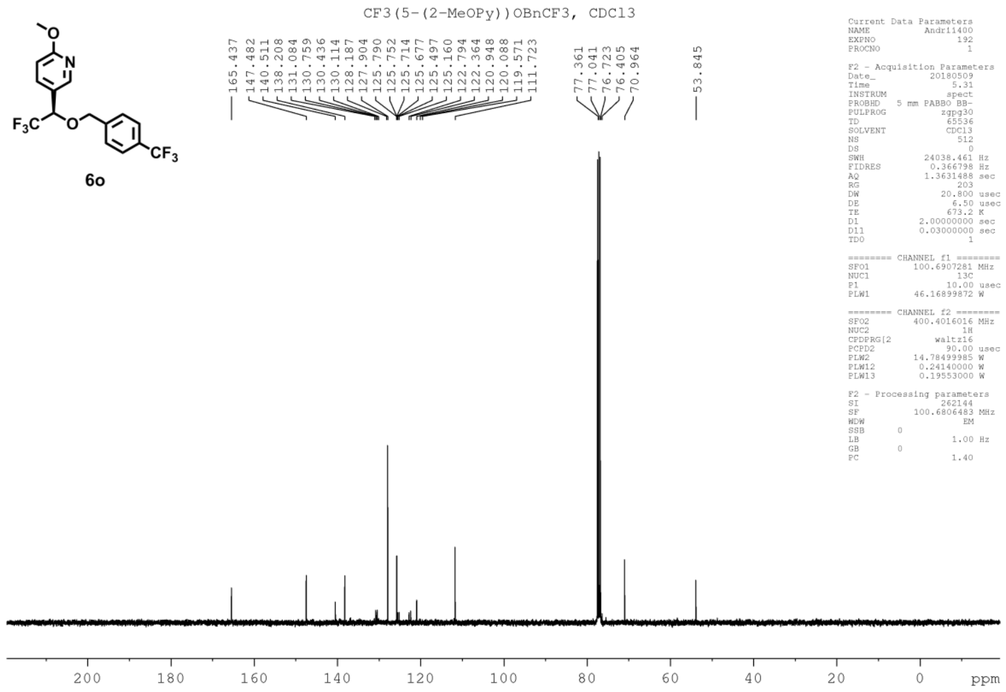

Supplementary figure 103. ^{13}C NMR spectra of **6o** in $CDCl_3$

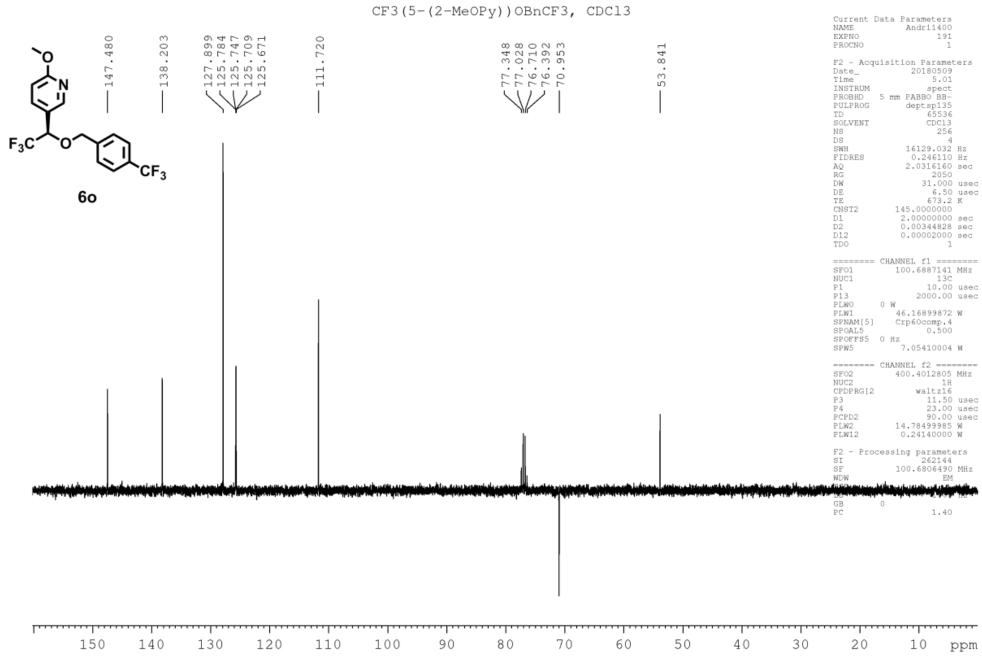

Supplementary figure 104. DEPT-135 NMR spectra of **6e** in $CDCl_3$

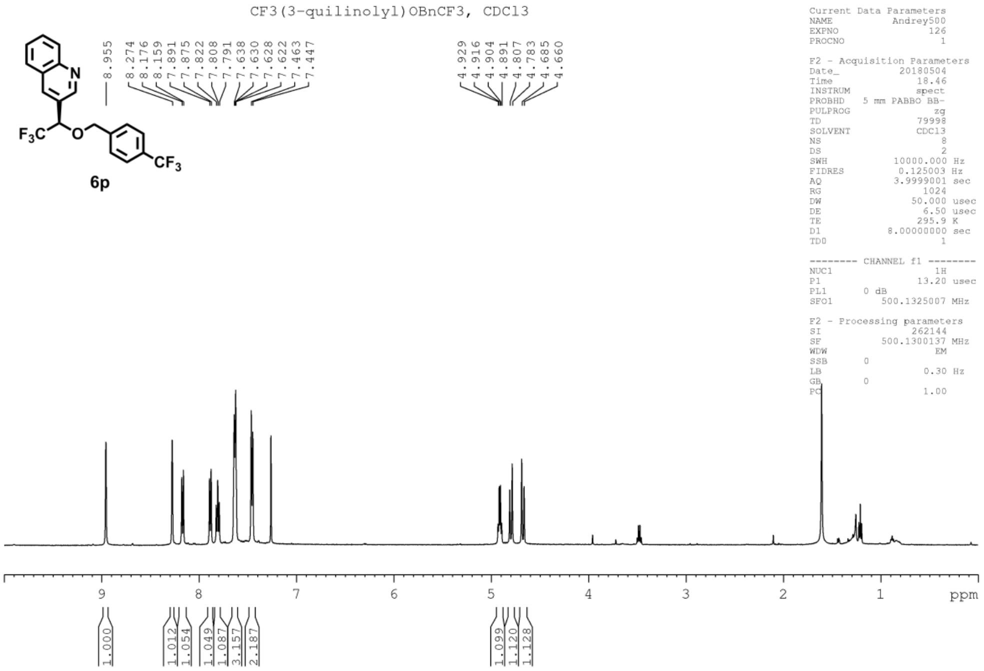

Supplementary figure 105. ^{1}H NMR spectra of **6p** in $CDCl_3$

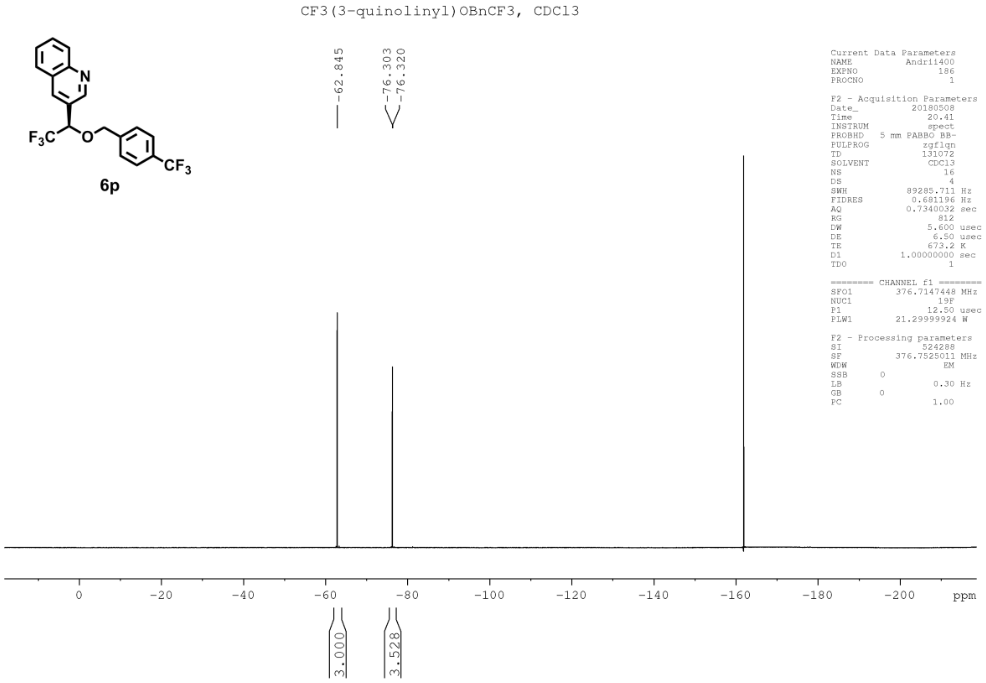

Supplementary figure 106. ^{19}F NMR spectra of **6p** in $CDCl_3$

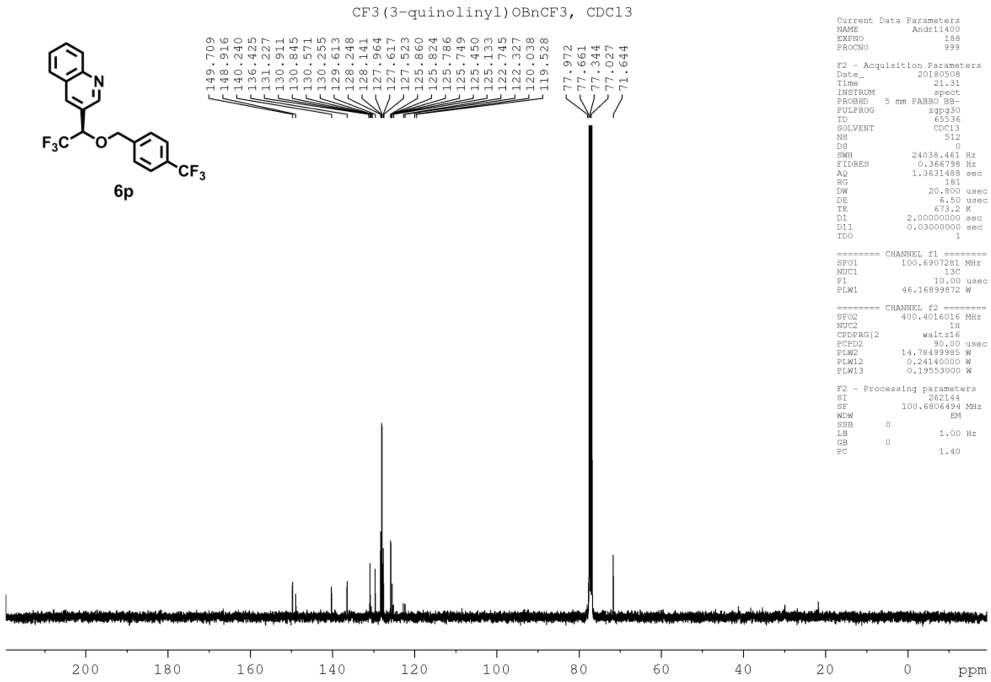

Supplementary figure 107. ^{13}C NMR spectra of **6p** in $CDCl_3$

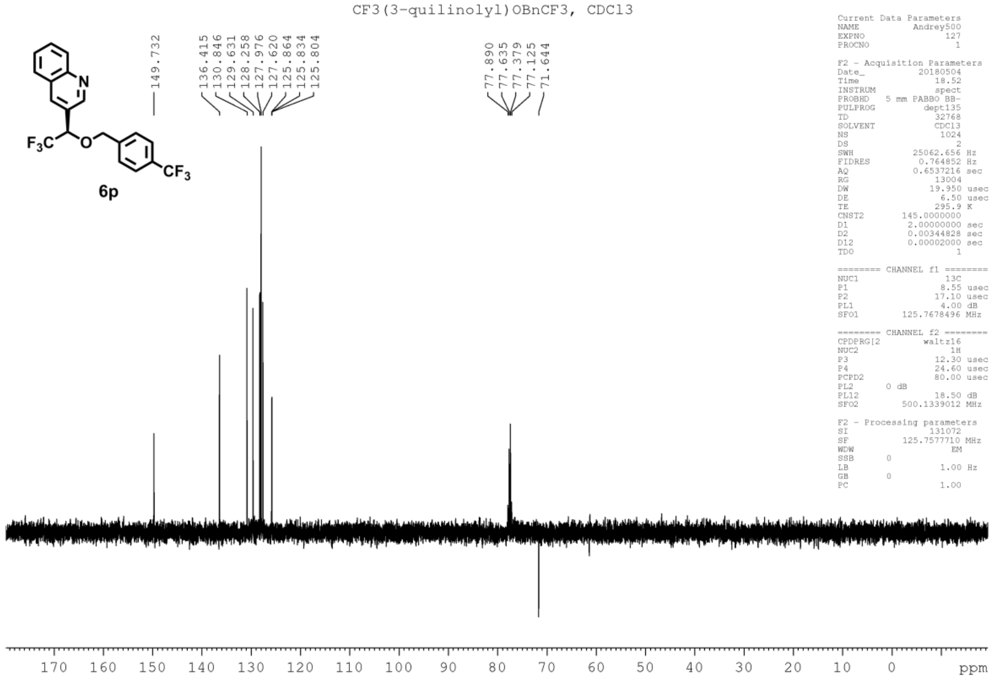

Supplementary figure 108. DEPT-135 spectra of **6p** in $CDCl_3$

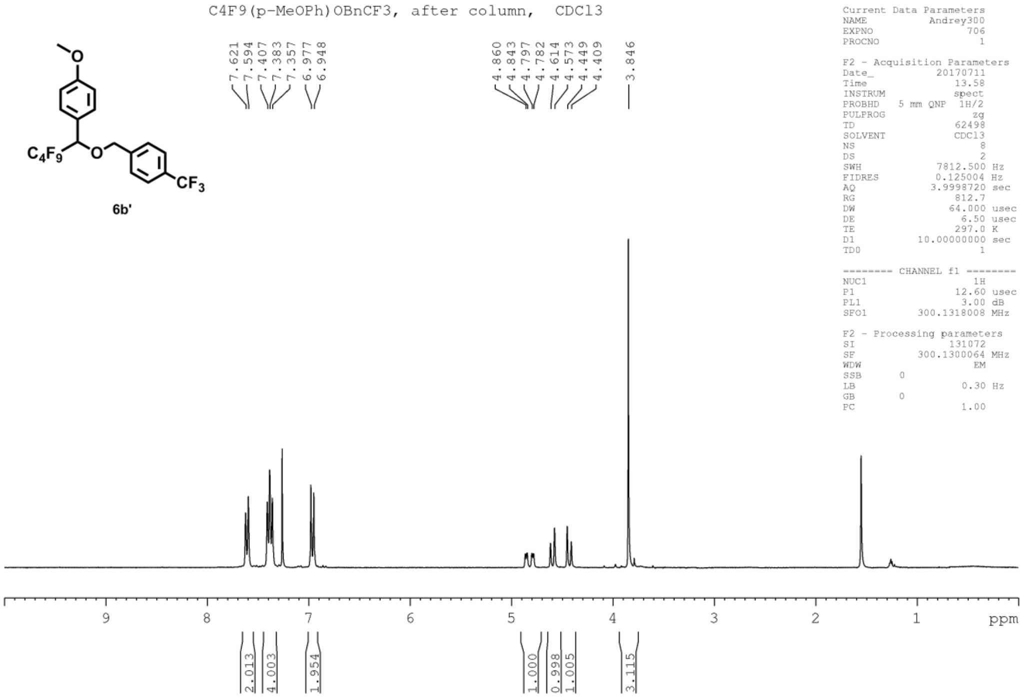

Supplementary figure 109. ^{1}H NMR spectra of **6b`** in $CDCl_3$

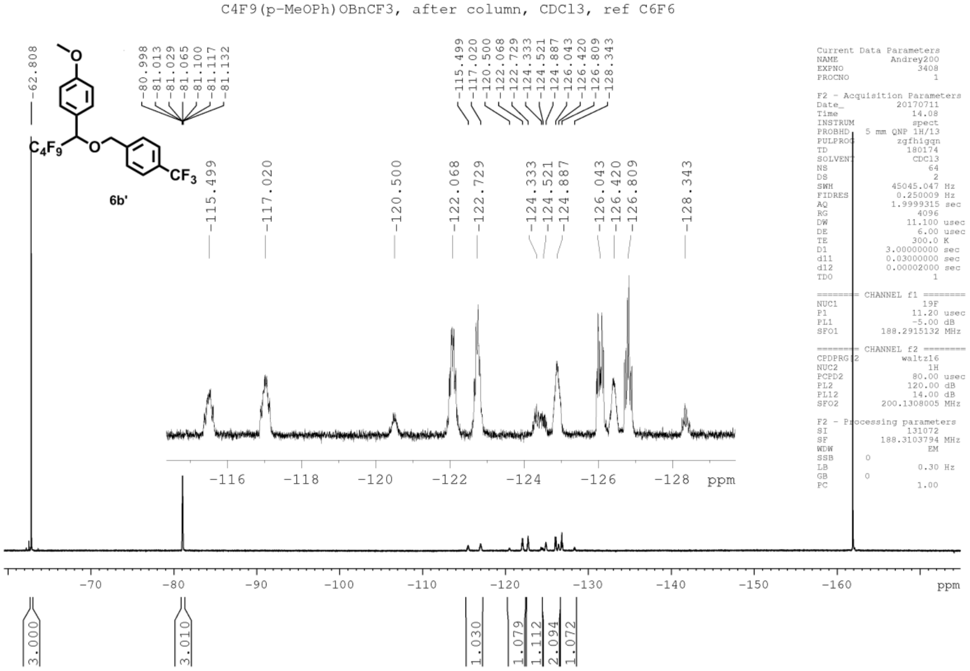

Supplementary figure 110. ^{19}F NMR spectra of **6b`** in $CDCl_3$

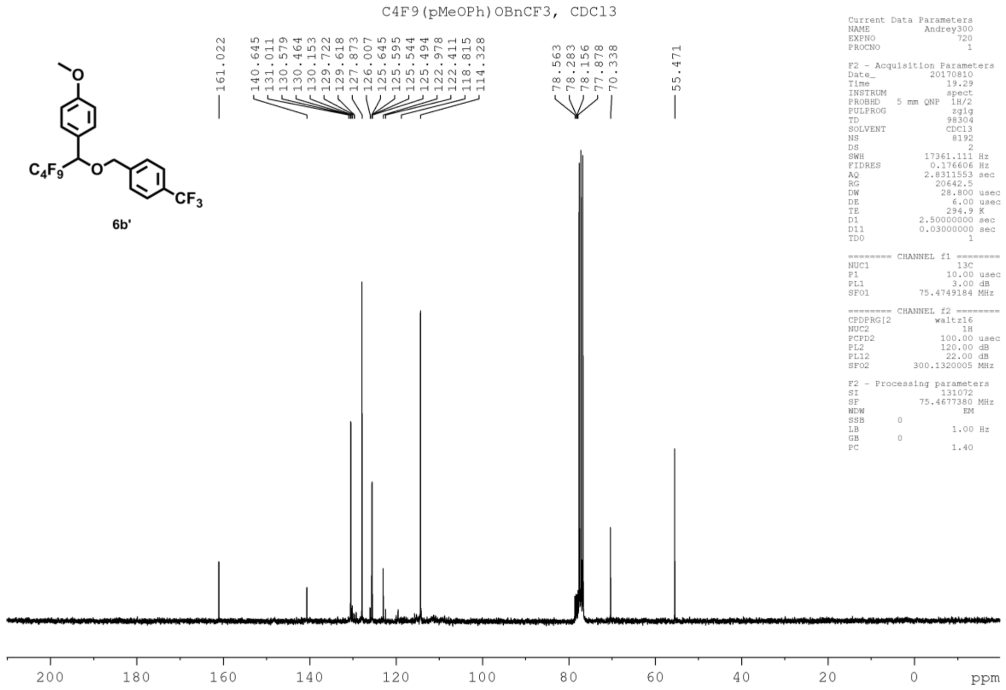

Supplementary figure 111. ^{13}C NMR spectra of **6b`** in $CDCl_3$

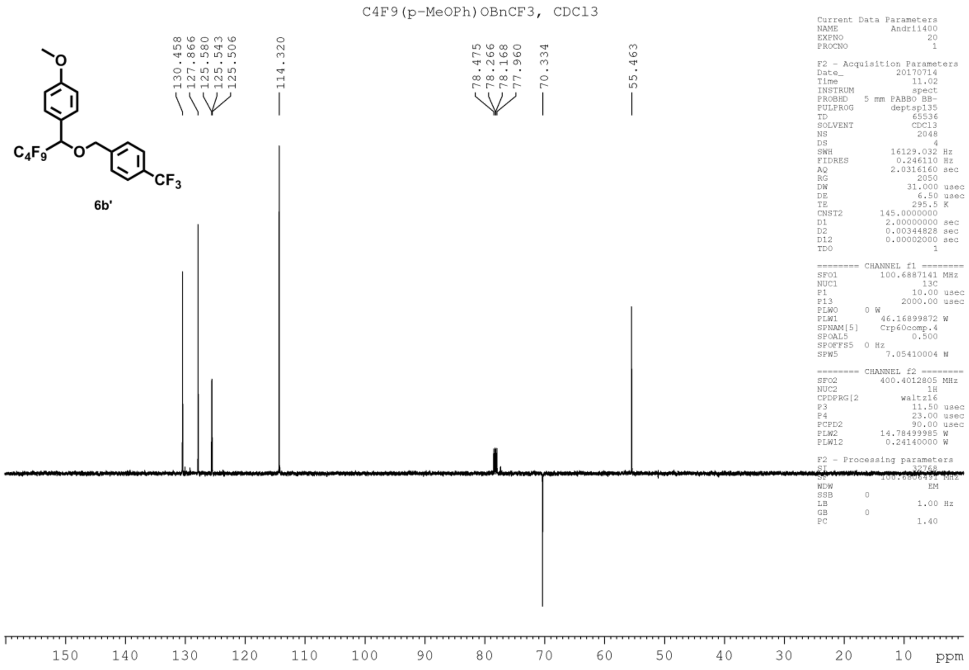

Supplementary figure 112. DEPT-135 NMR spectra of **6b`** in $CDCl_3$

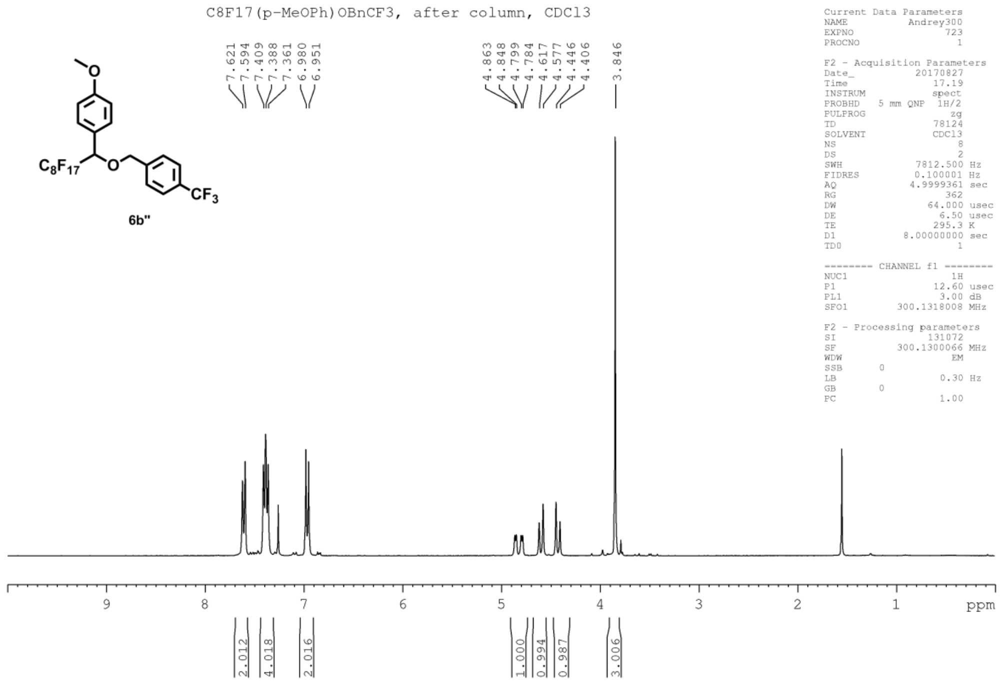

Supplementary figure 113. ^{1}H NMR spectra of **6b``** in $CDCl_3$

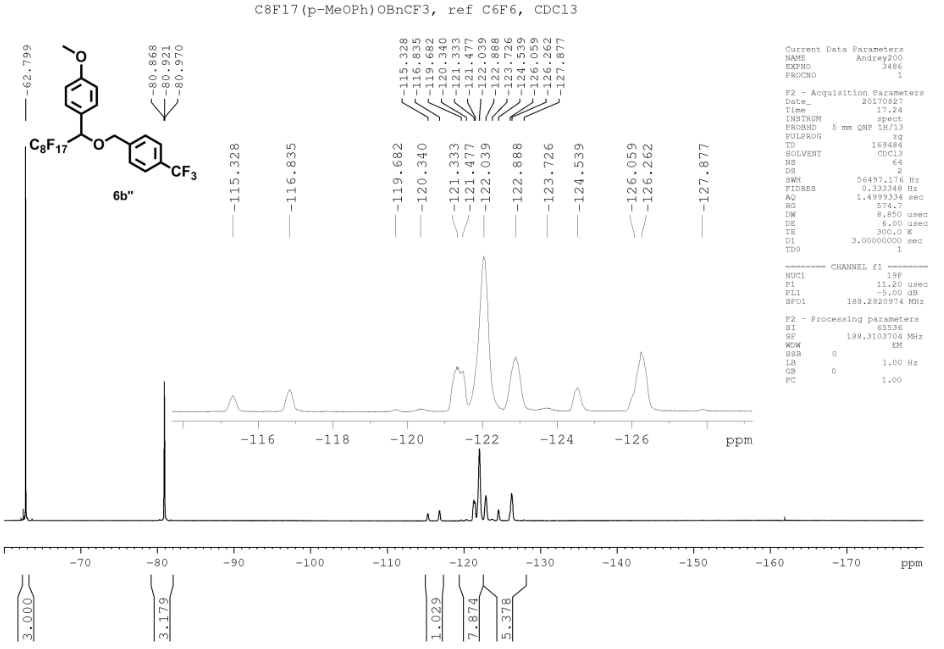

Supplementary figure 114. ^{19}F NMR spectra of **6b``** in $CDCl_3$

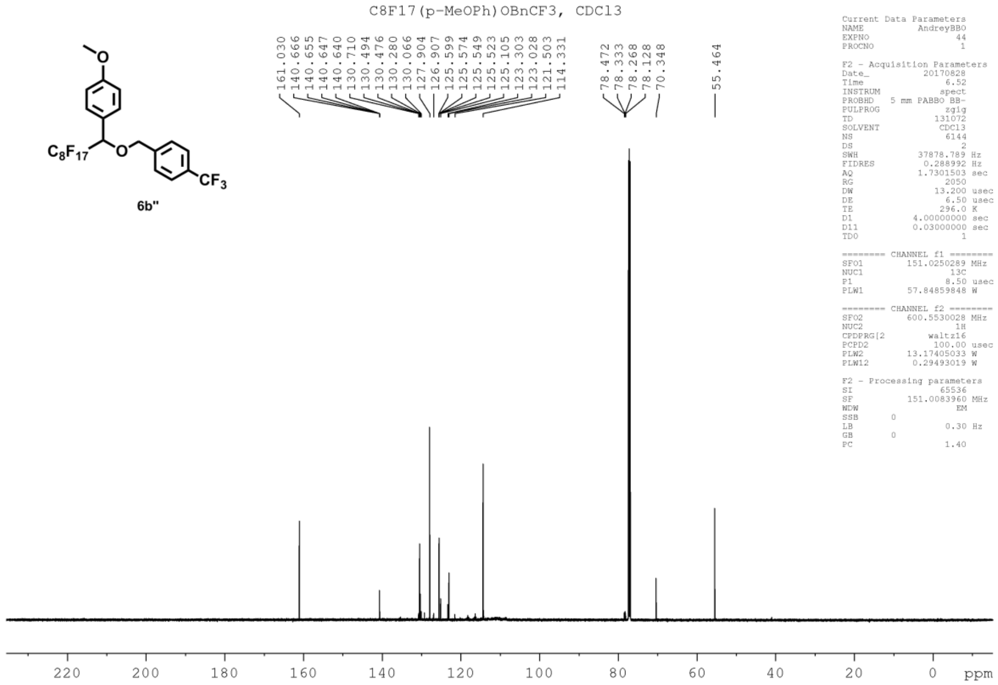

Supplementary figure 115. ^{13}C NMR spectra of **6b``** in $CDCl_3$

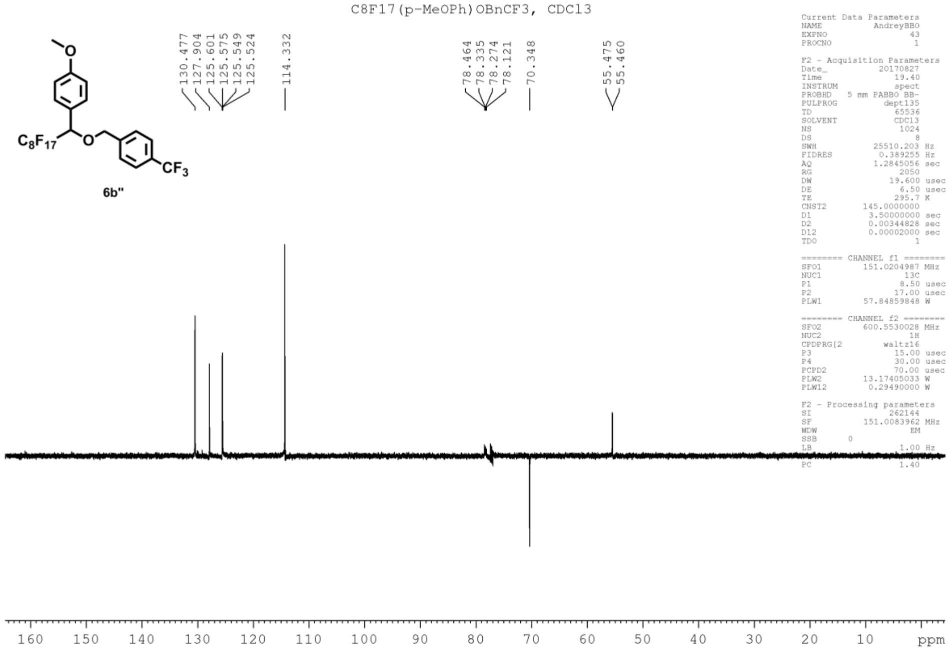

Supplementary figure 116. DEPT-135 NMR spectra of **6b``** in $CDCl_3$

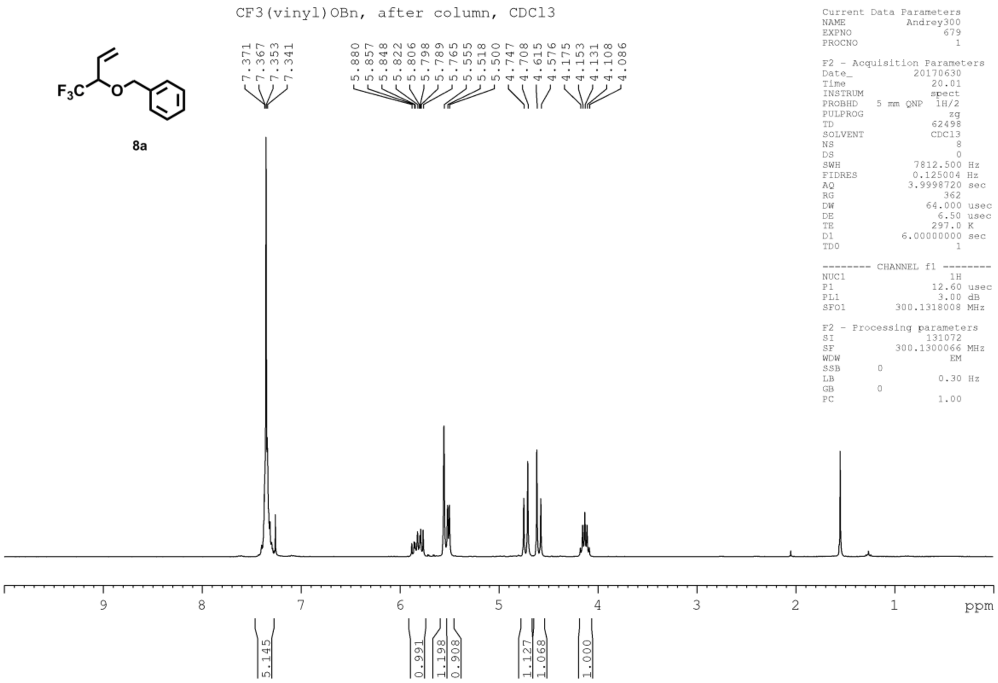

Supplementary figure 117. ^{1}H NMR spectra of **8a** in $CDCl_3$

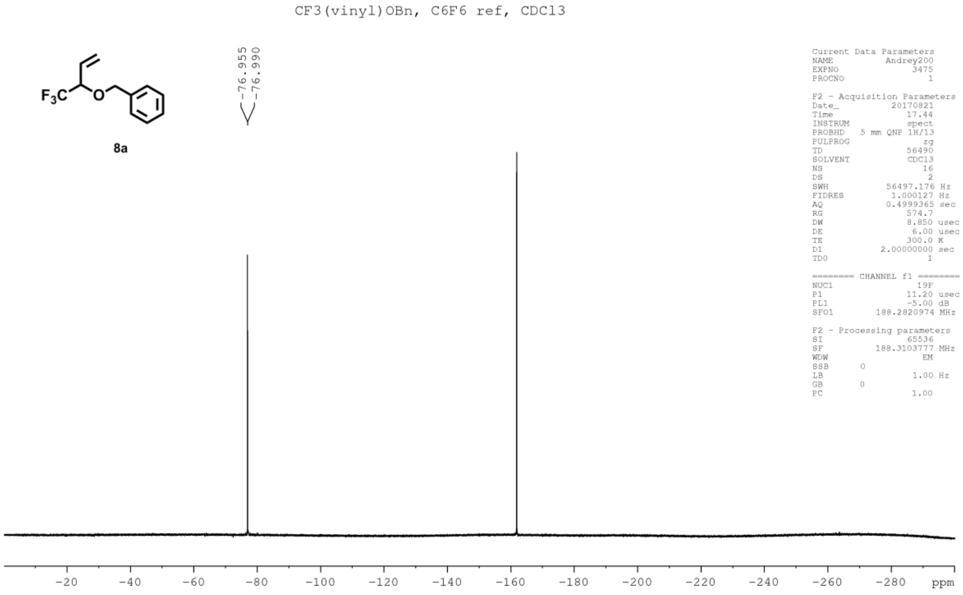

Supplementary figure 118. ^{19}F NMR spectra of **8a** in $CDCl_3$

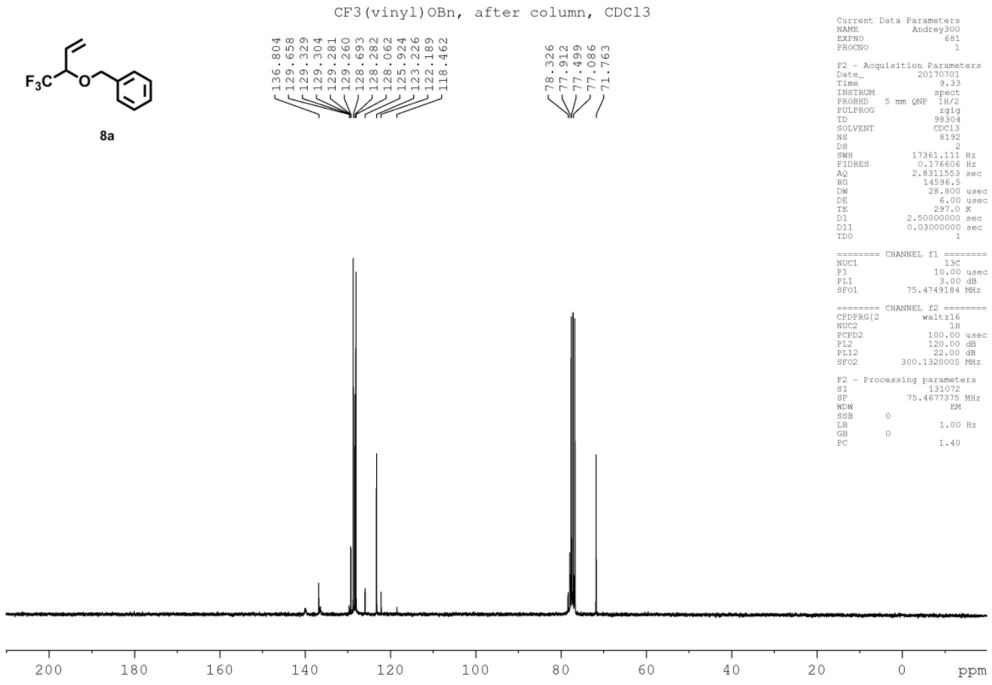

Supplementary figure 119. ^{13}C NMR spectra of **8a** in $CDCl_3$

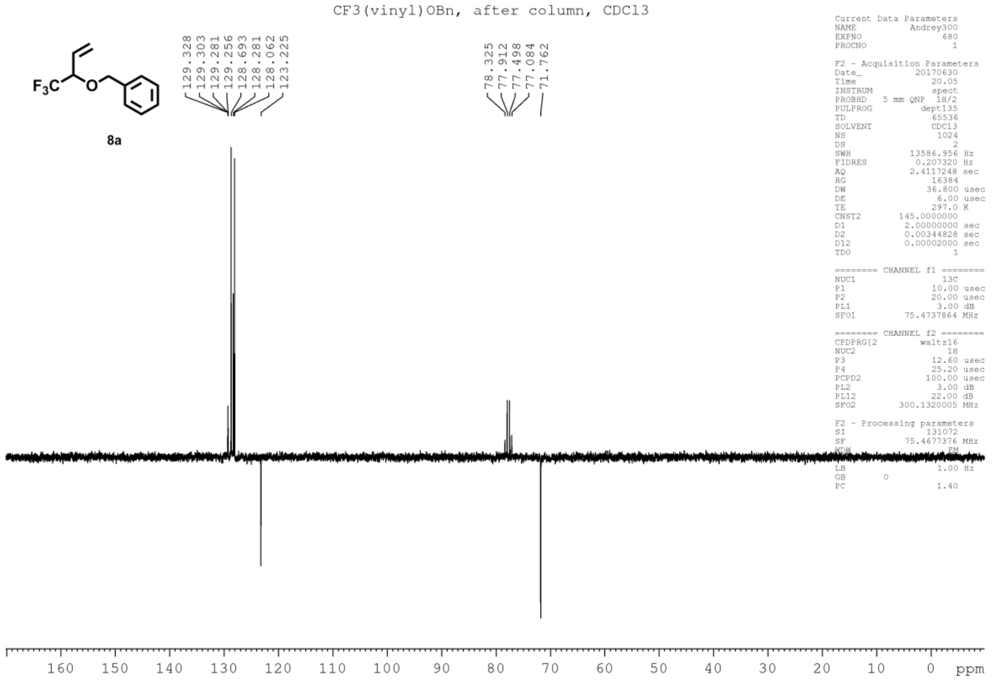

Supplementary figure 120. DEPT-135 NMR spectra of **8a** in $CDCl_3$

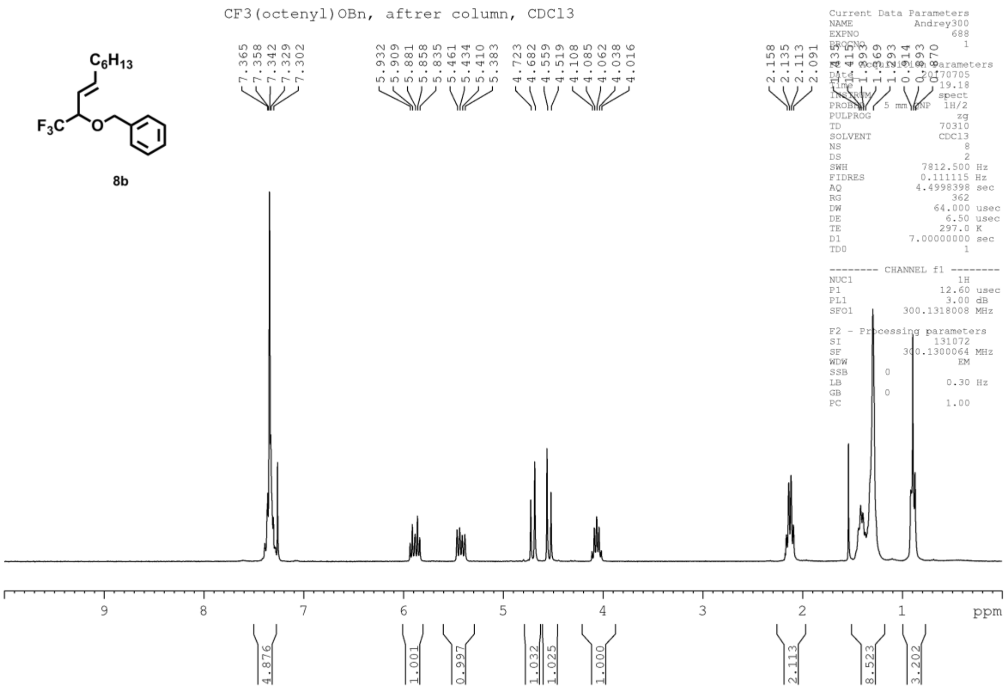

Supplementary figure 121. ^{1}H NMR spectra of **8b** in $CDCl_3$

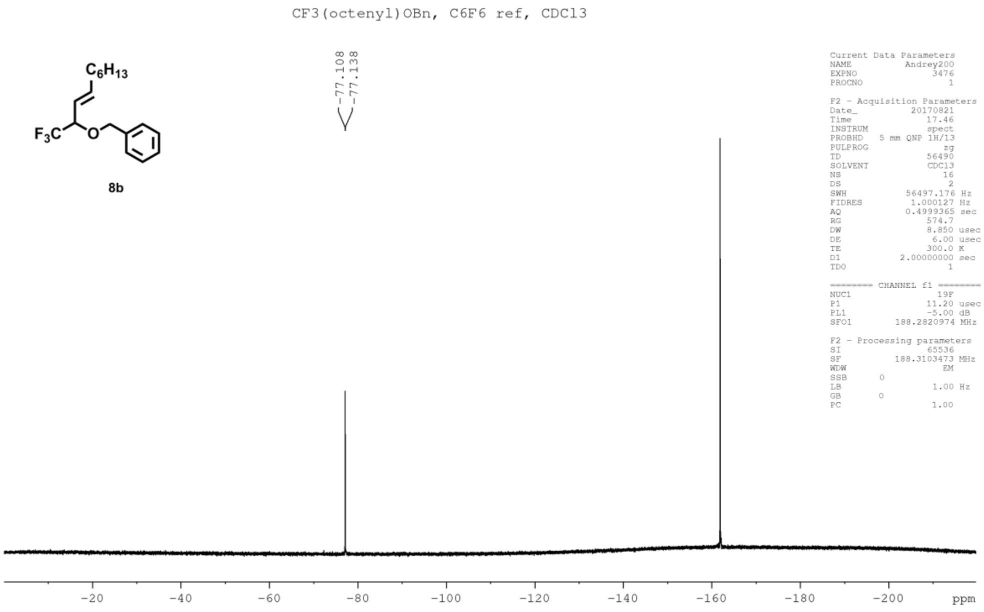

Supplementary figure 122. ^{19}F NMR spectra of **8b** in $CDCl_3$

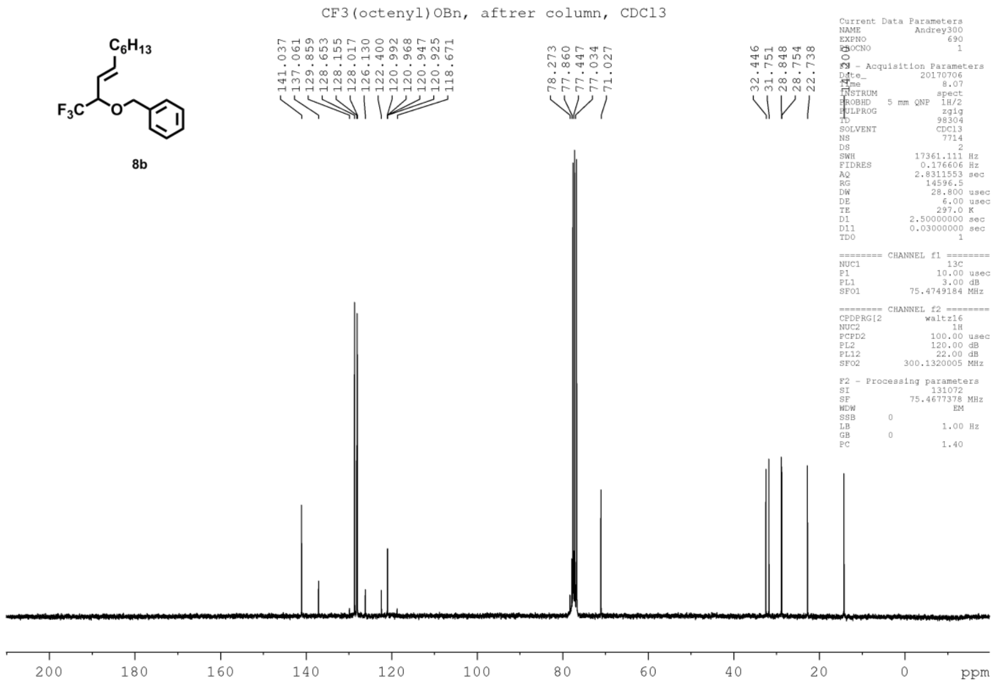

Supplementary figure 123. ^{13}C NMR spectra of **8b** in $CDCl_3$

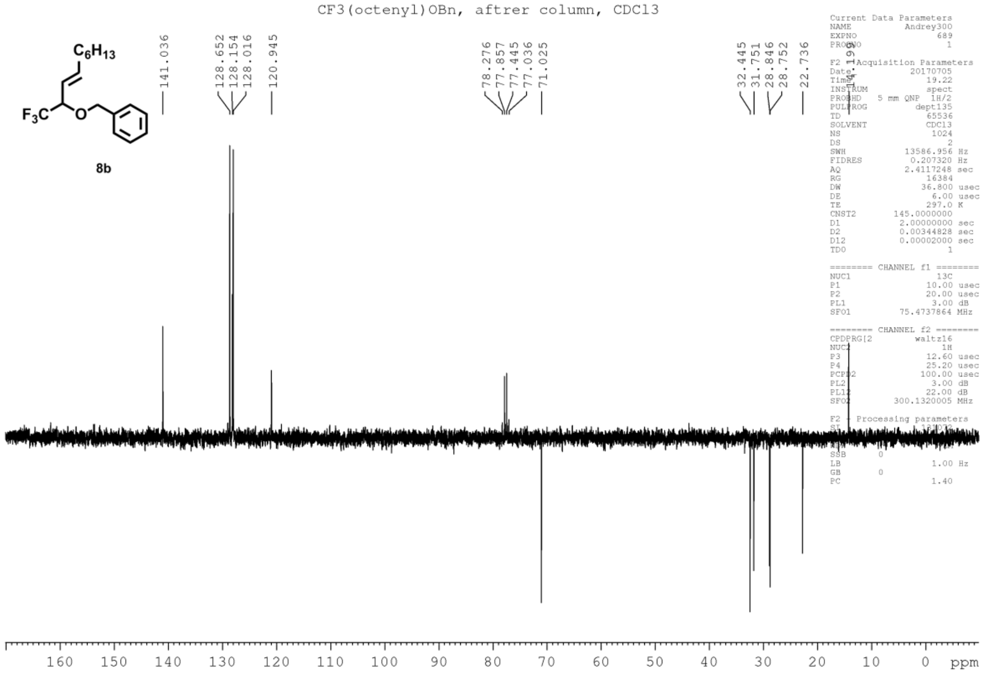

Supplementary figure 124. DEPT-135 NMR spectra of **8b** in $CDCl_3$

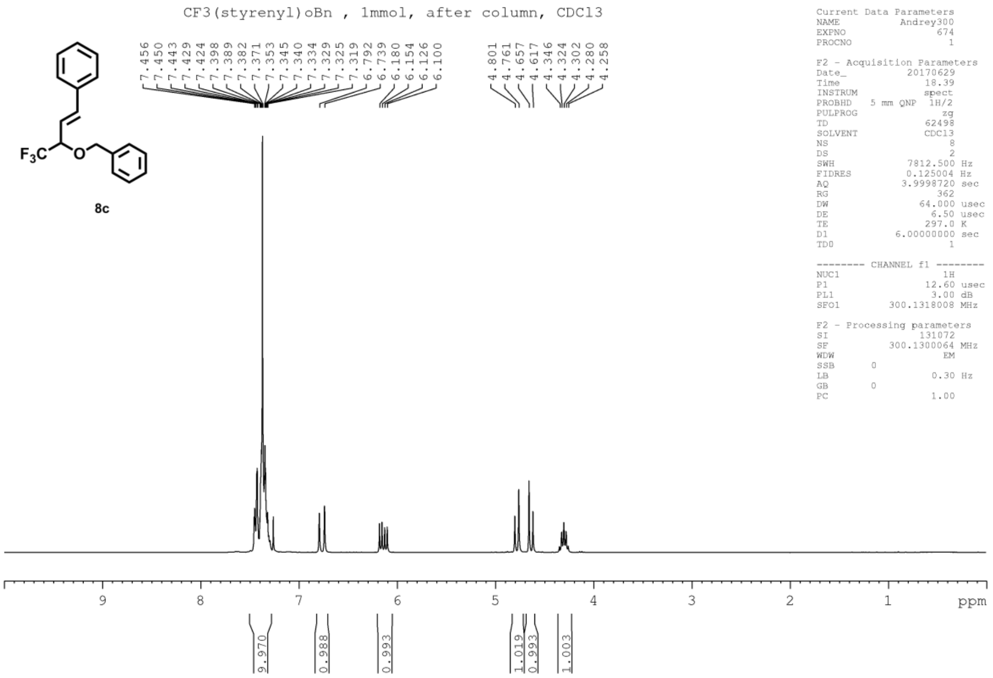

Supplementary figure 125. ^{1}H NMR spectra of **8c** in $CDCl_3$

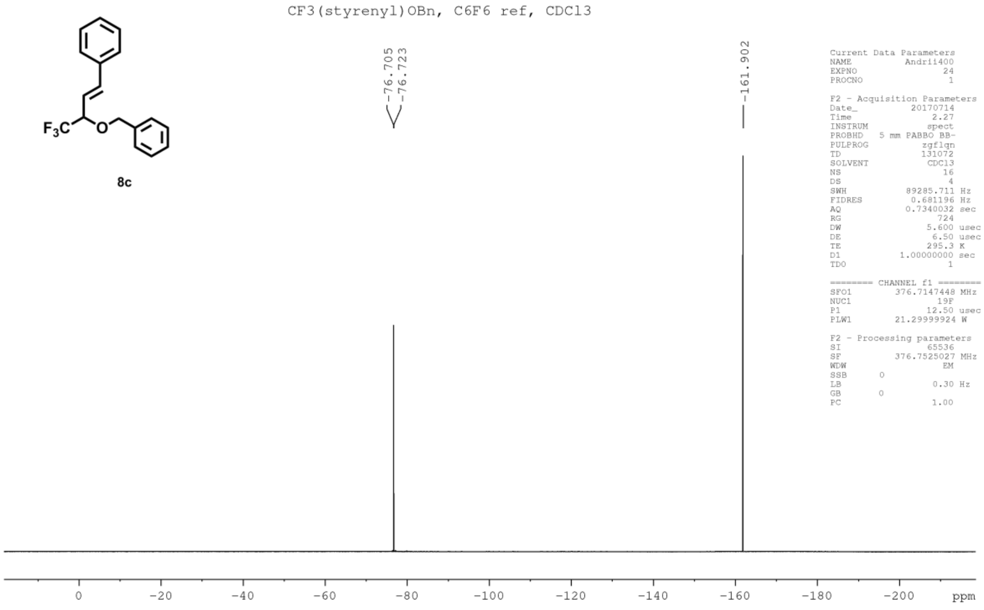

Supplementary figure 126. ^{19}F NMR spectra of **8c** in $CDCl_3$

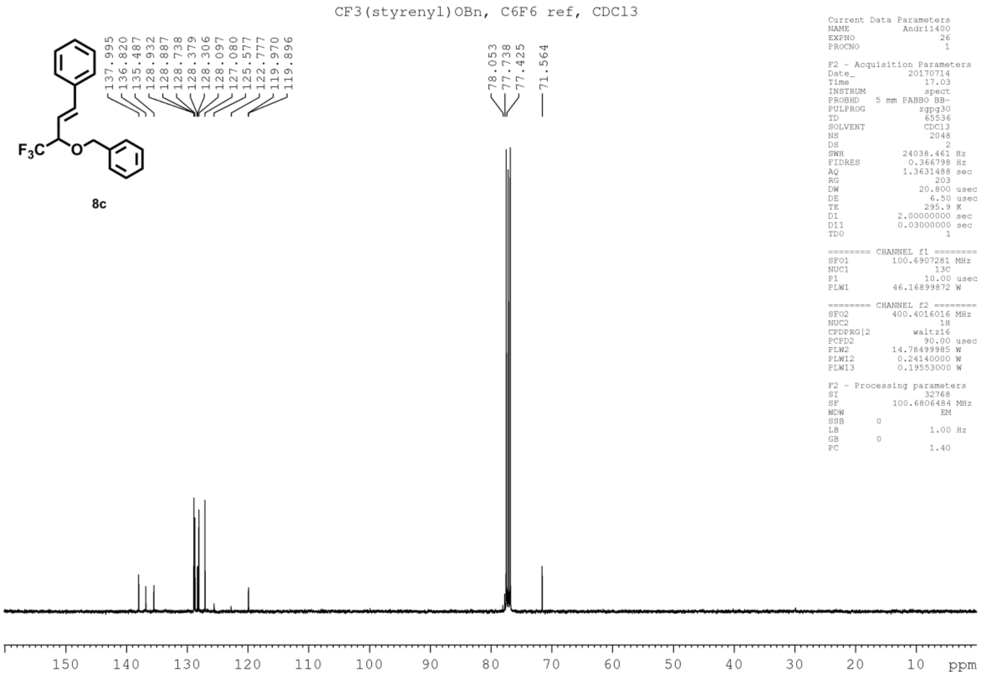

Supplementary figure 127. ^{13}C NMR spectra of **8c** in $CDCl_3$

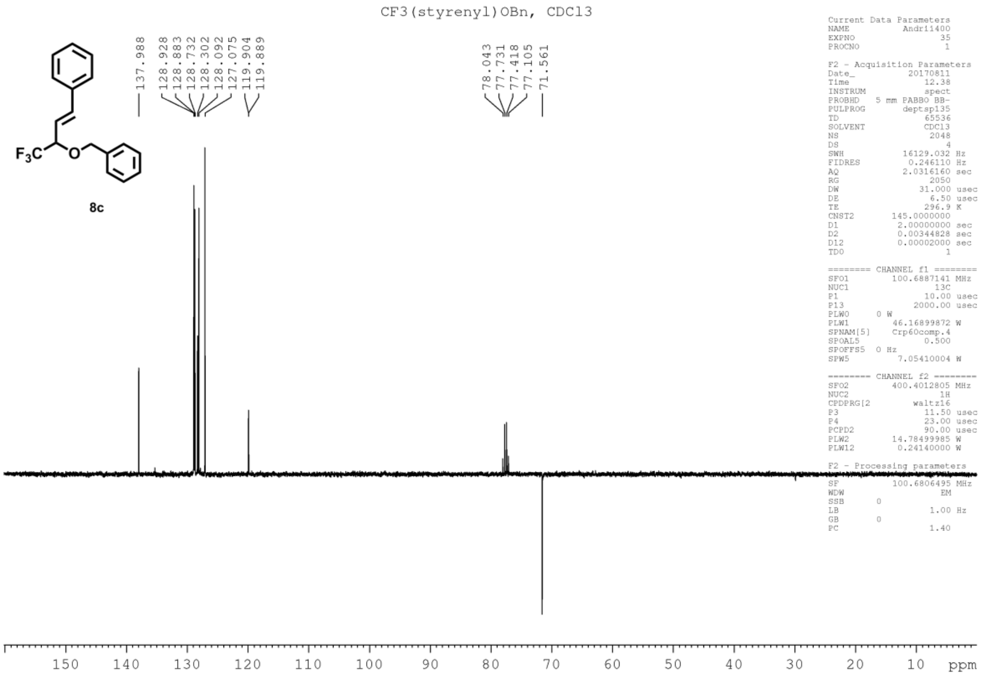

Supplementary figure 128. DEPT-135 NMR spectra of **8c** in $CDCl_3$

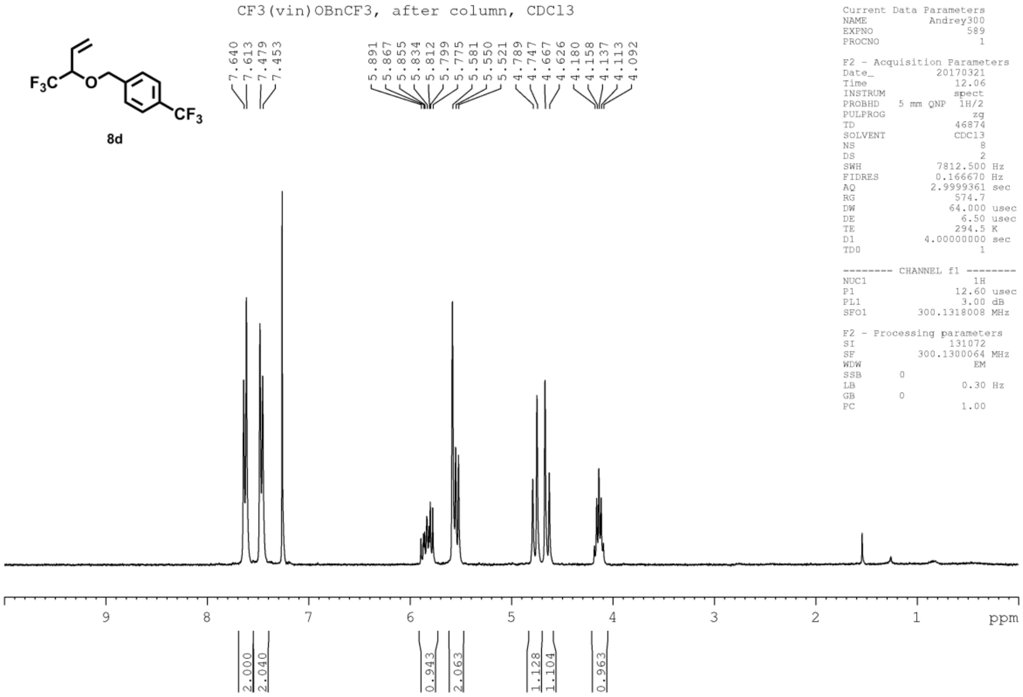

Supplementary figure 129. ^{1}H NMR spectra of **8d** in $CDCl_3$

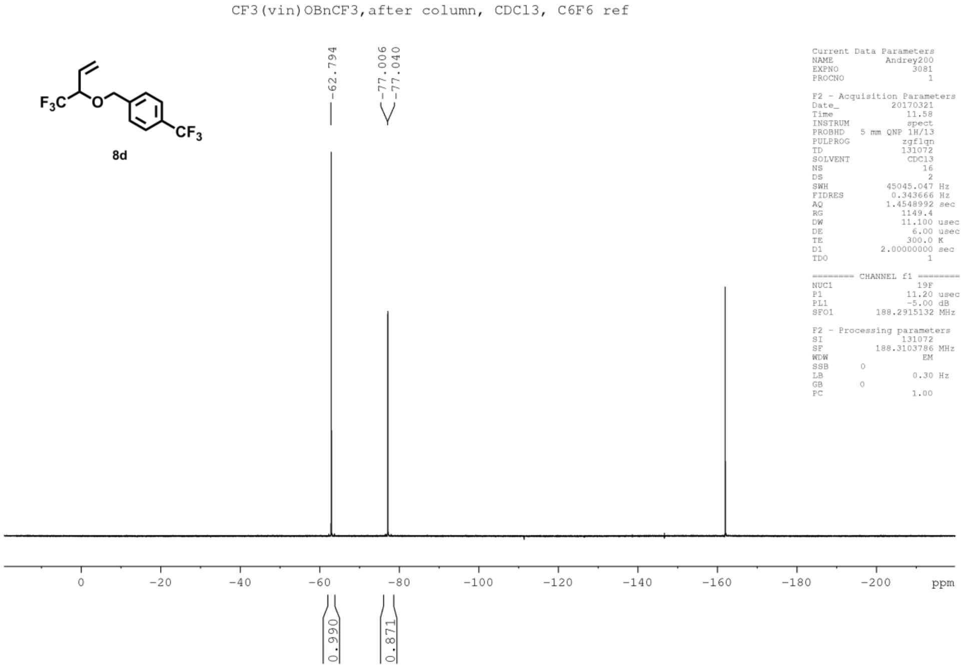

Supplementary figure 130. ^{19}F NMR spectra of **8d** in $CDCl_3$

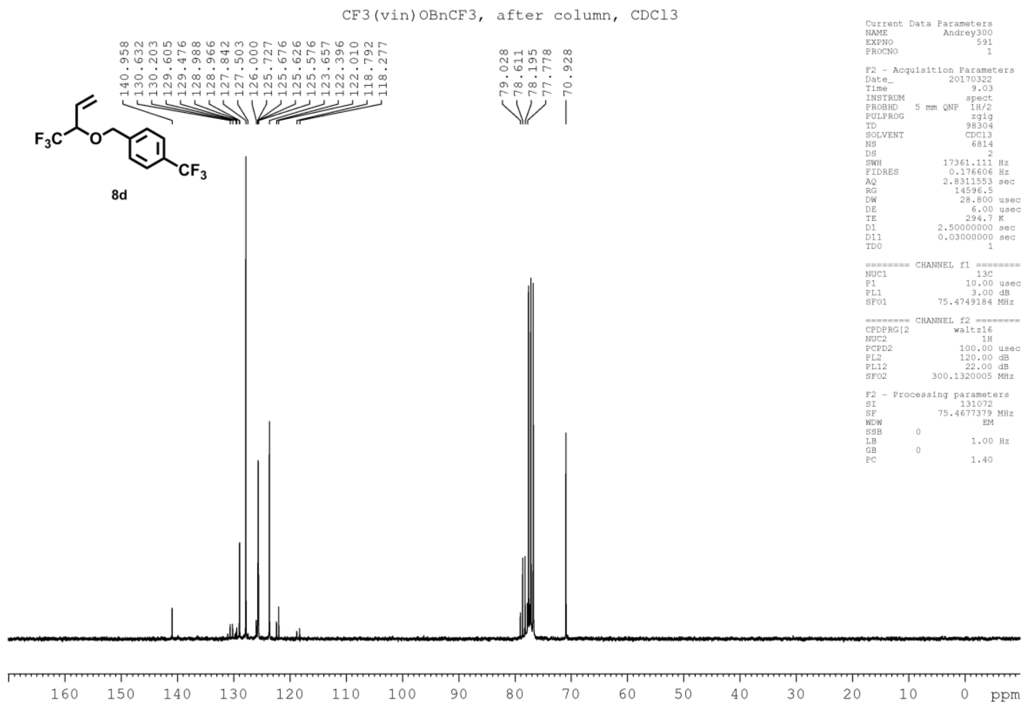

Supplementary figure 131. ^{13}C NMR spectra of **8d** in $CDCl_3$

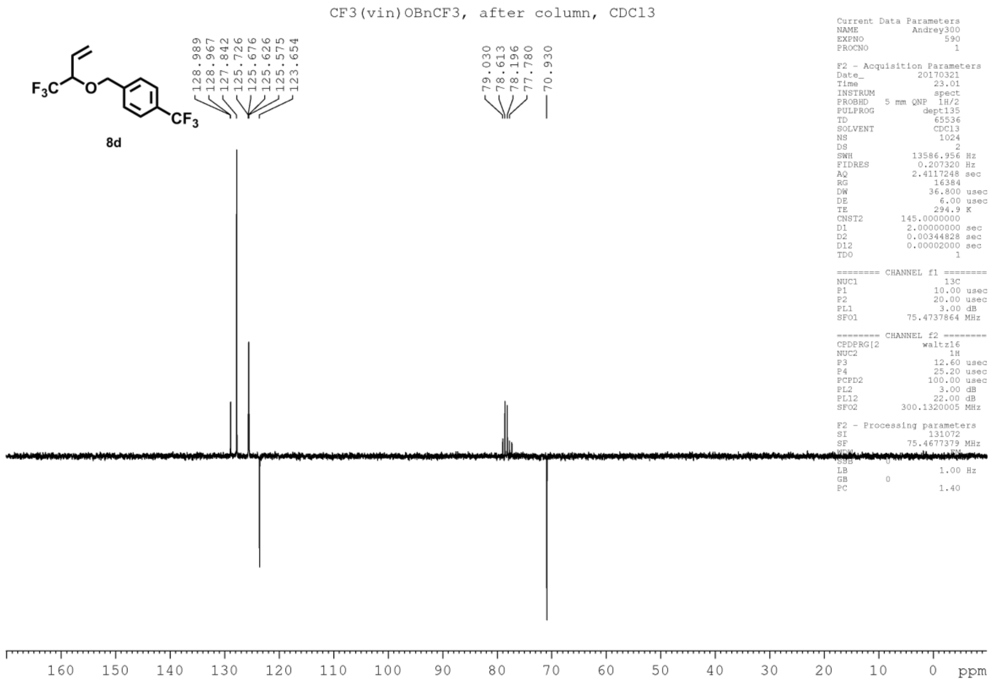

Supplementary figure 132. DEPT-135 NMR spectra of **8d** in $CDCl_3$

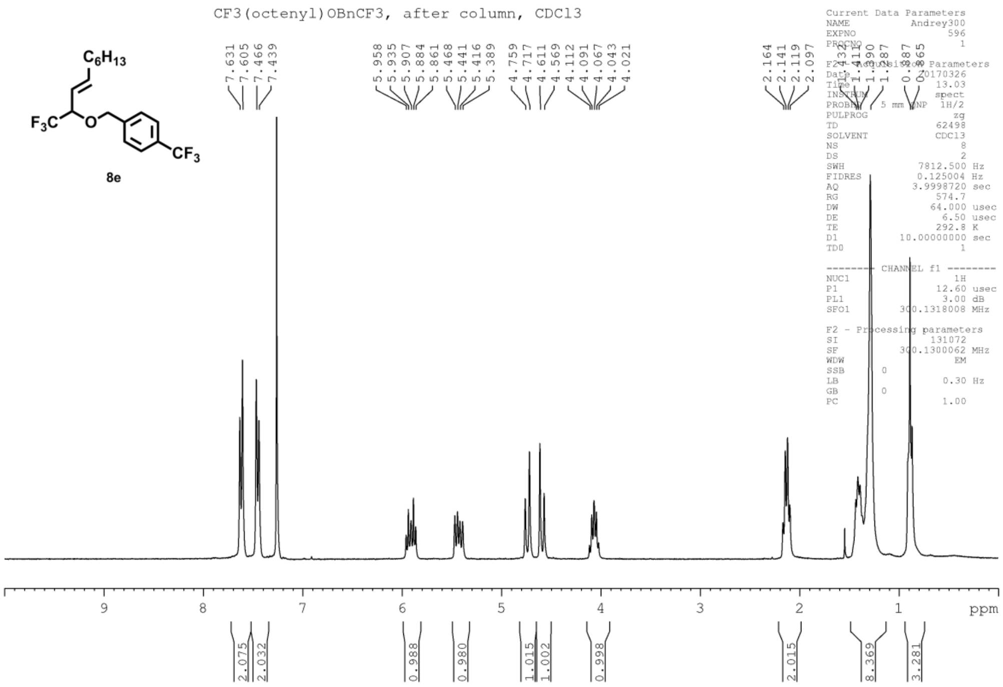

Supplementary figure 133. ^{1}H NMR spectra of **8e** in $CDCl_3$

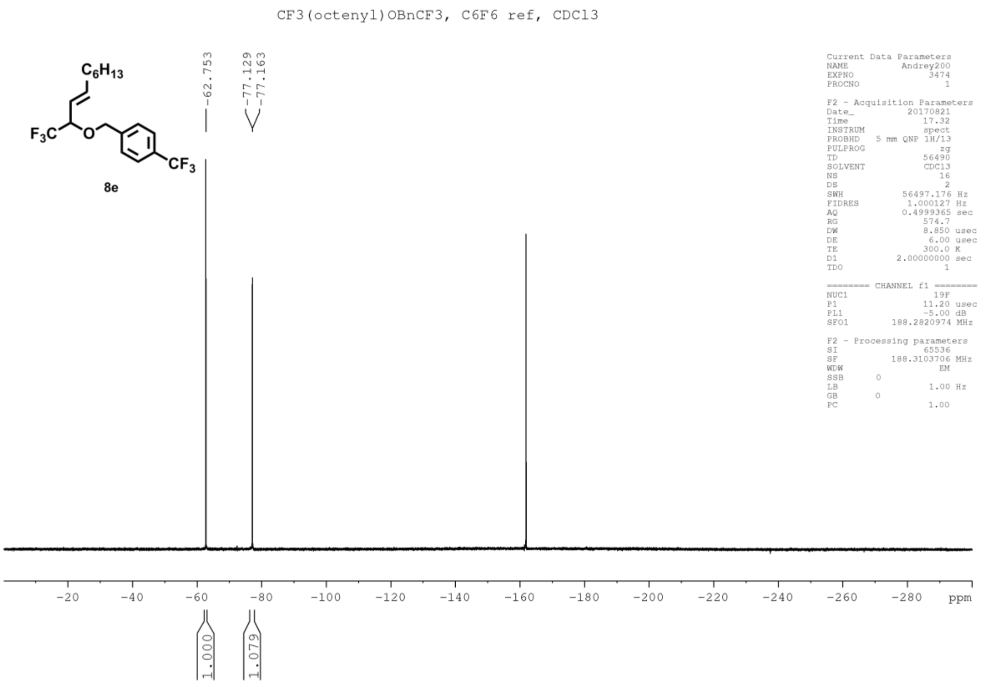

Supplementary figure 134. ^{19}F NMR spectra of **8e** in $CDCl_3$

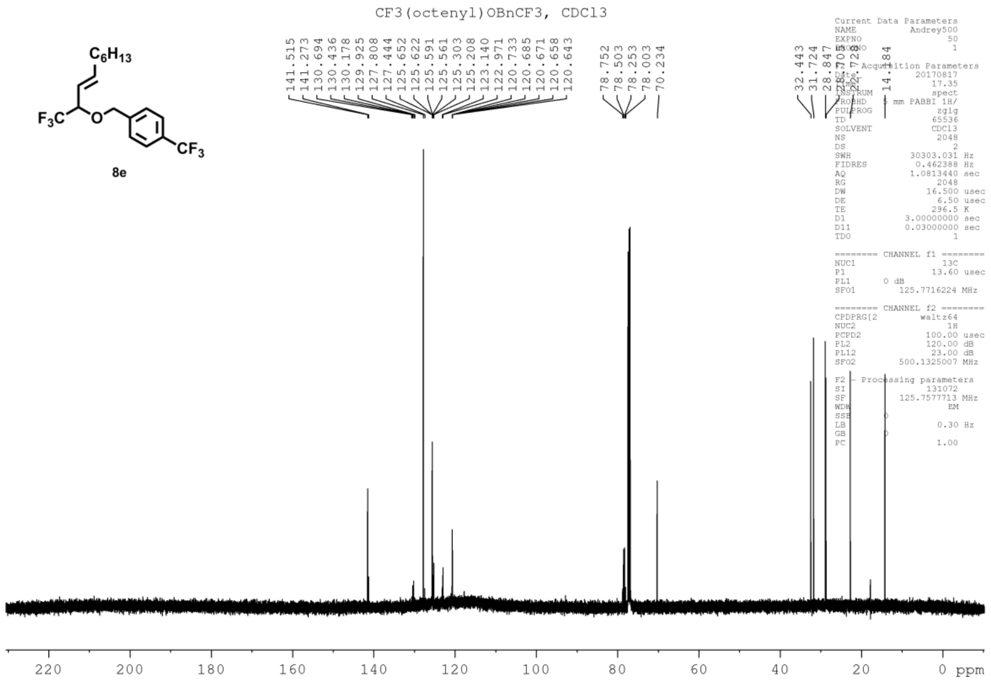

Supplementary figure 135. ^{13}C NMR spectra of **8e** in $CDCl_3$

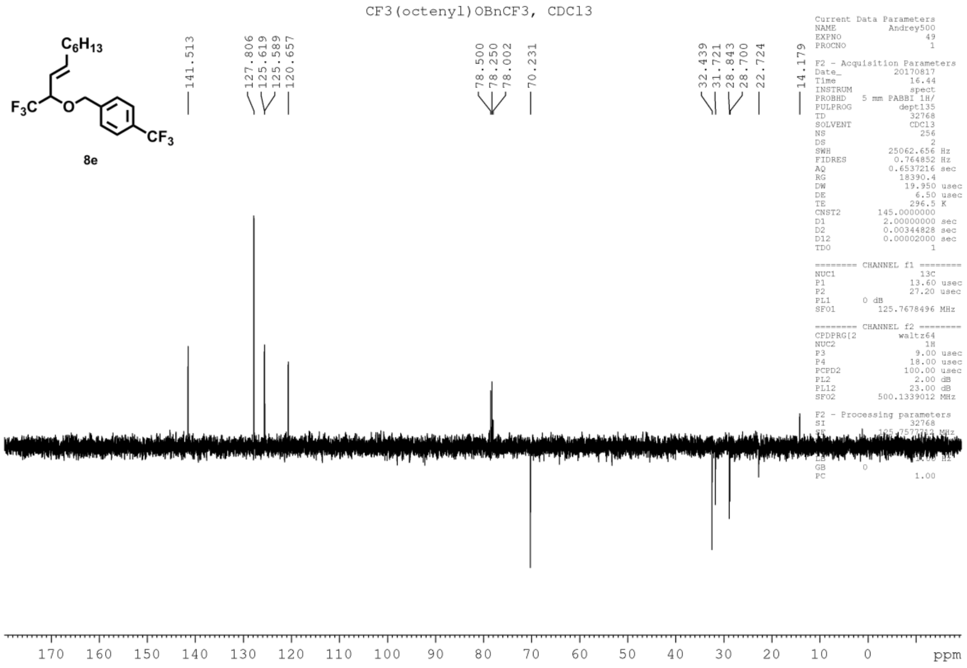

Supplementary figure 136. DEPT-135 NMR spectra of **8e** in $CDCl_3$

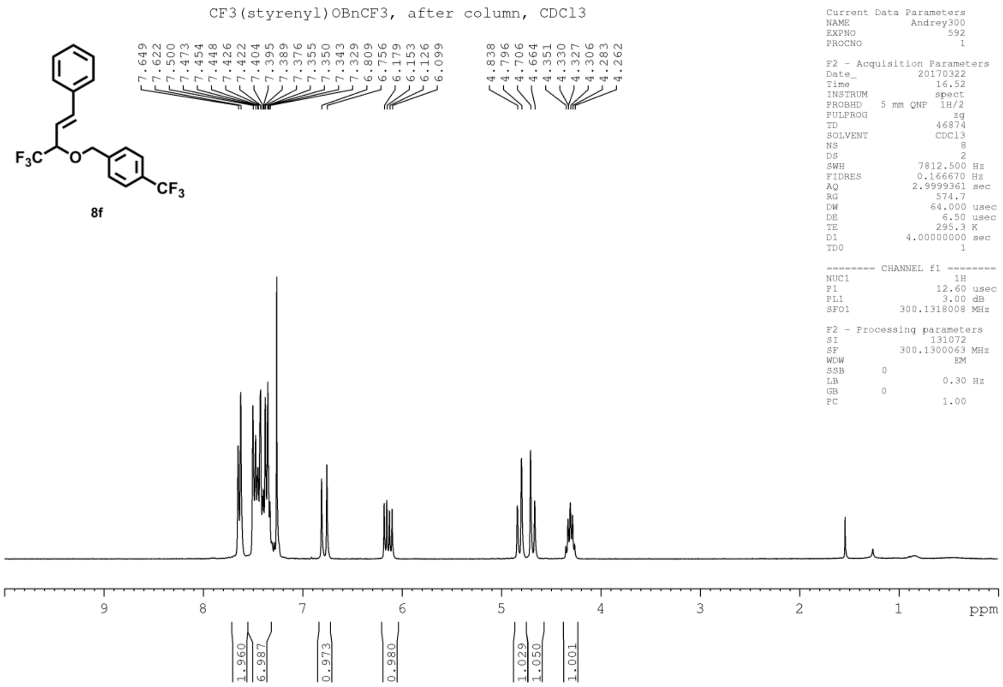

Supplementary figure 137. ^{1}H NMR spectra of **8f** in $CDCl_3$

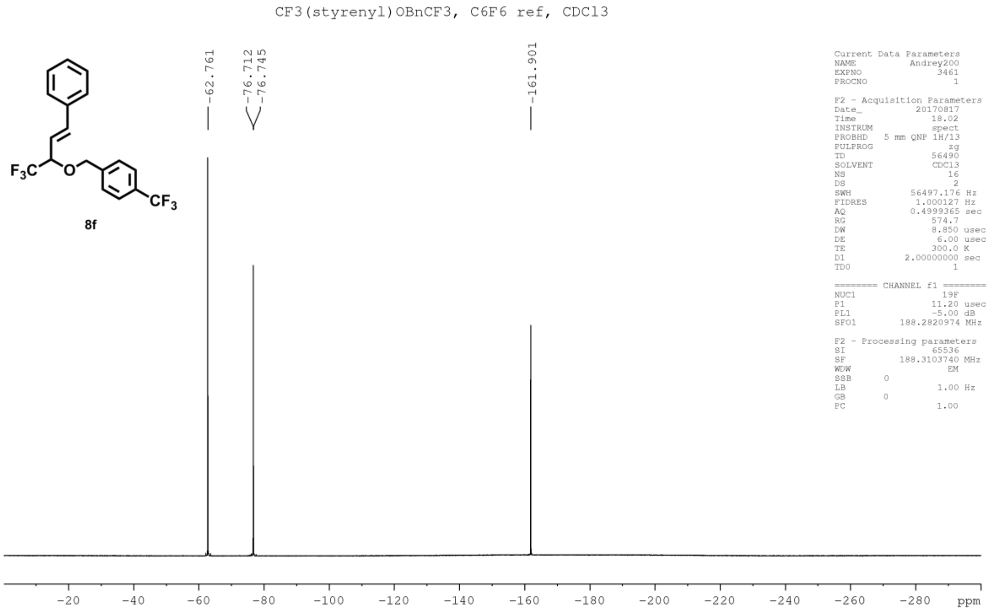

Supplementary figure 138. ^{19}F NMR spectra of **8f** in $CDCl_3$

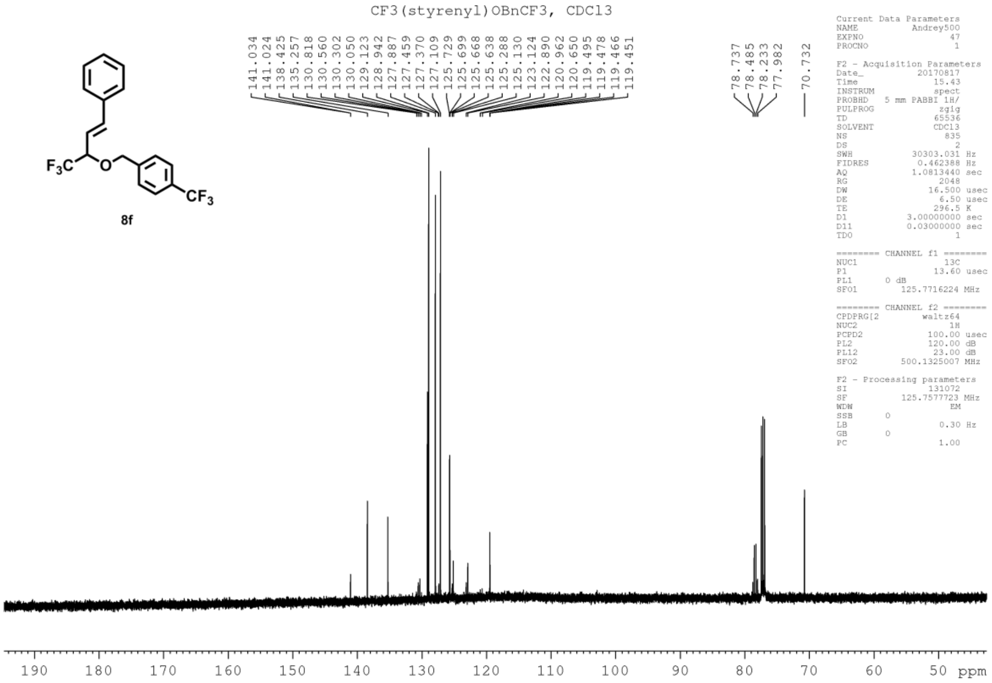

Supplementary figure 139. ^{13}C NMR spectra of **8f** in $CDCl_3$

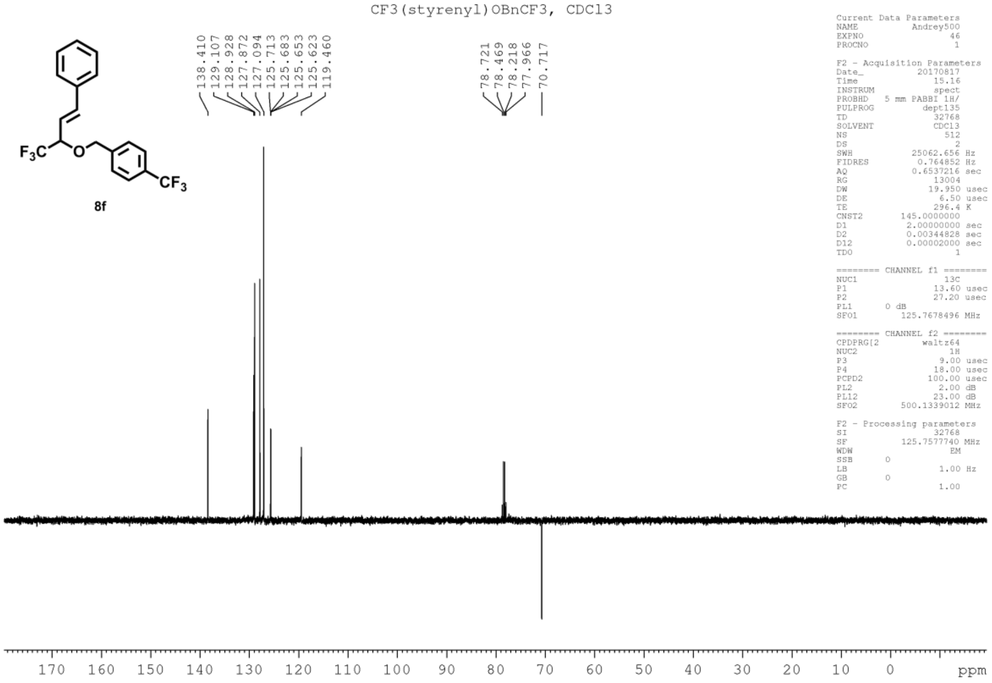

Supplementary figure 140. DEPT-135 NMR spectra of **8f** in $CDCl_3$

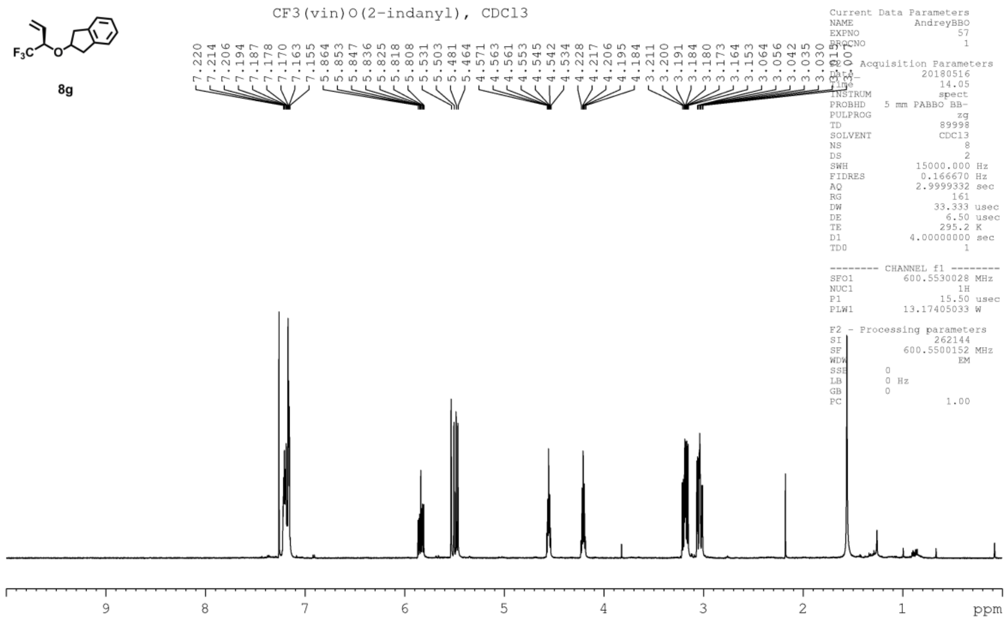

Supplementary figure 141. ^{1}H NMR spectra of **8g** in $CDCl_3$

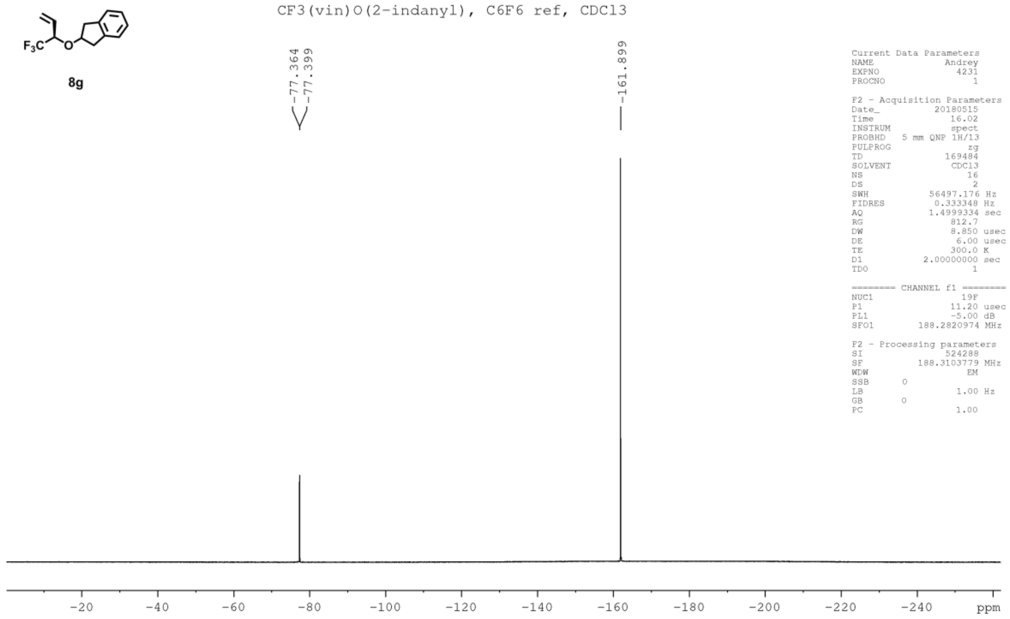

Supplementary figure 142. ^{19}F NMR spectra of **8g** in $CDCl_3$

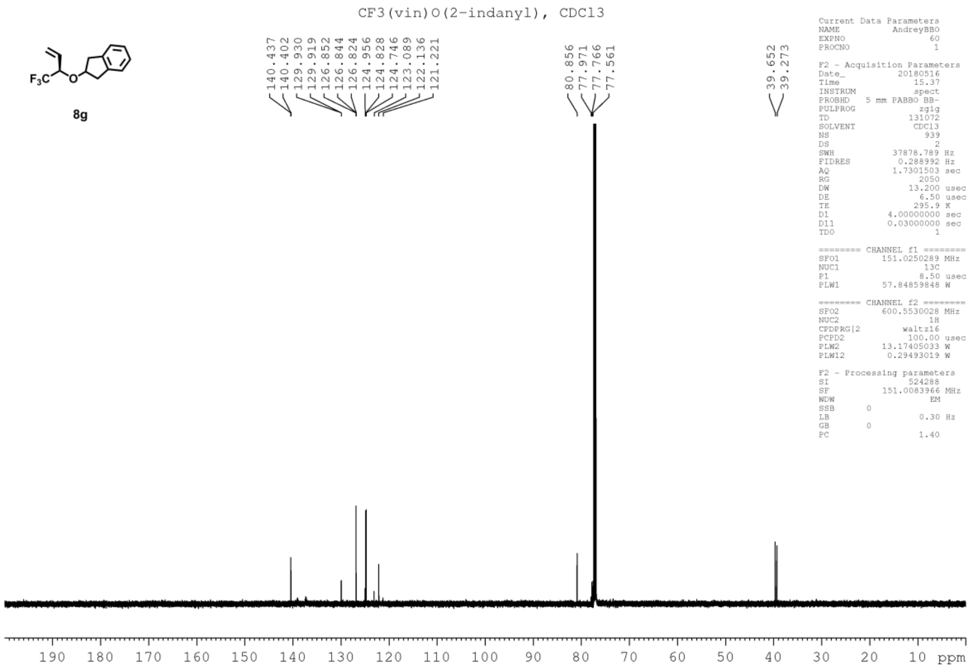

Supplementary figure 143. ^{13}C NMR spectra of **8g** in $CDCl_3$

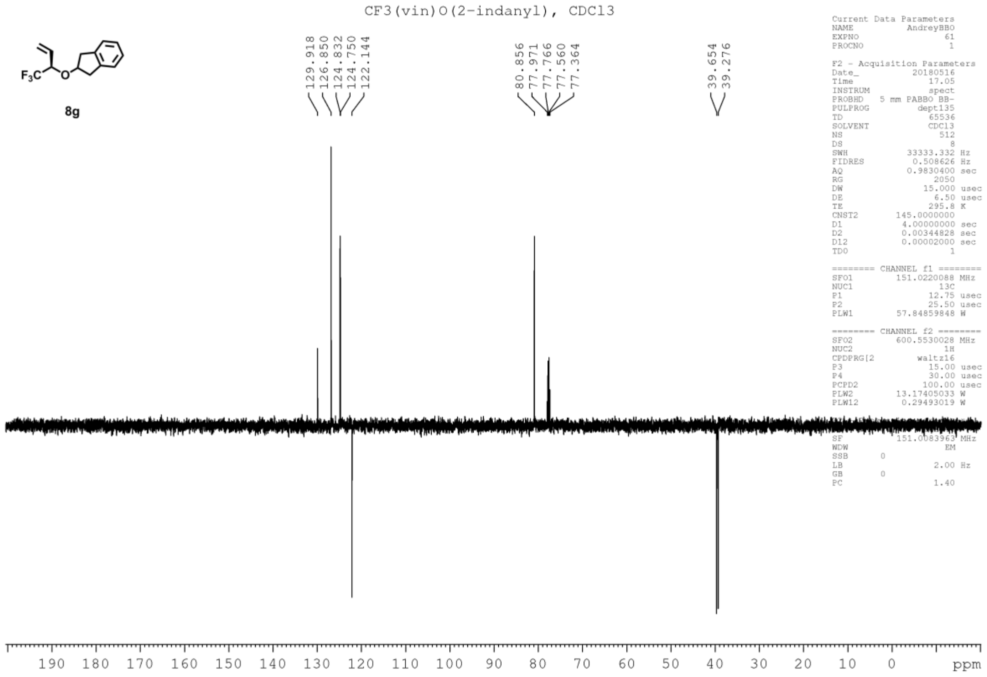

Supplementary figure 144. DEPT-135 NMR spectra of **8g** in $CDCl_3$

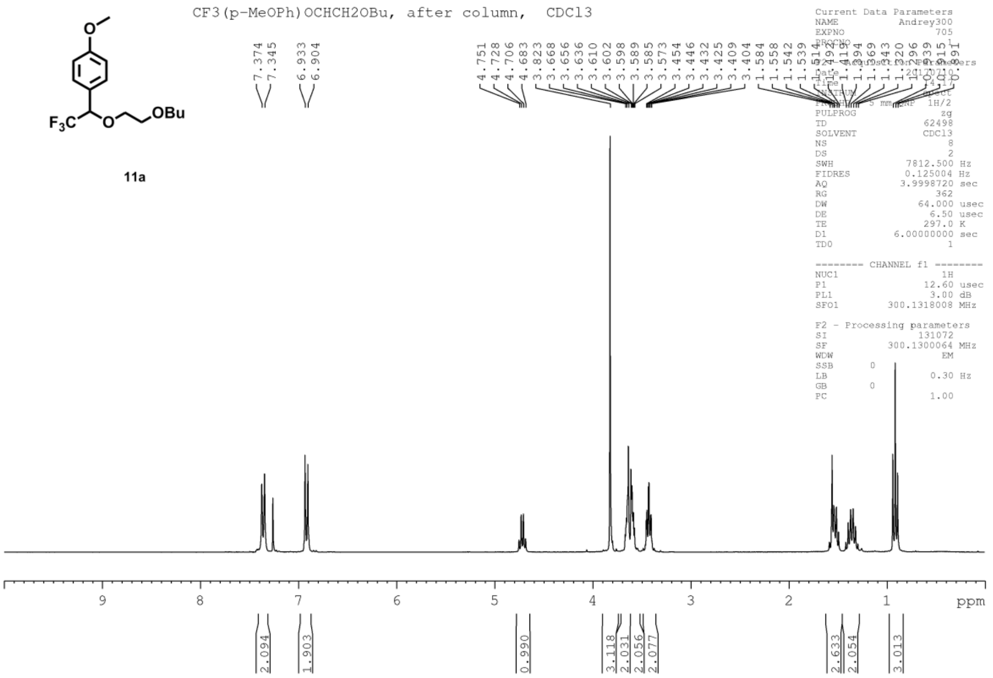

Supplementary figure 145. ^{1}H NMR spectra of **11a** in $CDCl_3$

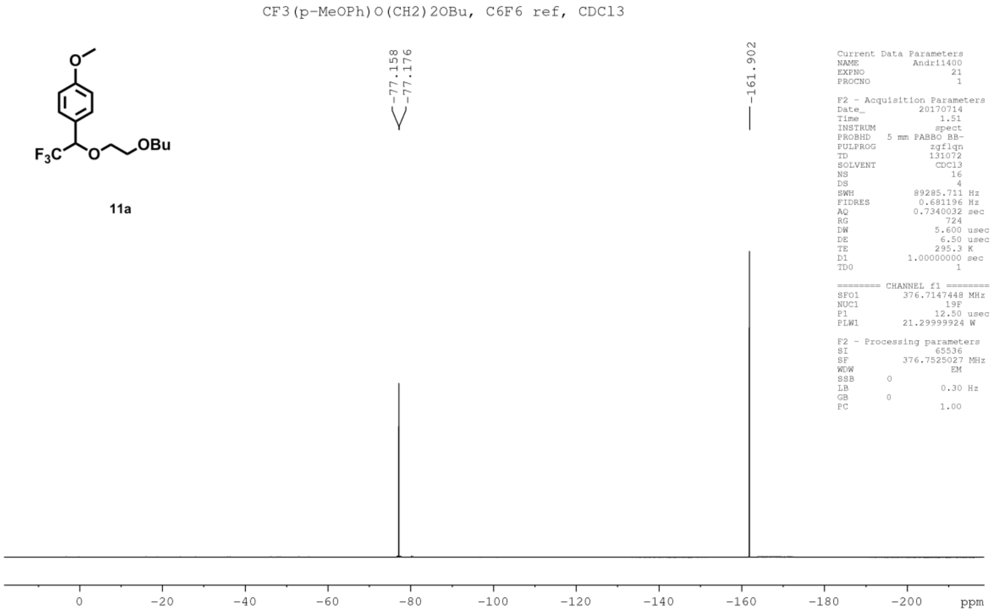

Supplementary figure 146. ^{19}F NMR spectra of **11a** in $CDCl_3$

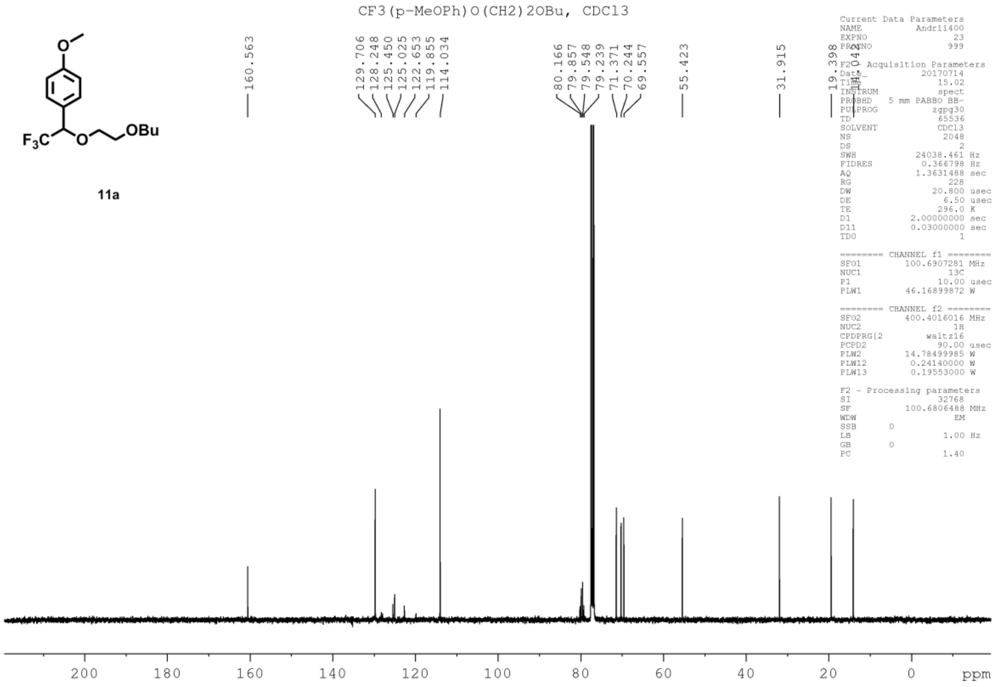

Supplementary figure 147. ^{13}C NMR spectra of **11a** in $CDCl_3$

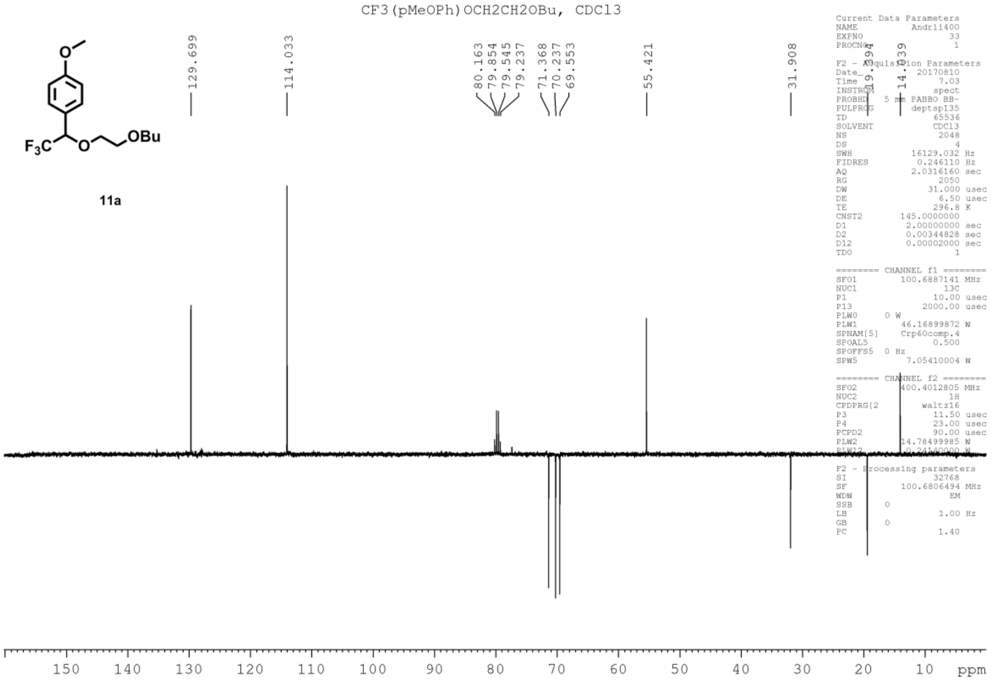

Supplementary figure 148. DEPT-135 NMR spectra of **11a** in $CDCl_3$

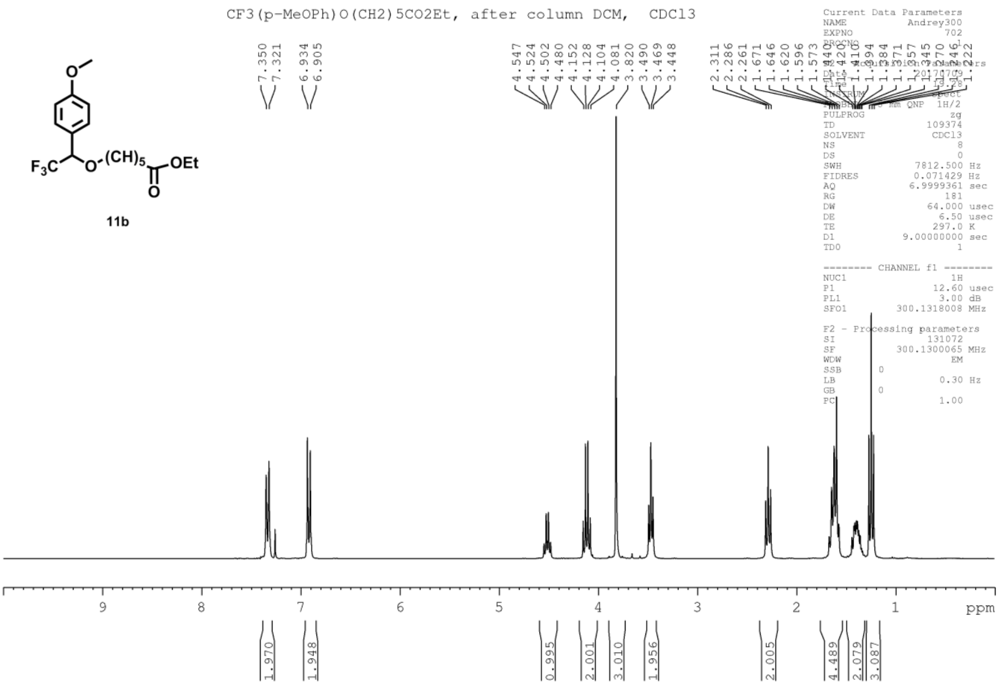

Supplementary figure 149. ^{1}H NMR spectra of **11b** in $CDCl_3$

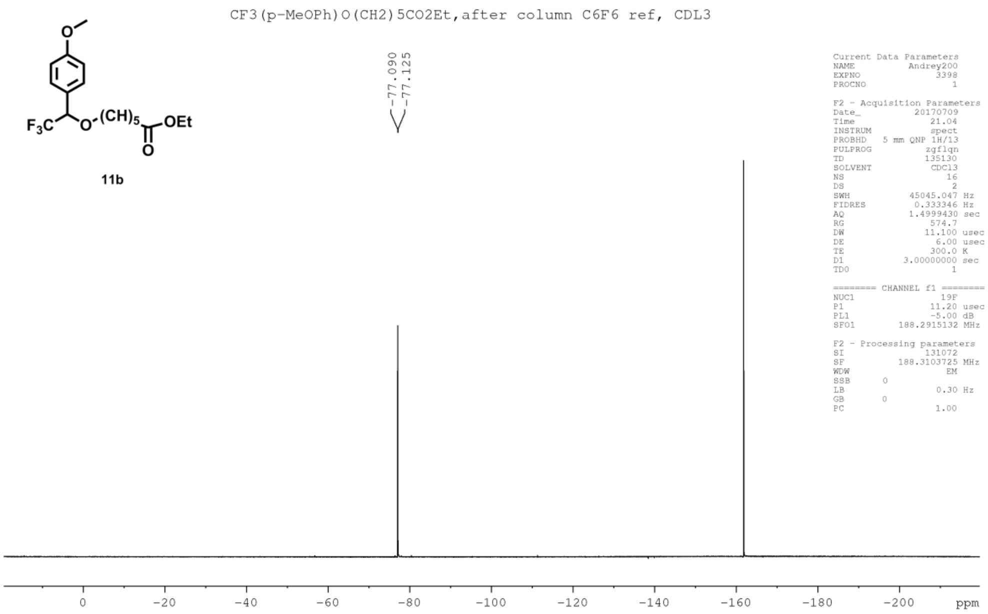

Supplementary figure 150. ^{19}F NMR spectra of **11b** in $CDCl_3$

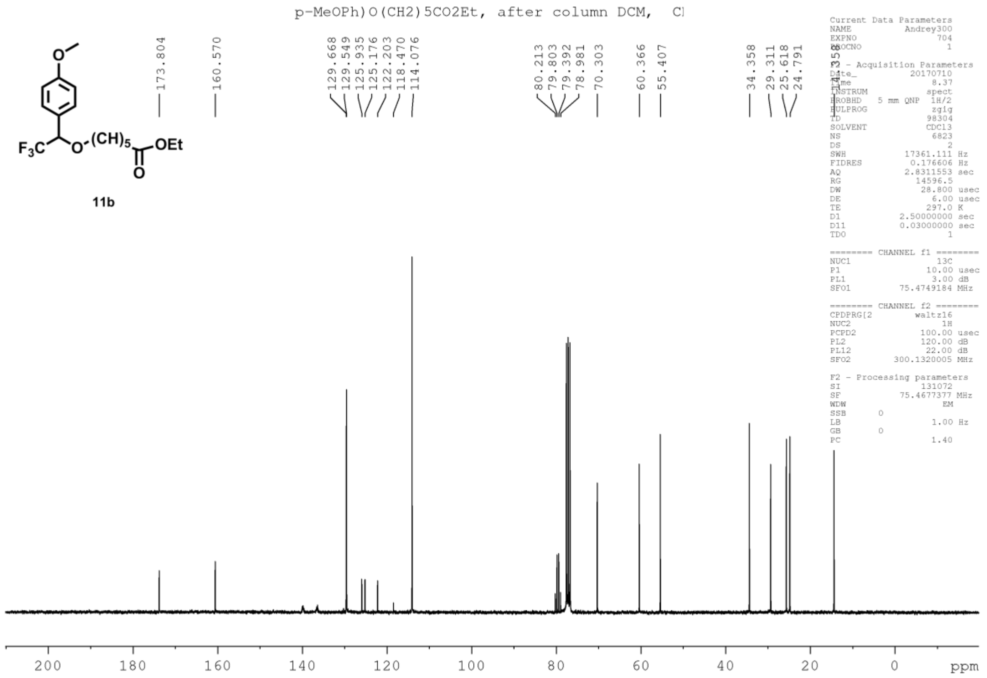

Supplementary figure 151. ^{13}C NMR spectra of **11b** in $CDCl_3$

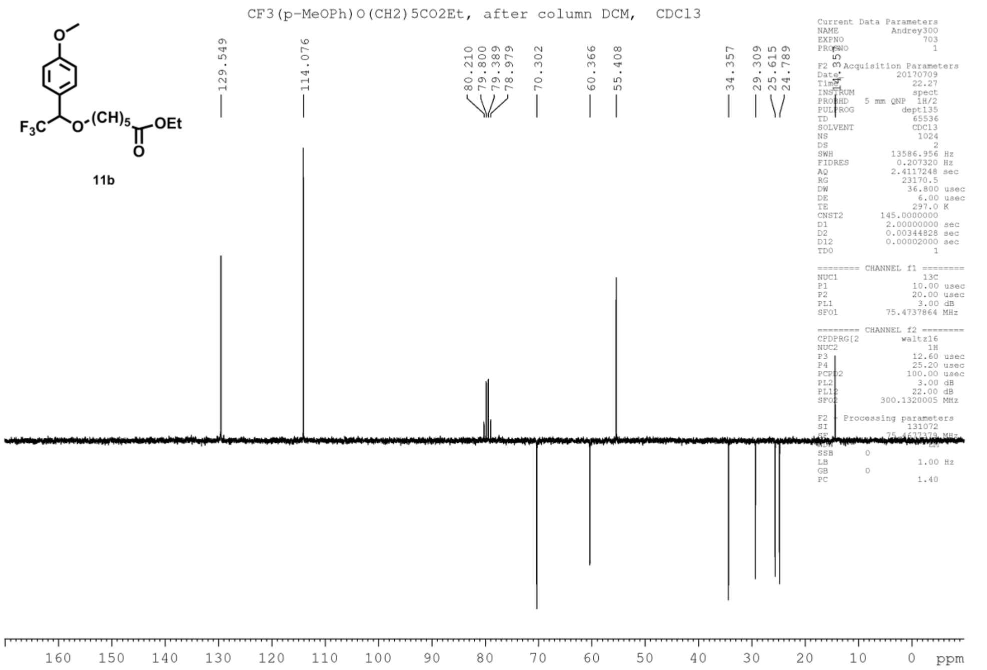

Supplementary figure 152. DEPT-135 NMR spectra of **11b** in $CDCl_3$

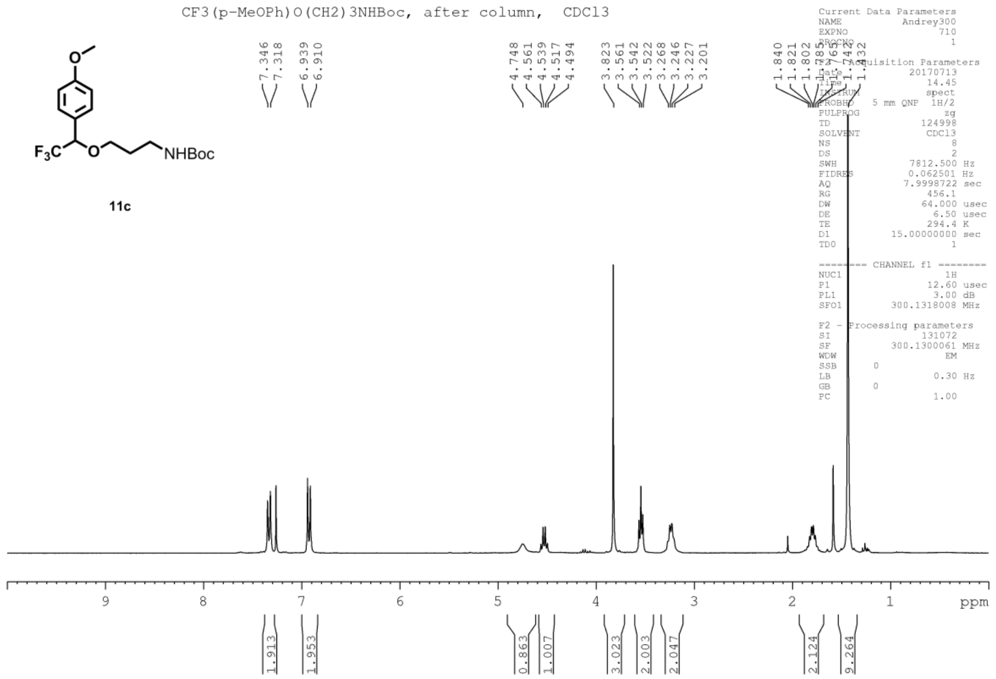

Supplementary figure 153. ^{1}H NMR spectra of **11c** in $CDCl_3$

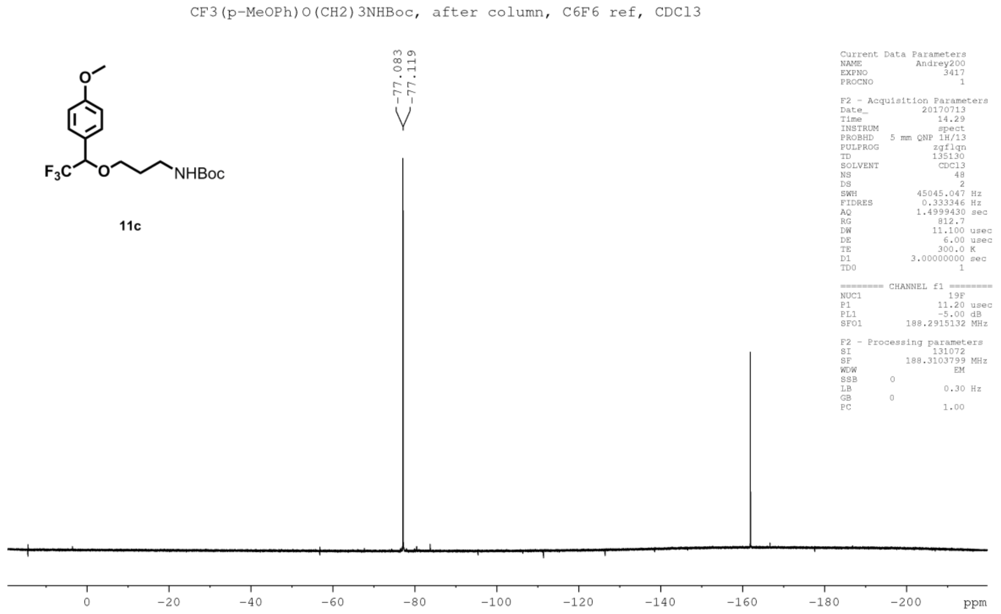

Supplementary figure 154. ^{19}F NMR spectra of **11c** in $CDCl_3$

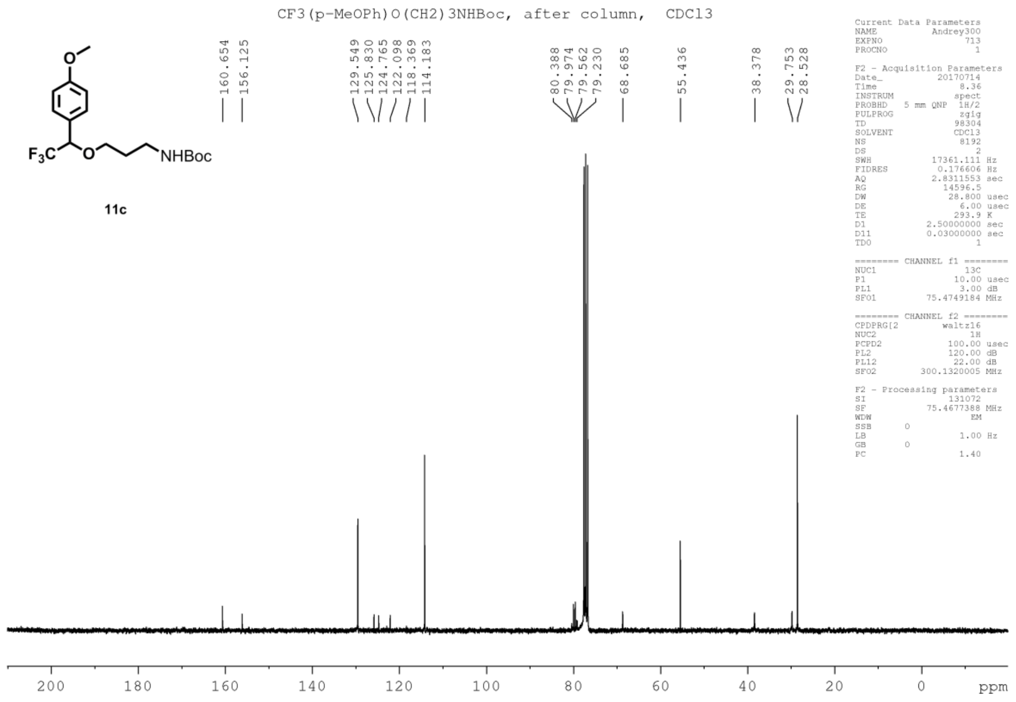

Supplementary figure 155. ^{13}C NMR spectra of **11c** in $CDCl_3$

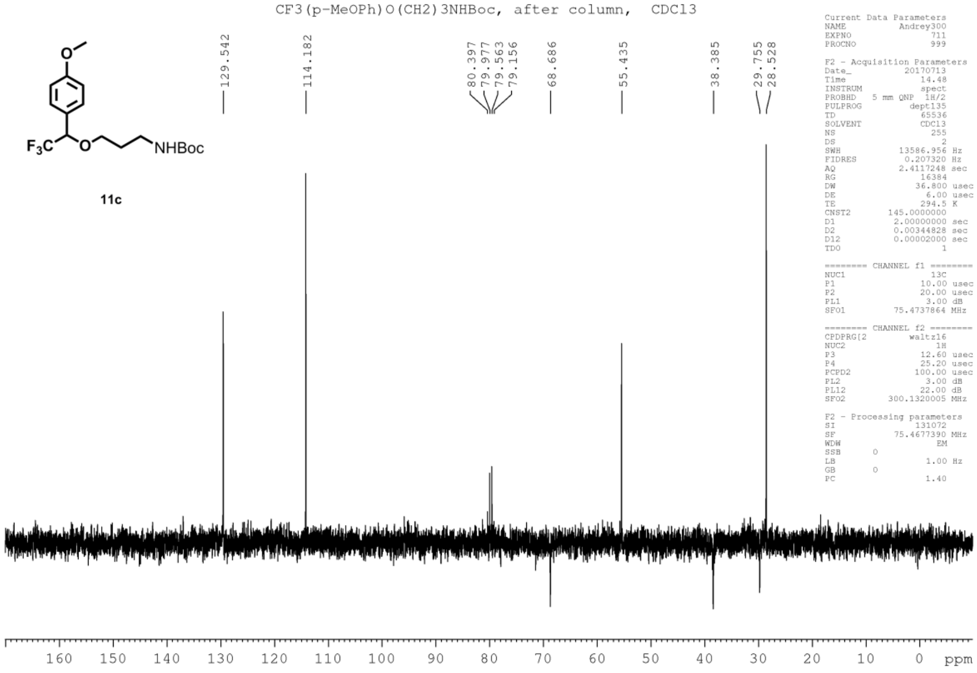

Supplementary figure 156. DEPT-135 NMR spectra of **11c** in $CDCl_3$

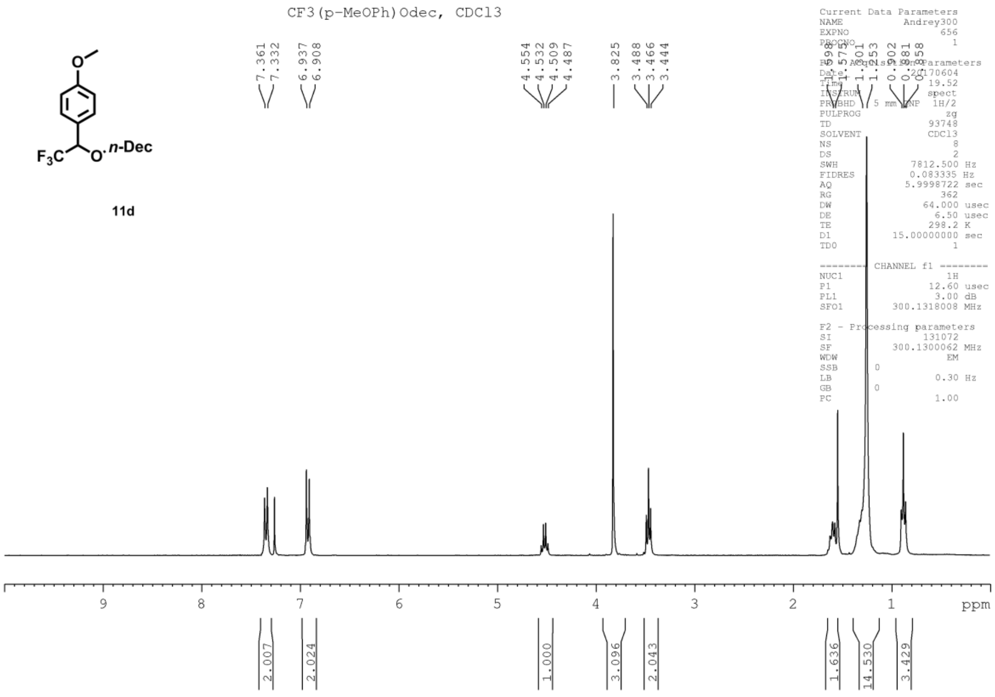

Supplementary figure 157. ^{1}H NMR spectra of **11d** in $CDCl_3$

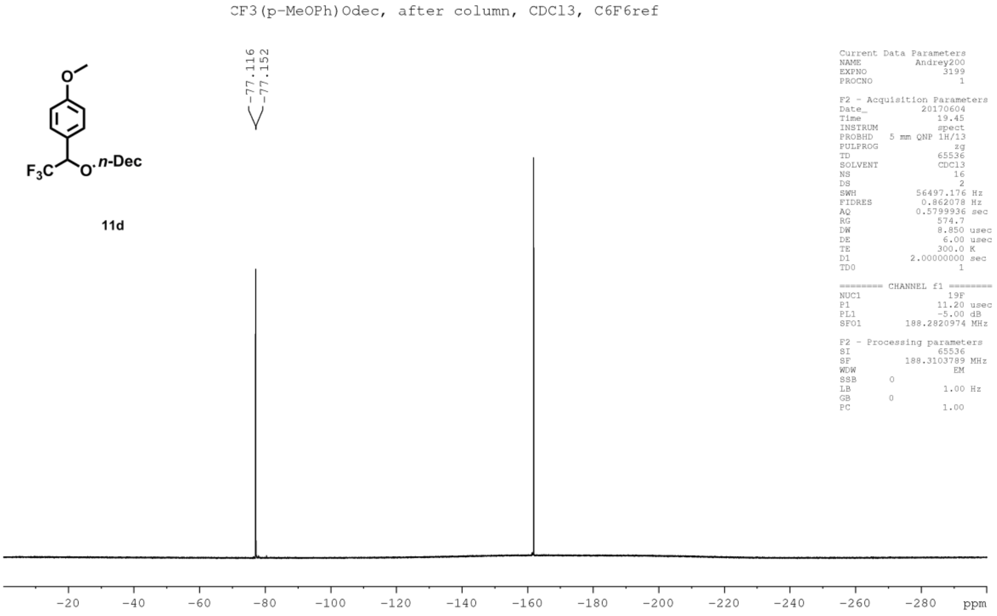

Supplementary figure 158. ^{19}F NMR spectra of **11d** in $CDCl_3$

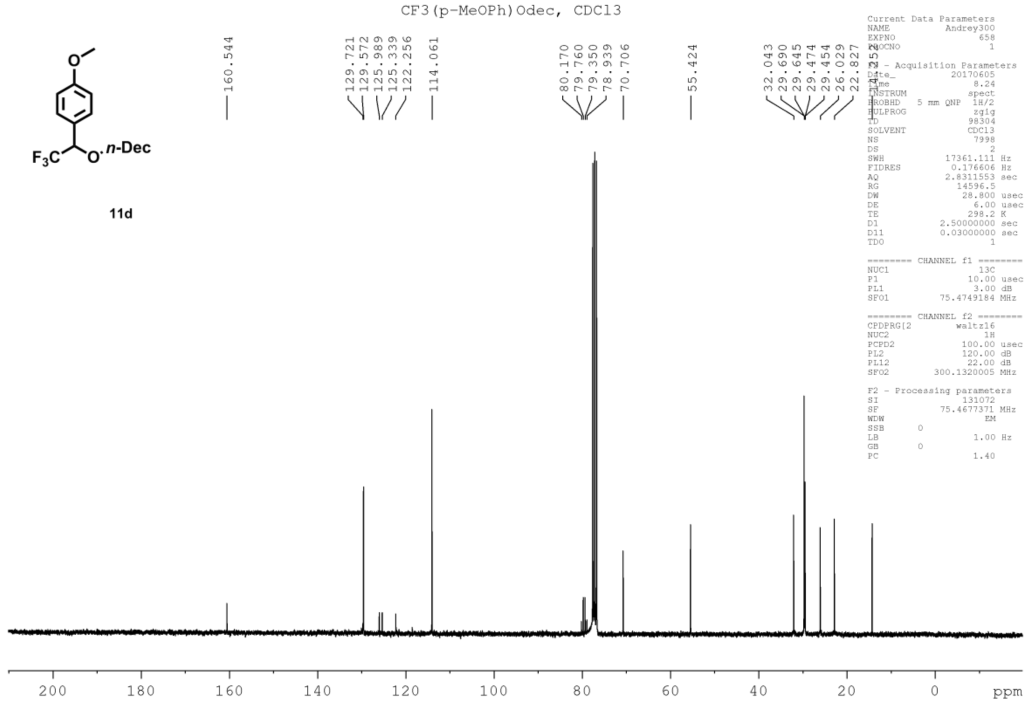

Supplementary figure 159. ^{13}C NMR spectra of **11d** in $CDCl_3$

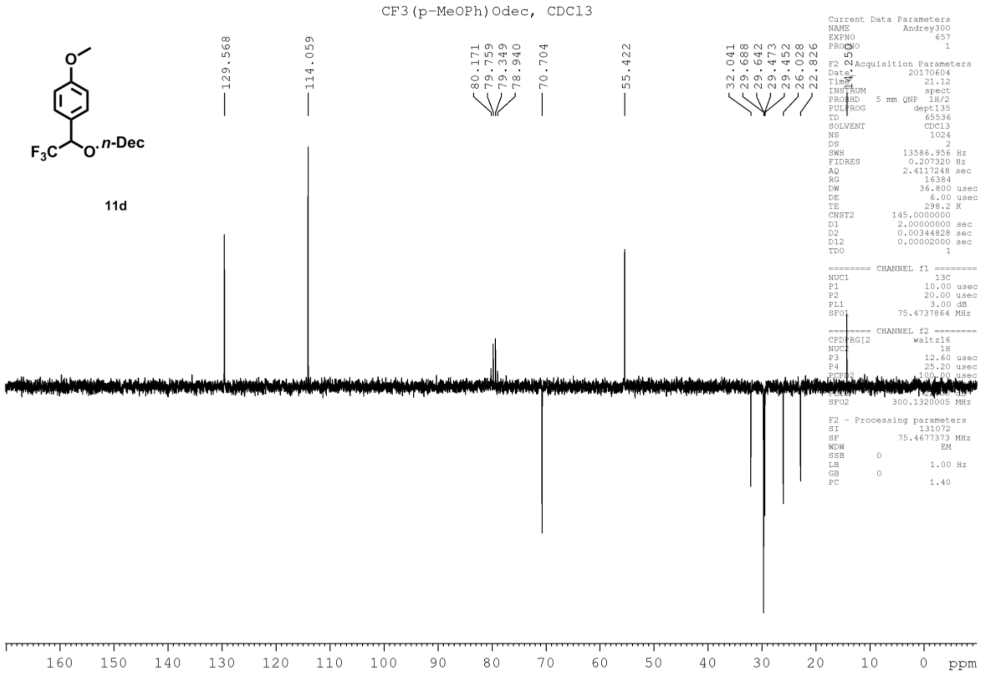

Supplementary figure 160. DEPT-135 NMR spectra of **11d** in $CDCl_3$

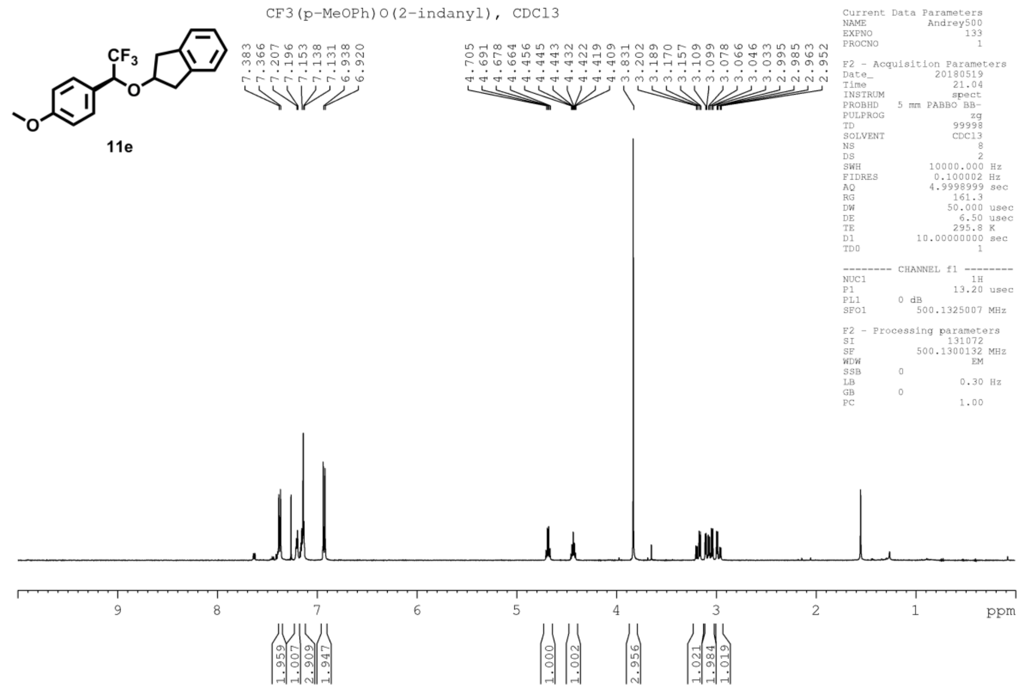

Supplementary figure 161. ^{1}H NMR spectra of **11e** in $CDCl_3$

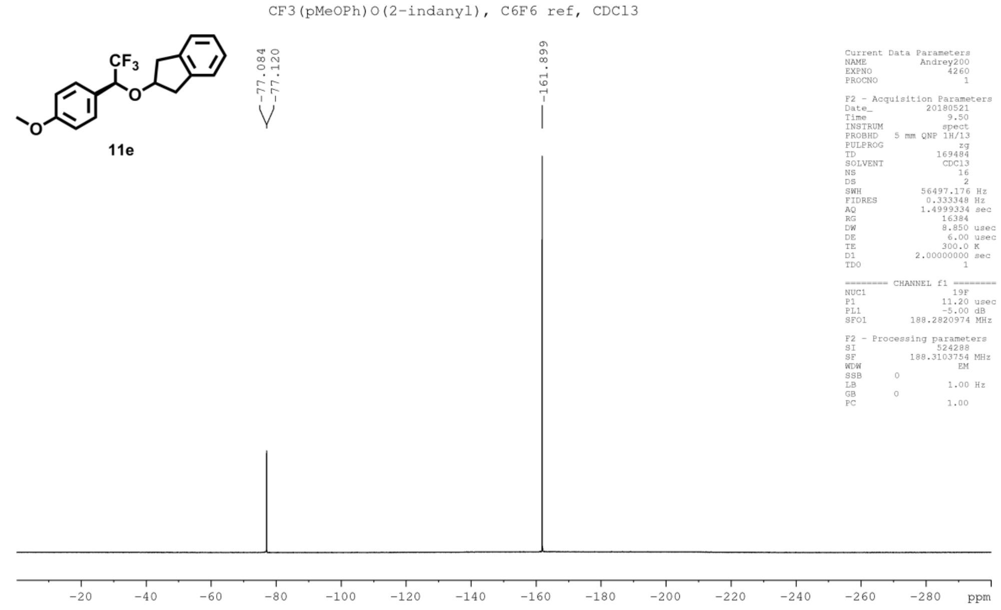

Supplementary figure 162. ^{19}F NMR spectra of **11e** in $CDCl_3$

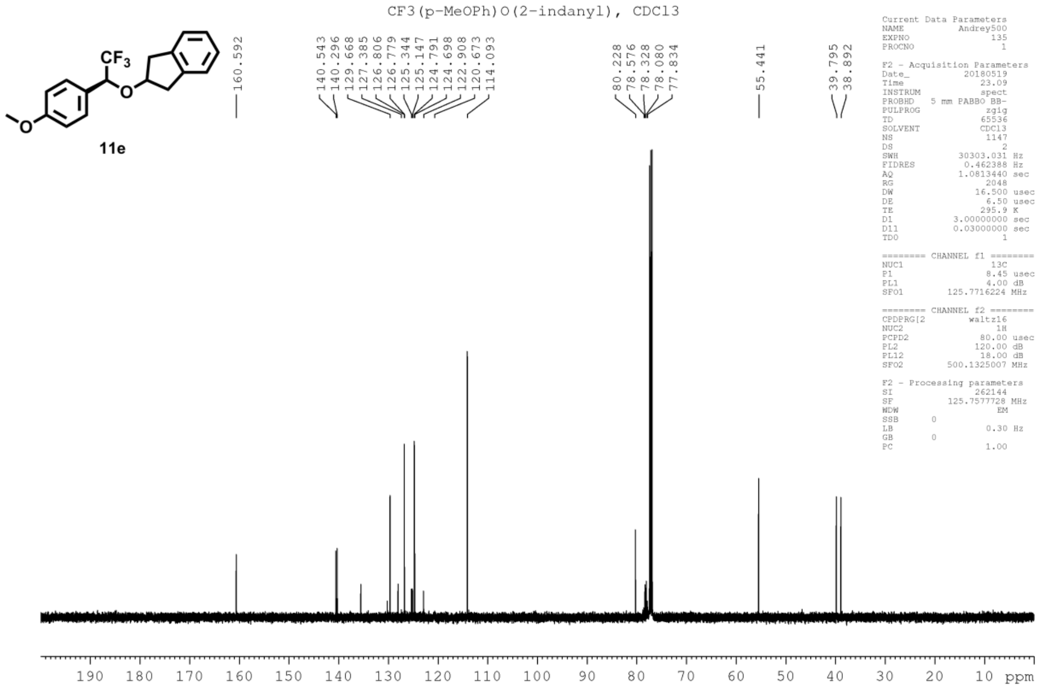

Supplementary figure 163. ^{13}C NMR spectra of **11e** in $CDCl_3$

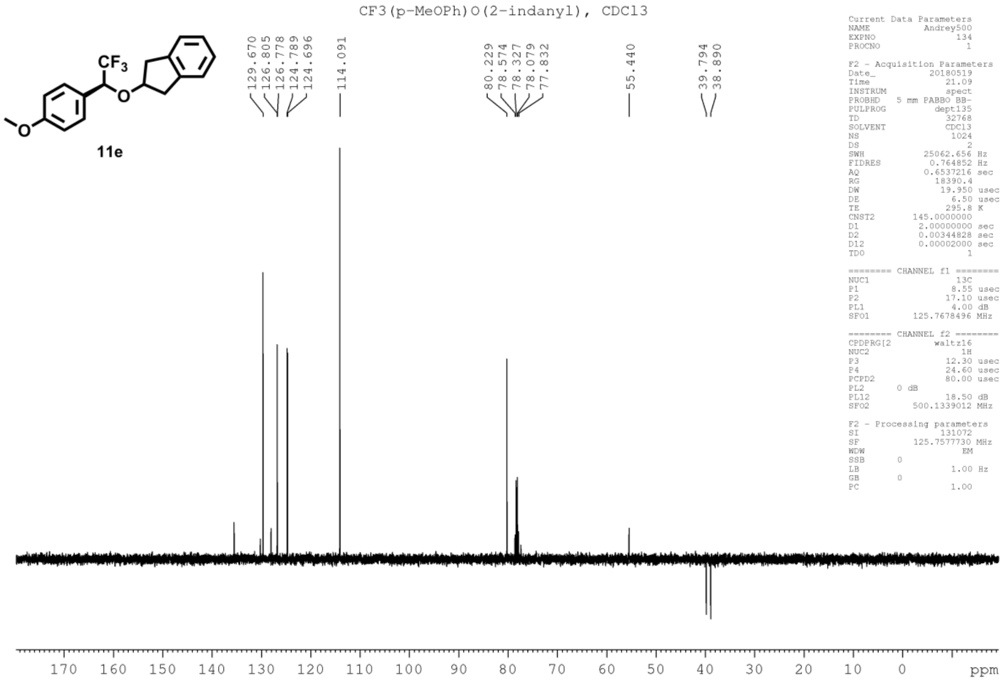

Supplementary figure 164. DEPT-135 NMR spectra of **11e** in $CDCl_3$

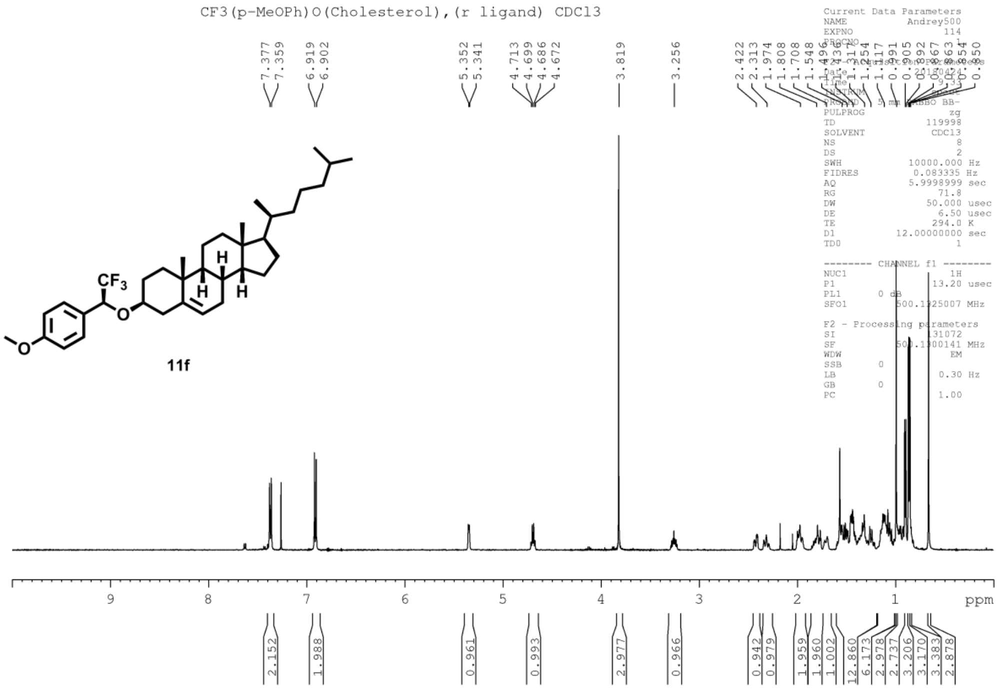

Supplementary figure 165. ^{1}H NMR spectra of (S)-**11f** in $CDCl_3$

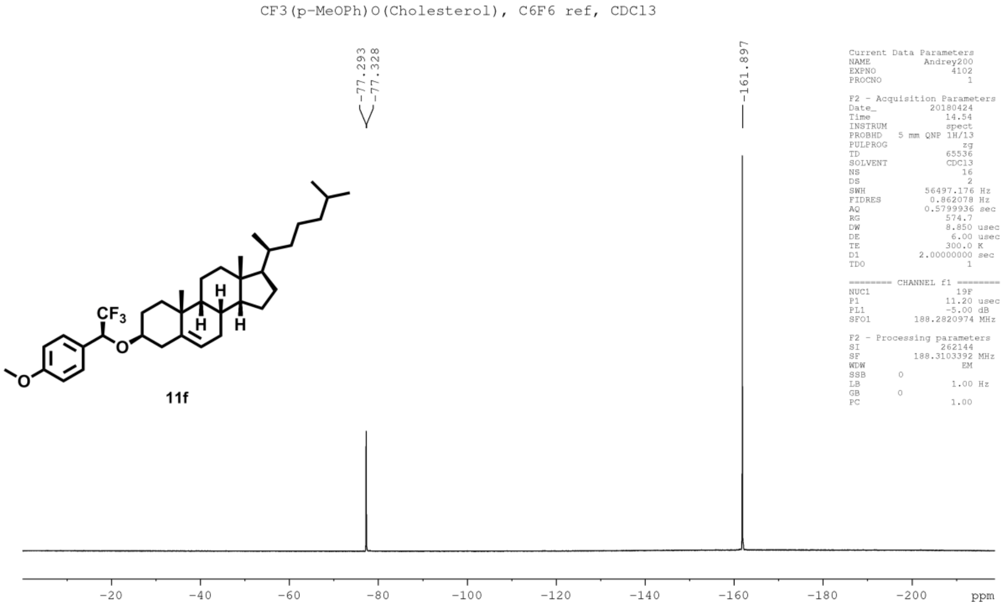

Supplementary figure 166. ^{19}F NMR spectra of (S)-**11f** in $CDCl_3$

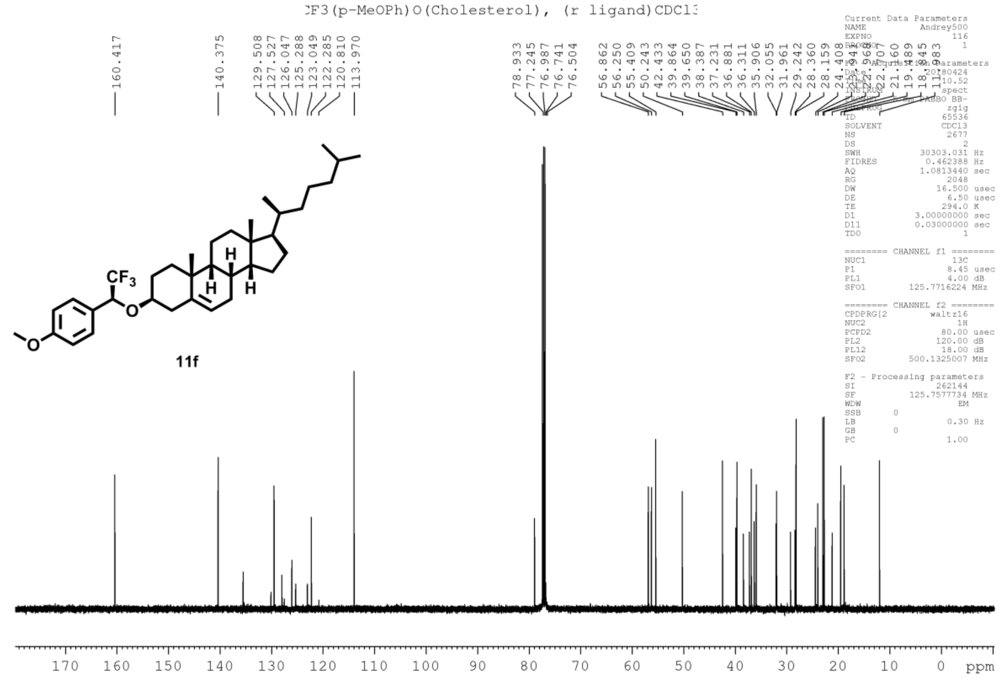

Supplementary figure 167. ^{13}C NMR spectra of (S)-**11f** in $CDCl_3$

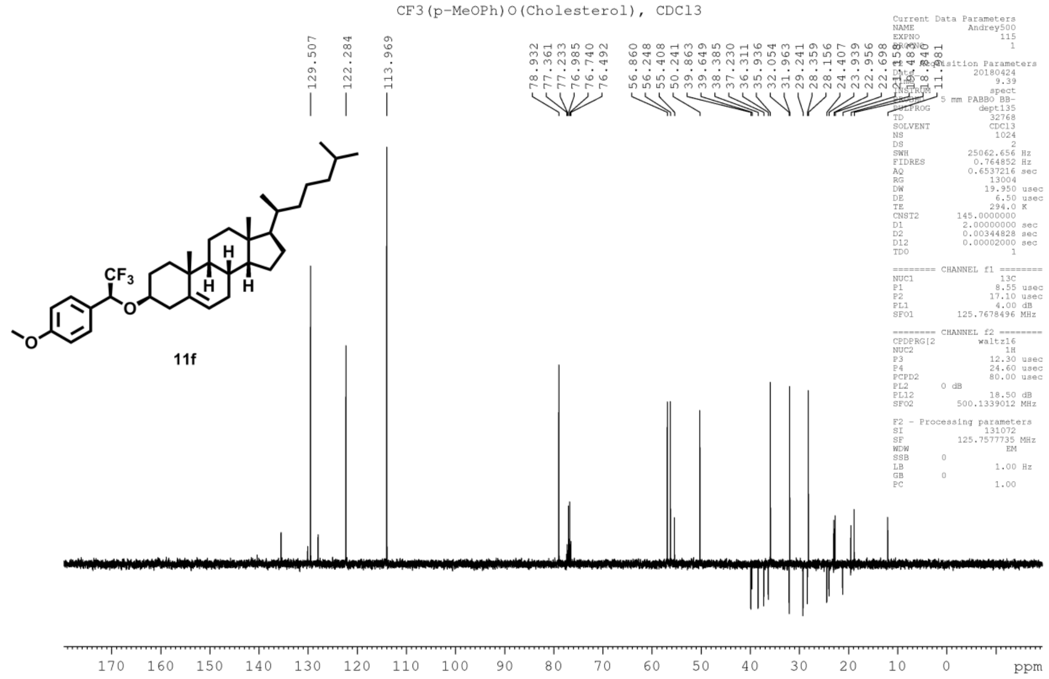

Supplementary figure 168. DEPR-135 NMR spectra of (S)-**11f** in $CDCl_3$

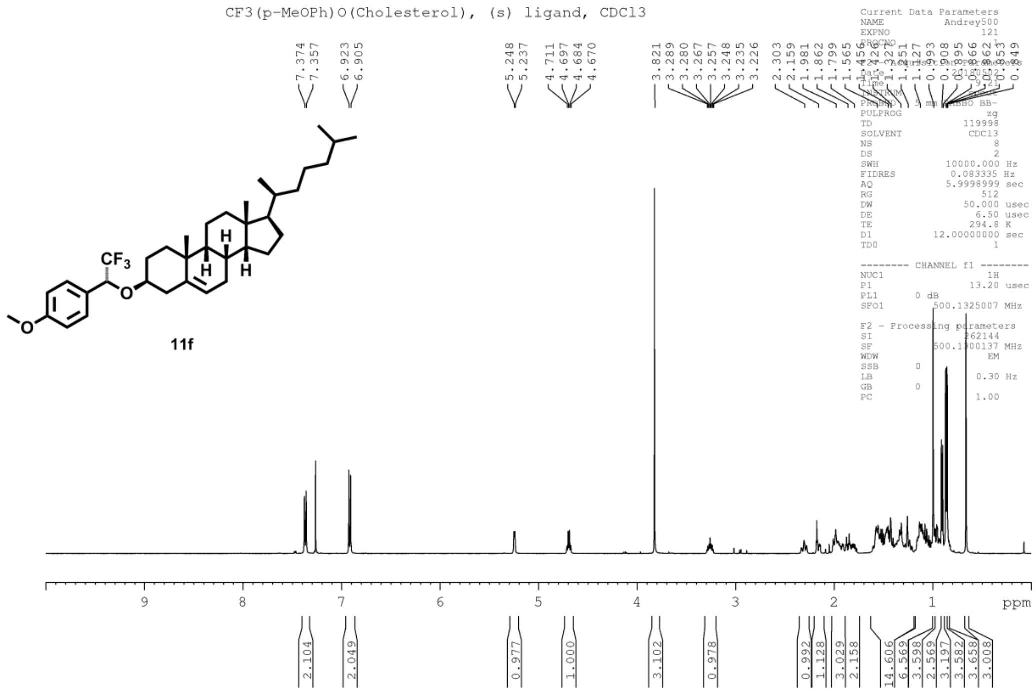

Supplementary figure 169. ^{1}H NMR spectra of (R)-**11f** in $CDCl_3$

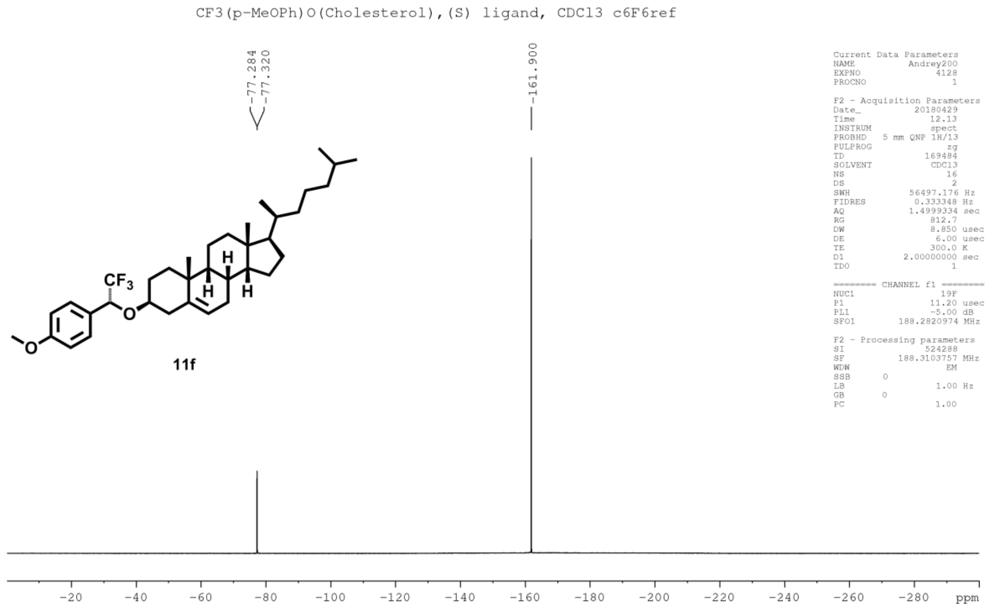

Supplementary figure 170. ^{19}F NMR spectra of (R)-**11f** in $CDCl_3$

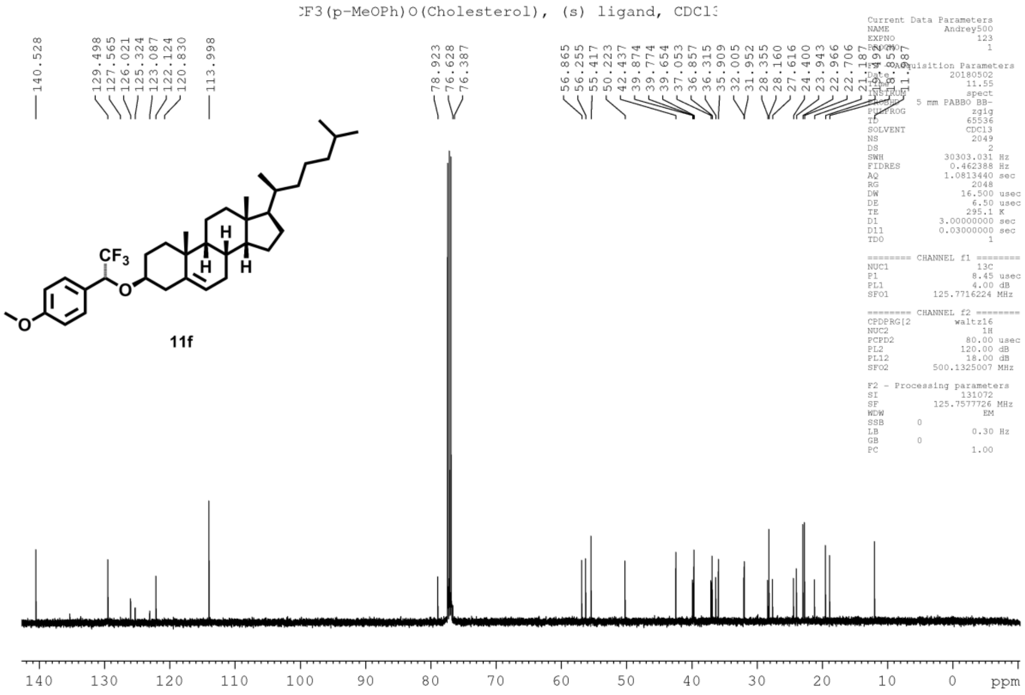

Supplementary figure 171. ^{13}C NMR spectra of (R)-**11f** in $CDCl_3$

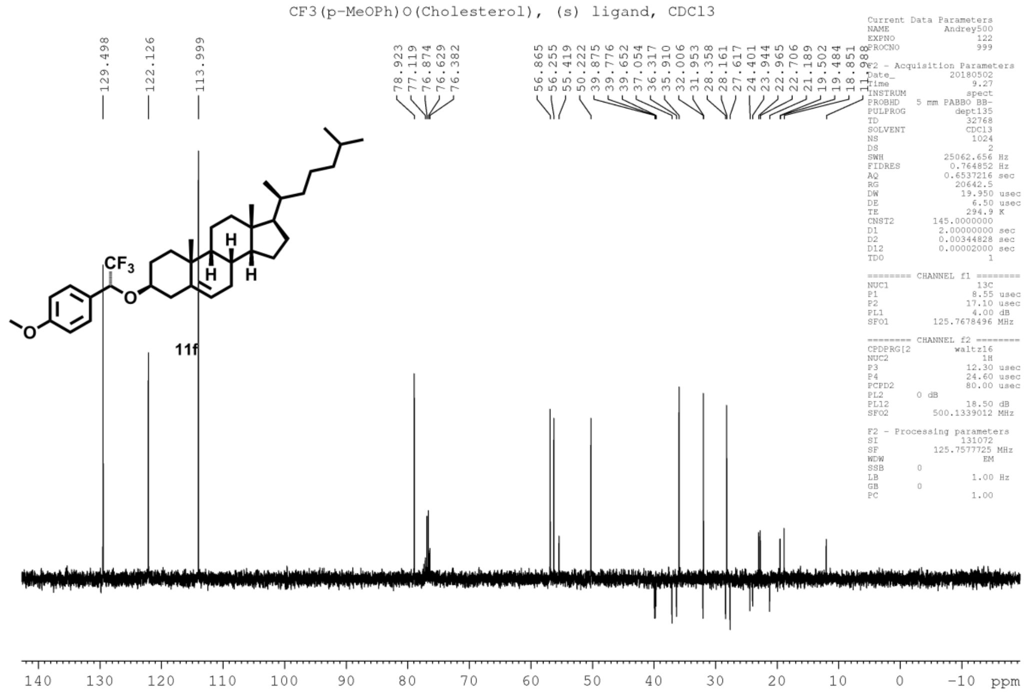

Supplementary figure 172. DEPT-135 NMR spectra of (R)-**11f** in $CDCl_3$

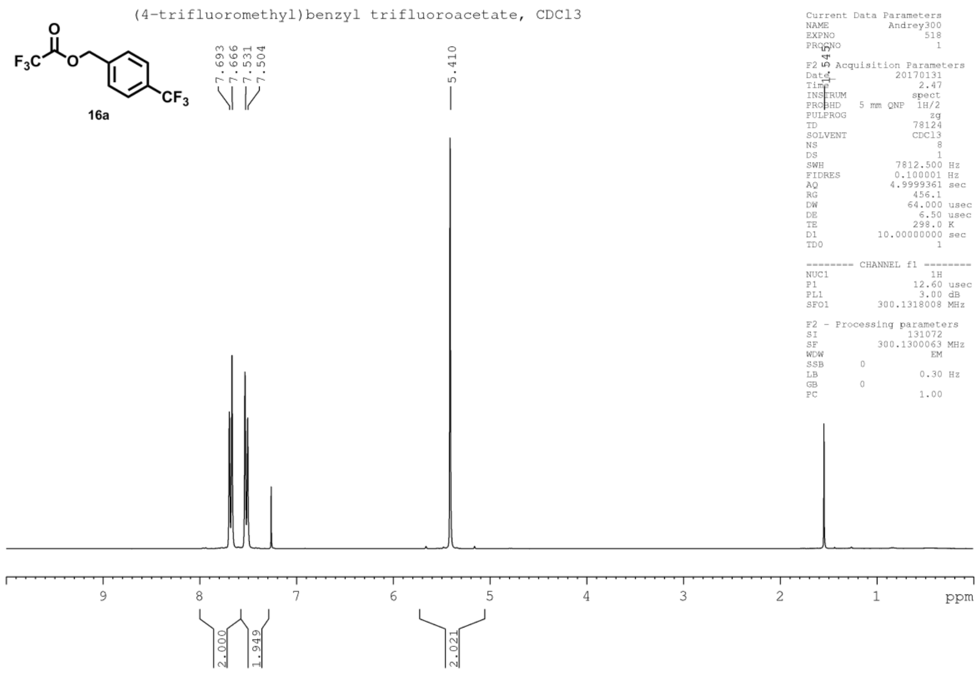

Supplementary figure 173. ^{1}H NMR spectra of **16a** in $CDCl_3$

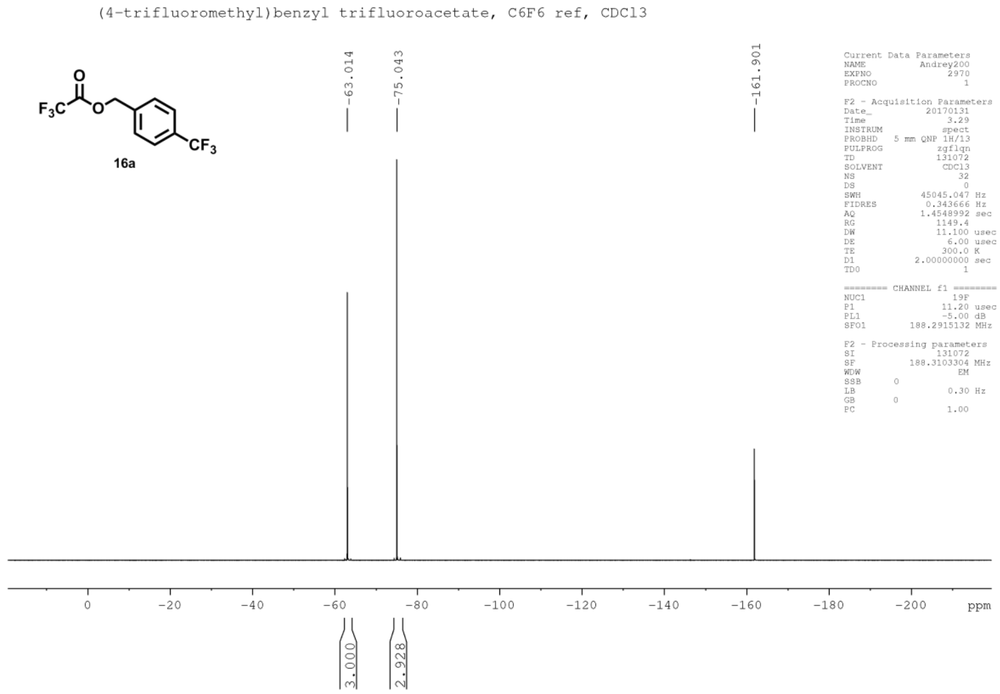

Supplementary figure 174. ^{19}F NMR spectra of **16a** in $CDCl_3$

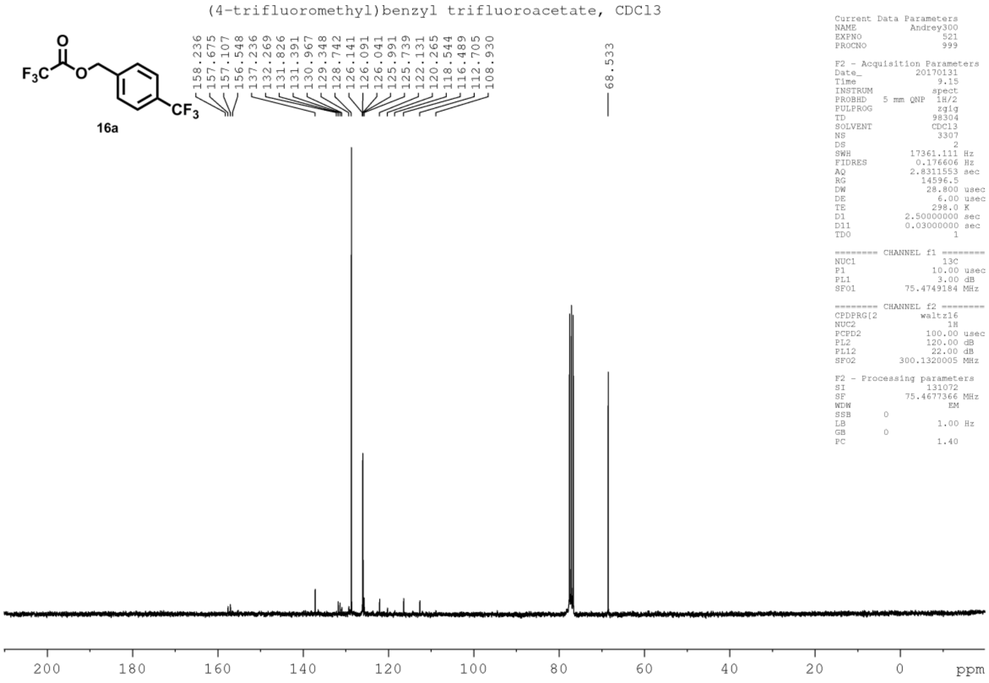

Supplementary figure 175. ^{13}C NMR spectra of **16a** in $CDCl_3$

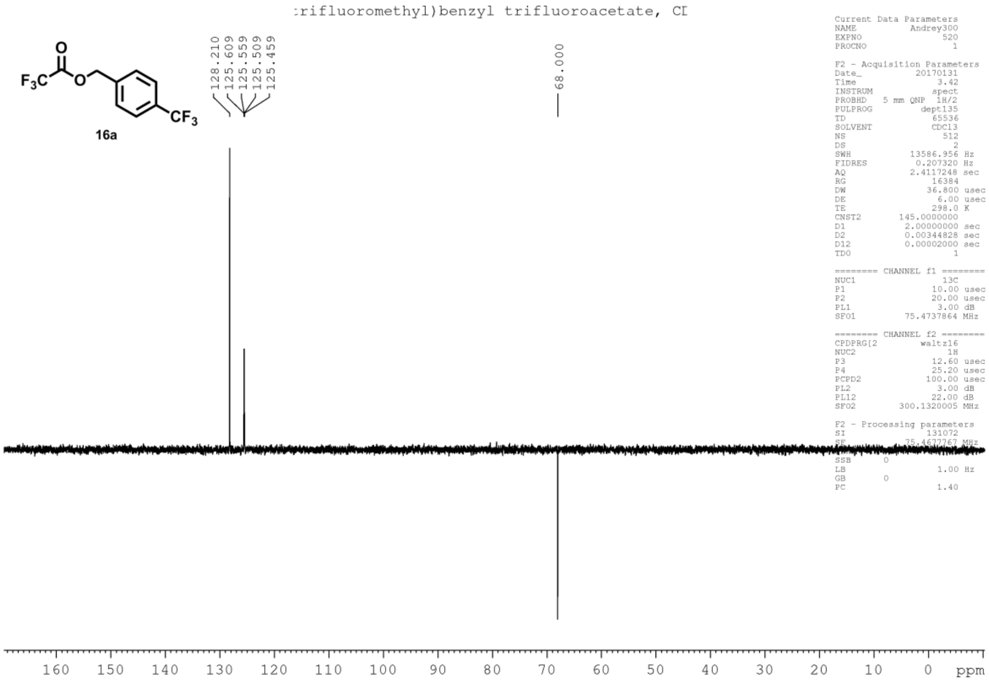

Supplementary figure 176. DEPT-135 NMR spectra of **16a** in $CDCl_3$

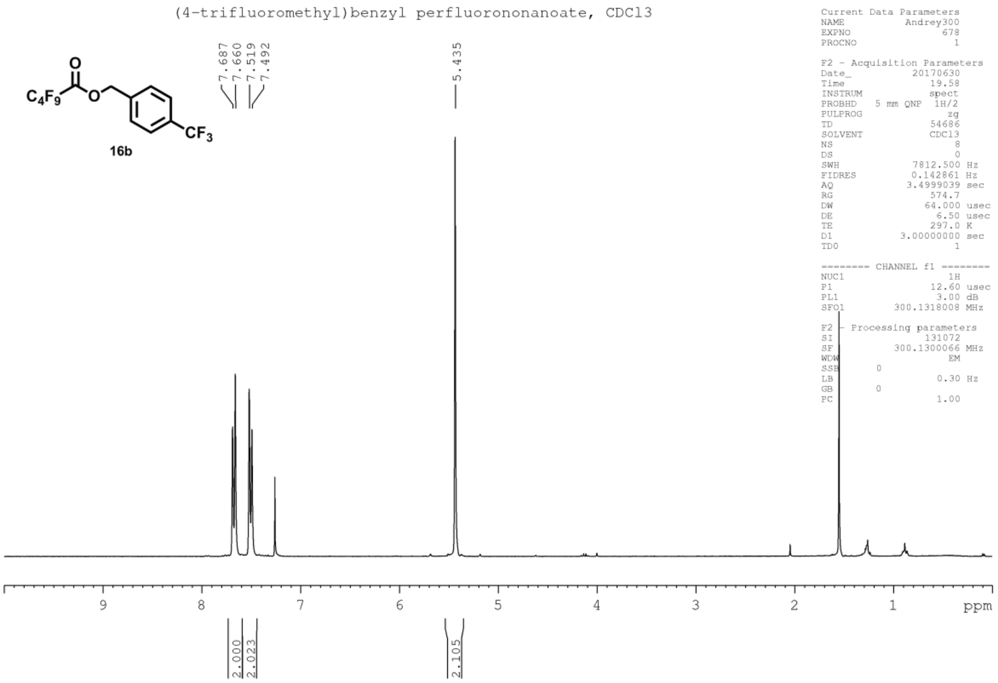

Supplementary figure 177. ^{1}H NMR spectra of **16b** in $CDCl_3$

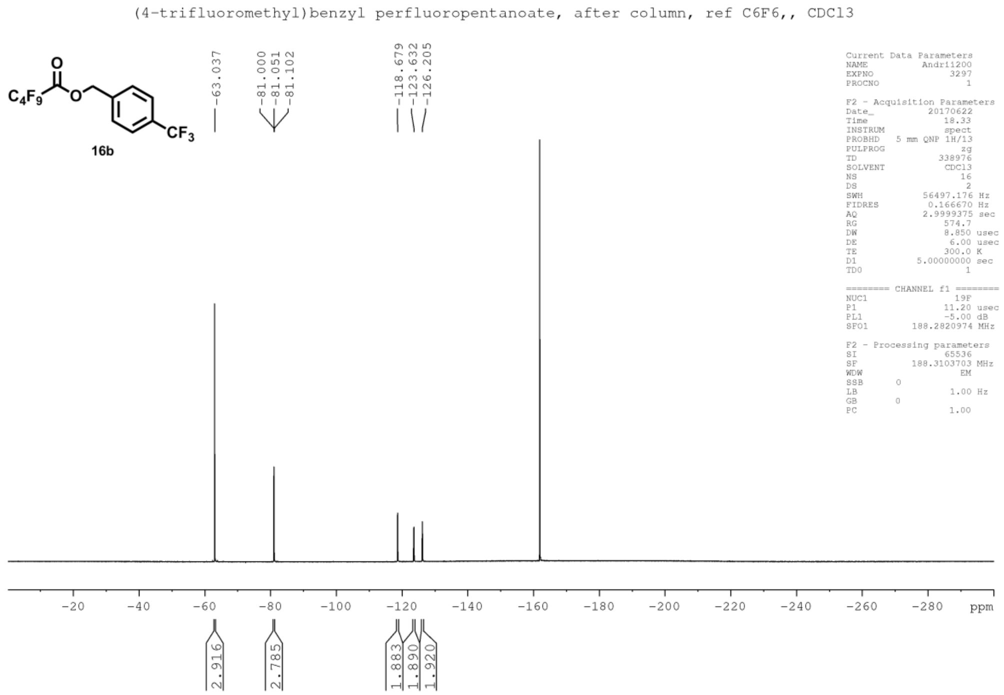

Supplementary figure 178. ^{19}F NMR spectra of **16b** in $CDCl_3$

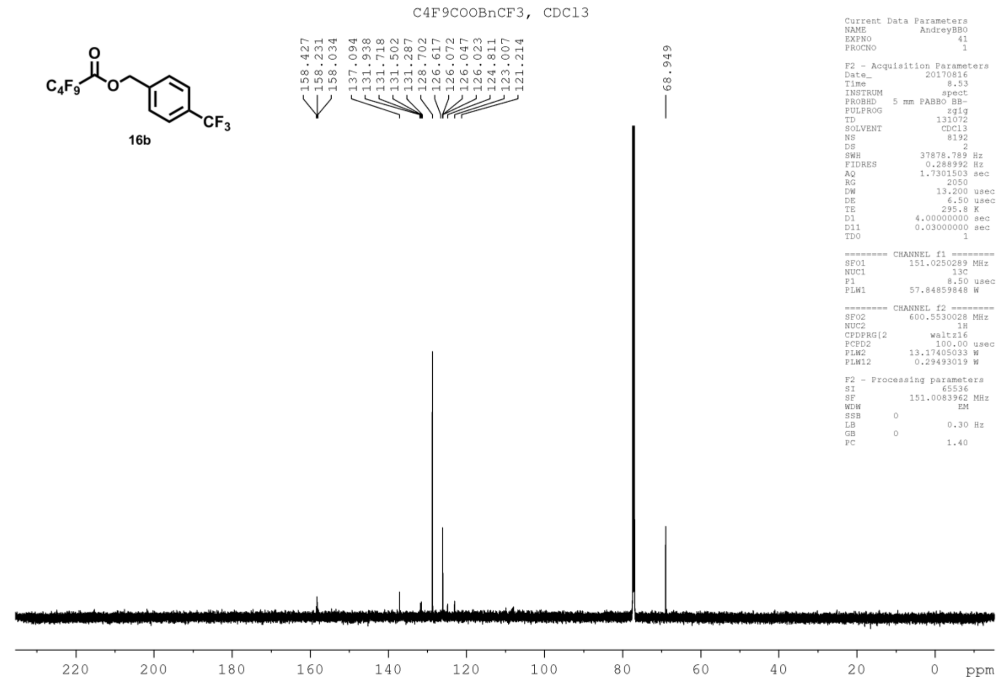

Supplementary figure 179. ^{13}C NMR spectra of **16b** in $CDCl_3$

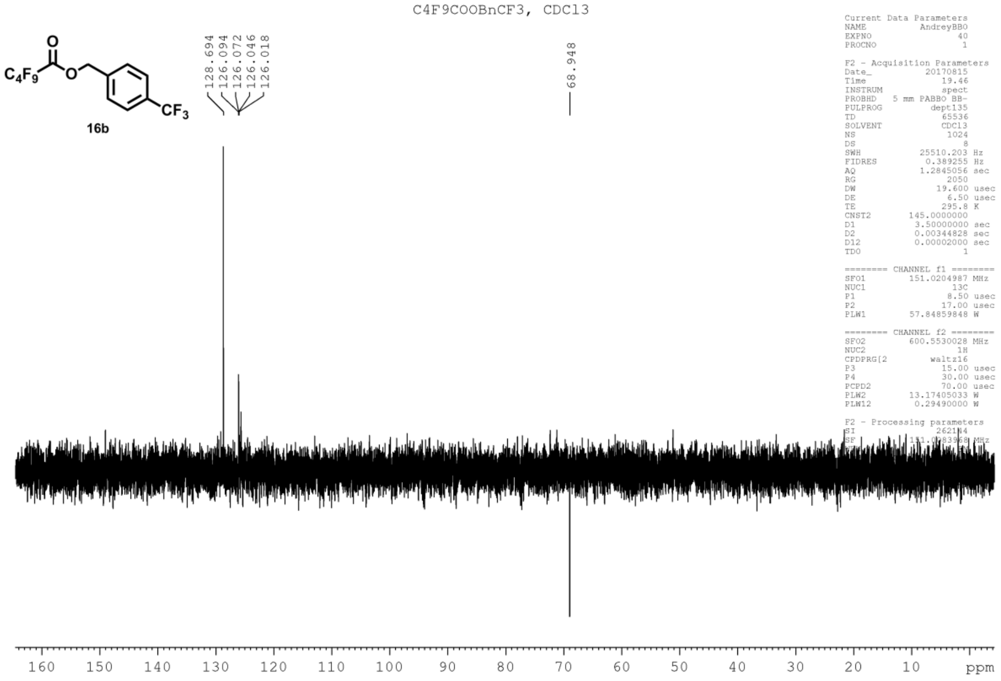

Supplementary figure 180. DEPT-135 NMR spectra of **16b** in $CDCl_3$

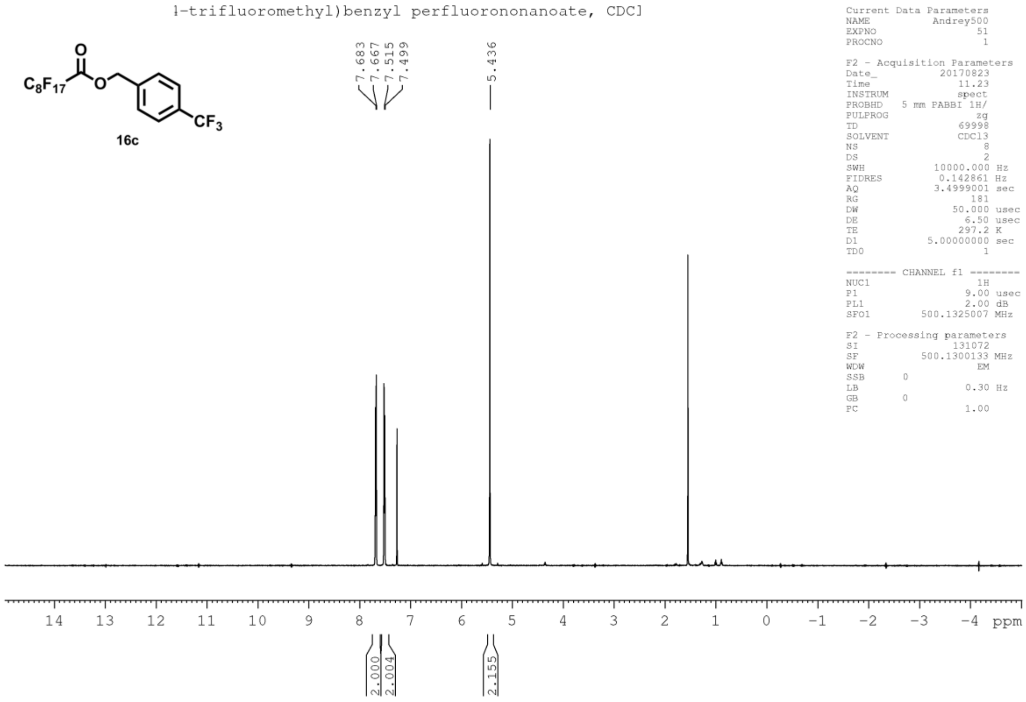

Supplementary figure 181. ^1H NMR spectra of **16c** in $CDCl_3$

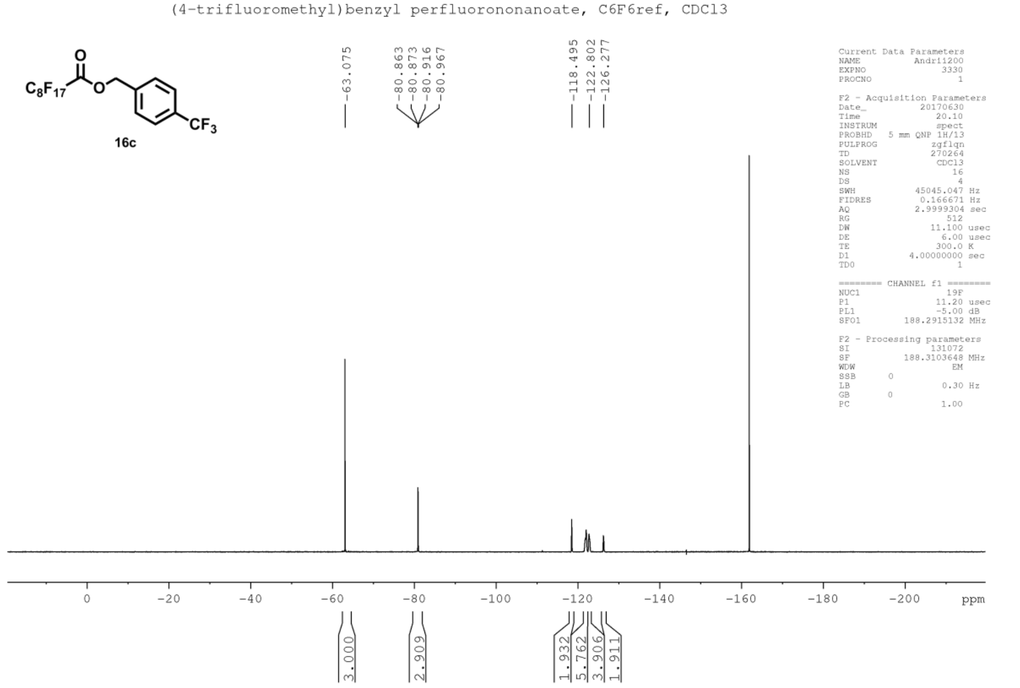

Supplementary figure 182. ^{19}F NMR spectra of **16c** in $CDCl_3$

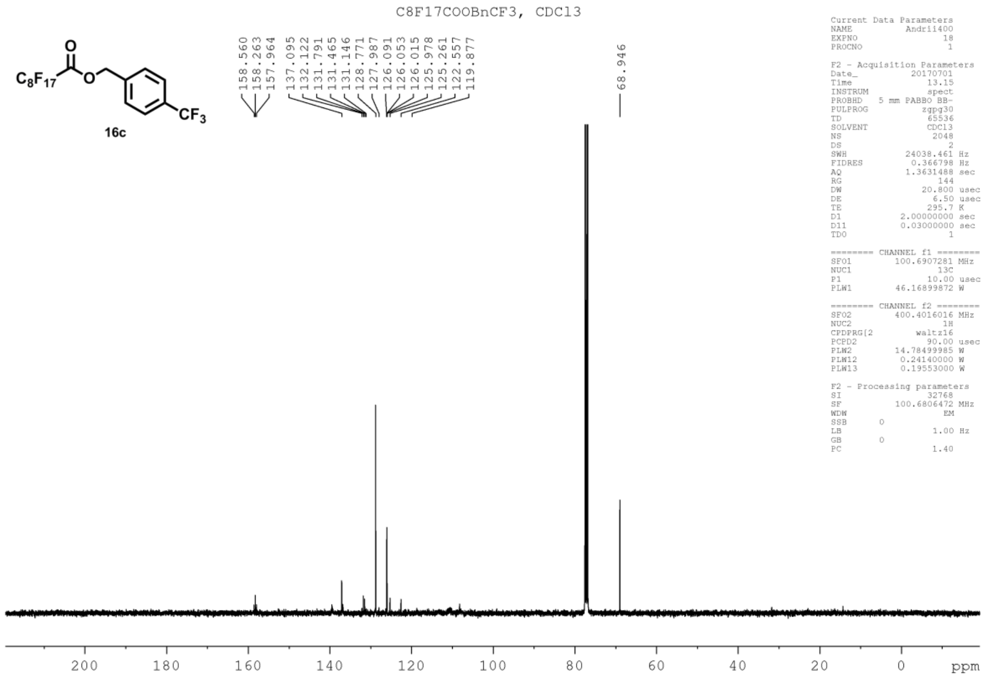

Supplementary figure 183. ^{13}C NMR spectra of **16c** in $CDCl_3$

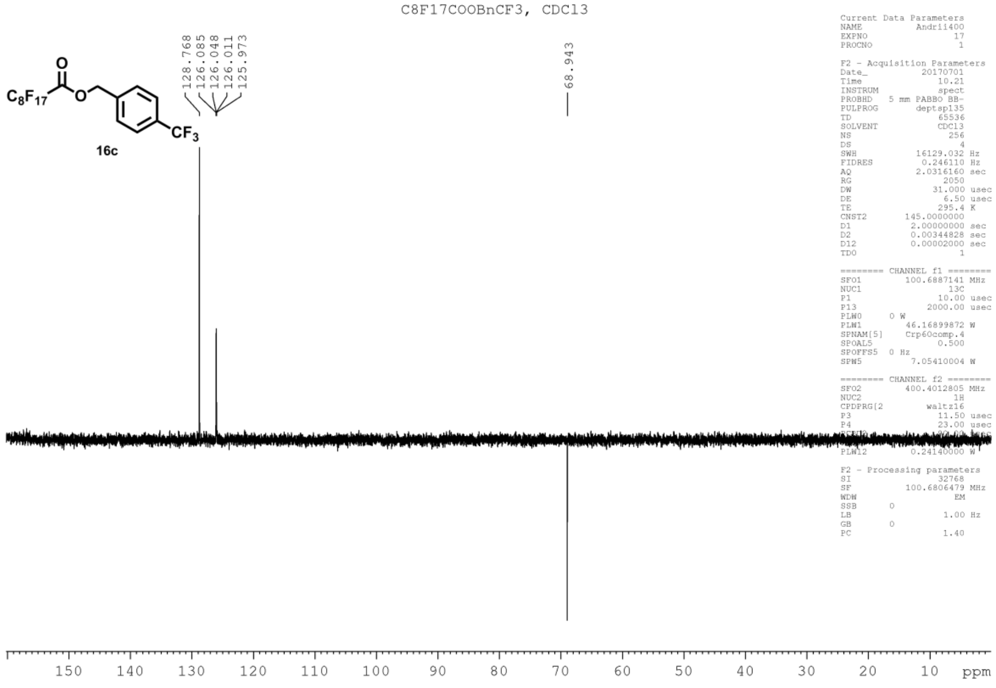

Supplementary figure 184. DEPT-135 NMR spectra of **16c** in $CDCl_3$

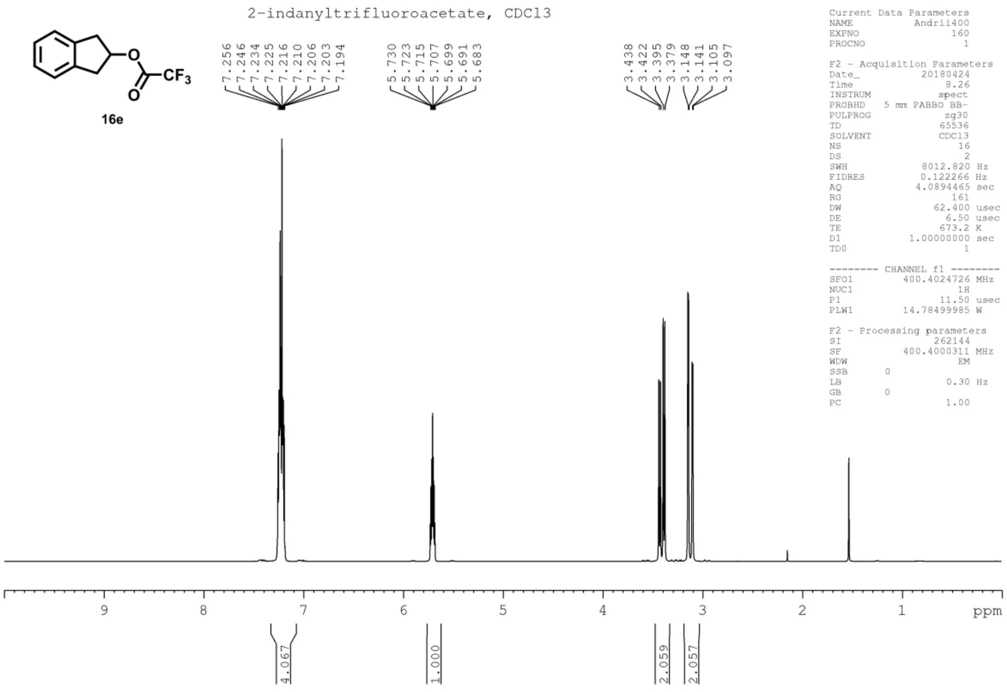

Supplementary figure 185. ^{1}H NMR spectra of **16e** in $CDCl_3$

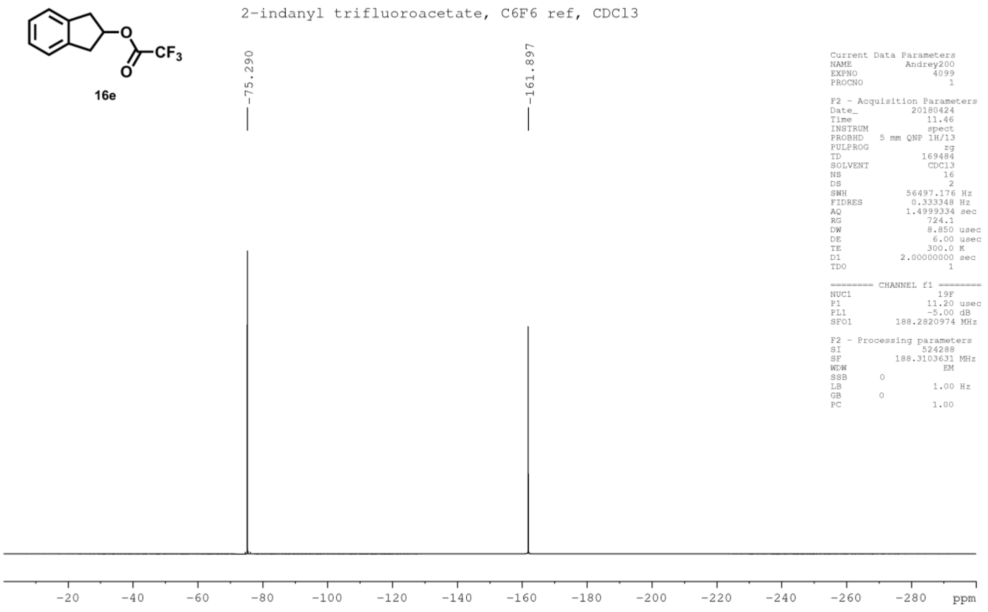

Supplementary figure 186. ^{19}F NMR spectra of **16e** in $CDCl_3$

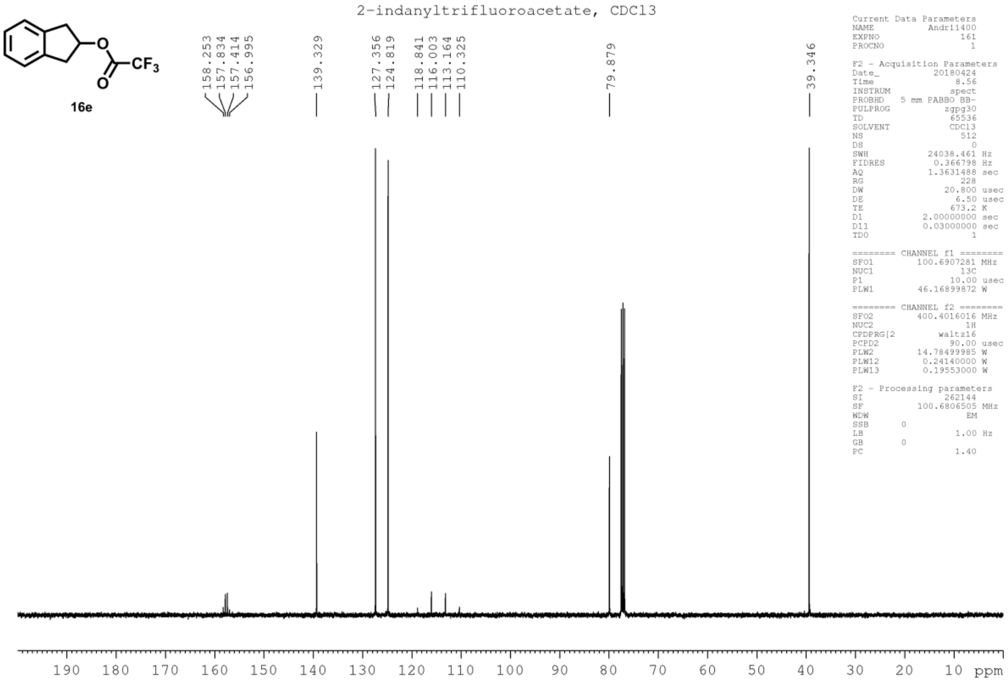

Supplementary figure 187. ^{13}C NMR spectra of **16e** in $CDCl_3$

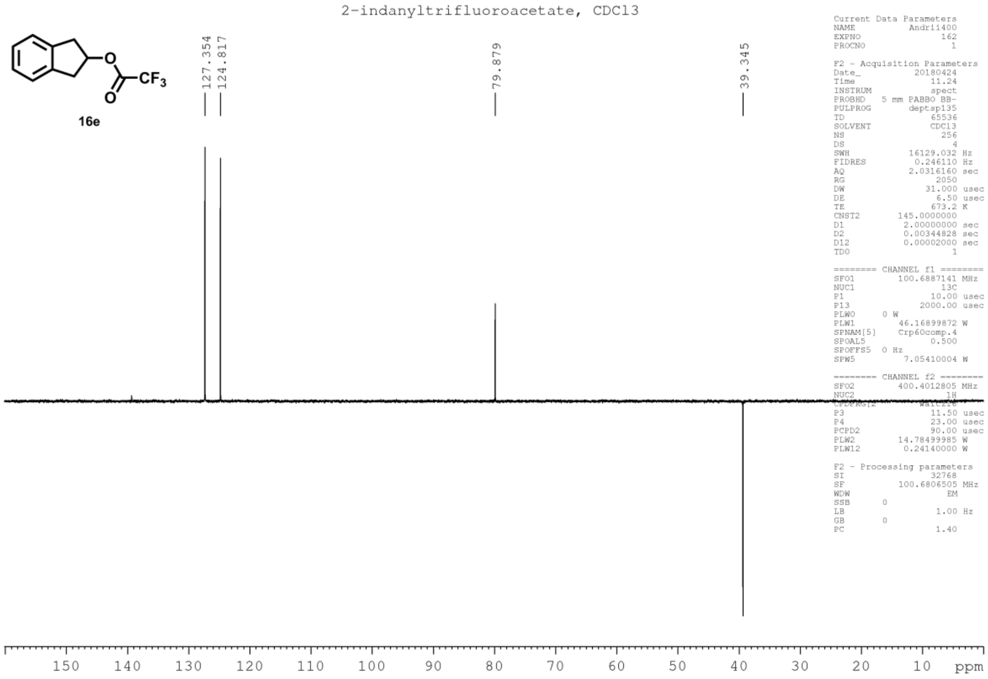

Supplementary figure 188. DEPT-135 NMR spectra of **16e** in $CDCl_3$

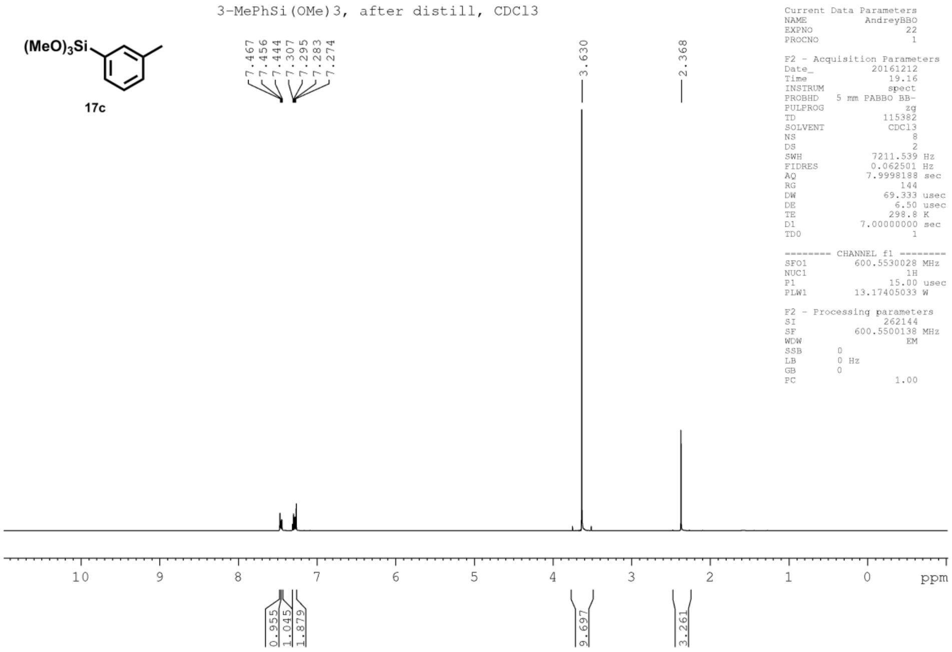

Supplementary figure 189. ^{1}H NMR spectra of **17c** in $CDCl_3$

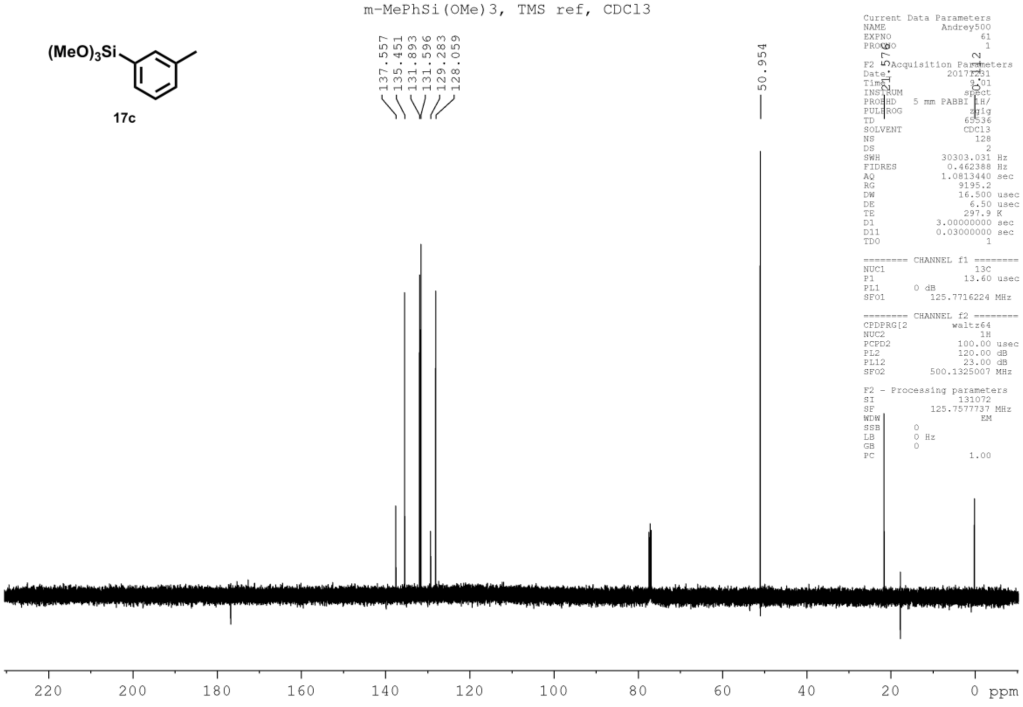

Supplementary figure 190. ^{13}C NMR spectra of **17c** in $CDCl_3$

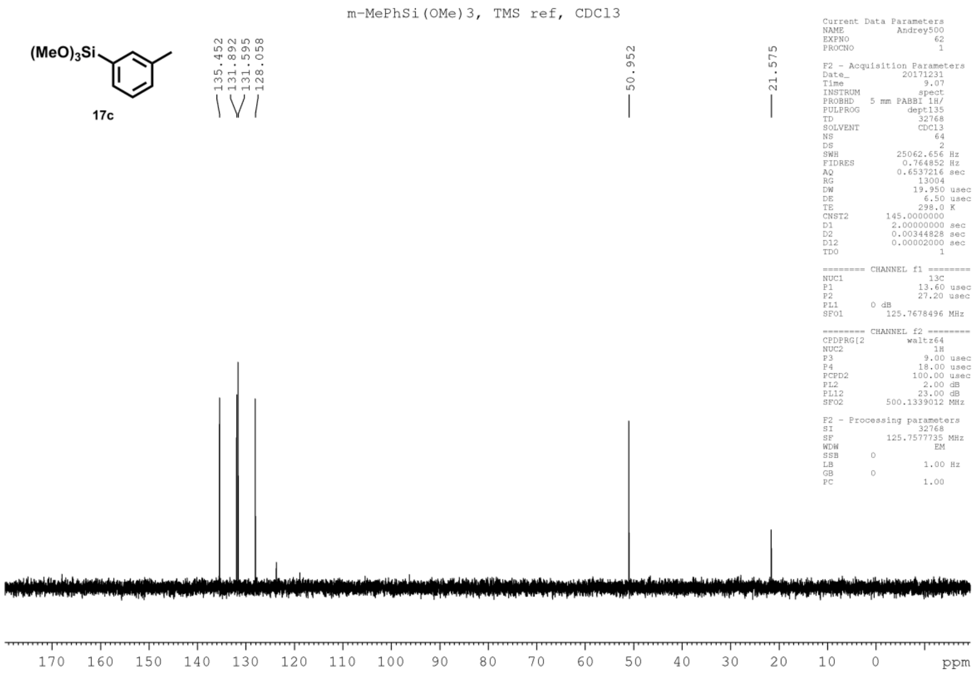

Supplementary figure 191. DEPT-135 NMR spectra of **17c** in $CDCl_3$

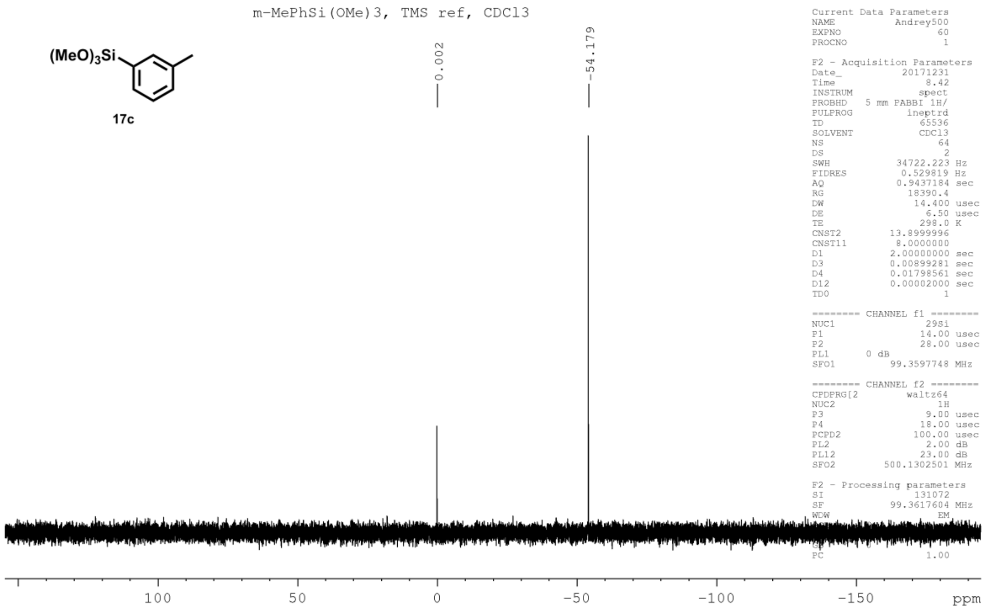

Supplementary figure 192. ^{29}Si NMR spectra of **17c** in $CDCl_3$

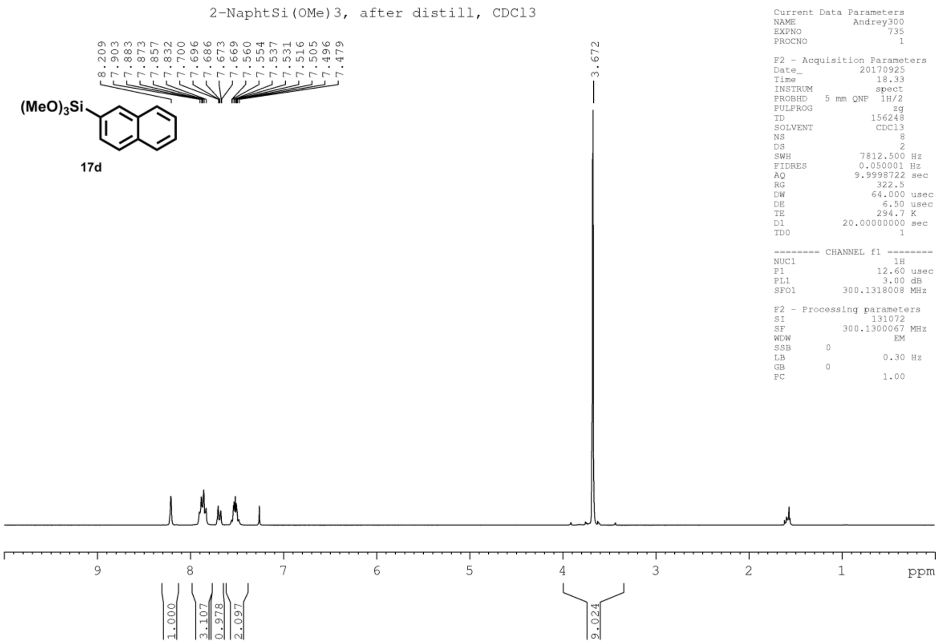

Supplementary figure 193. ^{1}H NMR spectra of **17d** in $CDCl_3$

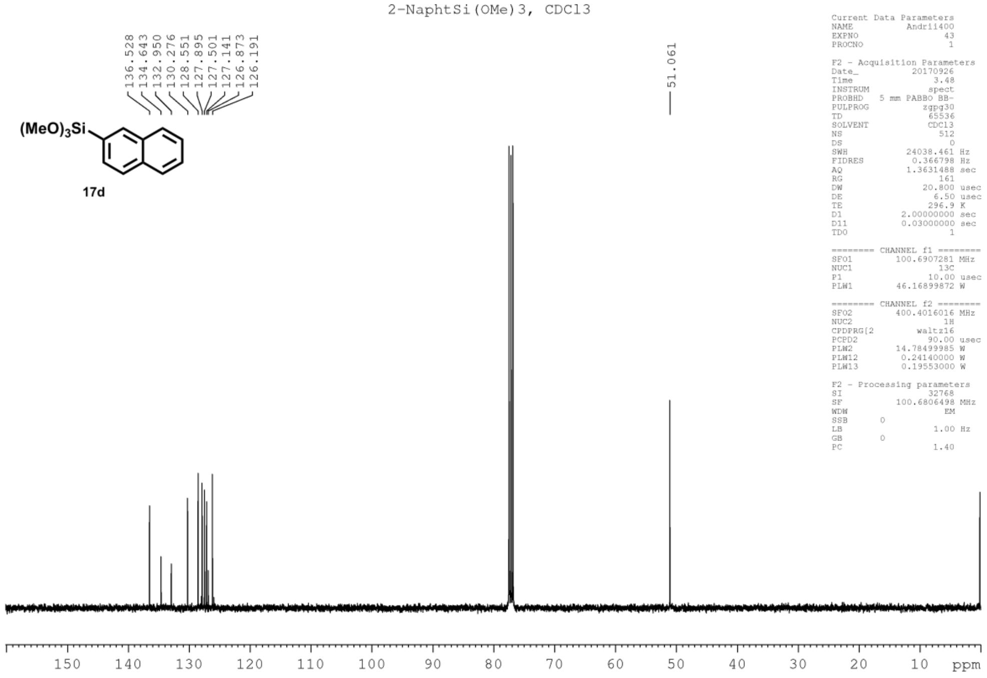

Supplementary figure 194. ^{13}C NMR spectra of **17d** in $CDCl_3$

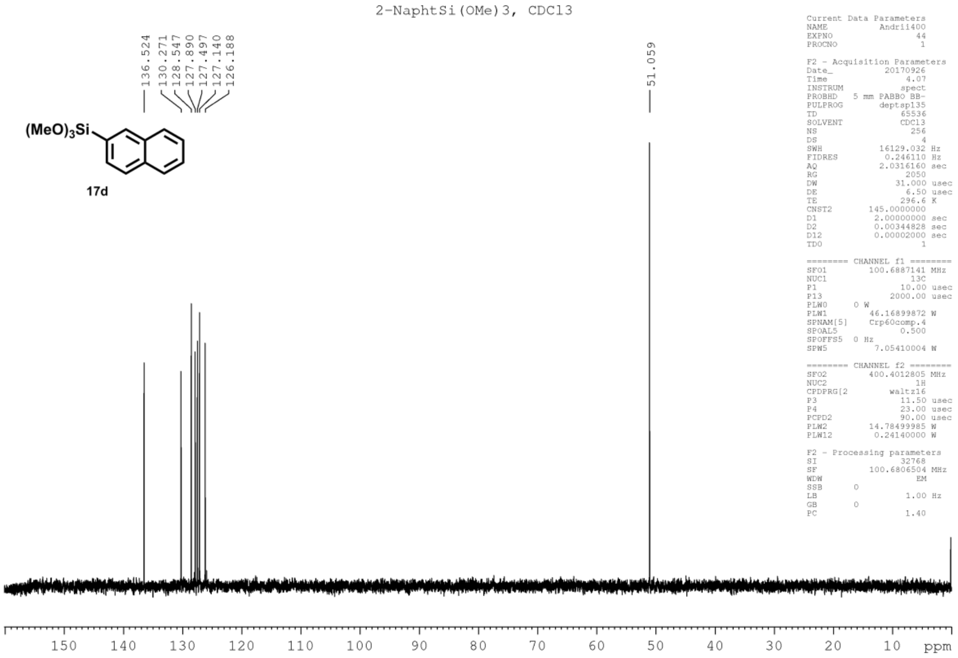

Supplementary figure 195. DEPT-135 NMR spectra of **17d** in $CDCl_3$

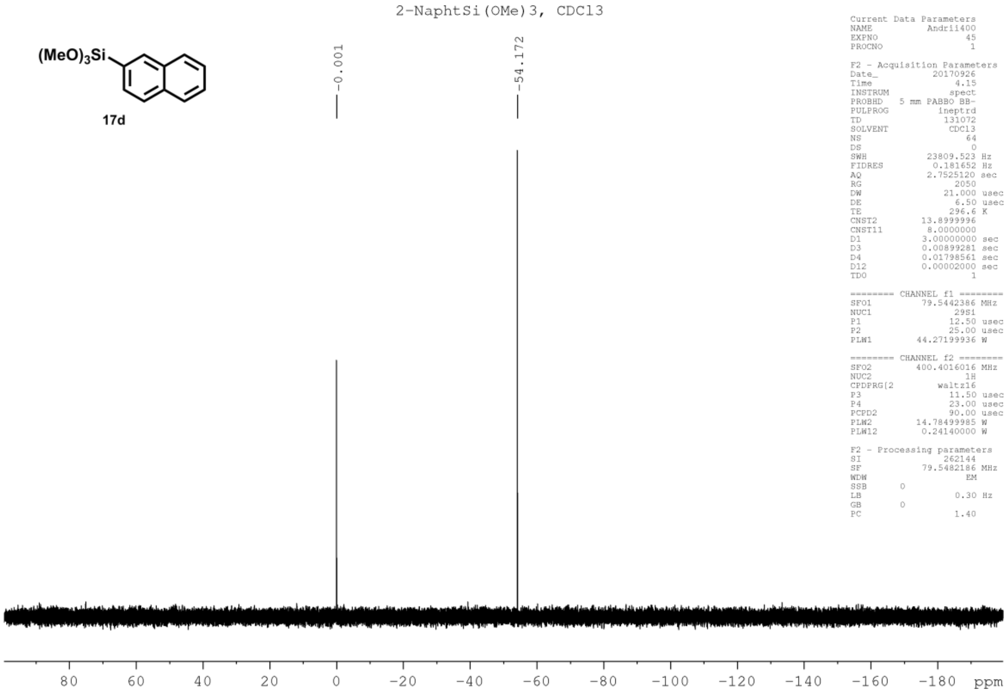

Supplementary figure 196. ^{29}Si NMR spectra of **17d** in $CDCl_3$

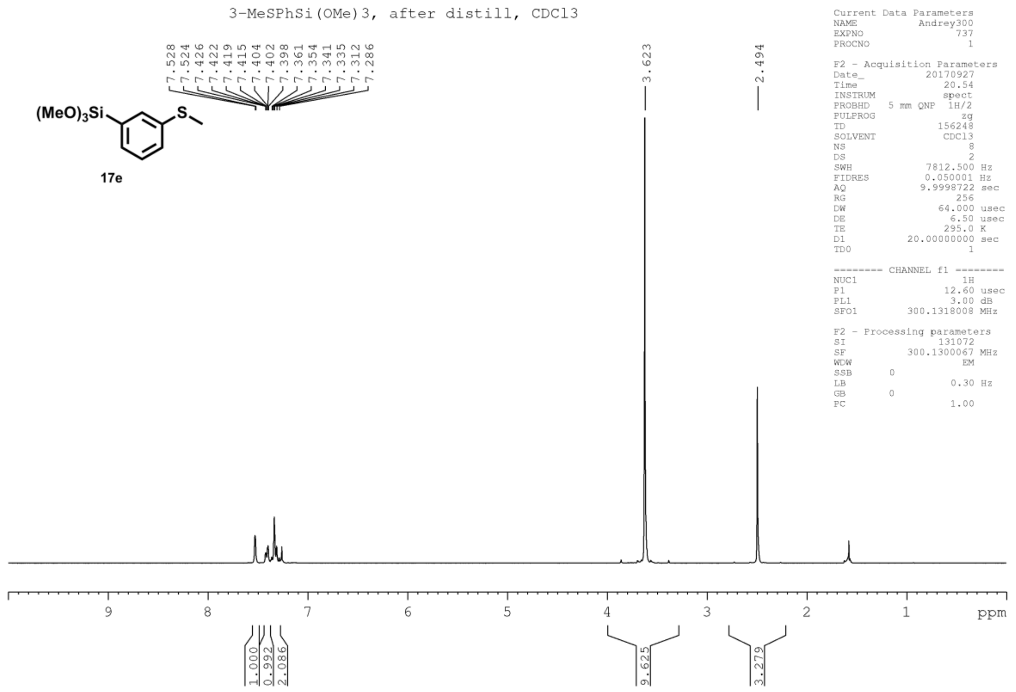

Supplementary figure 197. ^{1}H NMR spectra of **17e** in $CDCl_3$

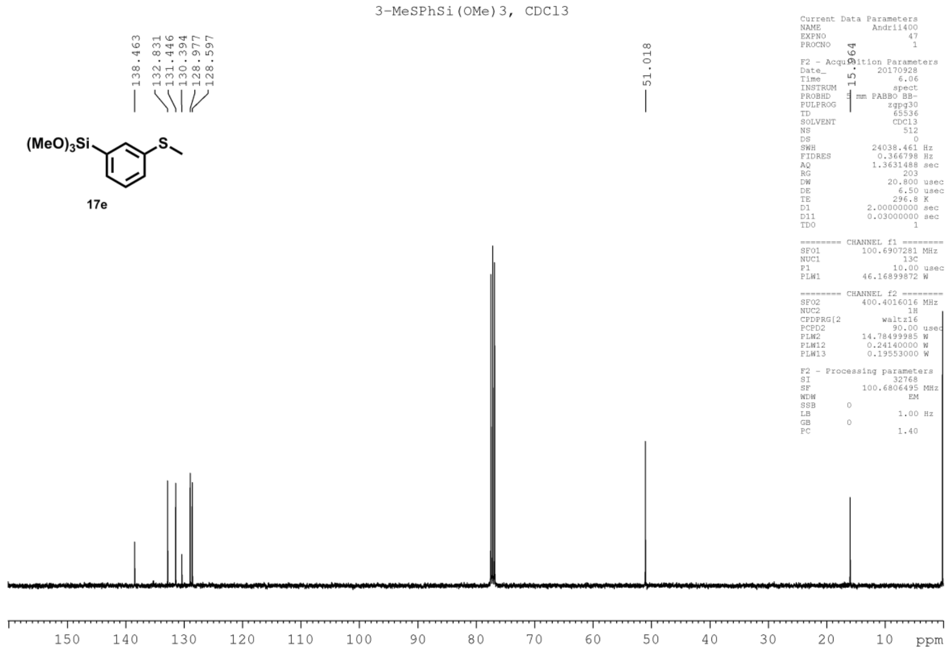

Supplementary figure 198. ^{13}C NMR spectra of **17e** in $CDCl_3$

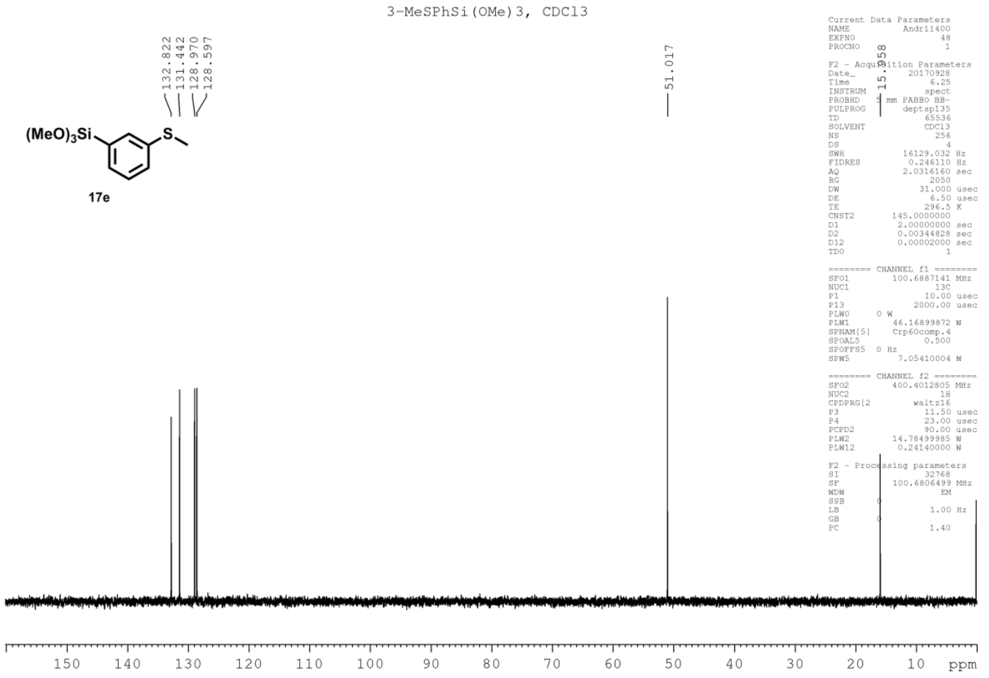

Supplementary figure 199. DEPT-135 NMR spectra of **17e** in $CDCl_3$

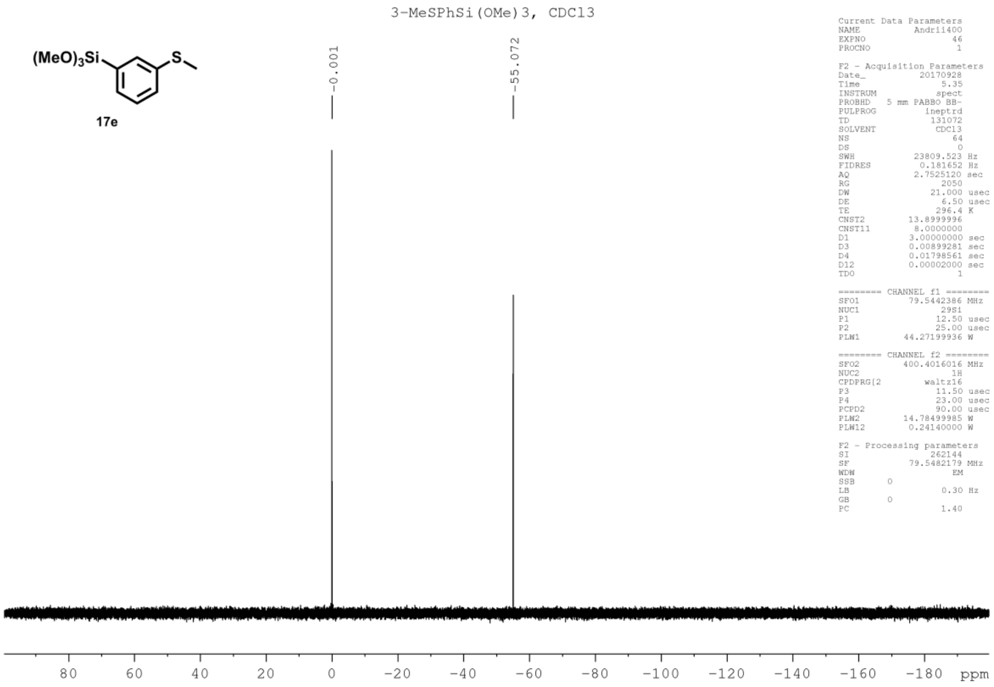

Supplementary figure 200. ^{29}Si NMR spectra of **17e** in $CDCl_3$

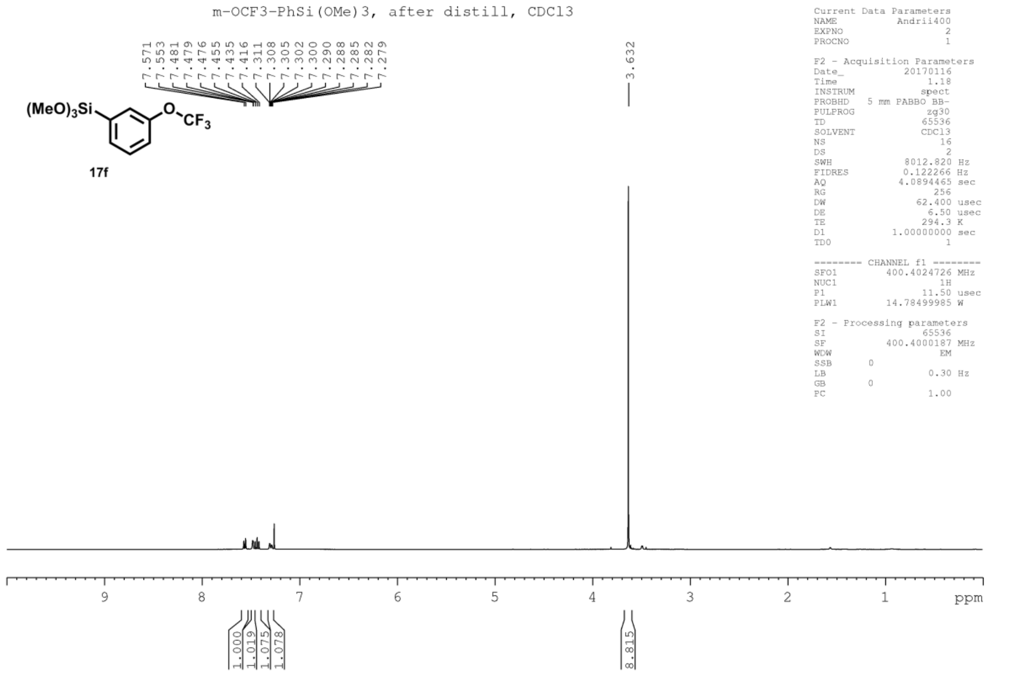

Supplementary figure 201. ^{1}H NMR spectra of **17f** in $CDCl_3$

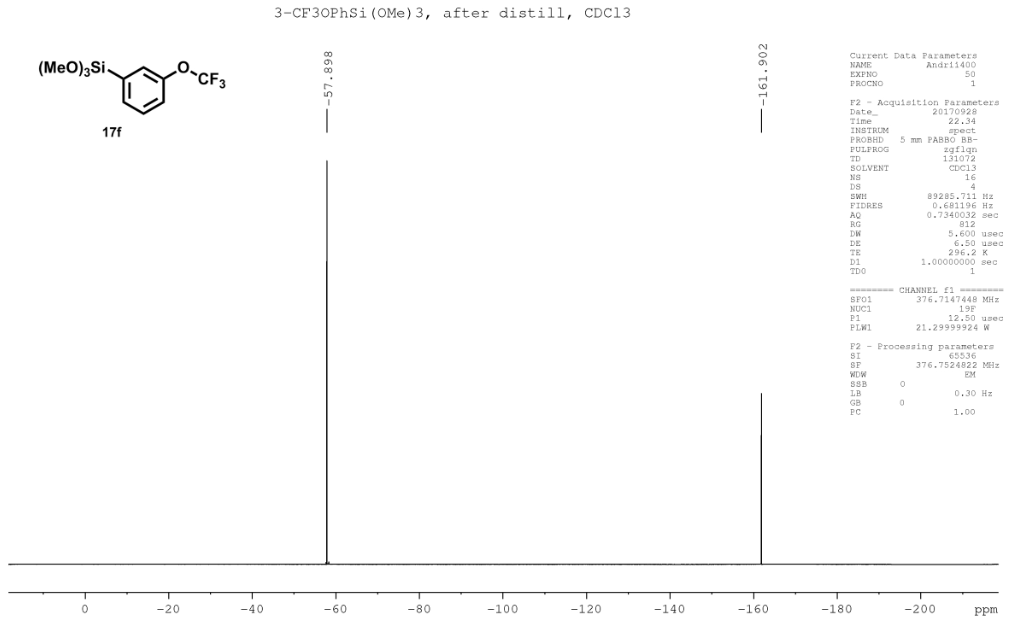

Supplementary figure 202. ^{19}F NMR spectra of **17f** in $CDCl_3$

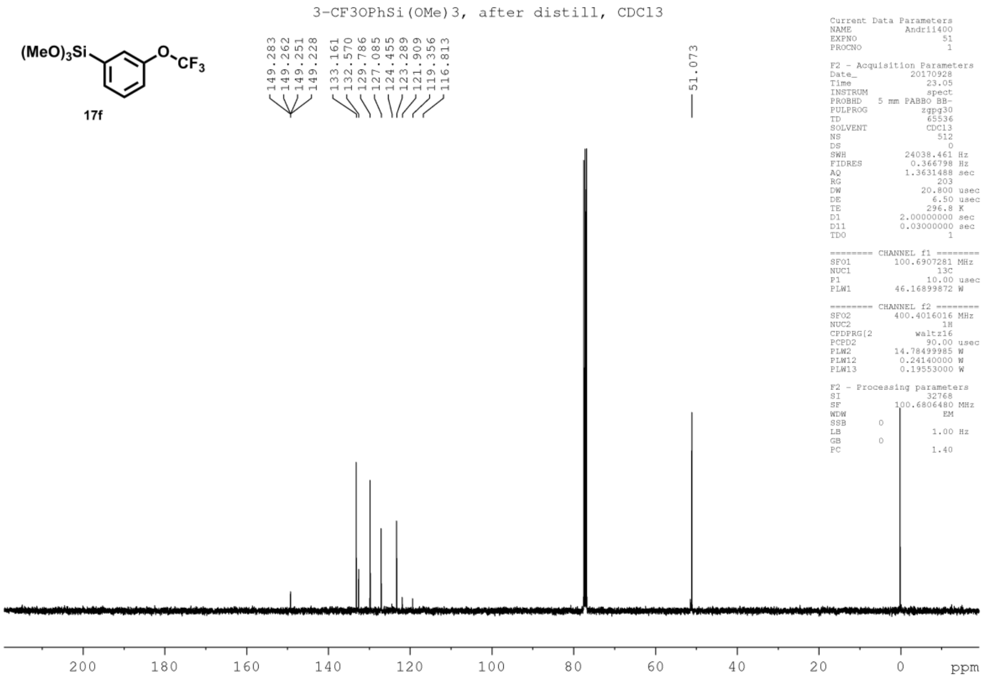

Supplementary figure 203. ^{13}C NMR spectra of **17f** in $CDCl_3$

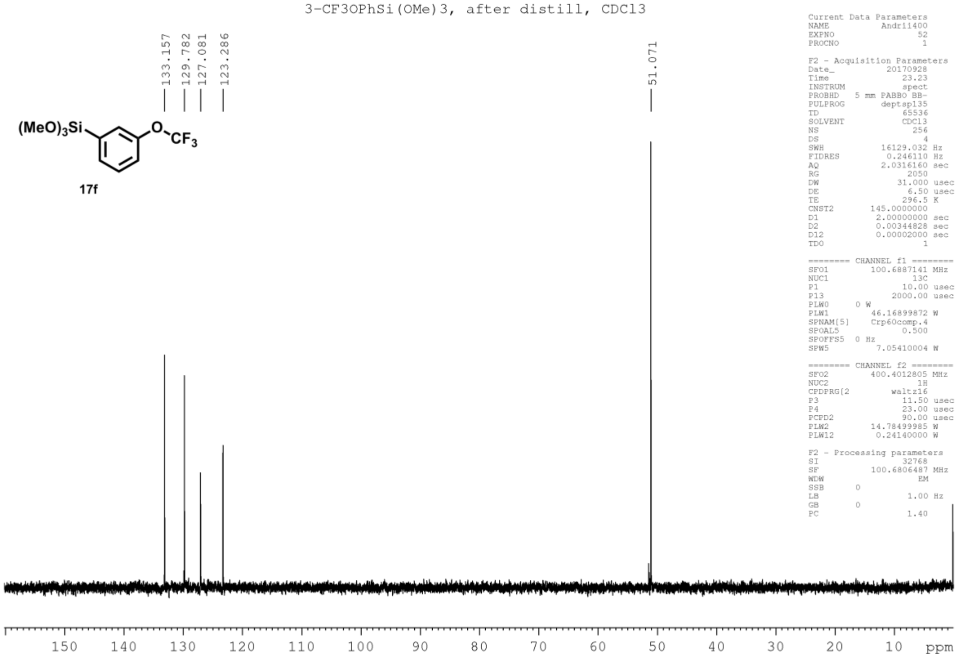

Supplementary figure 204. DEPT-135 NMR spectra of **17f** in $CDCl_3$

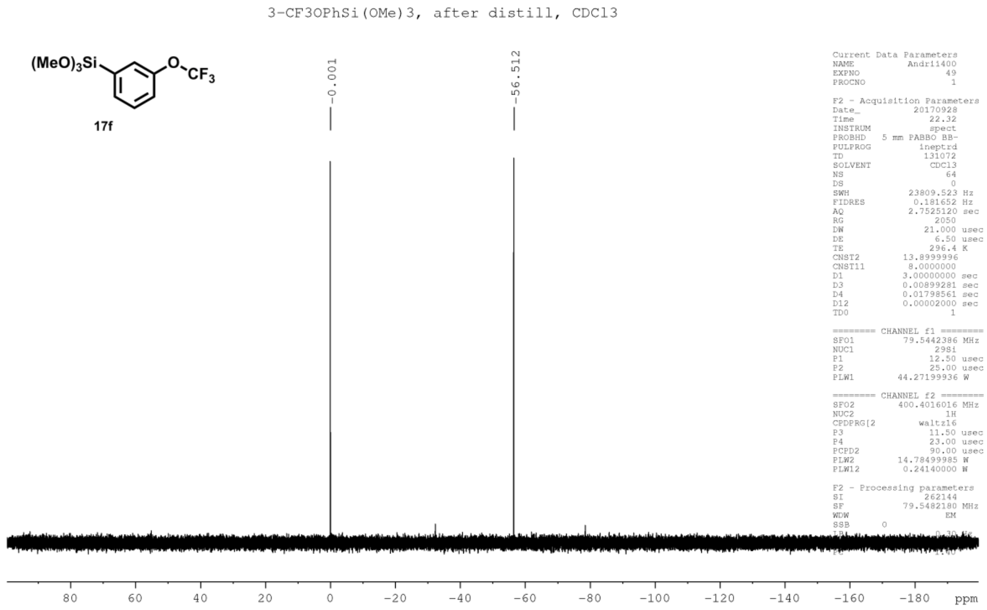

Supplementary figure 205. ^{29}Si NMR spectra of **17f** in $CDCl_3$

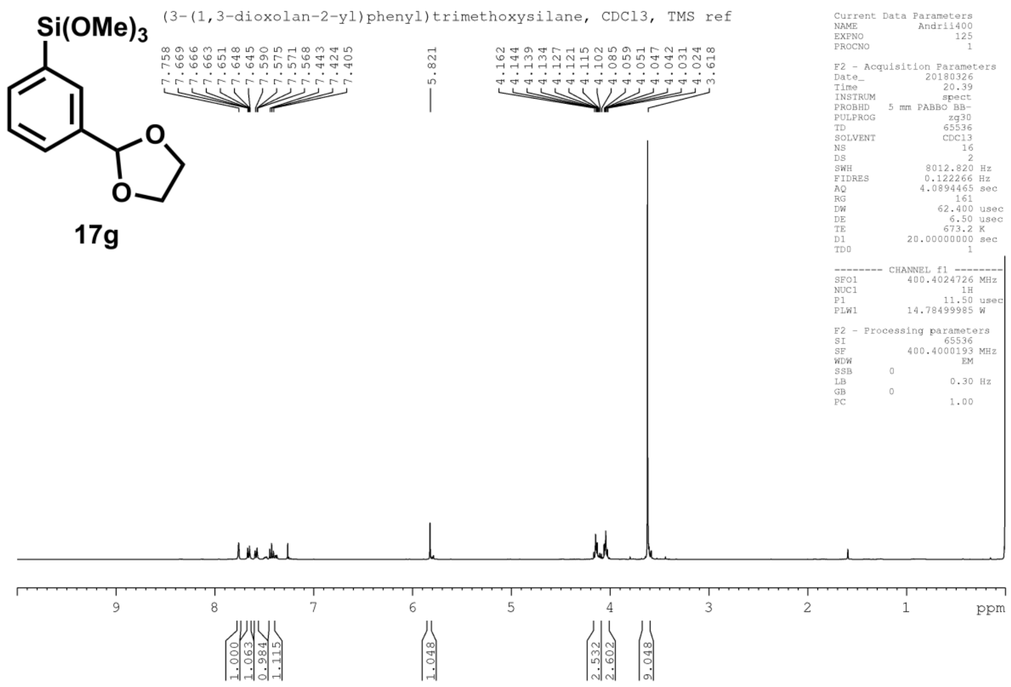

Supplementary figure 206. ^{1}H NMR spectra of **17g** in $CDCl_3$

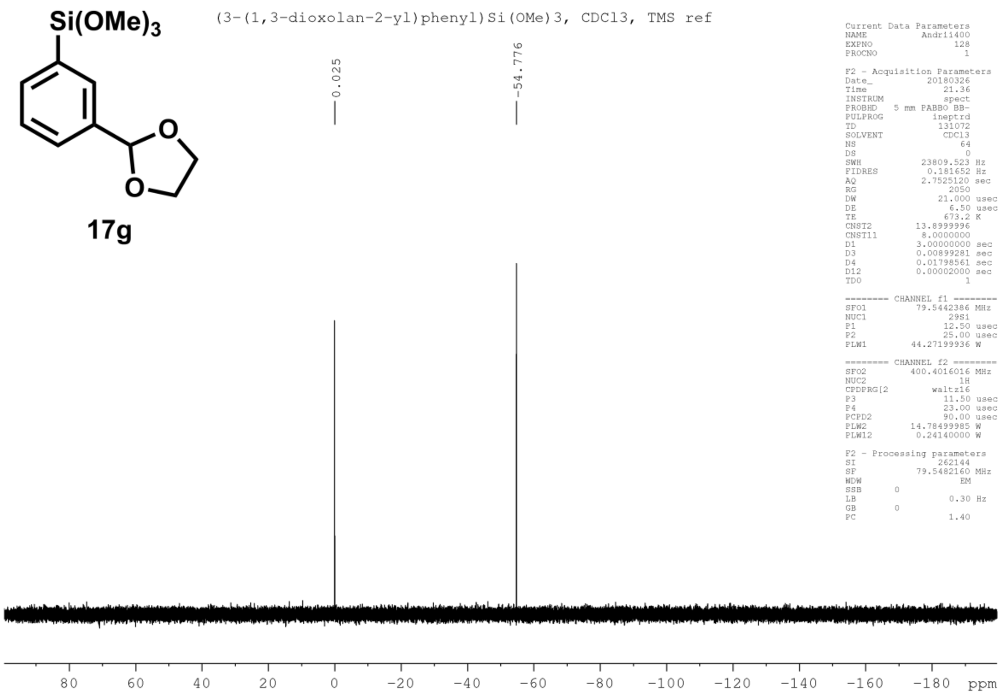

Supplementary figure 207. ^{29}Si NMR spectra of **17g** in $CDCl_3$

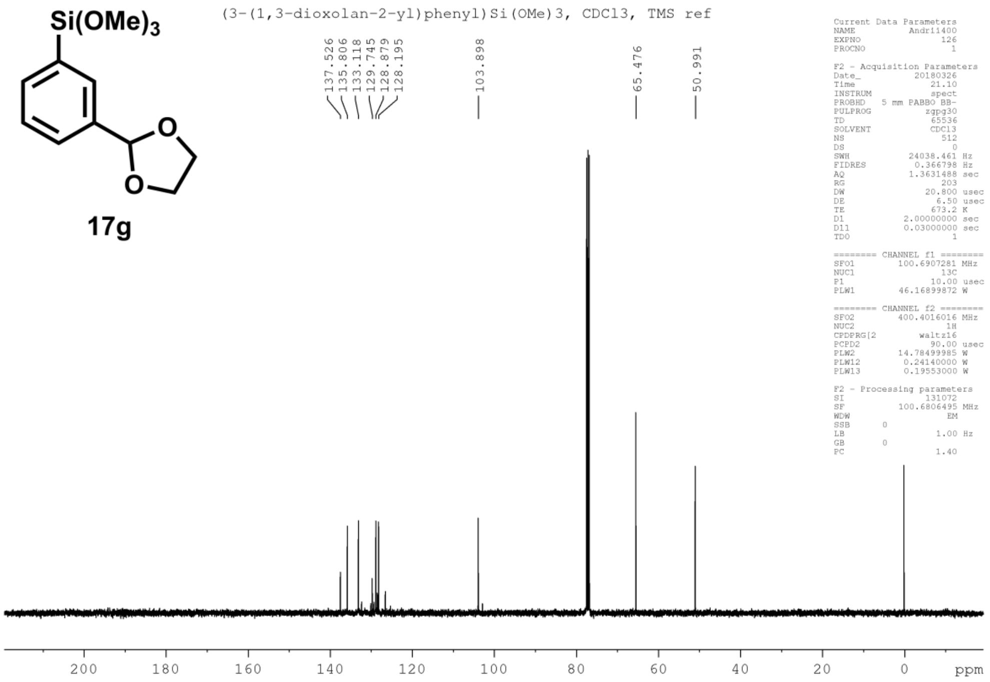

Supplementary figure 208. ^{13}C NMR spectra of **17g** in $CDCl_3$

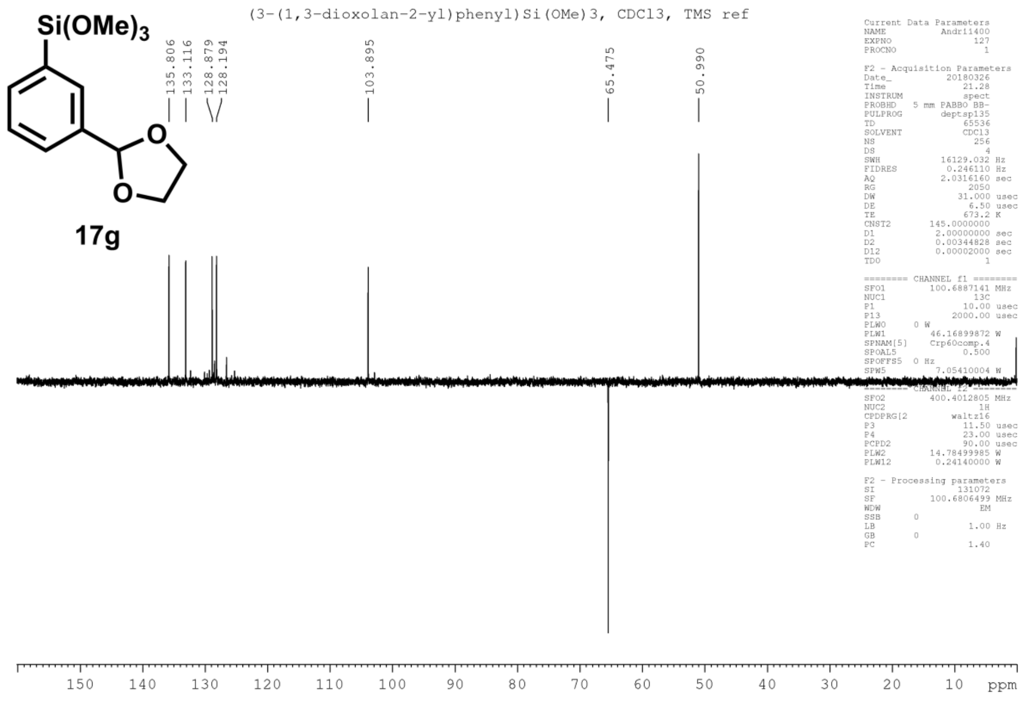

Supplementary figure 209. SEPT-135 NMR spectra of **17g** in $CDCl_3$

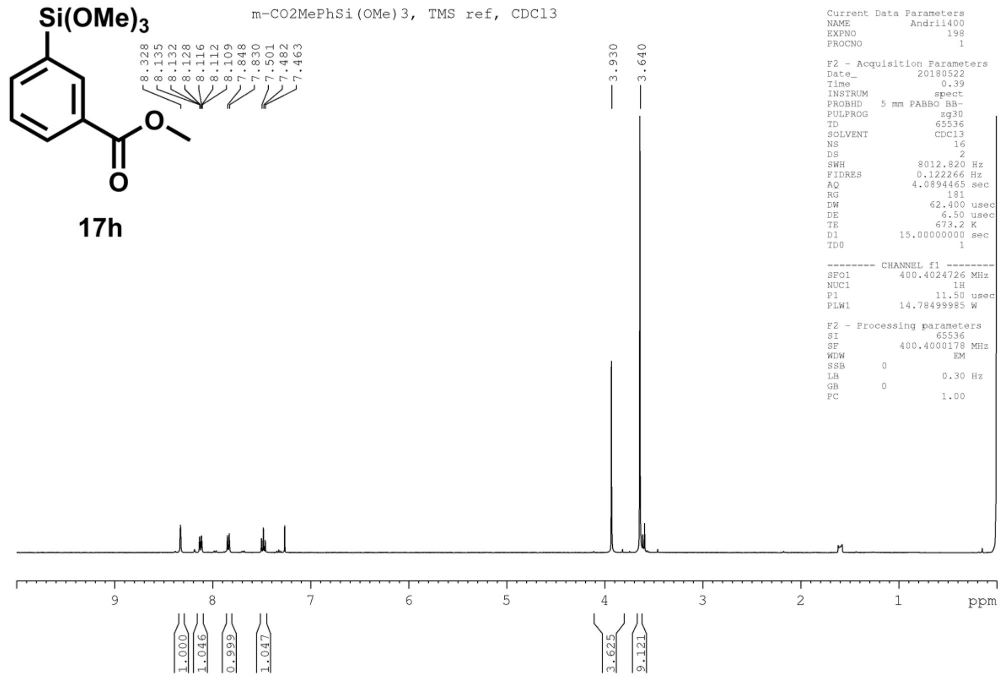

Supplementary figure210. ^{1}H NMR spectra of **17h** in $CDCl_3$

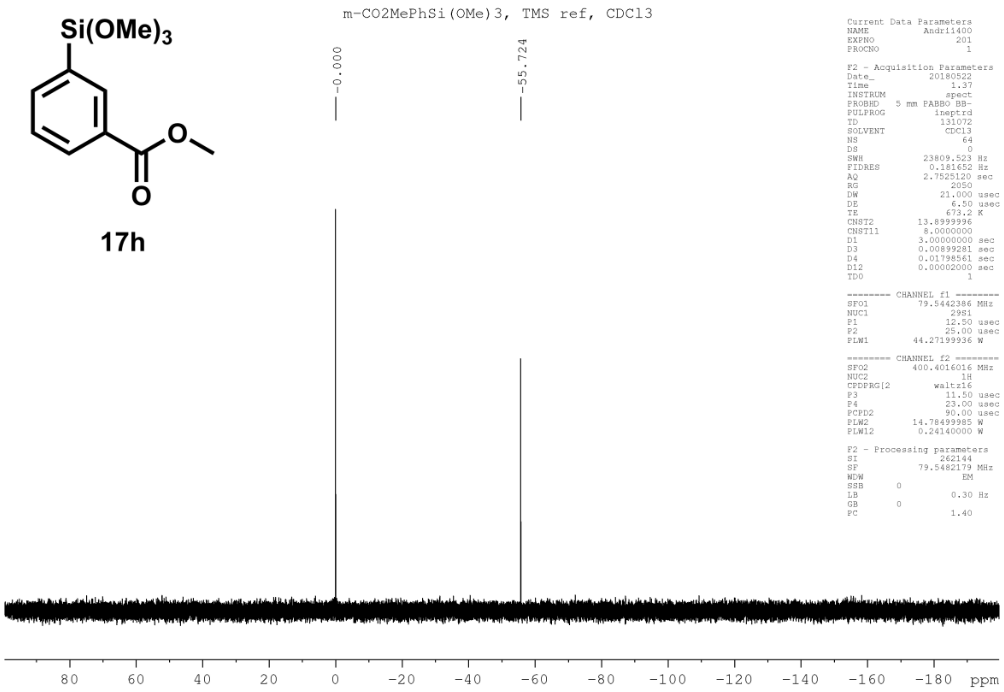

Supplementary figure 211. ^{29}Si NMR spectra of **17h** in $CDCl_3$

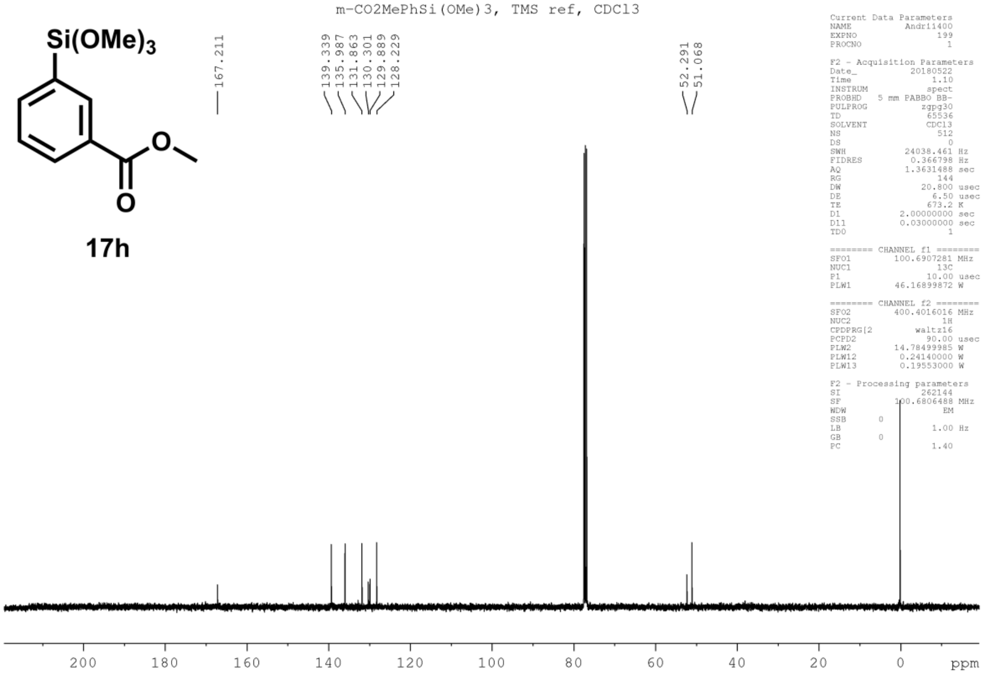

Supplementary figure 212. ^{13}C NMR spectra of **17h** in $CDCl_3$

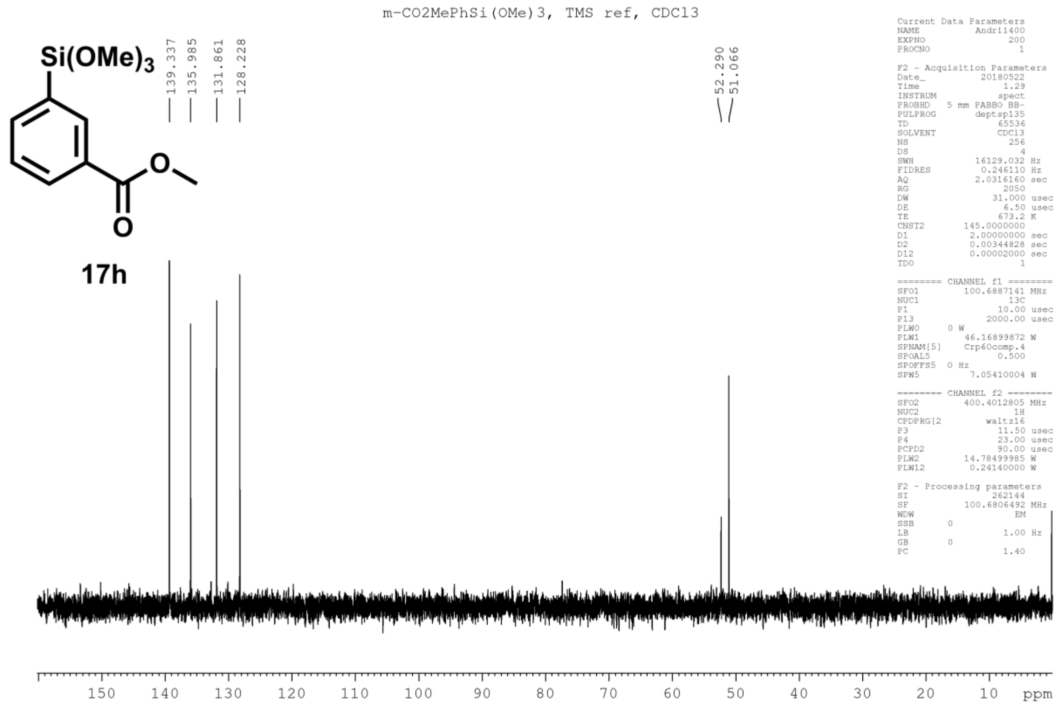

Supplementary figure 213. DEPT-135 NMR spectra of **17h** in $CDCl_3$

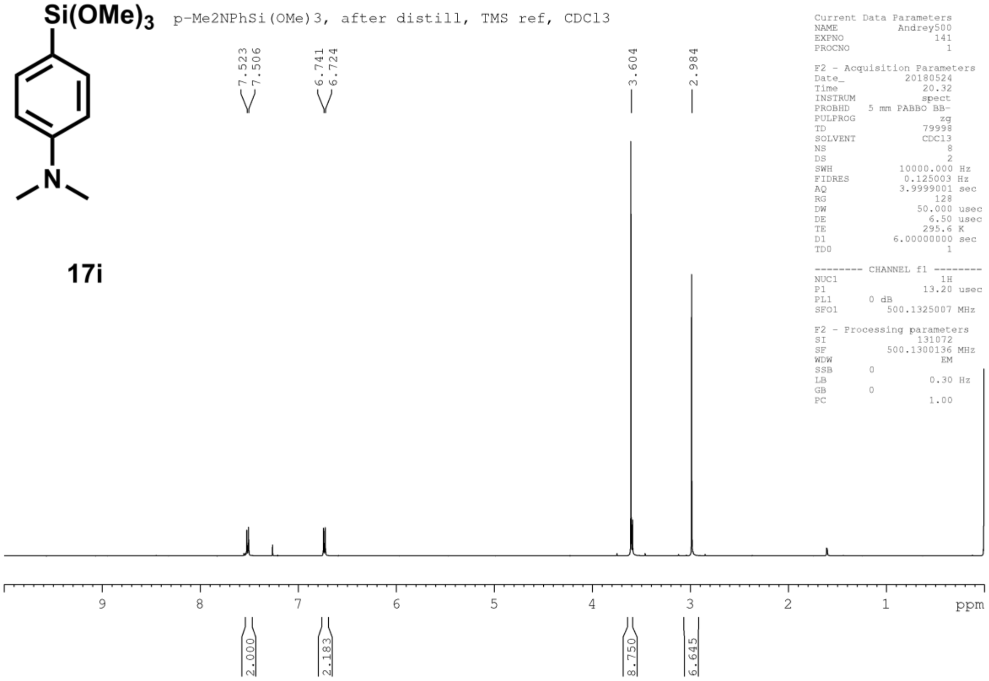

Supplementary figure 214. ^{1}H NMR spectra of **17i** in $CDCl_3$

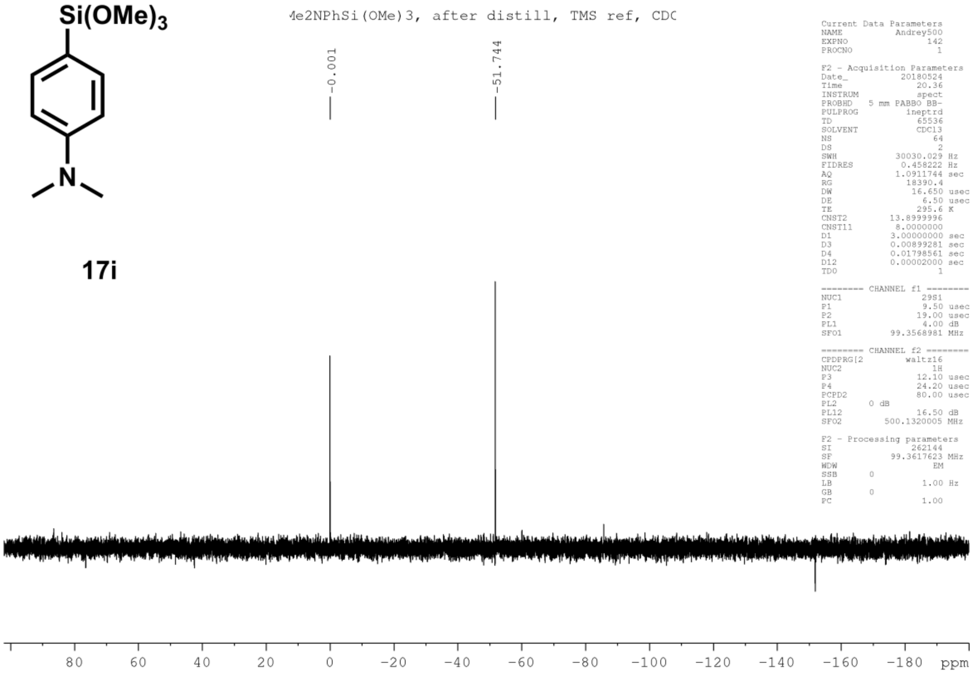

Supplementary figure 215. ^{29}Si NMR spectra of **17i** in $CDCl_3$

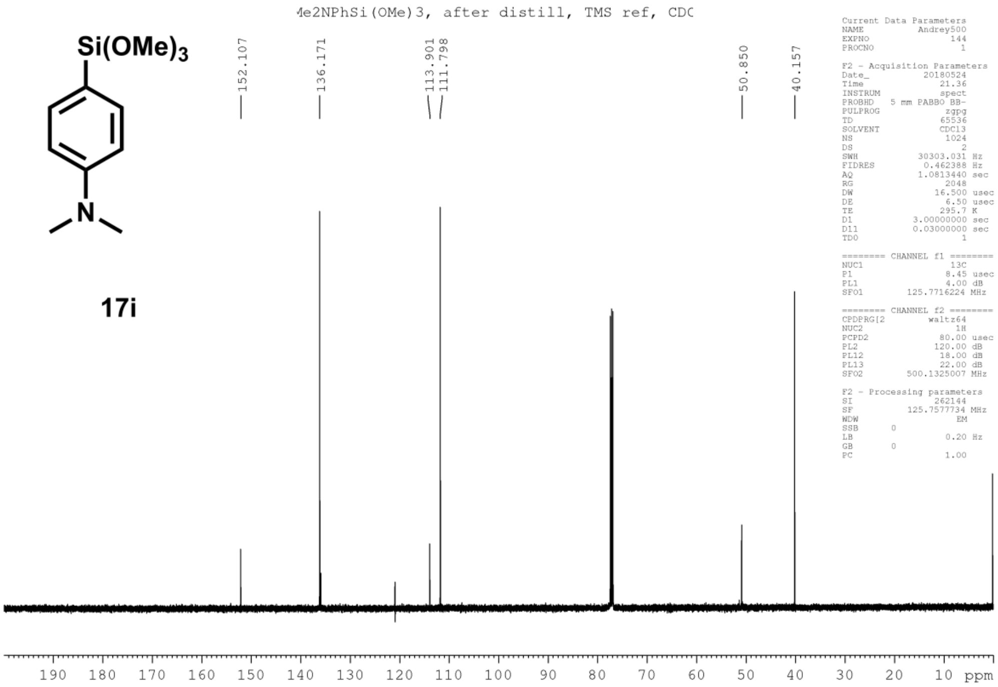

Supplementary figure 216. ^{13}C NMR spectra of **17i** in $CDCl_3$

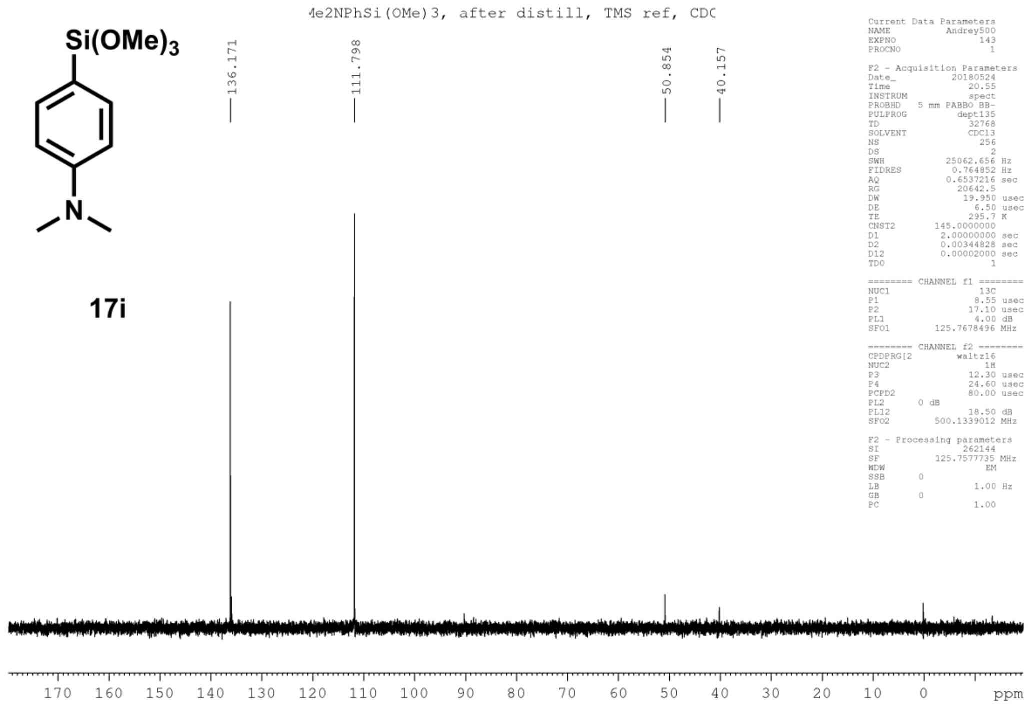

Supplementary figure 217. DEPT-135 NMR spectra of **17i** in $CDCl_3$

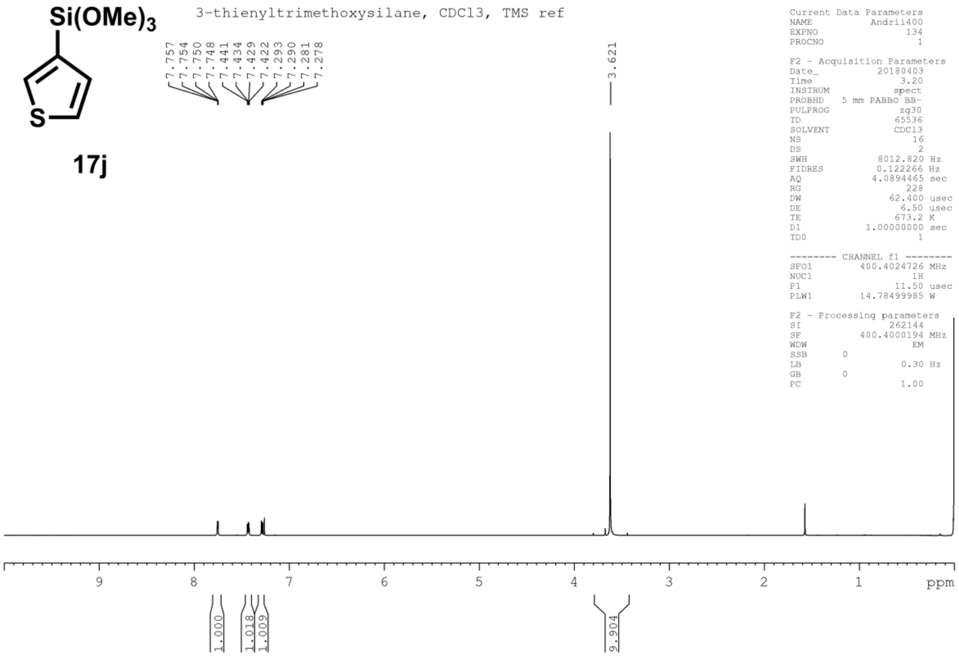

Supplementary figure 218. ^1H NMR spectra of **17j** in $CDCl_3$

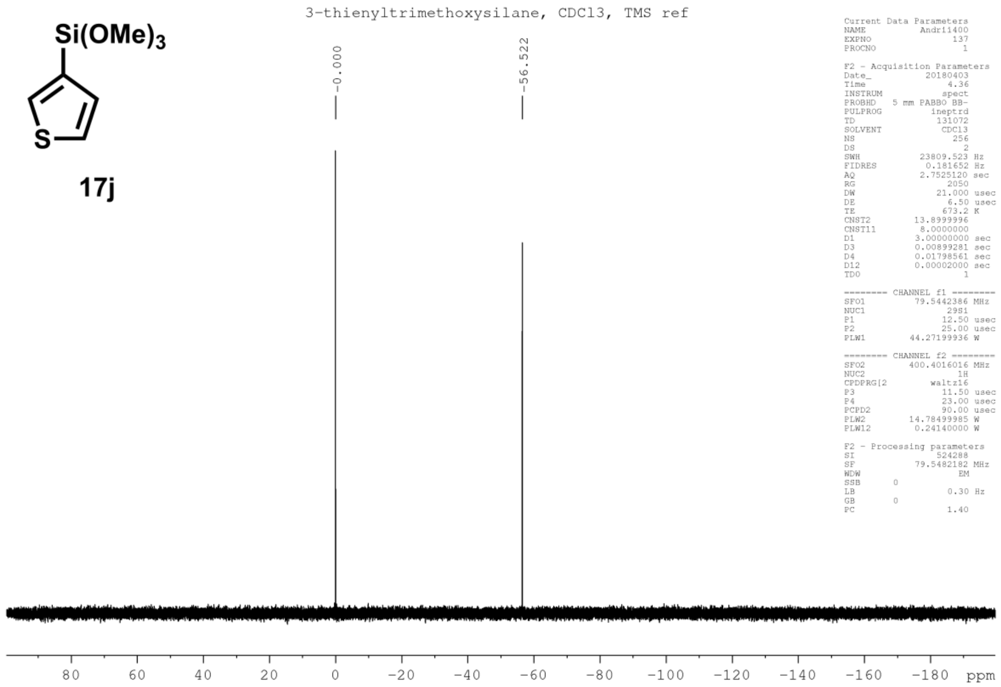

Supplementary figure 219. ^{29}Si NMR spectra of **17j** in $CDCl_3$

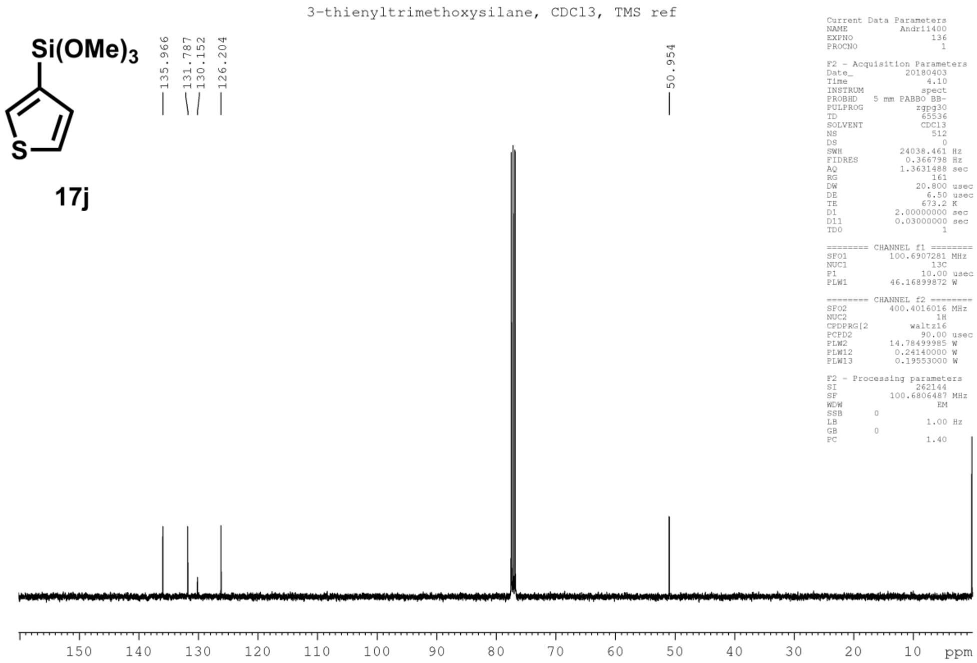

Supplementary figure 220. ^{13}C NMR spectra of **17j** in $CDCl_3$

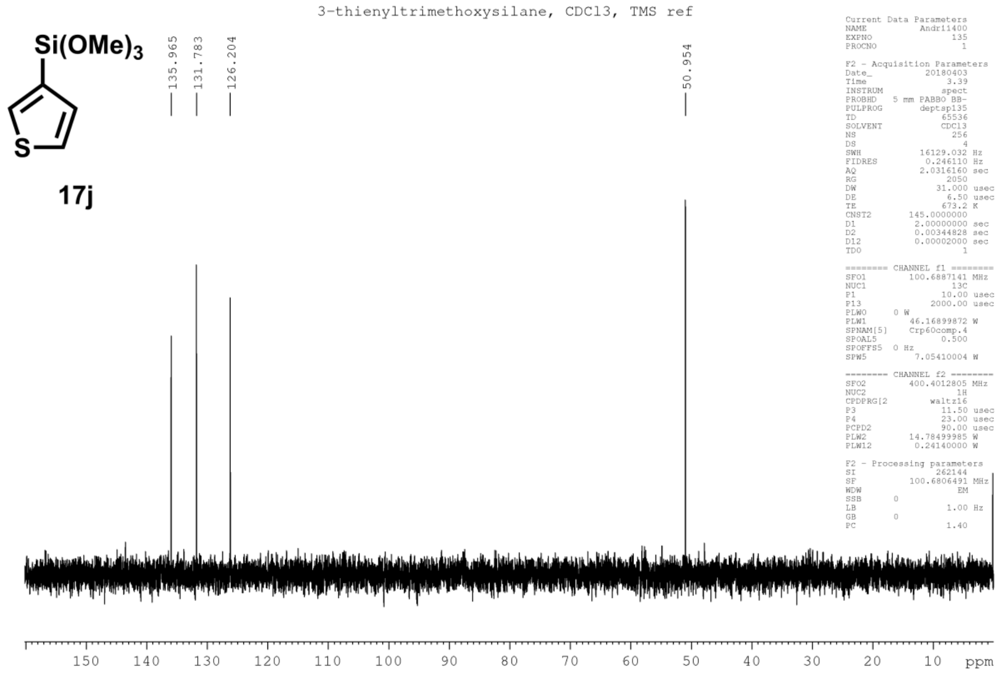

Supplementary figure 221. DEPT-135 NMR spectra of **17j** in $CDCl_3$

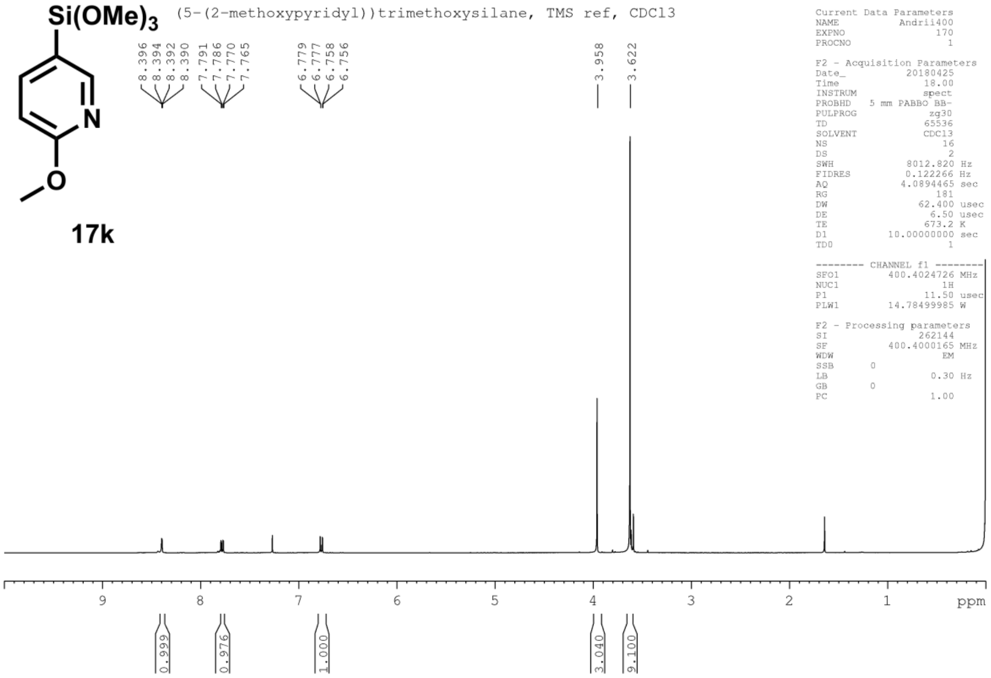

Supplementary figure 222. ^{1}H NMR spectra of **17k** in $CDCl_3$

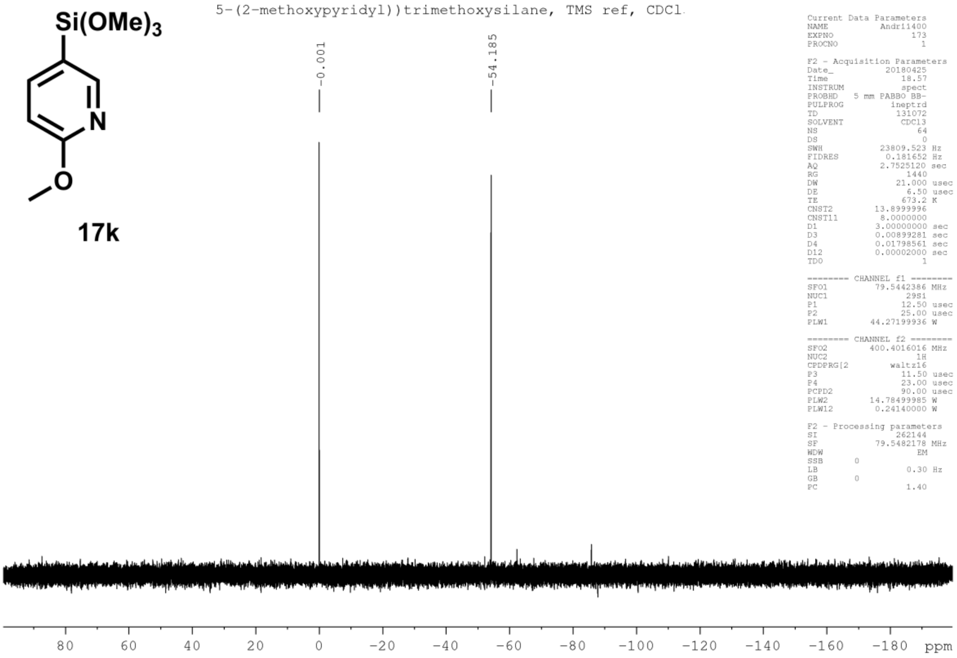

Supplementary figure 223. ^{29}Si NMR spectra of **17k** in $CDCl_3$

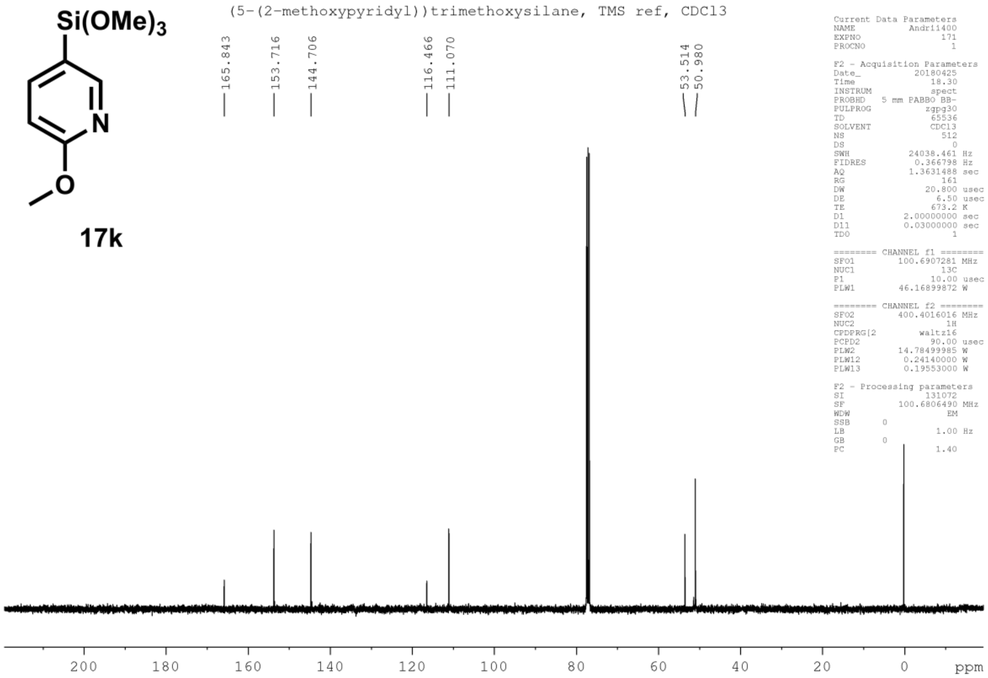

Supplementary figure 224. ^{13}C NMR spectra of **17k** in $CDCl_3$

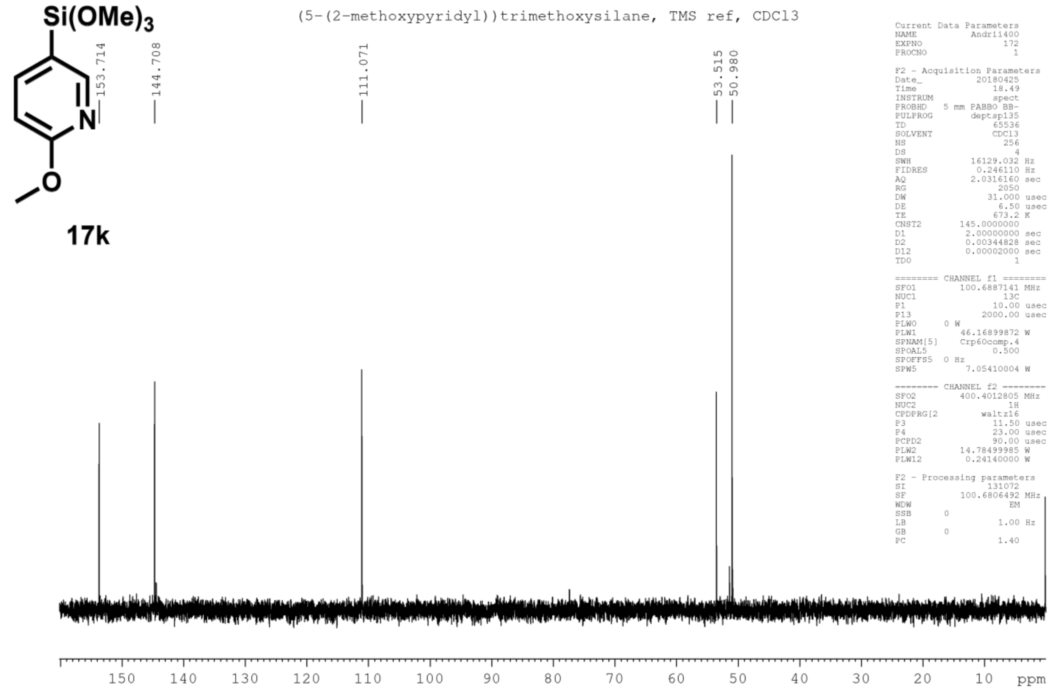

Supplementary figure 225. DEPT-135 NMR spectra of **17k** in $CDCl_3$

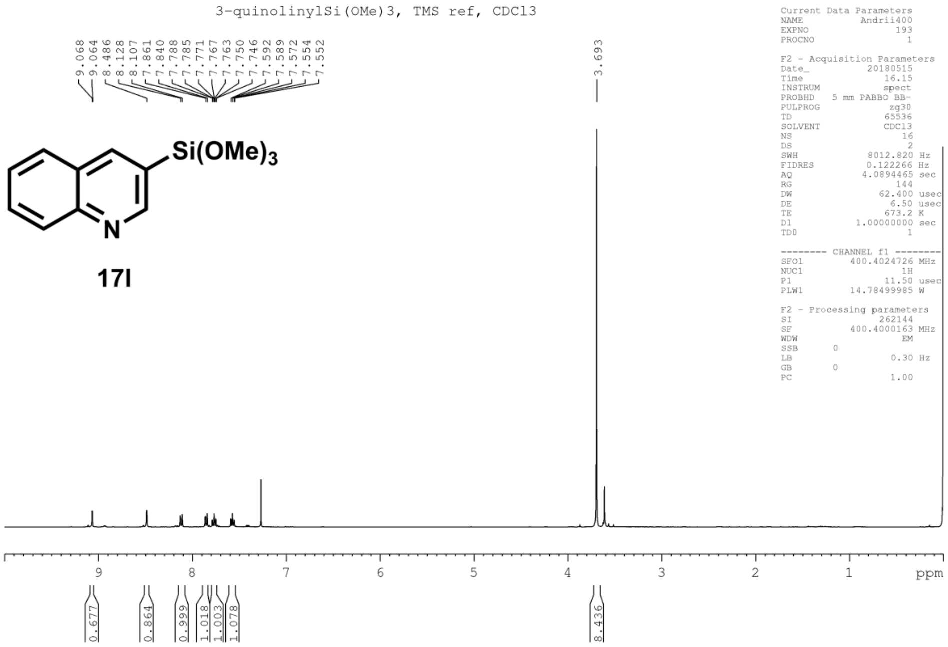

Supplementary figure 226. ^{1}H NMR spectra of **17l** in $CDCl_3$

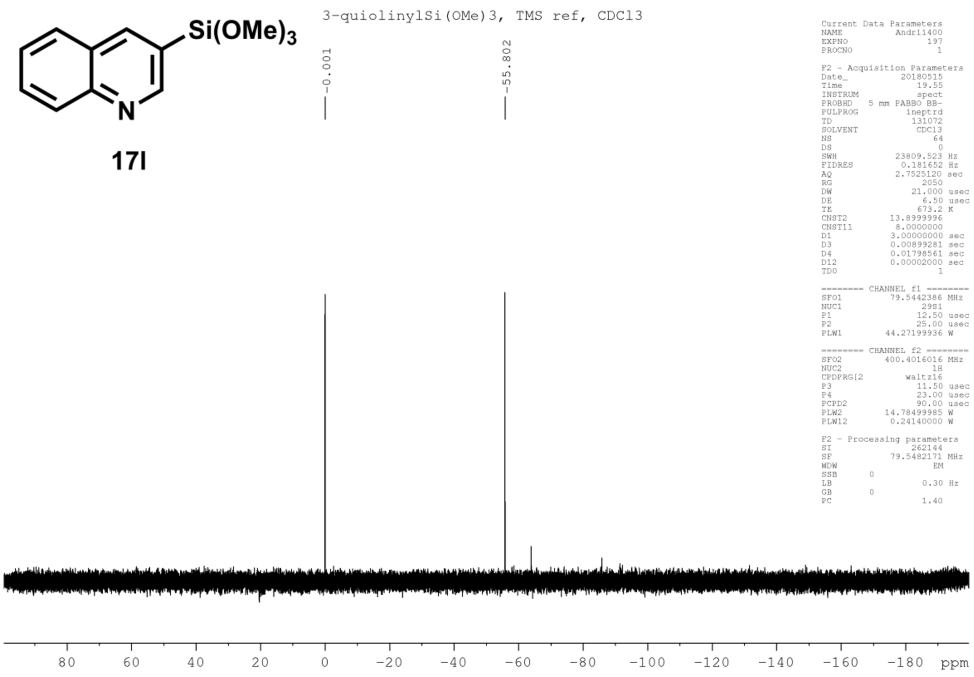

Supplementary figure 227. ^{29}Si NMR spectra of **17l** in $CDCl_3$

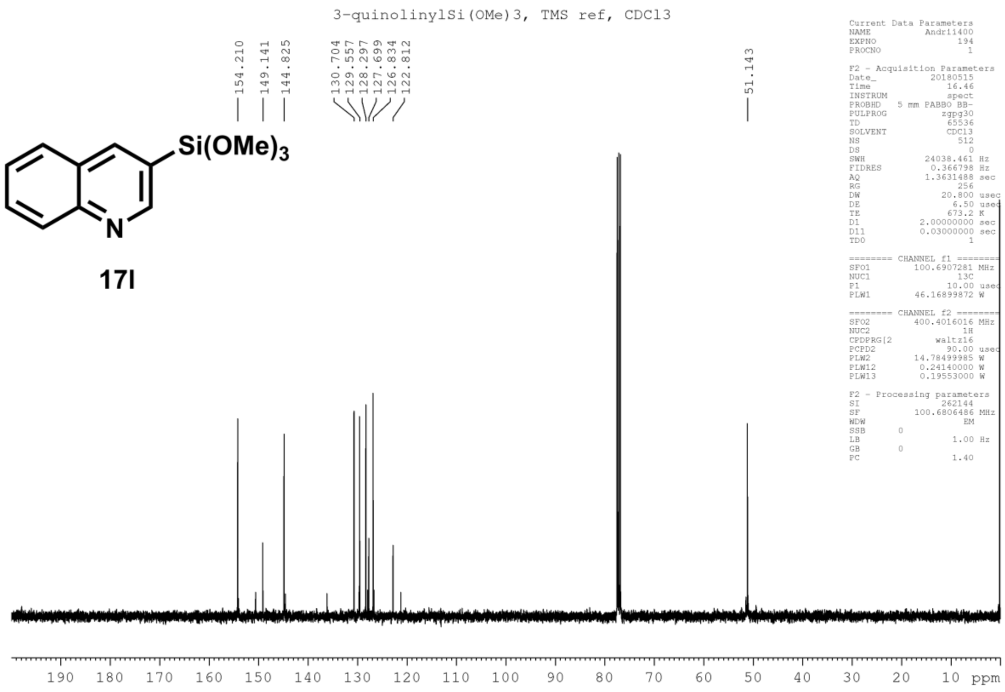

Supplementary figure 228. ^{13}C NMR spectra of **17l** in $CDCl_3$

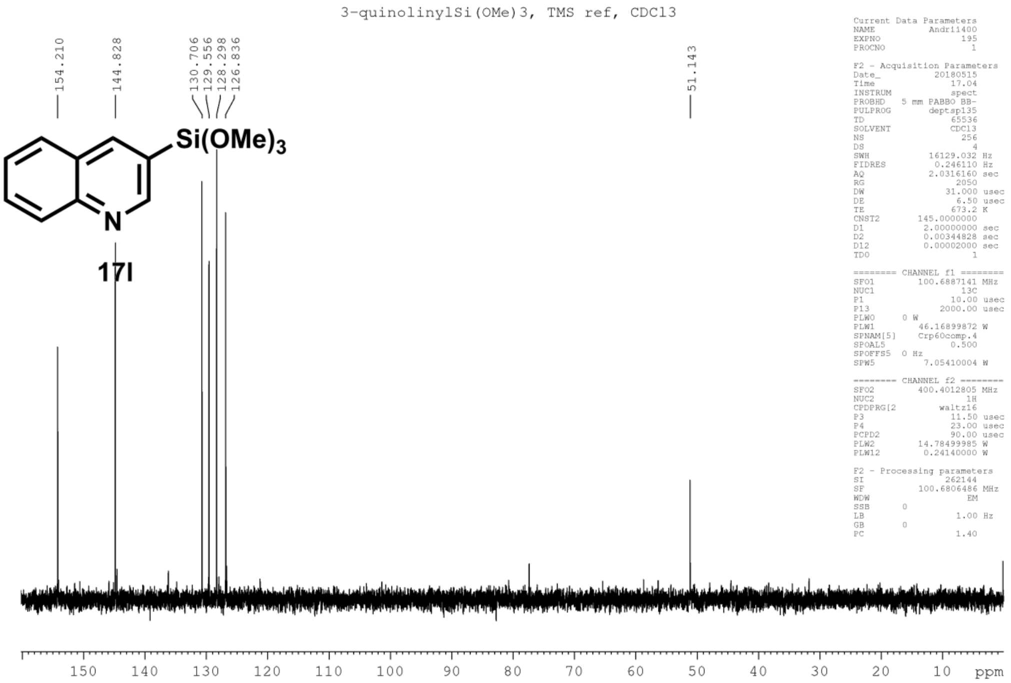

Supplementary figure 229. DEPT NMR spectra of **17l** in $CDCl_3$

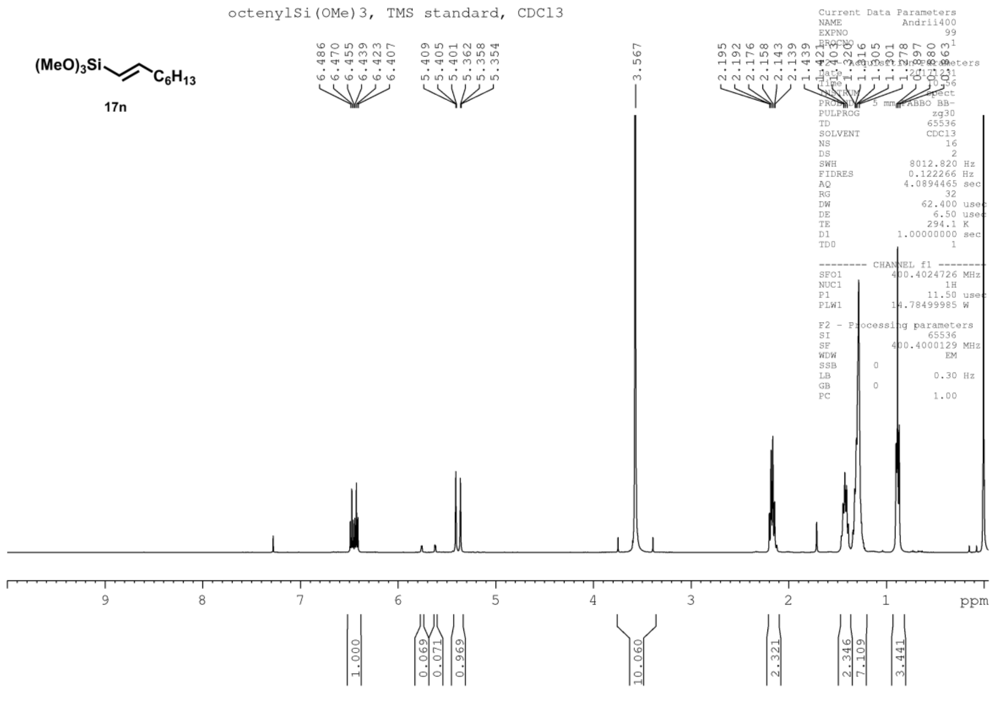

Supplementary figure 230. ^{1}H NMR spectra of **17n** in $CDCl_3$

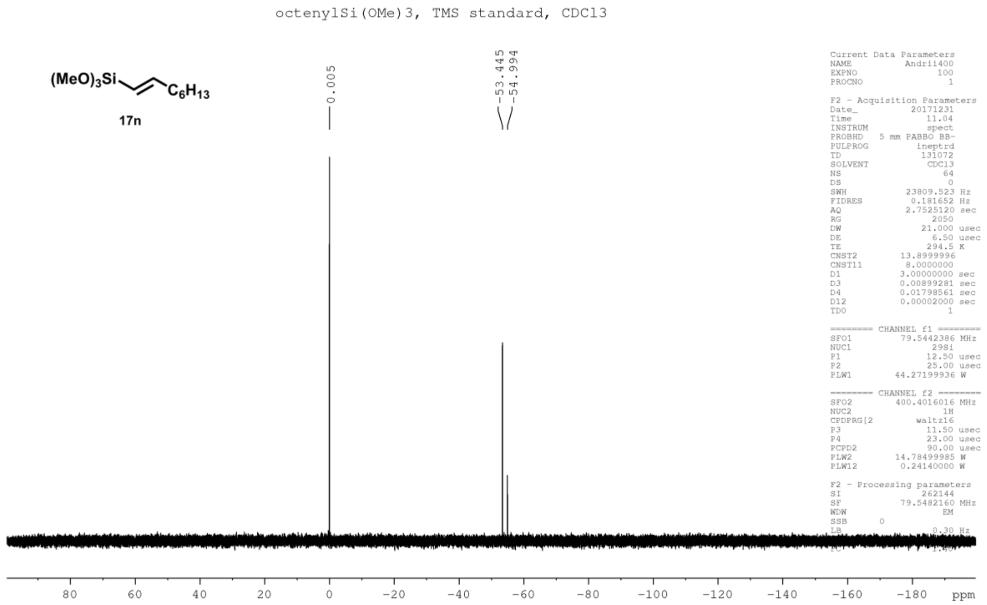

Supplementary figure 231. ^{29}Si NMR spectra of **17n** in $CDCl_3$

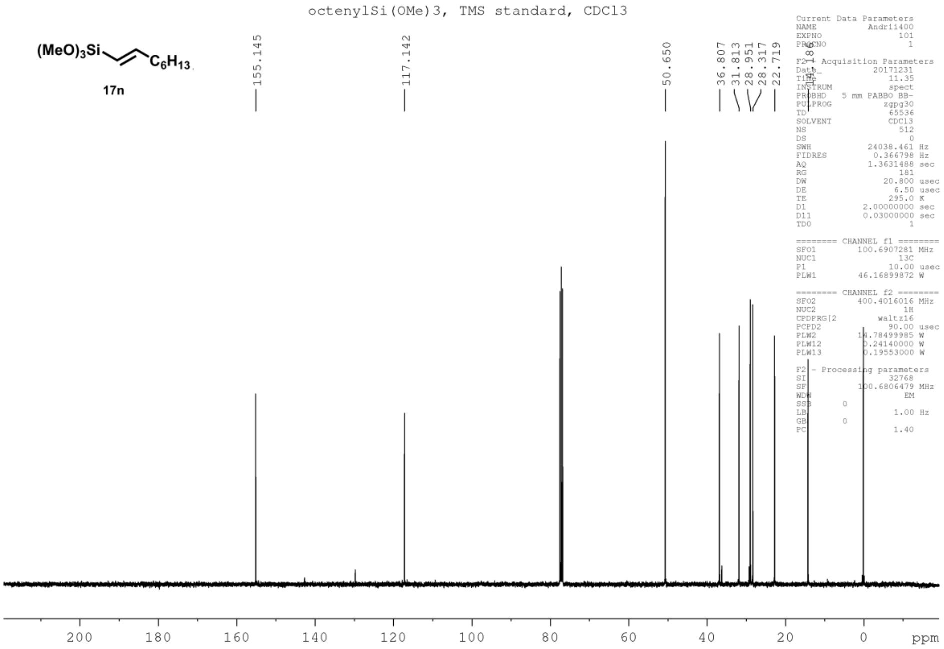

Supplementary figure 232. ^{13}C NMR spectra of **17n** in $CDCl_3$

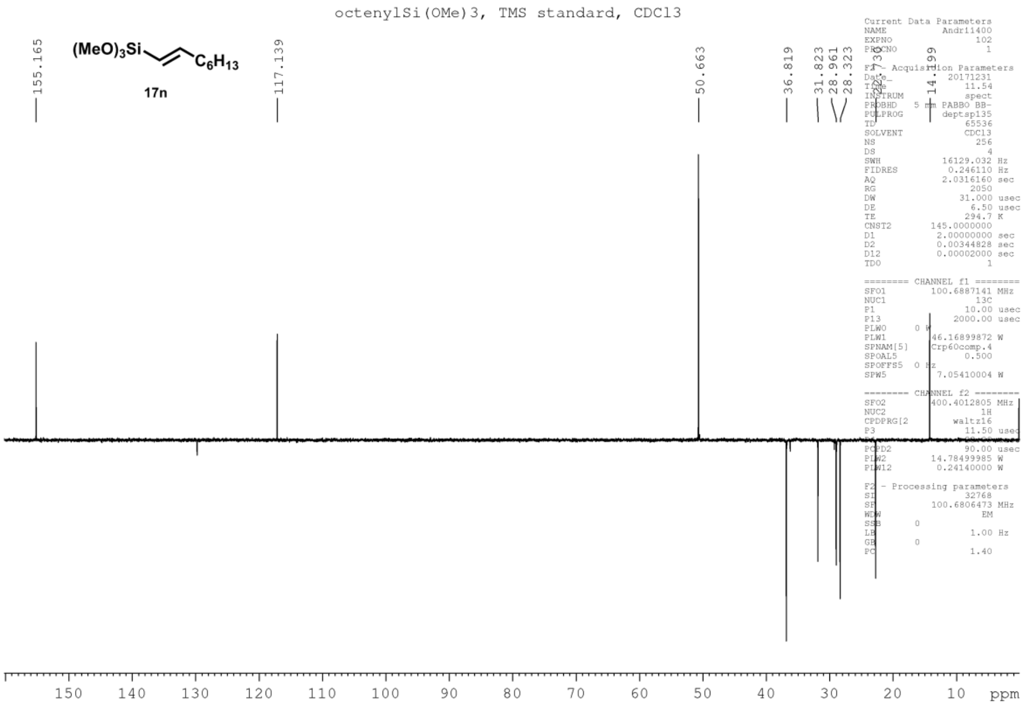

Supplementary figure 233. DEPT-135 NMR spectra of **17n** in $CDCl_3$

SHIMADZU LabSolutions **Analysis Report**

<Sample Information>

Sample Name	: CF3-(Ph)OBnCF3, old Me(4,5diPh)BOX		
Sample ID	: CF3-(Ph)OBnCF3, oldMe(4,5diPh)B		
Data Filename	: CF3-(Ph)OBnCF3, old Me(4,5diPh)BOX8.lcd		
Method Filename	: new.lcm		
Batch Filename	:		
Vial #	: 1-19	Sample Type	: Unknown
Injection Volume	: 4 uL		
Date Acquired	: 10/02/2017 02:19:18	Acquired by	: System Administrator
Date Processed	: 10/02/2017 05:26:51	Processed by	: System Administrator

<Chromatogram>

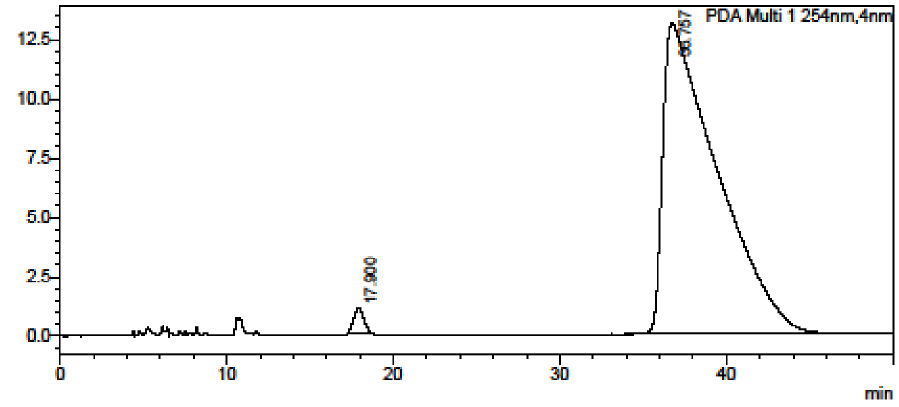

<Peak Table>

PDA Ch1 254nm

Peak#	Ret. Time	Area	Area%	Height
1	17.900	46797	1.548	1074
2	36.757	2976625	98.452	13088
Total		3023422	100.000	14163

C:\Andrey\CF3-(Ph)OBnCF3, old Me(4,5diPh)BOX8.lcd

Supplementary figure 234. HPLC chromatogram for compound **6a,** Ligand:**(4S,5R)-7a**

10/05/2017 16:17:48 Page 1 / 1

SHIMADZU LabSolutions **Analysis Report**

<Sample Information>

Sample Name	: CF3(Ph)-OBnCF3		
Sample ID	: CF3(Ph)-OBnCF3		
Data Filename	: CF3(Ph)-OBnCF3		
Method Filename	: andrey.lcm		
Batch Filename	:		
Vial #	: 1-43	Sample Type	: Unknown
Injection Volume	: 6 uL		
Date Acquired	: 03/05/2017 13:14:41	Acquired by	: System Administrator
Date Processed	: 10/05/2017 16:15:56	Processed by	: System Administrator

<Chromatogram>

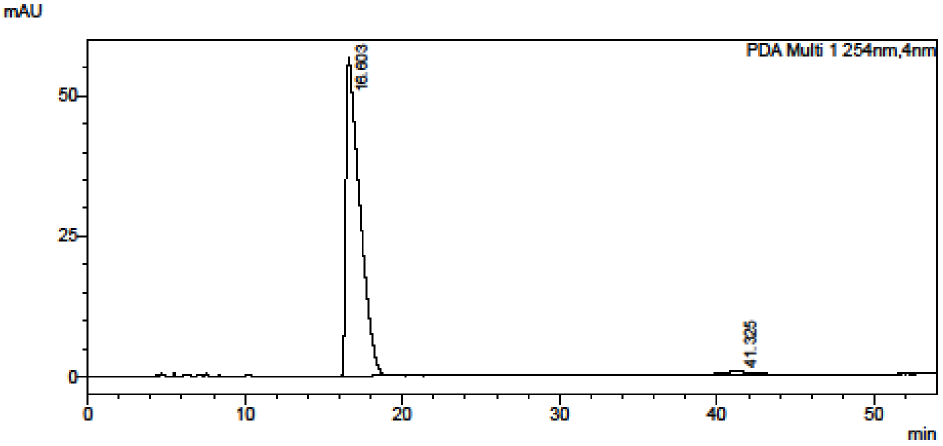

<Peak Table>

PDA Ch1 254nm

Peak#	Ret. Time	Area	Area%	Height
1	16.603	3451656	97.991	56650
2	41.325	70766	2.009	499
Total		3522423	100.000	57149

C:\Andrey\CF3(Ph)-OBnCF3_rac2.lcd

Supplementary figure 235. HPLC chromatogram for compound **6a,** Ligand:**(4R,5S)-7a**

Analysis Report

<Sample Information>

Sample Name	: CF3(Ph)-OBnCF3rac
Sample ID	: CF3(Ph)-OBnCF3rac
Data Filename	: CF3(Ph)-OBnCF3rac4.lcd
Method Filename	: andrey.lcm
Batch Filename	:
Vial #	: 1-50
Injection Volume	: 3 uL
Date Acquired	: 29/05/2017 12:29:41
Date Processed	: 01/06/2017 10:52:16
Sample Type	: Unknown
Acquired by	: System Administrator
Processed by	: System Administrator

<Chromatogram>

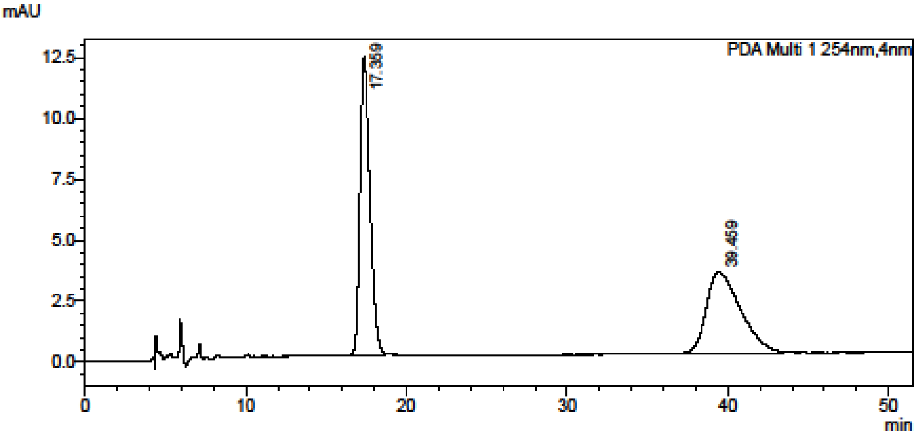

<Peak Table>

PDA Ch1 254nm

Peak#	Ret. Time	Area	Area%	Height
1	17.359	531010	51.844	12244
2	39.459	493232	48.156	3340
Total		1024242	100.000	15584

C:\Andrey\CF3(Ph)-OBnCF3rac4.lcd

Supplementary figure 236. HPLC chromatogram for the compound **6a,** Racemic

Analysis Report

SHIMADZU LabSolutions

<Sample Information>

Sample Name	: CF3(p-MeO)-OBnCF3,Me(4,5diPh)_BOX		
Sample ID	: CF3(p-MeO)-OBnCF3,Me(4,5diPh)_B		
Data Filename	: CF3(p-MeO)-OBnCF3,Me(4,5diPh)_BOX1.lcd		
Method Filename	: new.lcm		
Batch Filename	:		
Vial #	: 1-33	Sample Type	: Unknown
Injection Volume	: 2 uL		
Date Acquired	: 14/03/2017 16:52:42	Acquired by	: System Administrator
Date Processed	: 10/05/2017 16:24:31	Processed by	: System Administrator

<Chromatogram>

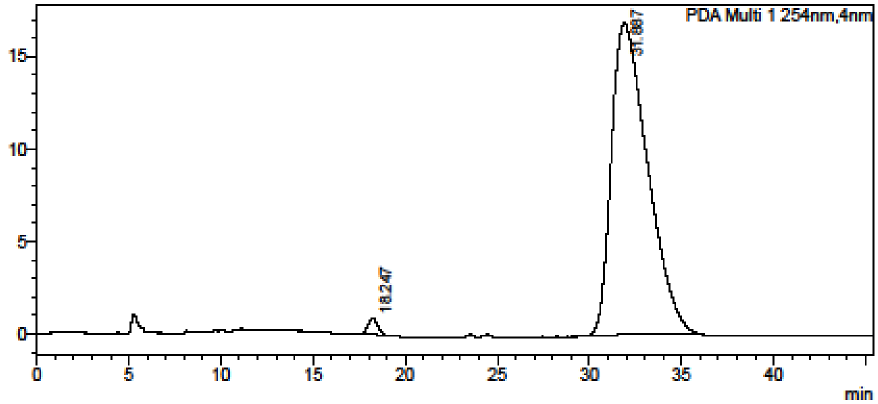

<Peak Table>

PDA Ch1 254nm

Peak#	Ret. Time	Area	Area%	Height
1	18.247	32275	1.382	915
2	31.887	2302353	98.618	16856
Total		2334628	100.000	17771

C:\Andrey\CF3(p-MeO)-OBnCF3,Me(4,5diPh)_BOX1.lcd

Supplementary figure 237. HPLC chromatogram for compound **6b,** Ligand:**(4S,5R)-7a**

Analysis Report

<Sample Information>

Sample Name	: CF3-(p-MeOPh)OBnCF3, Me(4,5diPh)BOX		
Sample ID	: CF3-(p-MeOPh)OBnCF3, Me(4,5diPh		
Data Filename	: CF3-(p-MeOPh)OBnCF3, Me(4,5diPh)BOX2.lcd		
Method Filename	: new.lcm		
Batch Filename	:		
Vial #	: 1-17	Sample Type	: Unknown
Injection Volume	: 4 uL		
Date Acquired	: 07/02/2017 02:17:17	Acquired by	: System Administrator
Date Processed	: 07/02/2017 03:07:18	Processed by	: System Administrator

<Chromatogram>

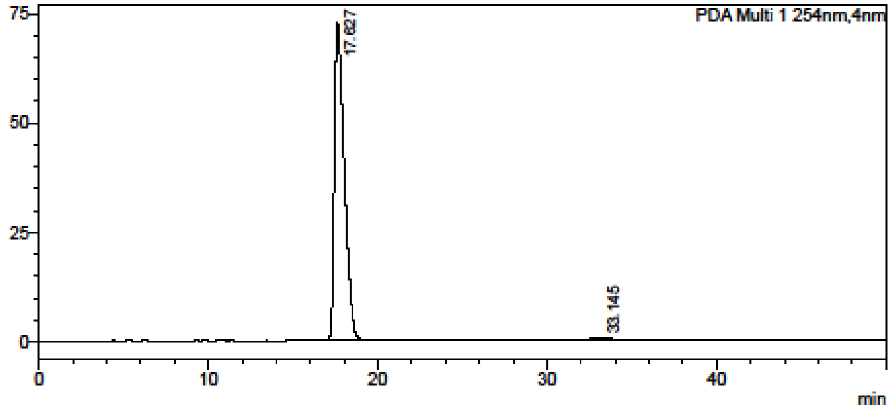

<Peak Table>

PDA Ch1 254nm

Peak#	Ret. Time	Area	Area%	Height
1	17.627	2905052	98.415	72681
2	33.145	46790	1.585	441
Total		2951842	100.000	73122

C:\Andrey\CF3-(p-MeOPh)OBnCF3, Me(4,5diPh)BOX2.lcd

Supplementary figure 238. HPLC chromatogram for compound **6b,** Ligand:**(4R,5S)-7a**

SHIMADZU LabSolutions **Analysis Report**

<Sample Information>

Sample Name	: CF3(p-MeOPh)-OBnCF3_rac
Sample ID	: CF3(p-MeOPh)-OBnCF3_rac
Data Filename	: CF3(p-MeOPh)-OBnCF3_rac1.lcd
Method Filename	: andrey.lcm
Batch Filename	:
Vial #	: 1-85
Injection Volume	: 4 uL
Date Acquired	: 28/06/2017 14:06:51
Date Processed	: 30/06/2017 17:24:26
Sample Type	: Unknown
Acquired by	: System Administrator
Processed by	: System Administrator

<Chromatogram>

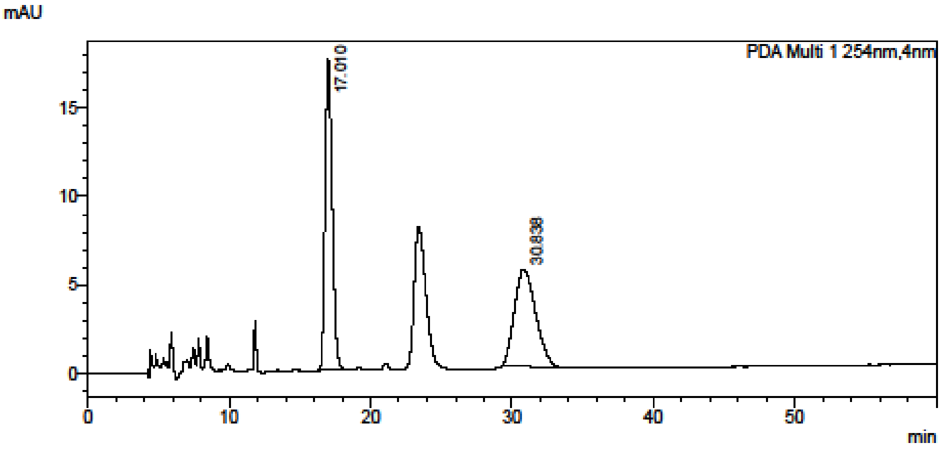

<Peak Table>

PDA Ch1 254nm

Peak#	Ret. Time	Area	Area%	Height
1	17.010	605676	51.352	17509
2	30.838	573779	48.648	5490
Total		1179455	100.000	22998

C:\Andrey\CF3(p-MeOPh)-OBnCF3_rac1.lcd

Supplementary figure 239. HPLC chromatogram for compound **6b,** Racemic

SHIMADZU LabSolutions **Analysis Report**

<Sample Information>

Sample Name	: CF3-(p-FPh)OBnCF3, Me(4,5diPh)BOX		
Sample ID	: CF3-(p-FPh)OBnCF3, Me(4,5diPh)B		
Data Filename	: CF3-(p-FPh)OBnCF3, Me(4,5diPh)BOX1.lcd		
Method Filename	: new.lcm		
Batch Filename	:		
Vial #	: 1-19	Sample Type	: Unknown
Injection Volume	: 4 uL		
Date Acquired	: 09/02/2017 04:30:49	Acquired by	: System Administrator
Date Processed	: 10/02/2017 01:53:46	Processed by	: System Administrator

<Chromatogram>

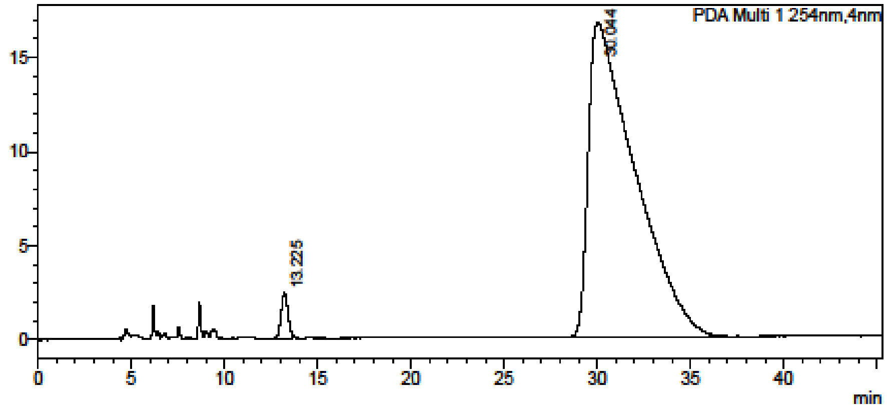

<Peak Table>

PDA Ch1 254nm

Peak#	Ret. Time	Area	Area%	Height
1	13.225	63452	2.132	2410
2	30.044	2913069	97.868	16682
Total		2976521	100.000	19092

C:\Andrey\CF3-(p-FPh)OBnCF3, Me(4,5diPh)BOX1.lcd

Supplementary figure 240. HPLC chromatogram for compound **6c,** Ligand:**(4S,5R)-7a**

SHIMADZU LabSolutions

Analysis Report

<Sample Information>

Sample Name	: CF3(p-F-Ph)-OBnCF3,Me(4,5diPh)BOX		
Sample ID	: CF3(p-F-Ph)-OBnCF3,Me(4,5diPh)B		
Data Filename	: CF3(p-F-Ph)-OBnCF3,Me(4,5diPh)BOX2.lcd		
Method Filename	: new.lcm		
Batch Filename	:		
Vial #	: 1-27	Sample Type	: Unknown
Injection Volume	: 4 uL		
Date Acquired	: 05/03/2017 13:09:46	Acquired by	: System Administrator
Date Processed	: 05/03/2017 13:55:05	Processed by	: System Administrator

<Chromatogram>

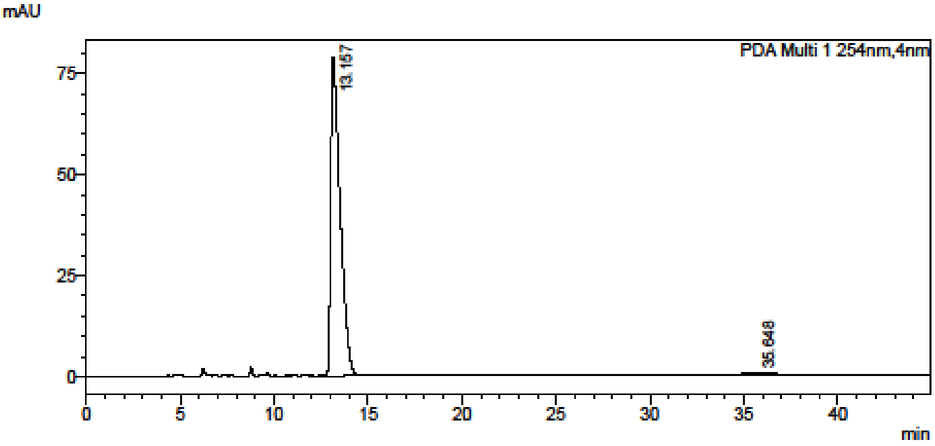

<Peak Table>

PDA Ch1 254nm

Peak#	Ret. Time	Area	Area%	Height
1	13.157	2739781	97.848	78587
2	35.648	60260	2.152	397
Total		2800041	100.000	78984

C:\Andrey\CF3(p-F-Ph)-OBnCF3,Me(4,5diPh)BOX2.lcd

Supplementary figure 241. HPLC chromatogram for compound **6c,** Ligand:**(4R,5S)-7a**

SHIMADZU LabSolutions **Analysis Report**

<Sample Information>

Sample Name	: CF3(p-F-Ph)-OBnCF3_rac		
Sample ID	: CF3(p-F-Ph)-OBnCF3_rac		
Data Filename	: CF3(p-F-Ph)-OBnCF3_rac1.lcd		
Method Filename	: shutdown.lcm		
Batch Filename	:		
Vial #	: 1-62	Sample Type	: Unknown
Injection Volume	: 3 uL		
Date Acquired	: 06/07/2017 13:26:35	Acquired by	: System Administrator
Date Processed	: 09/07/2017 10:01:19	Processed by	: System Administrator

<Chromatogram>

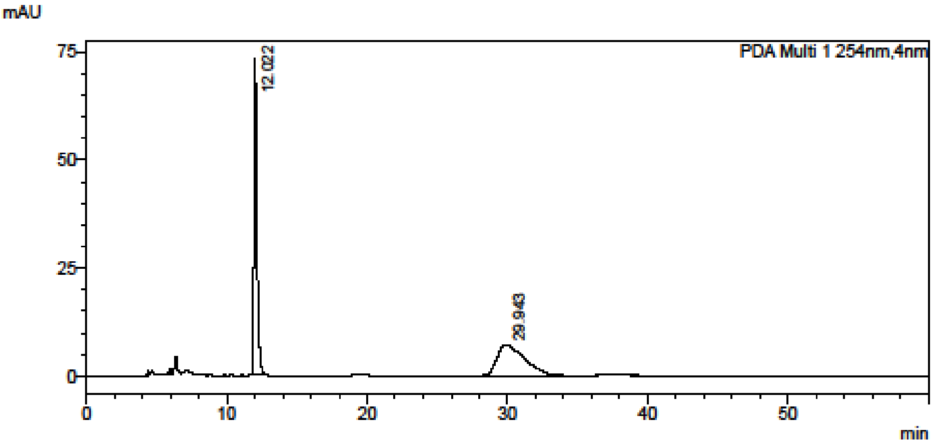

<Peak Table>

PDA Ch1 254nm

Peak#	Ret. Time	Area	Area%	Height
1	12.022	1067534	52.039	73198
2	29.943	983892	47.961	7166
Total		2051425	100.000	80365

C:\Andrey\CF3(p-F-Ph)-OBnCF3_rac1.lcd

Supplementary figure 242. HPLC chromatogram for compound **6c,** Racemic

02/03/2017 13:32:05 Page 1 / 1

Analysis Report

<Sample Information>

Sample Name : CF3-(p-MePh)OBnCF3,Me(4,5diPh)BOX
Sample ID : CF3-(pMePh)OBnCF3, Me(4,5diPh)
Data Filename : CF3-(m-MePh)OBnCF3,Me(4,5diPh)BOX8.lcd
Method Filename : new.lcm
Batch Filename :
Vial # : 1-26 Sample Type : Unknown
Injection Volume : 3 uL
Date Acquired : 02/03/2017 12:28:51 Acquired by : System Administrator
Date Processed : 02/03/2017 13:31:46 Processed by : System Administrator

<Chromatogram>

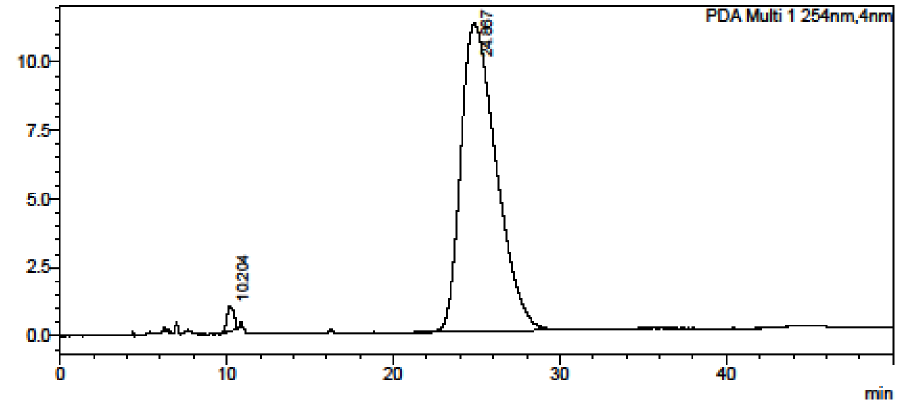

<Peak Table>

PDA Ch1 254nm

Peak#	Ret. Time	Area	Area%	Height
1	10.204	26268	1.551	933
2	24.867	1667052	98.449	11230
Total		1693320	100.000	12163

C:\Andrey\CF3-(m-MePh)OBnCF3,Me(4,5diPh)BOX8.lcd

Supplementary figure 243. HPLC chromatogram for compound **6d,** Ligand:**(4S,5R)-7a**

Analysis Report

<Sample Information>

Sample Name	: CF3-(p-MePh)OBnCF3,Me(4,5diPh)BOX		
Sample ID	: CF3-(p-MePh)OBnCF3, Me(4,5diPh)		
Data Filename	: CF3-(p-MePh)OBnCF3,Me(4,5diPh)BOX3.lcd		
Method Filename	: new.lcm		
Batch Filename	:		
Vial #	: 1-24	Sample Type	: Unknown
Injection Volume	: 2 uL		
Date Acquired	: 13/02/2017 01:59:12	Acquired by	: System Administrator
Date Processed	: 13/02/2017 02:43:09	Processed by	: System Administrator

<Chromatogram>

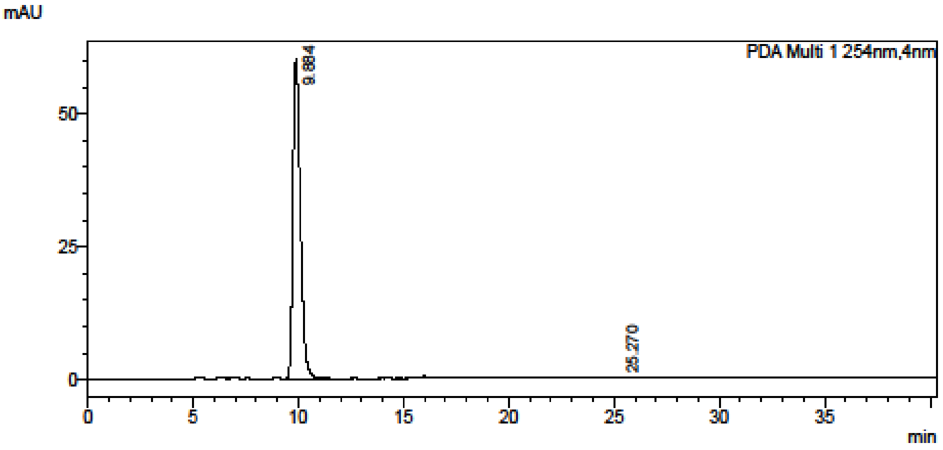

<Peak Table>

PDA Ch1 254nm

Peak#	Ret. Time	Area	Area%	Height
1	9.884	1480178	98.599	60145
2	25.270	21036	1.401	179
Total		1501214	100.000	60323

C:\Andrey\CF3-(p-MePh)OBnCF3,Me(4,5diPh)BOX3.lcd

Supplementary figure 244. HPLC chromatogram for compound **6d,** Ligand:**(4R,5S)-7a**

Analysis Report

<Sample Information>

Sample Name	: CF3(p-MePh)-OBnCF3rac		
Sample ID	: CF3(p-MePh)-OBnCF3rac		
Data Filename	: CF3(p-MePh)-OBnCF3rac5.lcd		
Method Filename	: andrey.lcm		
Batch Filename	:		
Vial #	: 1-51	Sample Type	: Unknown
Injection Volume	: 8 uL		
Date Acquired	: 29/05/2017 13:22:05	Acquired by	: System Administrator
Date Processed	: 01/06/2017 10:52:36	Processed by	: System Administrator

<Chromatogram>

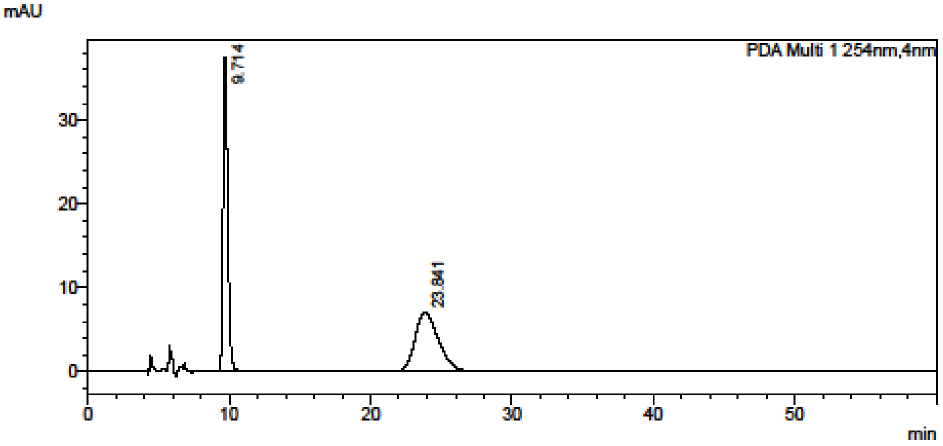

<Peak Table>

PDA Ch1 254nm

Peak#	Ret. Time	Area	Area%	Height
1	9.714	828559	51.130	37325
2	23.841	791945	48.870	6953
Total		1620504	100.000	44278

Supplementary figure 245. HPLC chromatogram for compound **6d,** racemic

10/02/2017 05:28:06 Page 1 / 1

SHIMADZU LabSolutions **Analysis Report**

<Sample Information>

Sample Name	: CF3-(m-MePh)OBnCF3,Me(4,5diPh)BOX		
Sample ID	: CF3-(mPh)OBnCF3, Me(4,5diPh)BOX		
Data Filename	: CF3-(m-Me-Ph)OBnCF3,Me(4,5diPh)BOX1.lcd		
Method Filename	: new.lcm		
Batch Filename	:		
Vial #	: 1-20	Sample Type	: Unknown
Injection Volume	: 2 uL		
Date Acquired	: 10/02/2017 03:11:00	Acquired by	: System Administrator
Date Processed	: 10/02/2017 04:01:02	Processed by	: System Administrator

<Chromatogram>

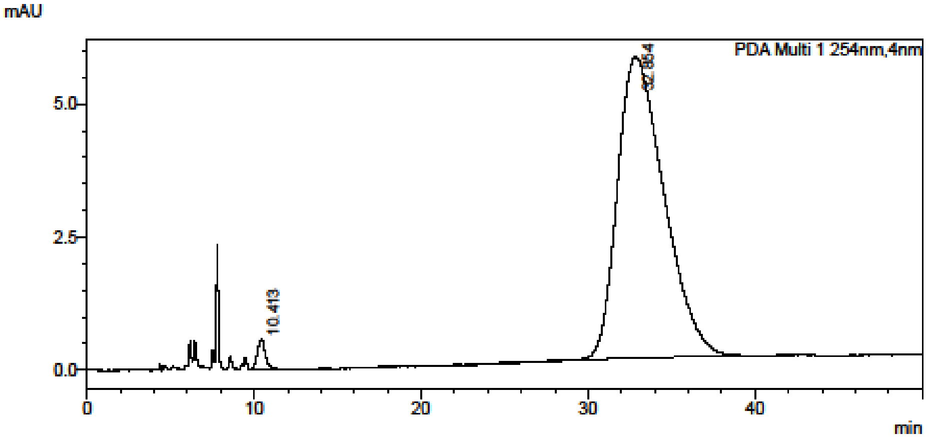

<Peak Table>

PDA Ch1 254nm

Peak#	Ret. Time	Area	Area%	Height
1	10.413	17192	1.538	548
2	32.854	1100550	98.462	5654
Total		1117743	100.000	6202

C:\Andrey\CF3-(m-Me-Ph)OBnCF3,Me(4,5diPh)BOX1.lcd

Supplementary figure 246. HPLC chromatogram for compound **6e,** Ligand:**(4S,5R)-7a**

Analysis Report

<Sample Information>

Sample Name	: CF3-(m-MePh)OBnCF3,Me(4,5diPh)BOX		
Sample ID	: CF3-(mMePh)OBnCF3, Me(4,5diPh)		
Data Filename	: CF3-(m-MePh)OBnCF3,Me(4,5diPh)BOX6.lcd		
Method Filename	: new.lcm		
Batch Filename	:		
Vial #	: 1-26	Sample Type	: Unknown
Injection Volume	: 3 uL		
Date Acquired	: 01/03/2017 13:00:42	Acquired by	: System Administrator
Date Processed	: 01/03/2017 13:49:26	Processed by	: System Administrator

<Chromatogram>

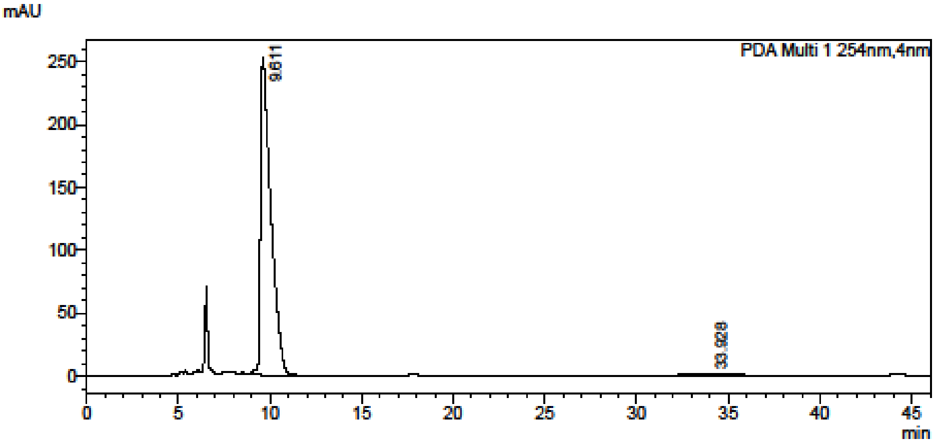

<Peak Table>

PDA Ch1 254nm

Peak#	Ret. Time	Area	Area%	Height
1	9.611	9906670	97.826	251978
2	33.928	220185	2.174	1196
Total		10126854	100.000	253175

C:\Andrey\CF3-(m-MePh)OBnCF3,Me(4,5diPh)BOX6.lcd

Supplementary figure 247. HPLC chromatogram for compound **6e,** Ligand:**(4R,5S)-7a**

Analysis Report

<Sample Information>

Sample Name : CF3(m-MePh)-OBnCF3_rac
Sample ID : CF3(m-MePh)-OBnCF3_rac
Data Filename : CF3(m-MePh)-OBnCF3_rac1.lcd
Method Filename : andrey.lcm
Batch Filename :
Vial # : 1-84
Sample Type : Unknown
Injection Volume : 4 uL
Date Acquired : 28/06/2017 13:22:39
Acquired by : System Administrator
Date Processed : 30/06/2017 17:25:27
Processed by : System Administrator

<Chromatogram>

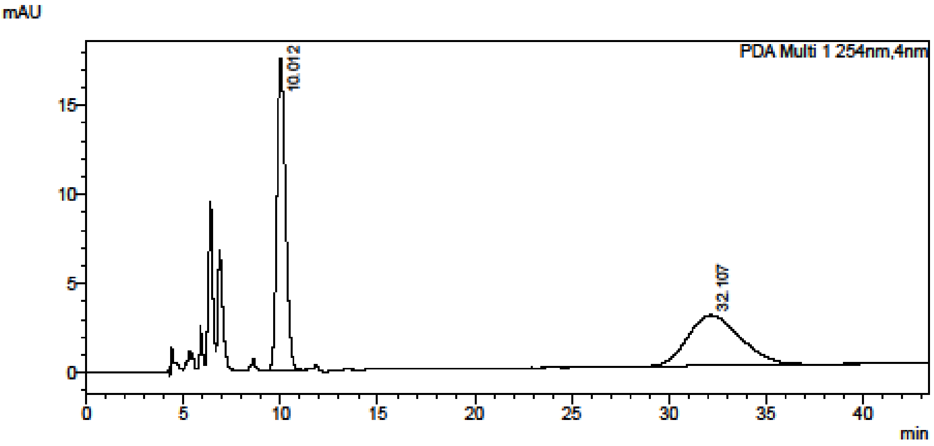

<Peak Table>

PDA Ch1 254nm

Peak#	Ret. Time	Area	Area%	Height
1	10.012	532703	50.063	17420
2	32.107	531373	49.937	2822
Total		1064075	100.000	20242

Supplementary figure 248. HPLC chromatogram for compound **6e,** racemic

SHIMADZU LabSolutions

Analysis Report

<Sample Information>

Sample Name	: CF3(o-Me-Ph)-OBnCF3,Me(4,5diPh)BOX		
Sample ID	: CF3(o-MePh)-OBnCF3,Me(4,5diPh)B		
Data Filename	: CF3(oMePh)-OBnCF3,Me(4,5diPh)BOX3.lcd		
Method Filename	: new.lcm		
Batch Filename	:		
Vial #	: 1-28	Sample Type	: Unknown
Injection Volume	: 4 uL		
Date Acquired	: 05/03/2017 15:28:52	Acquired by	: System Administrator
Date Processed	: 06/03/2017 09:12:12	Processed by	: System Administrator

<Chromatogram>

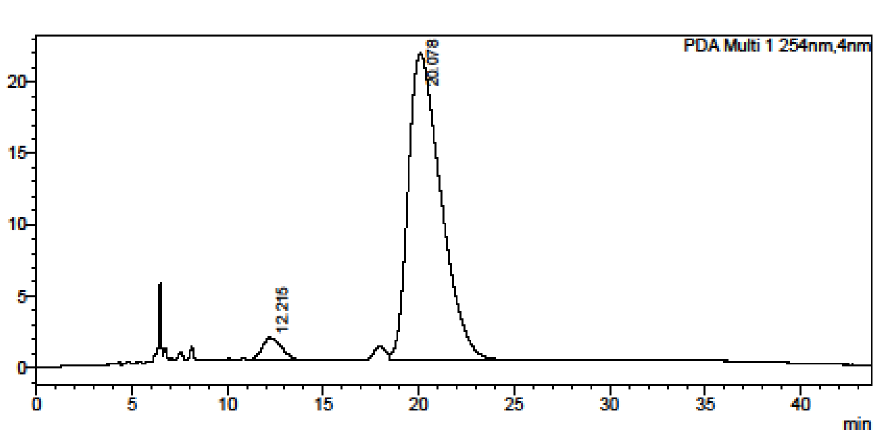

<Peak Table>

PDA Ch1 254nm

Peak#	Ret. Time	Area	Area%	Height
1	12.215	103381	3.895	1547
2	20.078	2550599	96.105	21476
Total		2653980	100.000	23023

C:\Andrey\CF3(oMePh)-OBnCF3,Me(4,5diPh)BOX3.lcd

Supplementary figure 249. HPLC chromatogram for compound **6f,** Ligand:**(4S,5R)-7a**

SHIMADZU LabSolutions

Analysis Report

<Sample Information>

Sample Name	: CF3(o-Me)-OBnCF3,Me(4,5diPh)_BOX		
Sample ID	: CF3(o-Me)-OBnCF3,Me(4,5diPh)_B		
Data Filename	: CF3(o-Me)-OBnCF3,Me(4,5diPh)_BOX2.lcd		
Method Filename	: new.lcm		
Batch Filename	:		
Vial #	: 1-34	Sample Type	: Unknown
Injection Volume	: 2 uL		
Date Acquired	: 14/03/2017 17:39:00	Acquired by	: System Administrator
Date Processed	: 22/03/2017 19:19:21	Processed by	: System Administrator

<Chromatogram>

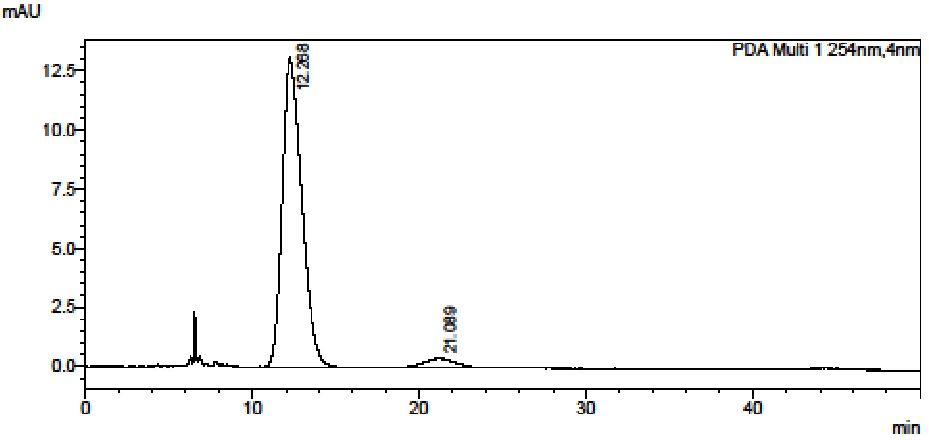

<Peak Table>

PDA Ch1 254nm

Peak#	Ret. Time	Area	Area%	Height
1	12.268	1065882	96.220	13088
2	21.089	41872	3.780	352
Total		1107754	100.000	13440

C:\Andrey\CF3(o-Me)-OBnCF3,Me(4,5diPh)_BOX2.lcd

Supplementary figure 250. HPLC chromatogram for compound **6f,** Ligand:**(4R,5S)-7a**

Analysis Report

<Sample Information>

Sample Name	: CF3(oMe-Ph)OBnCF3 rac		
Sample ID	: CF3(oMe-Ph)OBnCF3 rac		
Data Filename	: CF3(oMe-Ph)OBnCF3 rac3.lcd		
Method Filename	: andrey.lcm		
Batch Filename	:		
Vial #	: 1-91	Sample Type	: Unknown
Injection Volume	: 2 uL		
Date Acquired	: 14/08/2017 11:16:34	Acquired by	: System Administrator
Date Processed	: 14/08/2017 12:04:44	Processed by	: System Administrator

<Chromatogram>

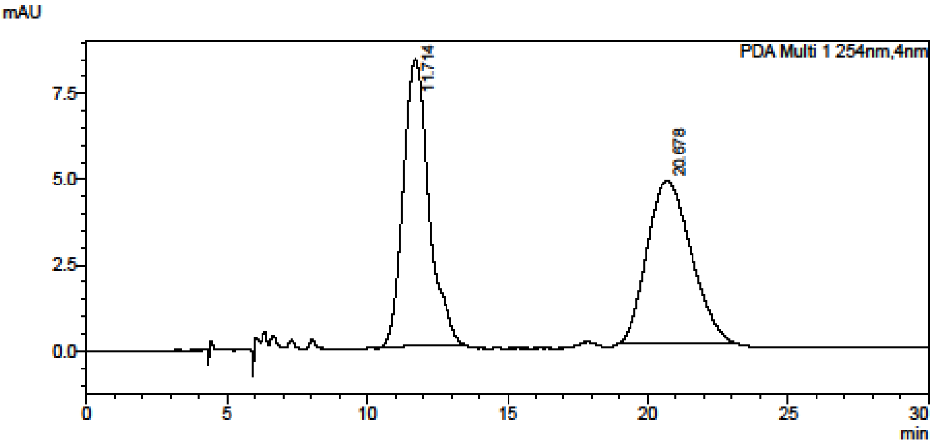

<Peak Table>

PDA Ch1 254nm

Peak#	Ret. Time	Area	Area%	Height
1	11.714	536885	50.819	8374
2	20.678	519579	49.181	4739
Total		1056464	100.000	13113

C:\Andrey\CF3(oMe-Ph)OBnCF3 rac3.lcd

Supplementary figure 251. HPLC chromatogram for compound **6f,** racemic

Analysis Report

<Sample Information>

Sample Name	: CF3-(m-MeSPh)OBnCF3, 4.5diPhMEBOX
Sample ID	: CF3-(m-MeSPh)OBnC, 4.5diPhMEBOX
Data Filename	: CF3-(m-MeSPh)OBnCF3, 4.5diPhMEBOX2.lcd
Method Filename	: new.lcm
Batch Filename	:
Vial #	: 1-4
Injection Volume	: 4 uL
Date Acquired	: 17/01/2017 02:20:05
Date Processed	: 13/08/2017 18:08:25
Sample Type	: Unknown
Acquired by	: System Administrator
Processed by	: System Administrator

<Chromatogram>

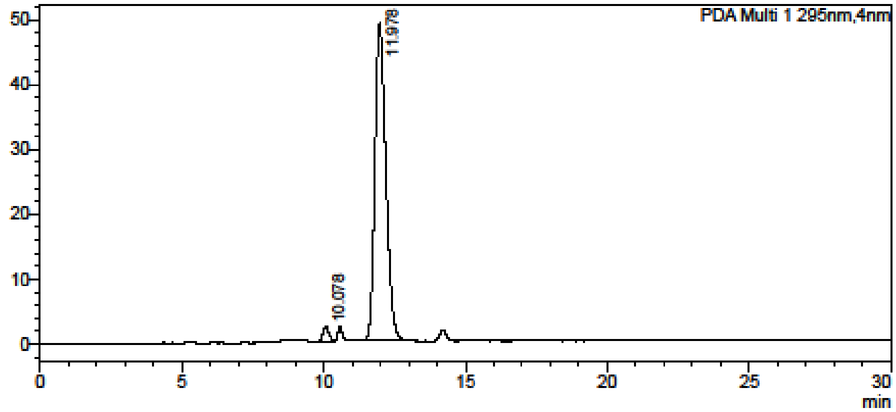

<Peak Table>

PDA Ch1 295nm

Peak#	Ret. Time	Area	Area%	Height
1	10.078	31408	2.291	2229
2	11.978	1339414	97.709	49023
Total		1370822	100.000	51251

C:\Andrey\CF3-(m-MeSPh)OBnCF3, 4.5diPhMEBOX2.lcd

Supplementary figure 252. HPLC chromatogram for compound **6g,** Ligand:**(4S,5R)-7a**

SHIMADZU LabSolutions

Analysis Report

<Sample Information>

Sample Name	: CF3-(m-MeSPh)OBnCF3,Me(4,5diPh)BOX
Sample ID	: CF3-(mMeSPh)OBnCF3, Me(4,5diPh)
Data Filename	: CF3-(m-MeSPh)OBnCF3,Me(4,5diPh)BOX4.lcd
Method Filename	: new.lcm
Batch Filename	:
Vial #	: 1-25
Sample Type	: Unknown
Injection Volume	: 3 uL
Date Acquired	: 13/02/2017 04:02:40
Acquired by	: System Administrator
Date Processed	: 13/02/2017 04:34:07
Processed by	: System Administrator

<Chromatogram>

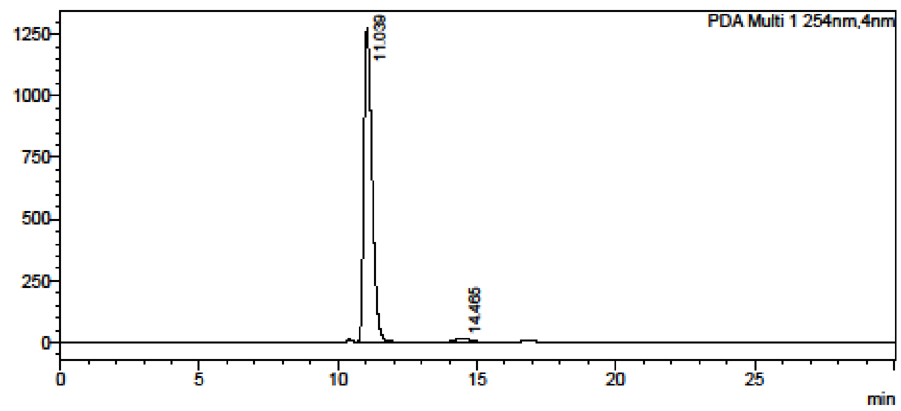

<Peak Table>

PDA Ch1 254nm

Peak#	Ret. Time	Area	Area%	Height
1	11.039	25923074	97.728	1270475
2	14.465	602792	2.272	16467
Total		26525866	100.000	1286942

Supplementary figure 253. HPLC chromatogram for compound **6g,** Ligand:**(4R,5S)-7a**

Analysis Report

<Sample Information>

Sample Name	: CF3(m-MeSPh)OBnCF3 _rac		
Sample ID	: CF3(m-MeSPh)OBnCF3 _rac		
Data Filename	: CF3(m-MeSPh)OBnCF3 _rac2.lcd		
Method Filename	: andrey.lcm		
Batch Filename	:		
Vial #	: 1-94	Sample Type	: Unknown
Injection Volume	: 1 uL		
Date Acquired	: 16/08/2017 15:34:17	Acquired by	: System Administrator
Date Processed	: 21/08/2017 17:21:04	Processed by	: System Administrator

<Chromatogram>

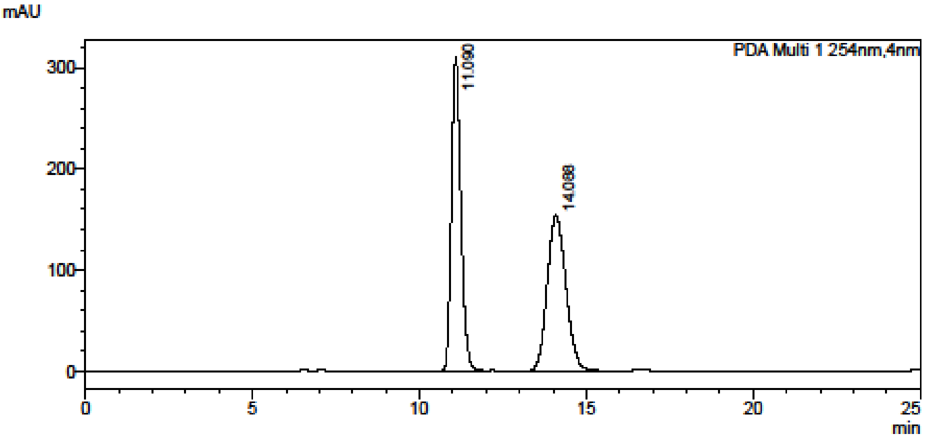

<Peak Table>

PDA Ch1 254nm

Peak#	Ret. Time	Area	Area%	Height
1	11.090	6085880	50.206	309750
2	14.088	6036038	49.794	154163
Total		12121918	100.000	463913

Supplementary figure 254. HPLC chromatogram for compound **6g,** racemic

Analysis Report

<Sample Information>

Sample Name	: CF3(m-OCF3Ph)-OBnCF3 (diPh)BOX old		
Sample ID	: CF3(m-OCF3Ph)-OBnCF3 (diPh)BOX		
Data Filename	: CF3(m-OCF3Ph)-OBnCF3 (diPh)BOX old1.lcd		
Method Filename	: andrey.lcm		
Batch Filename	:		
Vial #	: 1-56	Sample Type	: Unknown
Injection Volume	: 5 uL		
Date Acquired	: 05/06/2017 18:50:14	Acquired by	: System Administrator
Date Processed	: 21/08/2017 15:28:34	Processed by	: System Administrator

<Chromatogram>

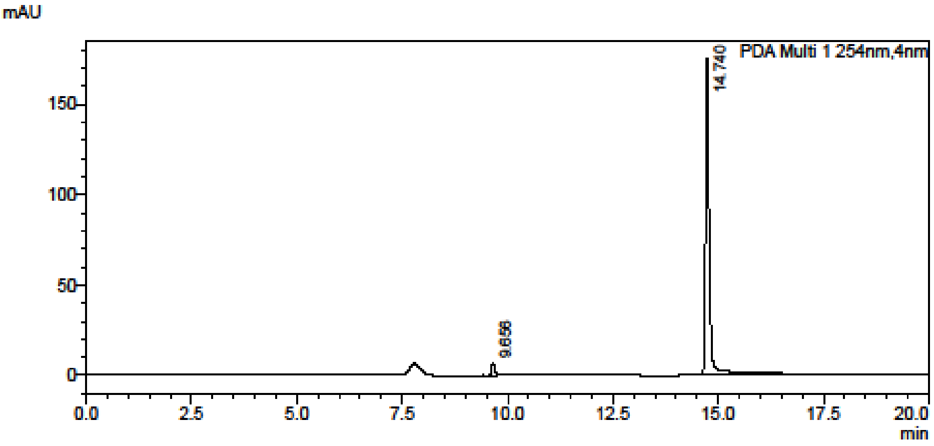

<Peak Table>

PDA Ch1 254nm

Peak#	Ret. Time	Area	Area%	Height
1	9.656	48496	4.051	6901
2	14.740	1148754	95.949	175108
Total		1197250	100.000	182009

C:\Andrey\CF3(m-OCF3Ph)-OBnCF3 (diPh)BOX old1.lcd

Supplementary figure 255. HPLC chromatogram for compound **6h,** Ligand:**(4S,5R)-7a**

SHIMADZU LabSolutions

Analysis Report

<Sample Information>

Sample Name	: CF3(m-CF3O-Ph)-OBnCF3,Me(4,5diPh)BOX		
Sample ID	: CF3(m-CF3O-Ph)-OBnCF3,Me(4,5diP		
Data Filename	: CF3(m-CF3O-Ph)-OBnCF3,Me(4,5diPh)BOX5.lcd		
Method Filename	: new.lcm		
Batch Filename	:		
Vial #	: 1-29	Sample Type	: Unknown
Injection Volume	: 4 uL		
Date Acquired	: 07/03/2017 17:36:52	Acquired by	: System Administrator
Date Processed	: 08/03/2017 13:21:07	Processed by	: System Administrator

<Chromatogram>

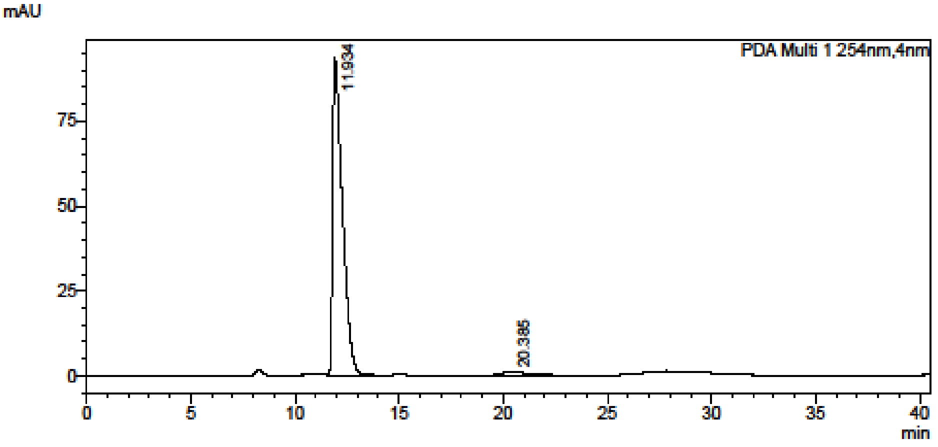

<Peak Table>

PDA Ch1 254nm

Peak#	Ret. Time	Area	Area%	Height
1	11.934	3016018	96.838	93438
2	20.385	98478	3.162	1093
Total		3114496	100.000	94531

C:\Andrey\CF3(m-CF3O-Ph)-OBnCF3,Me(4,5diPh)BOX5.lcd

Supplementary figure 256. HPLC chromatogram for compound **6h,** Ligand:**(4R,5S)-7a**

SHIMADZU LabSolutions **Analysis Report**

<Sample Information>

Sample Name	: CF3(m-OCF3)-OBnCF3 rac		
Sample ID	: CF3(m-OCF3)-OBnCF3 rac		
Data Filename	: CF3(m-OCF3)-OBnCF3 rac4.lcd		
Method Filename	: andrey.lcm		
Batch Filename	:		
Vial #	: 1-64	Sample Type	: Unknown
Injection Volume	: 2 uL		
Date Acquired	: 06/07/2017 20:11:48	Acquired by	: System Administrator
Date Processed	: 09/07/2017 10:37:24	Processed by	: System Administrator

<Chromatogram>

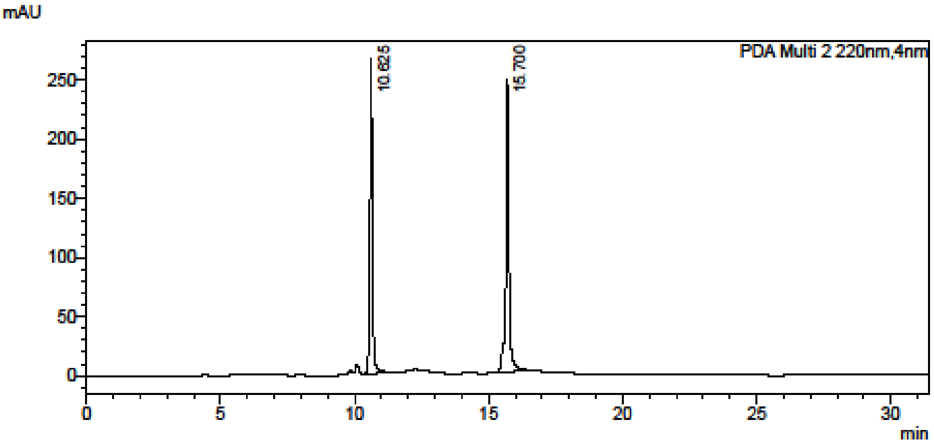

<Peak Table>

PDA Ch2 220nm

Peak#	Ret. Time	Area	Area%	Height
1	10.625	1856527	46.387	265953
2	15.700	2145722	53.613	247095
Total		4002250	100.000	513048

C:\Andrey\CF3(m-OCF3)-OBnCF3 rac4.lcd

Supplementary figure 257. HPLC chromatogram for compound **6h,** racemic

SHIMADZU LabSolutions

Analysis Report

<Sample Information>

Sample Name	: CF3-(2-Napht)OBnCF3, 4.5diPhMEBOX		
Sample ID	: CF3-(2-Napht)OBnC, 4.5diPhMEBOX		
Data Filename	: CF3-(2-Napht)OBnCF3, 4.5diPhMEBOX2.lcd		
Method Filename	: new.lcm		
Batch Filename	:		
Vial #	: 1-4	Sample Type	: Unknown
Injection Volume	: 2 uL		
Date Acquired	: 11/01/2017 16:34:54	Acquired by	: System Administrator
Date Processed	: 21/08/2017 15:22:12	Processed by	: System Administrator

<Chromatogram>

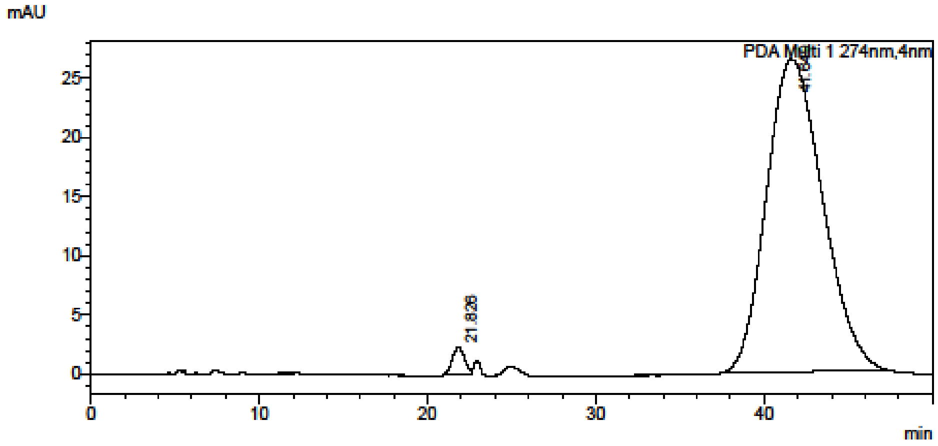

<Peak Table>

PDA Ch1 274nm

Peak#	Ret. Time	Area	Area%	Height
1	21.826	122036	1.952	2372
2	41.649	6130344	98.048	26421
Total		6252380	100.000	28793

C:\Andrey\CF3-(2-Napht)OBnCF3, 4.5diPhMEBOX2.lcd

Supplementary figure 258. HPLC chromatogram for compound **6i,** Ligand:**(4S,5R)-7a**

Analysis Report

<Sample Information>

Sample Name	: CF3(2-Napht)-OBnCF3,Me(4.5diPh) old
Sample ID	: CF3(2-Napht)-OBnCF3,Me(4.5diPh)
Data Filename	: CF3(2-Napht)-OBnCF3,Me(4.5diPh) old1.lcd
Method Filename	: andrey.lcm
Batch Filename	:
Vial #	: 1-41
Injection Volume	: 5 uL
Date Acquired	: 20/04/2017 16:44:57
Date Processed	: 20/04/2017 18:07:47
Sample Type	: Unknown
Acquired by	: System Administrator
Processed by	: System Administrator

<Chromatogram>

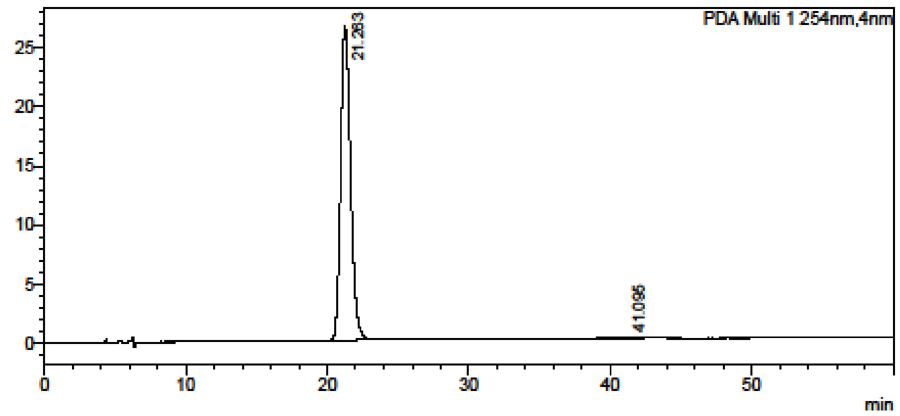

<Peak Table>

PDA Ch1 254nm

Peak#	Ret. Time	Area	Area%	Height
1	21.263	1289315	97.956	26502
2	41.095	26909	2.044	151
Total		1316224	100.000	26652

Supplementary figure 259. HPLC chromatogram for compound **6i,** Ligand:**(4R,5S)-7a**

SHIMADZU LabSolutions

Analysis Report

<Sample Information>

Sample Name	: CF3(napht)-OBnCF3_rac		
Sample ID	: CF3(napht)-OBnCF3_rac		
Data Filename	: CF3(napht)-OBnCF3_rac2.lcd		
Method Filename	: shutdown.lcm		
Batch Filename	:		
Vial #	: 1-61	Sample Type	: Unknown
Injection Volume	: 2 uL		
Date Acquired	: 06/07/2017 12:35:37	Acquired by	: System Administrator
Date Processed	: 09/07/2017 10:02:37	Processed by	: System Administrator

<Chromatogram>

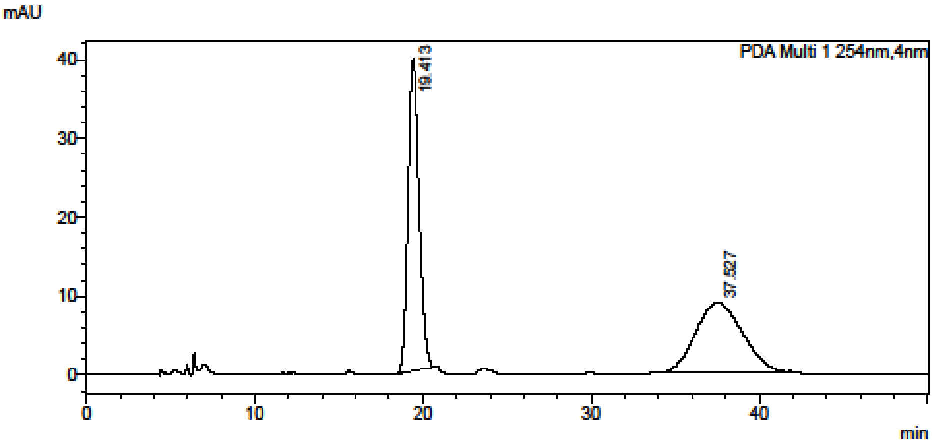

<Peak Table>

PDA Ch1 254nm

Peak#	Ret. Time	Area	Area%	Height
1	19.413	1850403	51.784	39514
2	37.527	1722902	48.216	8829
Total		3573305	100.000	48343

C:\Andrey\CF3(napht)-OBnCF3_rac2.lcd

Supplementary figure 260. HPLC chromatogram for compound **6i,** racemic

SHIMADZU LabSolutions **Analysis Report**

<Sample Information>

Sample Name	: CF3-(p-vinPh)OBnCF3, 4.5diPhMEBOX
Sample ID	: CF3-(p-vPh)OBnCF3, 4.5diPhMEBOX
Data Filename	: CF3-(p-vinPh)OBnCF3, 4.5diPhMEBOX6.lcd
Method Filename	: new.lcm
Batch Filename	:
Vial #	: 1-3
Sample Type	: Unknown
Injection Volume	: 2 uL
Date Acquired	: 05/01/2017 19:36:13
Acquired by	: System Administrator
Date Processed	: 22/08/2017 15:18:35
Processed by	: System Administrator

<Chromatogram>

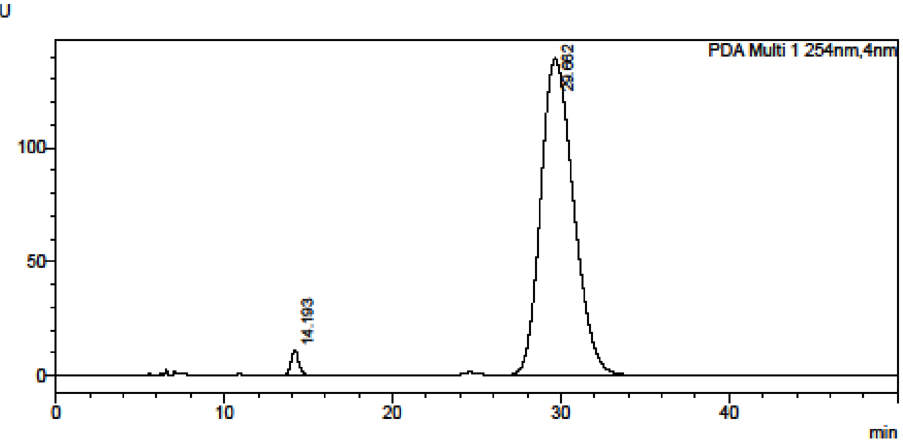

<Peak Table>

PDA Ch1 254nm

Peak#	Ret. Time	Area	Area%	Height
1	14.193	347576	1.807	11003
2	29.662	18892586	98.193	139231
Total		19240162	100.000	150234

C:\Andrey\CF3-(p-vinPh)OBnCF3, 4.5diPhMEBOX6.lcd

Supplementary figure 261. HPLC chromatogram for compound **6j,** Ligand:**(4S,5R)-7a**

SHIMADZU LabSolutions

Analysis Report

<Sample Information>

Sample Name	: CF3(vinPh)-OBnCF3, 4.5diPhBOXold		
Sample ID	: CF3(vinPh)-OBnCF3, 4.5diPhBOXol		
Data Filename	: CF3(vinPh)-OBnCF3, 4.5diPhBOXold2.lcd		
Method Filename	: andrey.lcm		
Batch Filename	:		
Vial #	: 1-45	Sample Type	: Unknown
Injection Volume	: 4 uL		
Date Acquired	: 07/05/2017 14:46:42	Acquired by	: System Administrator
Date Processed	: 09/05/2017 18:50:42	Processed by	: System Administrator

<Chromatogram>

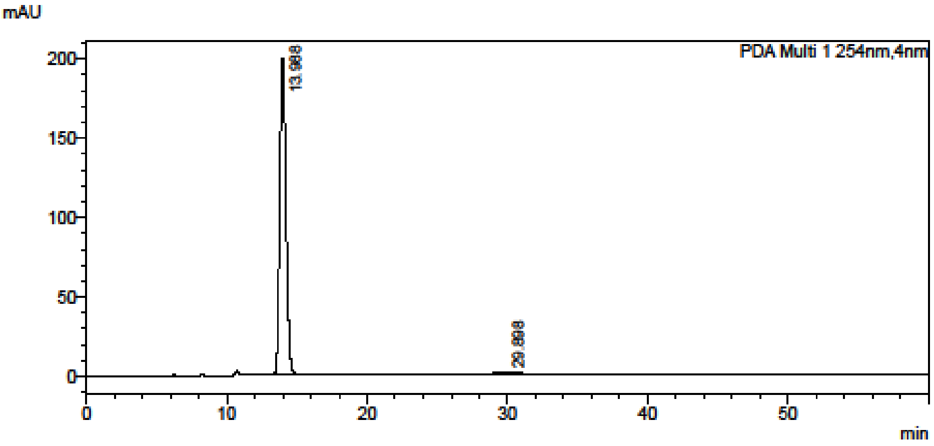

<Peak Table>

PDA Ch1 254nm

Peak#	Ret. Time	Area	Area%	Height
1	13.988	5962161	97.209	199085
2	29.898	171204	2.791	1358
Total		6133366	100.000	200443

C:\Andrey\CF3(vinPh)-OBnCF3, 4.5diPhBOXold2.lcd

Supplementary figure 262. HPLC chromatogram for compound **6j,** Ligand:**(4R,5S)-7a**

Analysis Report

<Sample Information>

Sample Name : CF3(4-vinPh)OBnCF3_rac
Sample ID : CF3(4-vinPh)OBnCF3_rac
Data Filename : CF3(4-vinPh)OBnCF3_rac2.lcd
Method Filename : andrey.lcm
Batch Filename :
Vial # : 1-69
Sample Type : Unknown
Injection Volume : 1 uL
Date Acquired : 22/08/2017 15:41:07
Acquired by : System Administrator
Date Processed : 22/08/2017 19:05:41
Processed by : System Administrator

<Chromatogram>

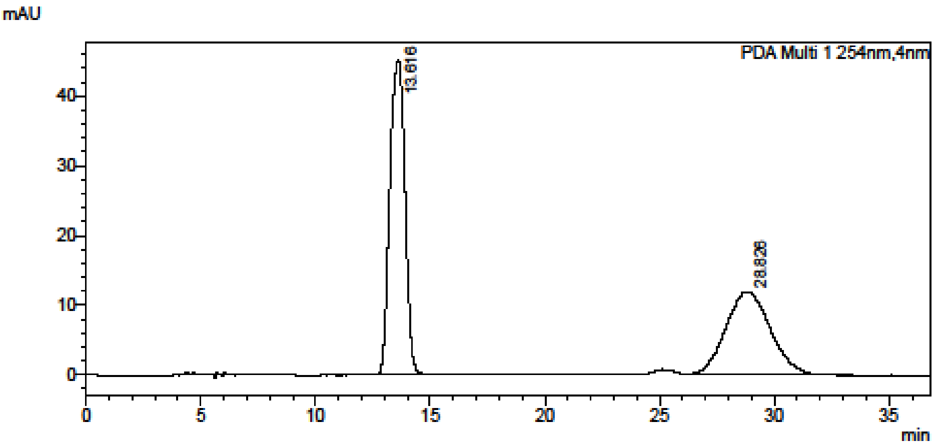

<Peak Table>

PDA Ch1 254nm

Peak#	Ret. Time	Area	Area%	Height
1	13.616	1688406	50.805	40406
2	28.826	1634886	49.195	11960
Total		3323292	100.000	52366

Supplementary figure 263. HPLC chromatogram for compound **6j,** racemic

Analysis Report

<Sample Information>

Sample Name	: CF3(3-(1,3-dioxalen-2-yl)Ph)OBnCF3		
Sample ID	: CF3(3-(1,3-dioxalen-2-yl)Ph)OBn		
Data Filename	: CF3(3-(1,3-dioxalen-2-yl)Ph)OBnCF3__4.lcd		
Method Filename	: andrey.lcm		
Batch Filename	:		
Vial #	: 1-84	Sample Type	: Unknown
Injection Volume	: 2 uL		
Date Acquired	: 07/05/2018 15:54:22	Acquired by	: System Administrator
Date Processed	: 07/05/2018 16:38:16	Processed by	: System Administrator

<Chromatogram>

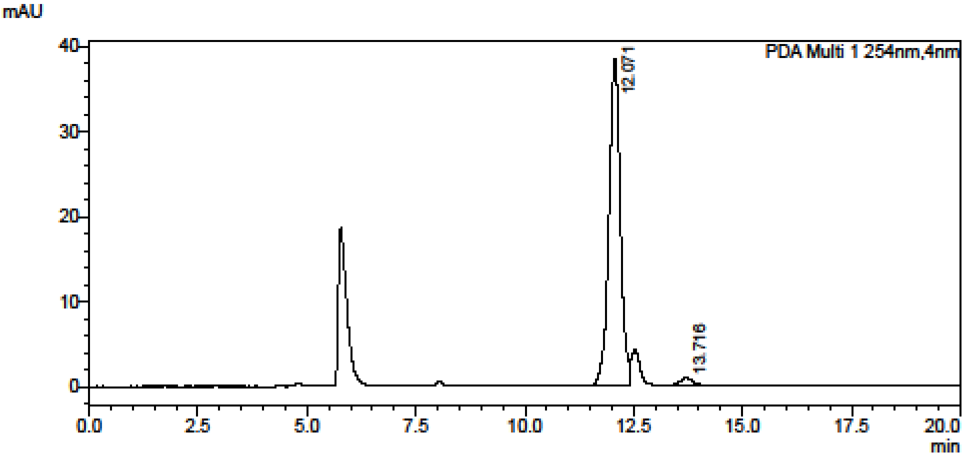

<Peak Table>

PDA Ch1 254nm

Peak#	Ret. Time	Area	Area%	Height
1	12.071	694846	97.307	38253
2	13.716	19232	2.693	890
Total		714078	100.000	39143

C:\Andrey\CF3(3-(1,3-dioxalen-2-yl)Ph)OBnCF3__4.lcd

Supplementary figure 264. HPLC chromatogram for compound **6k,** Ligand:**(4R,5S)-7a**

SHIMADZU LabSolutions **Analysis Report**

<Sample Information>

Sample Name	: CF3(3-(1,3-dioxalen-2-yl)Ph)OBnCF3		
Sample ID	: CF3(3-(1,3-dioxalen-2-yl)Ph)OBn		
Data Filename	: CF3(3-(1,3-dioxalen-2-yl)Ph)OBnCF3_1.lcd		
Method Filename	: andrey.lcm		
Batch Filename	:		
Vial #	: 1-67	Sample Type	: Unknown
Injection Volume	: 2 uL		
Date Acquired	: 29/04/2018 19:51:09	Acquired by	: System Administrator
Date Processed	: 07/05/2018 16:35:13	Processed by	: System Administrator

<Chromatogram>

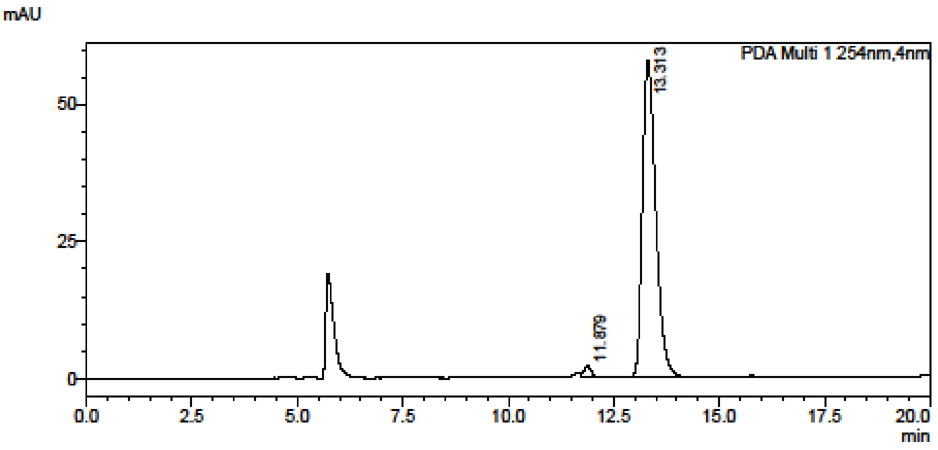

<Peak Table>

PDA Ch1 254nm

Peak#	Ret. Time	Area	Area%	Height
1	11.879	26818	2.081	2059
2	13.313	1261879	97.919	57682
Total		1288697	100.000	59741

C:\Andrey\CF3(3-(1,3-dioxalen-2-yl)Ph)OBnCF3_1.lcd

Supplementary figure 265. HPLC chromatogram for compound **6k**, Ligand:**(4S,5R)-7a**

Analysis Report

<Sample Information>

Sample Name : CF3(3-(1,3-dioxalen-2-yl)Ph)OBnCF3
Sample ID : CF3(3-(1,3-dioxalen-2-yl)Ph)OBn
Data Filename : CF3(3-(1,3-dioxalen-2-yl)Ph)OBnCF3__1.lcd
Method Filename : andrey.lcm
Batch Filename :
Vial # : 1-79 Sample Type : Unknown
Injection Volume : 2 uL
Date Acquired : 06/05/2018 14:37:50 Acquired by : System Administrator
Date Processed : 07/05/2018 16:37:14 Processed by : System Administrator

<Chromatogram>

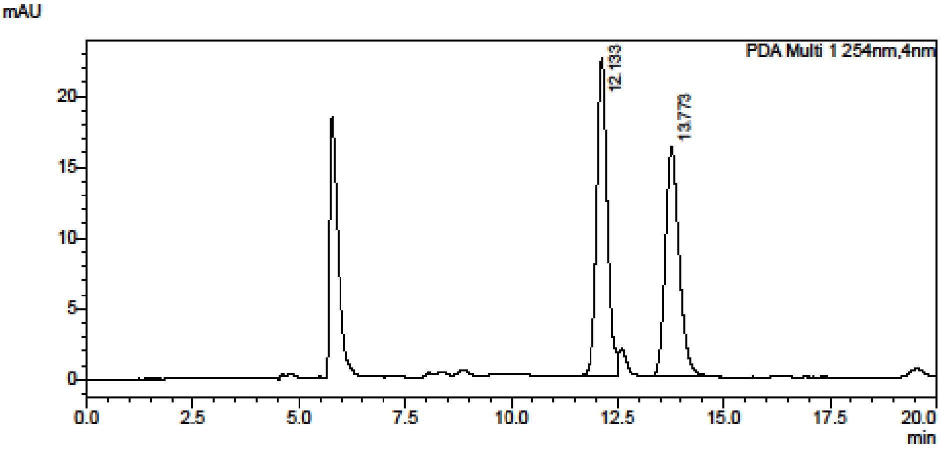

<Peak Table>

PDA Ch1 254nm

Peak#	Ret. Time	Area	Area%	Height
1	12.133	386366	50.557	22416
2	13.773	377848	49.443	16329
Total		764215	100.000	38745

Supplementary figure 266. HPLC chromatogram for compound **6k**, Ligand: racemic

SHIMADZU LabSolutions

Analysis Report

<Sample Information>

Sample Name	: CF3(m-CO2MePh)OBnCF3		
Sample ID	: CF3(m-CO2MePh)OBnCF3		
Data Filename	: CF3(m-CO2MePh)OBnCF3_r6.lcd		
Method Filename	: andrey.lcm		
Batch Filename	:		
Vial #	: 1-99	Sample Type	: Unknown
Injection Volume	: 1 uL		
Date Acquired	: 23/05/2018 15:35:24	Acquired by	: System Administrator
Date Processed	: 23/05/2018 20:18:50	Processed by	: System Administrator

<Chromatogram>

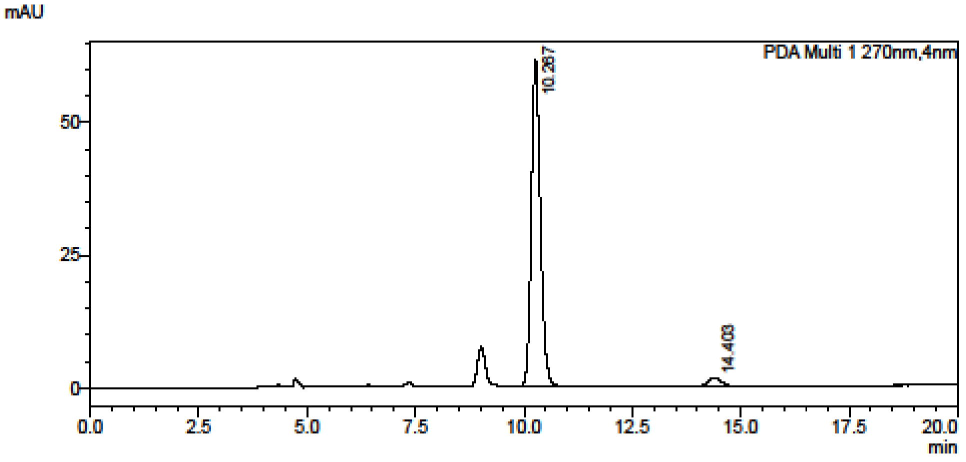

<Peak Table>

PDA Ch1 270nm

Peak#	Ret. Time	Area	Area%	Height
1	10.267	886989	96.662	61283
2	14.403	30630	3.338	1506
Total		917619	100.000	62789

C:\Andrey\CF3(m-CO2MePh)OBnCF3_r6.lcd

Supplementary figure 267. HPLC chromatogram for compound **6m**, Ligand: Ligand:**(4R,5S)-7a**

Analysis Report

<Sample Information>

Sample Name : CF3(m-CO2MePh)OBnCF3
Sample ID : CF3(m-CO2MePh)OBnCF3
Data Filename : CF3(m-CO2MePh)OBnCF3_s5.lcd
Method Filename : andrey.lcm
Batch Filename :
Vial # : 1-100 Sample Type : Unknown
Injection Volume : 1 uL
Date Acquired : 23/05/2018 15:05:34 Acquired by : System Administrator
Date Processed : 23/05/2018 20:16:47 Processed by : System Administrator

<Chromatogram>

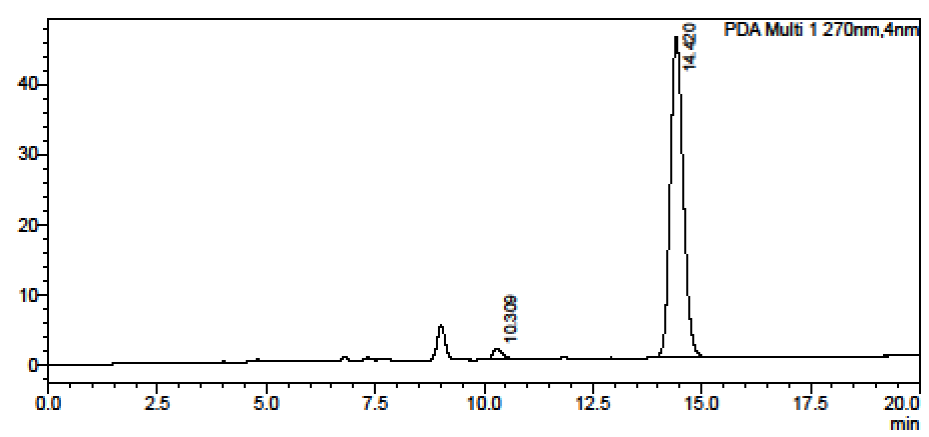

<Peak Table>

PDA Ch1 270nm

Peak#	Ret. Time	Area	Area%	Height
1	10.309	25902	2.666	1665
2	14.420	945834	97.334	45633
Total		971736	100.000	47299

C:\Andrey\CF3(m-CO2MePh)OBnCF3_s5.lcd

Supplementary figure 268. HPLC chromatogram for compound **6m**, Ligand: Ligand:**(4S,5R)-7a**

SHIMADZU LabSolutions **Analysis Report**

<Sample Information>

Sample Name	: CF3(m-CO2MePh)OBnCF3		
Sample ID	: CF3(m-CO2MePh)OBnCF3		
Data Filename	: CF3(m-CO2MePh)OBnCF3_rac1.lcd		
Method Filename	: andrey.lcm		
Batch Filename	:		
Vial #	: 1-101	Sample Type	: Unknown
Injection Volume	: 1 uL		
Date Acquired	: 23/05/2018 15:56:07	Acquired by	: System Administrator
Date Processed	: 23/05/2018 20:19:25	Processed by	: System Administrator

<Chromatogram>

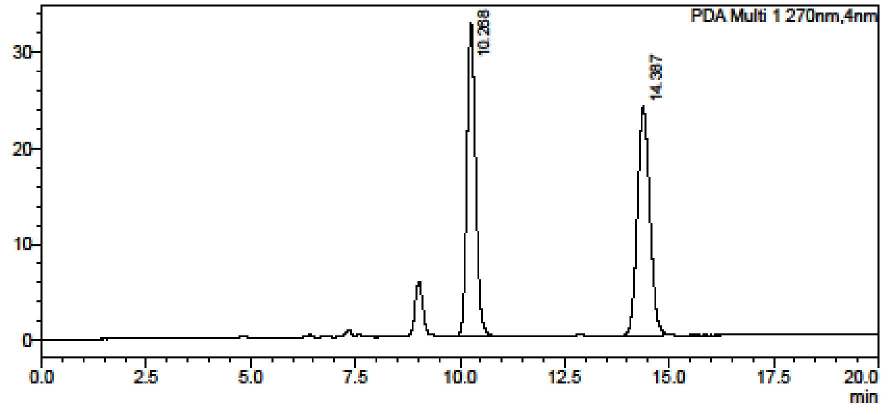

<Peak Table>

PDA Ch1 270nm

Peak#	Ret. Time	Area	Area%	Height
1	10.268	468728	48.745	32652
2	14.387	492868	51.255	23883
Total		961596	100.000	56535

C:\Andrey\CF3(m-CO2MePh)OBnCF3_rac1.lcd

Supplementary figure 269. HPLC chromatogram for compound **6m**, Ligand: racemic

SHIMADZU LabSolutions

Analysis Report

<Sample Information>

Sample Name	: CF3(p-Me2NPh)OBnCF3		
Sample ID	: CF3(p-Me2NPh)OBnCF3		
Data Filename	: CF3(p-Me2NPh)OBnCF3_r6.lcd		
Method Filename	: andrey.lcm		
Batch Filename	:		
Vial #	: 1-99	Sample Type	: Unknown
Injection Volume	: 1 uL		
Date Acquired	: 27/05/2018 13:33:40	Acquired by	: System Administrator
Date Processed	: 27/05/2018 20:05:10	Processed by	: System Administrator

<Chromatogram>

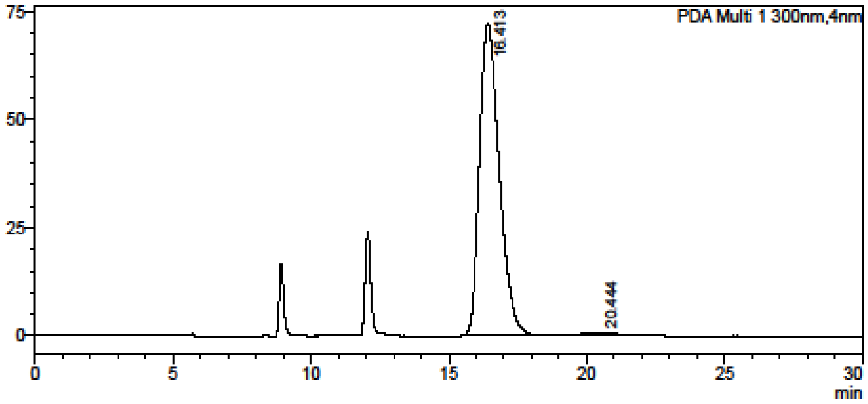

<Peak Table>

PDA Ch1 300nm

Peak#	Ret. Time	Area	Area%	Height
1	16.413	3612026	98.151	72168
2	20.444	68055	1.849	824
Total		3680081	100.000	72992

Supplementary figure 270. HPLC chromatogram for compound **6n**, Ligand: Ligand:**(4R,5S)-7a**

Analysis Report

<Sample Information>

Sample Name	: CF3(p-Me2NPh)OBnCF3		
Sample ID	: CF3(p-Me2NPh)OBnCF3		
Data Filename	: CF3(p-Me2NPh)OBnCF3_s2.lcd		
Method Filename	: andrey.lcm		
Batch Filename	:		
Vial #	: 1-100	Sample Type	: Unknown
Injection Volume	: 1 uL		
Date Acquired	: 27/05/2018 11:23:21	Acquired by	: System Administrator
Date Processed	: 27/05/2018 20:03:56	Processed by	: System Administrator

<Chromatogram>

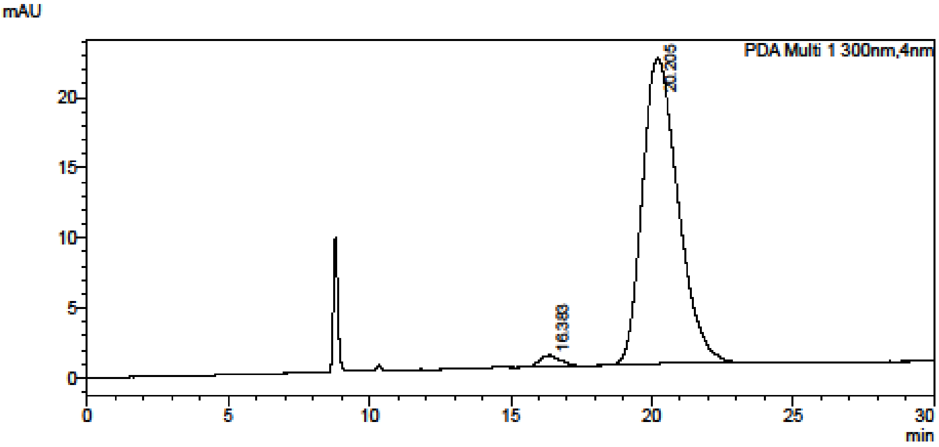

<Peak Table>

PDA Ch1 300nm

Peak#	Ret. Time	Area	Area%	Height
1	16.383	34524	1.770	724
2	20.205	1916105	98.230	21782
Total		1950629	100.000	22506

C:\Andrey\CF3(p-Me2NPh)OBnCF3_s2.lcd

Supplementary figure 271. HPLC chromatogram for compound **6n**, Ligand: Ligand:**(4S,5R)-7a**

Analysis Report

<Sample Information>

Sample Name : CF3(p-Me2NPh)OBnCF3
Sample ID : CF3(p-Me2NPh)OBnCF3
Data Filename : CF3(p-Me2NPh)OBnCF3_rac6.lcd
Method Filename : andrey.lcm
Batch Filename :
Vial # : 1-101 Sample Type : Unknown
Injection Volume : 1 uL
Date Acquired : 27/05/2018 18:36:19 Acquired by : System Administrator
Date Processed : 27/05/2018 20:03:41 Processed by : System Administrator

<Chromatogram>

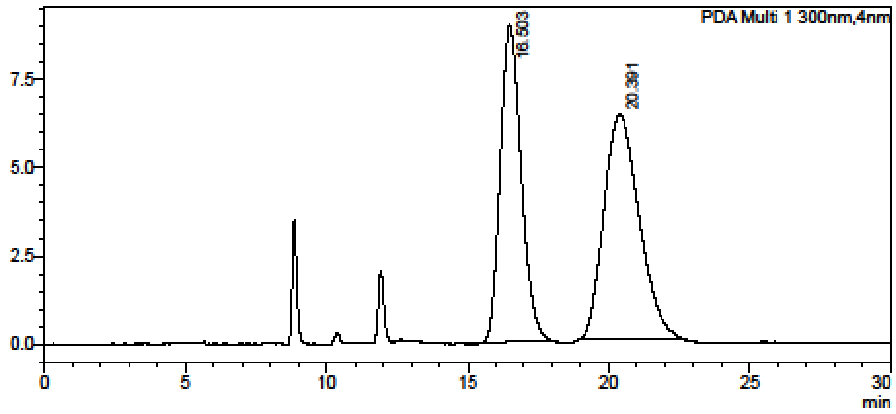

<Peak Table>

PDA Ch1 300nm

Peak#	Ret. Time	Area	Area%	Height
1	16.503	465699	45.831	8967
2	20.391	550421	54.169	6352
Total		1016121	100.000	15318

Supplementary figure 272. HPLC chromatogram for compound **6n**, Ligand: racemic

SHIMADZU LabSolutions **Analysis Report**

<Sample Information>

Sample Name	: CF3(3-thienyl)OBnCF3		
Sample ID	: CF3(3-thienyl)OBnCF3		
Data Filename	: CF3(3-thienyl)OBnCF3_____3.lcd		
Method Filename	: andrey.lcm		
Batch Filename	:		
Vial #	: 1-86	Sample Type	: Unknown
Injection Volume	: 2 uL		
Date Acquired	: 13/05/2018 18:05:34	Acquired by	: System Administrator
Date Processed	: 13/05/2018 18:59:43	Processed by	: System Administrator

<Chromatogram>

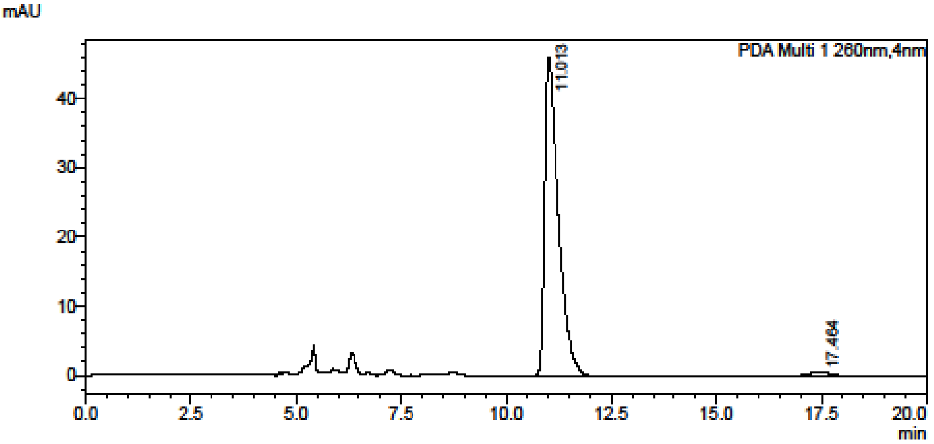

<Peak Table>

PDA Ch1 260nm

Peak#	Ret. Time	Area	Area%	Height
1	11.013	1043782	98.233	45975
2	17.464	18778	1.767	556
Total		1062561	100.000	46531

C:\Andrey\CF3(3-thienyl)OBnCF3_____3.lcd

Supplementary figure 273. HPLC chromatogram for compound **6o**, Ligand: Ligand:**(4R,5S)-7a**

SHIMADZU LabSolutions

Analysis Report

<Sample Information>

Sample Name : CF3(3-thienyl)OBnCF3
Sample ID : CF3(3-thienyl)OBnCF3
Data Filename : CF3(3-thienyl)OBnCF3_____1.lcd
Method Filename : andrey.lcm
Batch Filename :
Vial # : 1-64 Sample Type : Unknown
Injection Volume : 2 uL
Date Acquired : 13/05/2018 17:03:47 Acquired by : System Administrator
Date Processed : 13/05/2018 18:57:19 Processed by : System Administrator

<Chromatogram>

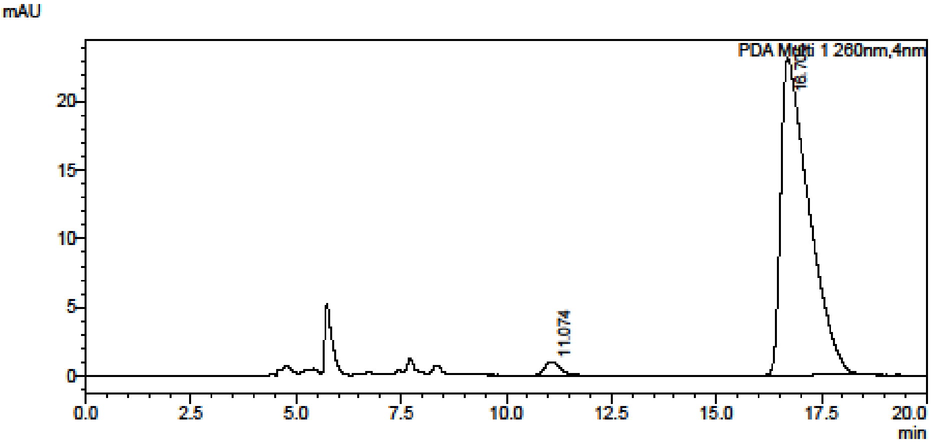

<Peak Table>

PDA Ch1 260nm

Peak#	Ret. Time	Area	Area%	Height
1	11.074	23280	2.121	999
2	16.705	1074550	97.879	23129
Total		1097830	100.000	24128

C:\Andrey\CF3(3-thienyl)OBnCF3_____1.lcd

Supplementary figure 274. HPLC chromatogram for compound **6o**, Ligand: Ligand:**(4S,5R)-7a**

13/05/2018 19:01:08 Page 1 / 1

SHIMADZU LabSolutions

Analysis Report

<Sample Information>

Sample Name	: CF3(3-thienyl)OBnCF3		
Sample ID	: CF3(3-thienyl)OBnCF3		
Data Filename	: CF3(3-thienyl)OBnCF3_____4.lcd		
Method Filename	: andrey.lcm		
Batch Filename	:		
Vial #	: 1-81	Sample Type	: Unknown
Injection Volume	: 2 uL		
Date Acquired	: 13/05/2018 18:33:19	Acquired by	: System Administrator
Date Processed	: 13/05/2018 19:00:49	Processed by	: System Administrator

<Chromatogram>

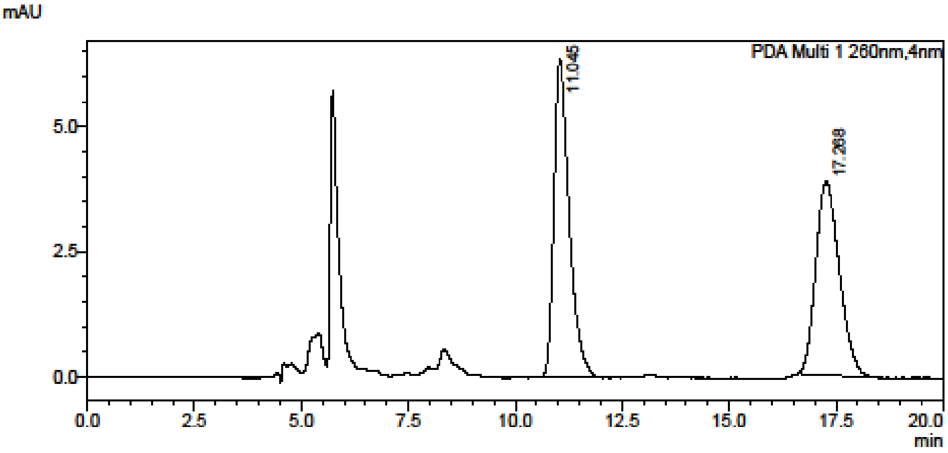

<Peak Table>

PDA Ch1 260nm

Peak#	Ret. Time	Area	Area%	Height
1	11.045	158580	51.603	6347
2	17.268	148728	48.397	3861
Total		307308	100.000	10208

C:\Andrey\CF3(3-thienyl)OBnCF3_____4.lcd

Supplementary figure 275. HPLC chromatogram for compound **6o**, racemic

Analysis Report

<Sample Information>

Sample Name	: CF3(5-(2-MeOPy))OBnCF3		
Sample ID	: CF3(5-(2-MeOPy))OBnCF3		
Data Filename	: CF3(5-(2-MeOPy))OBnCF3__2.lcd		
Method Filename	: andrey.lcm		
Batch Filename	:		
Vial #	: 1-77	Sample Type	: Unknown
Injection Volume	: 2 uL		
Date Acquired	: 06/05/2018 12:14:30	Acquired by	: System Administrator
Date Processed	: 07/05/2018 16:32:15	Processed by	: System Administrator

<Chromatogram>

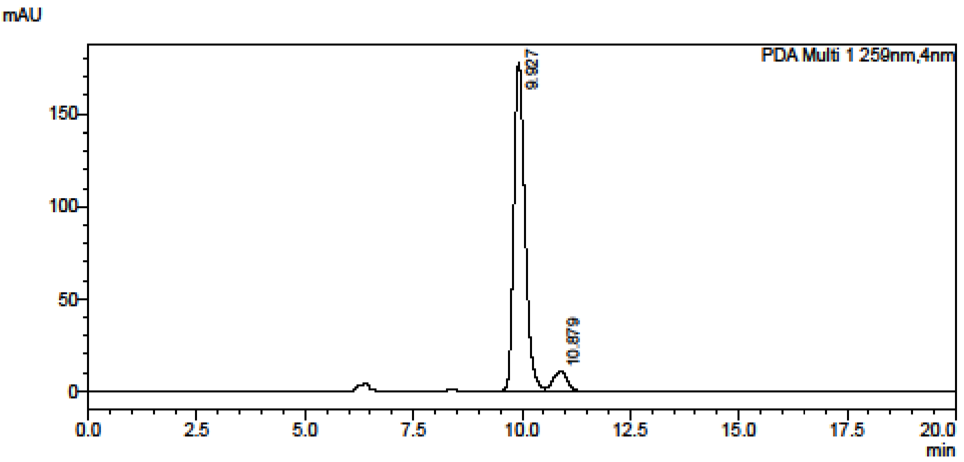

<Peak Table>

PDA Ch1 259nm

Peak#	Ret. Time	Area	Area%	Height
1	9.927	3191573	93.096	177314
2	10.879	236675	6.904	10475
Total		3428249	100.000	187790

Supplementary figure 276. HPLC chromatogram for compound **6p**, Ligand: Ligand:**(4R,5S)-7a**

Analysis Report

<Sample Information>

Sample Name : CF3(5-(2-MeOPy))OBnCF3
Sample ID : CF3(5-(2-MeOPy))OBnCF3
Data Filename : CF3(5-(2-MeOPy))OBnCF3__1.lcd
Method Filename : andrey.lcm
Batch Filename :
Vial # : 1-76 Sample Type : Unknown
Injection Volume : 2 uL
Date Acquired : 06/05/2018 11:50:23 Acquired by : System Administrator
Date Processed : 07/05/2018 16:28:45 Processed by : System Administrator

<Chromatogram>

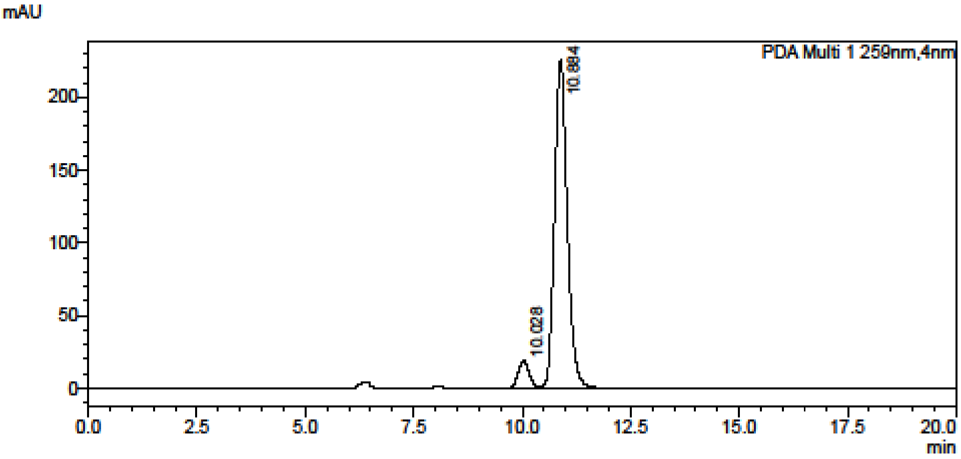

<Peak Table>

PDA Ch1 259nm

Peak#	Ret. Time	Area	Area%	Height
1	10.028	327869	6.730	18665
2	10.884	4543673	93.270	225184
Total		4871542	100.000	243849

PDA Ch2 259nm

Peak#	Ret. Time	Area	Area%	Height
1	10.028	328235	6.732	18672
2	10.884	4547551	93.268	225209
Total		4875786	100.000	243881

Supplementary figure 277. HPLC chromatogram for compound **6p**, Ligand: Ligand:**(4S,5R)-7a**

Analysis Report

<Sample Information>

Sample Name	: CF3(5-(2-MeOPy))OBnCF3		
Sample ID	: CF3(5-(2-MeOPy))OBnCF3		
Data Filename	: CF3(5-(2-MeOPy))OBnCF3__3.lcd		
Method Filename	: andrey.lcm		
Batch Filename	:		
Vial #	: 1-78	Sample Type	: Unknown
Injection Volume	: 2 uL		
Date Acquired	: 06/05/2018 12:35:34	Acquired by	: System Administrator
Date Processed	: 07/05/2018 16:33:19	Processed by	: System Administrator

<Chromatogram>

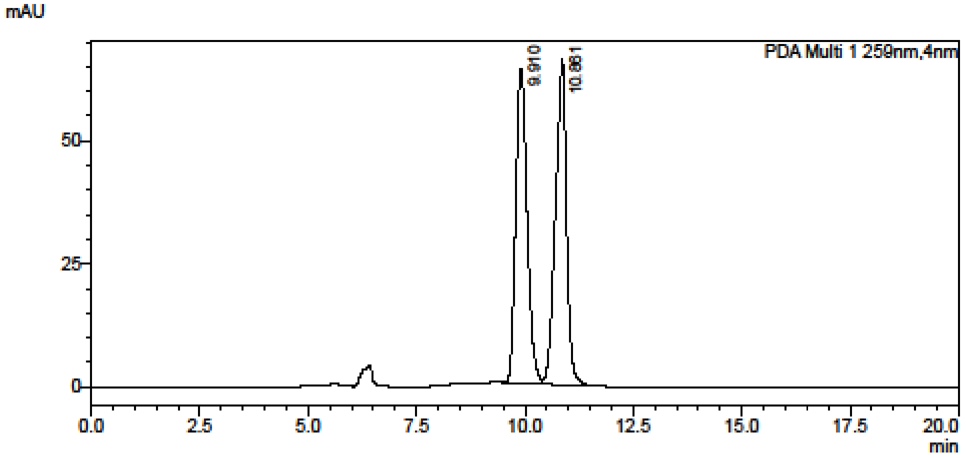

<Peak Table>

PDA Ch1 259nm

Peak#	Ret. Time	Area	Area%	Height
1	9.910	1165498	50.022	63723
2	10.861	1164474	49.978	66011
Total		2329972	100.000	129733

C:\Andrey\CF3(5-(2-MeOPy))OBnCF3__3.lcd

Supplementary figure 278. HPLC chromatogram for compound **6p**, racemic

Analysis Report

<Sample Information>

Sample Name	: CF3(3-quinolinyl)OBnCF3		
Sample ID	: CF3(3-quinolinyl)OBnCF3		
Data Filename	: CF3(3-quinolinyl)OBnCF3_1.lcd		
Method Filename	: andrey.lcm		
Batch Filename	:		
Vial #	: 1-75	Sample Type	: Unknown
Injection Volume	: 3 uL		
Date Acquired	: 04/05/2018 17:42:18	Acquired by	: System Administrator
Date Processed	: 07/05/2018 16:45:00	Processed by	: System Administrator

<Chromatogram>

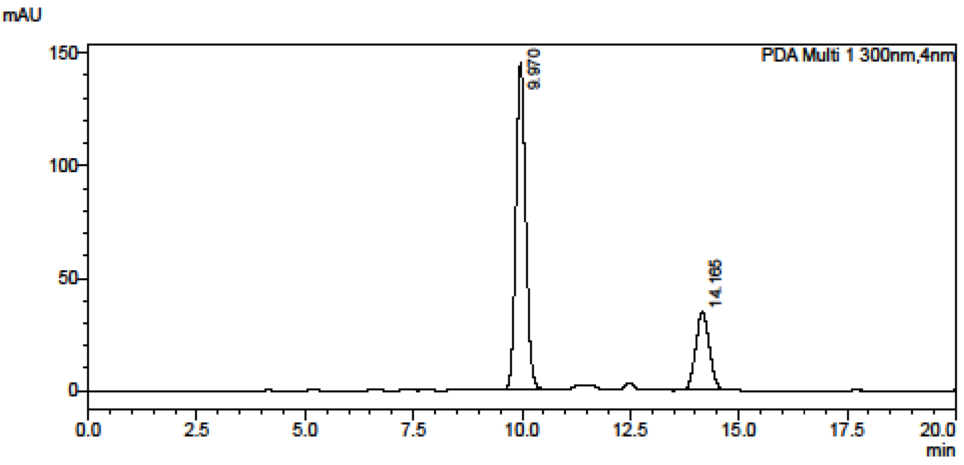

<Peak Table>

PDA Ch1 300nm

Peak#	Ret. Time	Area	Area%	Height
1	9.970	2283509	74.680	145216
2	14.165	774220	25.320	34848
Total		3057729	100.000	180064

Supplementary figure 279. HPLC chromatogram for compound **6q**, Ligand: Ligand:**(4R,5S)-7a**

SHIMADZU LabSolutions **Analysis Report**

<Sample Information>

Sample Name	: CF3(3-quinolinyl)OBnCF3		
Sample ID	: CF3(3-quinolinyl)OBnCF3		
Data Filename	: CF3(3-quinolinyl)OBnCF3_4.lcd		
Method Filename	: andrey.lcm		
Batch Filename	:		
Vial #	: 1-86	Sample Type	: Unknown
Injection Volume	: 2 uL		
Date Acquired	: 08/05/2018 13:41:49	Acquired by	: System Administrator
Date Processed	: 08/05/2018 14:22:19	Processed by	: System Administrator

<Chromatogram>

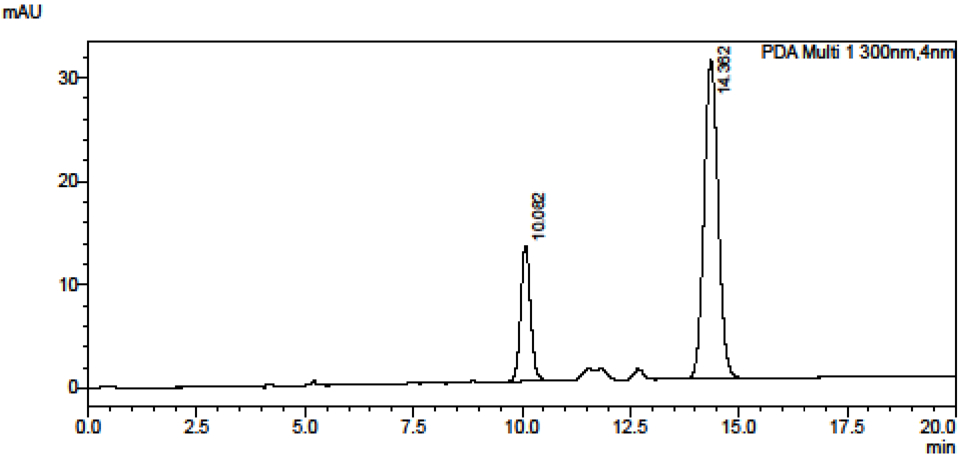

<Peak Table>

PDA Ch1 300nm

Peak#	Ret. Time	Area	Area%	Height
1	10.082	207263	22.989	13173
2	14.362	694312	77.011	30797
Total		901574	100.000	43970

C:\Andrey\CF3(3-quinolinyl)OBnCF3_4.lcd

Supplementary figure 280. HPLC chromatogram for compound **6q**, Ligand: Ligand:**(4S,5R)-7a**

SHIMADZU LabSolutions **Analysis Report**

<Sample Information>

Sample Name	: CF3(3-Quinolinyl)OBnCF3		
Sample ID	: CF3(3-Quinolinyl)OBnCF3		
Data Filename	: CF3(3-Quinolinyl)OBnCF3_rac4.lcd		
Method Filename	: andrey.lcm		
Batch Filename	:		
Vial #	: 1-105	Sample Type	: Unknown
Injection Volume	: 1 uL		
Date Acquired	: 16/05/2018 12:27:28	Acquired by	: System Administrator
Date Processed	: 18/05/2018 16:21:45	Processed by	: System Administrator

<Chromatogram>

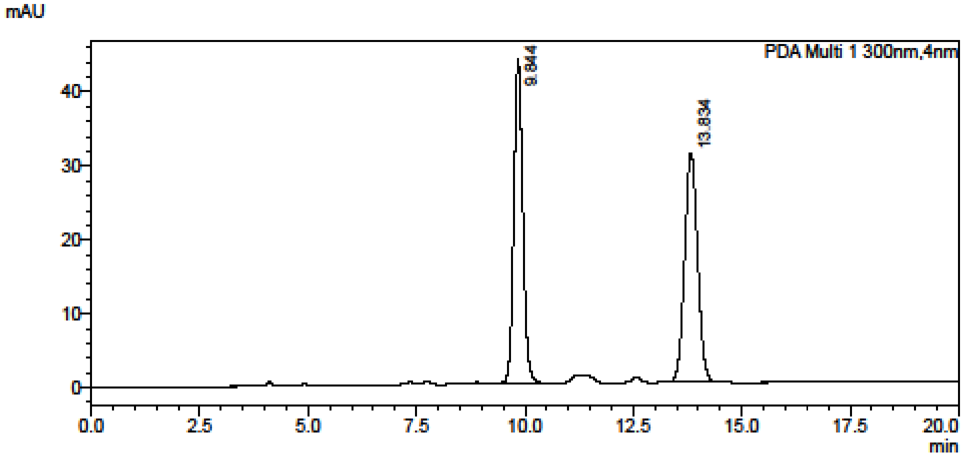

<Peak Table>

PDA Ch1 300nm

Peak#	Ret. Time	Area	Area%	Height
1	9.844	632363	49.450	43812
2	13.834	646422	50.550	31099
Total		1278784	100.000	74911

C:\Andrey\CF3(3-Quinolinyl)OBnCF3_rac4.lcd

Supplementary figure 281. HPLC chromatogram for compound **6n**, racemic

SHIMADZU LabSolutions

Analysis Report

<Sample Information>

Sample Name	: C4F9(p-MeOPh)-OBnCF3		
Sample ID	: C4F9(p-MeOPh)-OBnCF3		
Data Filename	: C4F9(p-MeOPh)-OBnCF3_7.lcd		
Method Filename	: andrey.lcm		
Batch Filename	:		
Vial #	: 1-77	Sample Type	: Unknown
Injection Volume	: 2 uL		
Date Acquired	: 11/07/2017 17:40:58	Acquired by	: System Administrator
Date Processed	: 22/08/2017 19:28:35	Processed by	: System Administrator

<Chromatogram>

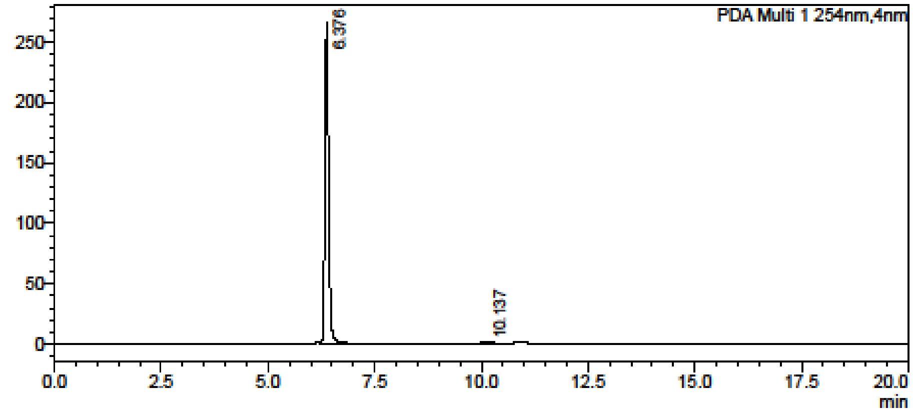

<Peak Table>

PDA Ch1 254nm

Peak#	Ret. Time	Area	Area%	Height
1	6.376	1602066	98.113	265753
2	10.137	30813	1.887	1277
Total		1632879	100.000	267031

C:\Andrey\C4F9(p-MeOPh)-OBnCF3_7.lcd

Supplementary figure 282. HPLC chromatogram for compound **6b’,** Ligand:**(4R,5S)-7a**

SHIMADZU LabSolutions

Analysis Report

<Sample Information>

Sample Name	: C4F9(p-MeOPh)OBnCF3		
Sample ID	: C4F9(p-MeOPh)OBnCF3		
Data Filename	: C4F9(p-MeOPh)OBnCF3_1.lcd		
Method Filename	: andrey.lcm		
Batch Filename	:		
Vial #	: 1-93	Sample Type	: Unknown
Injection Volume	: 1 uL		
Date Acquired	: 16/08/2017 13:22:41	Acquired by	: System Administrator
Date Processed	: 22/08/2017 19:25:05	Processed by	: System Administrator

<Chromatogram>

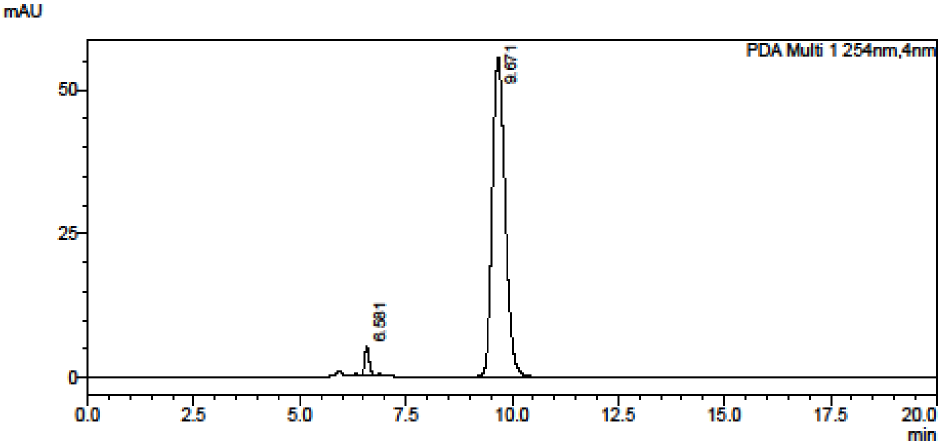

<Peak Table>

PDA Ch1 254nm

Peak#	Ret. Time	Area	Area%	Height
1	6.581	38738	3.206	5155
2	9.671	1169397	96.794	55444
Total		1208135	100.000	60599

C:\Andrey\C4F9(p-MeOPh)OBnCF3_1.lcd

Supplementary figure 283. HPLC chromatogram for compound **6b’,** Ligand:**(4S,5R)-7a**

Analysis Report

SHIMADZU LabSolutions

<Sample Information>

Sample Name	: C4F9(p-MeOPh)OBnCF3 rac		
Sample ID	: C4F9(p-MeOPh)OBnCF3 rac		
Data Filename	: C4F9(p-MeOPh)OBnCF3_rac2.lcd		
Method Filename	: andrey.lcm		
Batch Filename	:		
Vial #	: 1-92	Sample Type	: Unknown
Injection Volume	: 1 uL		
Date Acquired	: 15/08/2017 16:16:31	Acquired by	: System Administrator
Date Processed	: 15/08/2017 17:20:42	Processed by	: System Administrator

<Chromatogram>

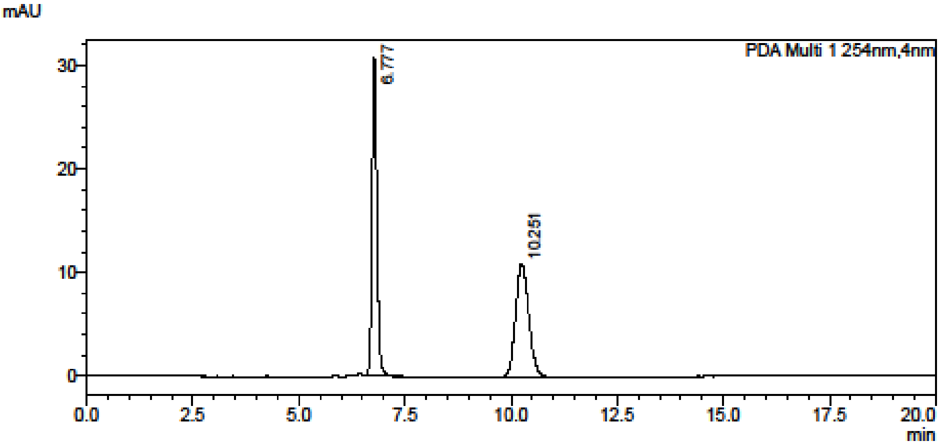

<Peak Table>

PDA Ch1 254nm

Peak#	Ret. Time	Area	Area%	Height
1	6.777	239138	50.028	30712
2	10.251	238875	49.972	10994
Total		478012	100.000	41706

C:\Andrey\C4F9(p-MeOPh)OBnCF3_rac2.lcd

Supplementary figure 284. HPLC chromatogram for compound **6b’,** racemic

SHIMADZU LabSolutions

Analysis Report

<Sample Information>

Sample Name	: C8F17(p-MeOPh)OBnCF3_old		
Sample ID	: C8F17(p-MeOPh)OBnCF3_old		
Data Filename	: C8F17(p-MeOPh)OBnCF3_old15.lcd		
Method Filename	: andrey.lcm		
Batch Filename	:		
Vial #	: 1-74	Sample Type	: Unknown
Injection Volume	: 1 uL		
Date Acquired	: 28/08/2017 18:47:12	Acquired by	: System Administrator
Date Processed	: 28/08/2017 19:21:16	Processed by	: System Administrator

<Chromatogram>

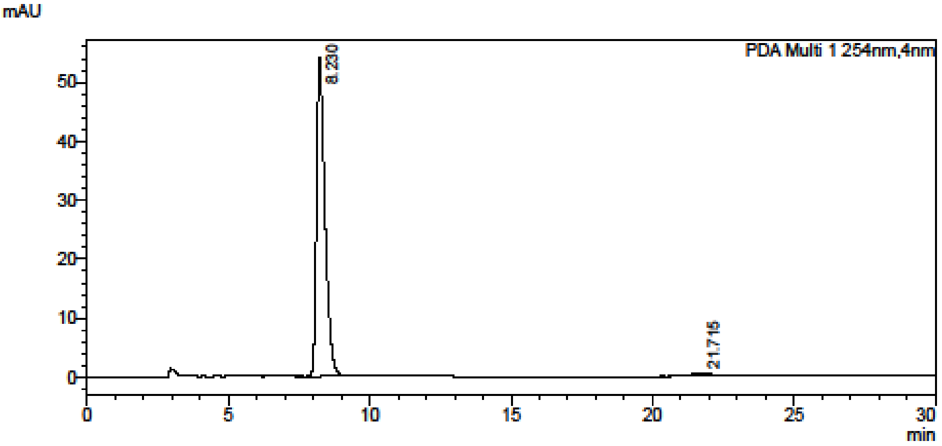

<Peak Table>

PDA Ch1 254nm

Peak#	Ret. Time	Area	Area%	Height
1	8.230	1142925	97.626	53869
2	21.715	27789	2.374	595
Total		1170714	100.000	54464

Supplementary figure 285. HPLC chromatogram for compound **6b''**, Ligand:**(4R,5S)-7a**

Analysis Report

<Sample Information>

Sample Name	: C8F17(p-MeOPh)OBnCF3_new		
Sample ID	: C8F17(p-MeOPh)OBnCF3_rac		
Data Filename	: C8F17(p-MeOPh)OBnCF3_new14.lcd		
Method Filename	: andrey.lcm		
Batch Filename	:		
Vial #	: 1-46	Sample Type	: Unknown
Injection Volume	: 2 uL		
Date Acquired	: 28/08/2017 18:12:33	Acquired by	: System Administrator
Date Processed	: 28/08/2017 19:03:30	Processed by	: System Administrator

<Chromatogram>

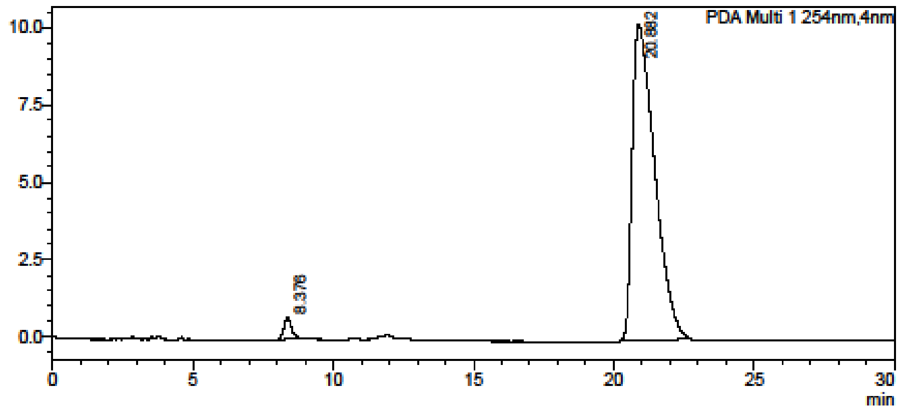

<Peak Table>

PDA Ch1 254nm

Peak#	Ret. Time	Area	Area%	Height
1	8.376	13354	2.296	689
2	20.882	568398	97.704	10188
Total		581752	100.000	10877

C:\Andrey\C8F17(p-MeOPh)OBnCF3_new14.lcd

Supplementary figure 286. HPLC chromatogram for compound **6b'',** Ligand:**(4S,5R)-7a**

Analysis Report

<Sample Information>

Sample Name : C8F17(p-MeOPh)OBnCF3_rac
Sample ID : C8F17(p-MeOPh)OBnCF3_rac
Data Filename : C8F17(p-MeOPh)OBnCF3_rac13.lcd
Method Filename : andrey.lcm
Batch Filename :
Vial # : 1-75
Sample Type : Unknown
Injection Volume : 1 uL
Date Acquired : 28/08/2017 17:41:41
Acquired by : System Administrator
Date Processed : 28/08/2017 19:01:38
Processed by : System Administrator

<Chromatogram>

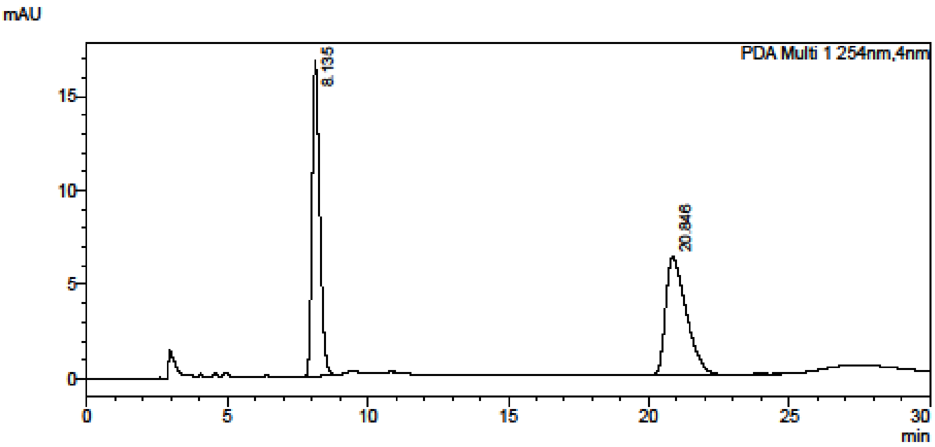

<Peak Table>

PDA Ch1 254nm

Peak#	Ret. Time	Area	Area%	Height
1	8.135	322601	50.080	16755
2	20.846	321568	49.920	6297
Total		644169	100.000	23052

C:\Andrey\C8F17(p-MeOPh)OBnCF3_rac13.lcd

Supplementary figure 287. HPLC chromatogram for compound **6b'',** racemic

09/07/2017 10:07:15 Page 1 / 1

SHIMADZU LabSolutions

Analysis Report

<Sample Information>

Sample Name	: CF3(vinyl)-OBn_new		
Sample ID	: CF3(vinyl)-OBn_new		
Data Filename	: CF3(vinyl)-OBn_new1.lcd		
Method Filename	: shutdown.lcm		
Batch Filename	:		
Vial #	: 1-100	Sample Type	: Unknown
Injection Volume	: 3 uL		
Date Acquired	: 02/07/2017 12:05:18	Acquired by	: System Administrator
Date Processed	: 09/07/2017 10:06:44	Processed by	: System Administrator

<Chromatogram>

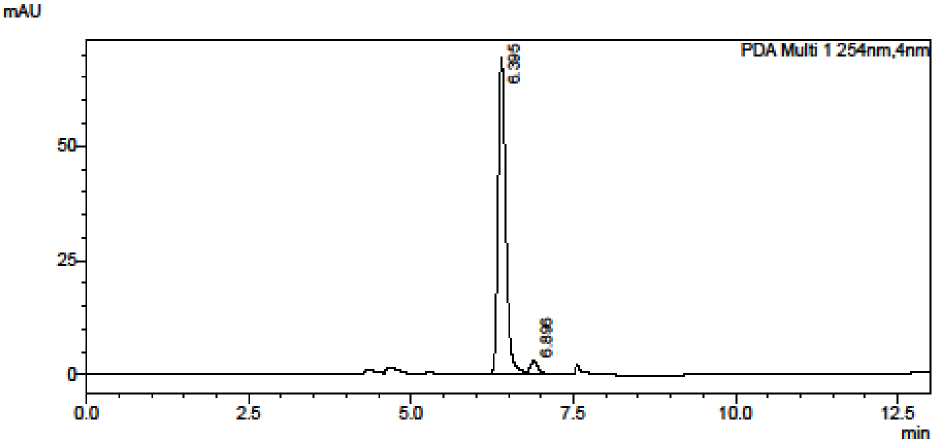

<Peak Table>

PDA Ch1 254nm

Peak#	Ret. Time	Area	Area%	Height
1	6.395	567555	95.308	69188
2	6.896	27943	4.692	2934
Total		595498	100.000	72122

C:\Andrey\CF3(vinyl)-OBn_new1.lcd

Supplementary figure 288. HPLC chromatogram for compound **8a,** Ligand:**(4R)-7g**

09/07/2017 10:07:15 Page 1 / 1

SHIMADZU LabSolutions **Analysis Report**

<Sample Information>

Sample Name	: CF3(vinyl)-OBn_new		
Sample ID	: CF3(vinyl)-OBn_new		
Data Filename	: CF3(vinyl)-OBn_new1.lcd		
Method Filename	: shutdown.lcm		
Batch Filename	:		
Vial #	: 1-100	Sample Type	: Unknown
Injection Volume	: 3 uL		
Date Acquired	: 02/07/2017 12:05:18	Acquired by	: System Administrator
Date Processed	: 09/07/2017 10:06:44	Processed by	: System Administrator

<Chromatogram>

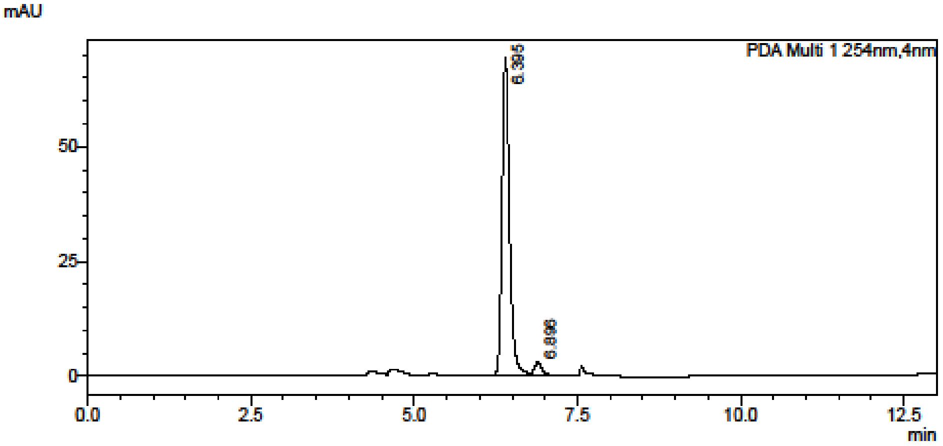

<Peak Table>

PDA Ch1 254nm

Peak#	Ret. Time	Area	Area%	Height
1	6.395	567555	95.308	69188
2	6.896	27943	4.692	2934
Total		595498	100.000	72122

C:\Andrey\CF3(vinyl)-OBn_new1.lcd

Supplementary figure 289. HPLC chromatogram for compound **8a,** Ligand:**(4S)-7g**

SHIMADZU LabSolutions **Analysis Report**

<Sample Information>

Sample Name	: CF3(vin)-OBnCF3		
Sample ID	: CF3(vin)-OBnCF3		
Data Filename	: CF3(vin)-OBnCF4.lcd		
Method Filename	: shutdown.lcm		
Batch Filename	:		
Vial #	: 1-63	Sample Type	: Unknown
Injection Volume	: 3 uL		
Date Acquired	: 06/07/2017 16:03:00	Acquired by	: System Administrator
Date Processed	: 09/07/2017 10:04:24	Processed by	: System Administrator

<Chromatogram>

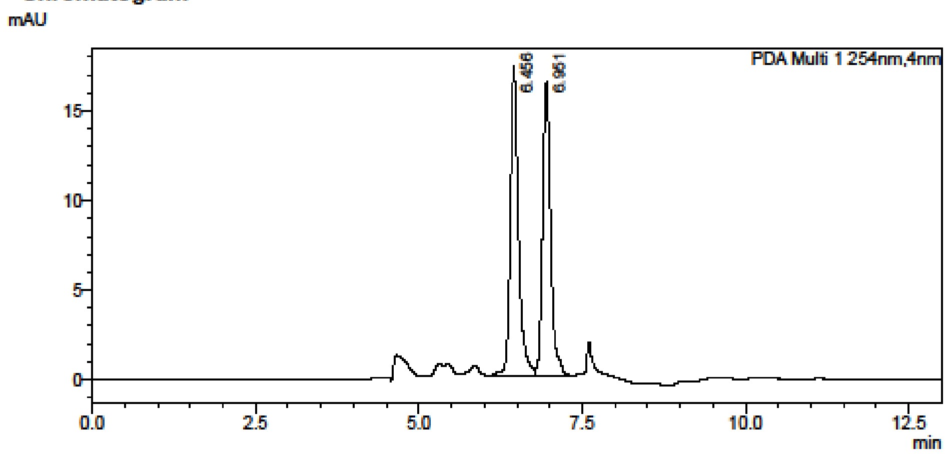

<Peak Table>

PDA Ch1 254nm

Peak#	Ret. Time	Area	Area%	Height
1	6.456	153978	52.697	17254
2	6.951	138216	47.303	16430
Total		292194	100.000	33685

C:\Andrey\CF3(vin)-OBnCF4.lcd

Supplementary figure 290. HPLC chromatogram for compound **8a,** racemic

Analysis Report

<Sample Information>

Sample Name	: CF3(octenyl)-OBn_new		
Sample ID	: CF3(octenyl)-OBn_new		
Data Filename	: CF3(octenyl)-OBn_new1.lcd		
Method Filename	: shutdown.lcm		
Batch Filename	:		
Vial #	: 1-101	Sample Type	: Unknown
Injection Volume	: 3 uL		
Date Acquired	: 02/07/2017 13:05:34	Acquired by	: System Administrator
Date Processed	: 09/07/2017 10:11:46	Processed by	: System Administrator

<Chromatogram>

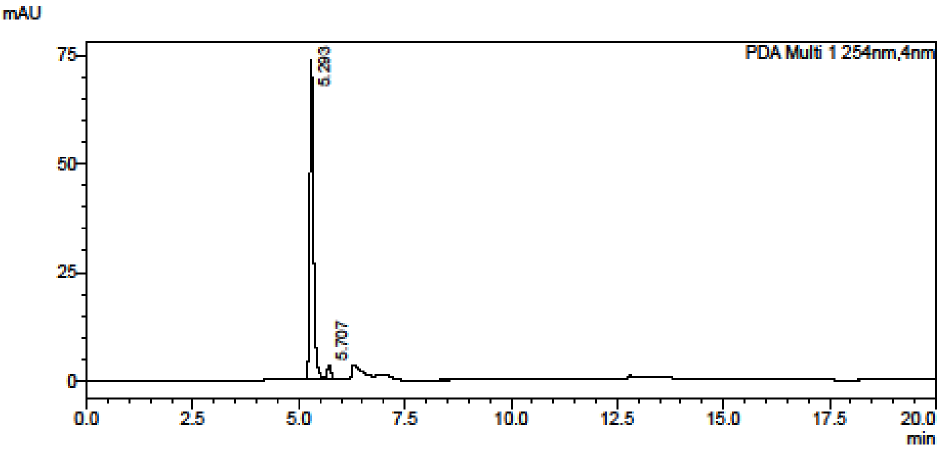

<Peak Table>

PDA Ch1 254nm

Peak#	Ret. Time	Area	Area%	Height
1	5.293	445048	95.298	73443
2	5.707	21960	4.702	3468
Total		467007	100.000	76911

Supplementary figure 291. HPLC chromatogram for compound **8b,** Ligand:**(4R)-7g**

Analysis Report

<Sample Information>

Sample Name	: CF3(octenyl)-OBn		
Sample ID	: CF3(octenyl)-OBn		
Data Filename	: CF3(octenyl)-OBn3.lcd		
Method Filename	: shutdown.lcm		
Batch Filename	:		
Vial #	: 1-89	Sample Type	: Unknown
Injection Volume	: 1 uL		
Date Acquired	: 01/07/2017 17:42:25	Acquired by	: System Administrator
Date Processed	: 09/07/2017 10:13:55	Processed by	: System Administrator

<Chromatogram>

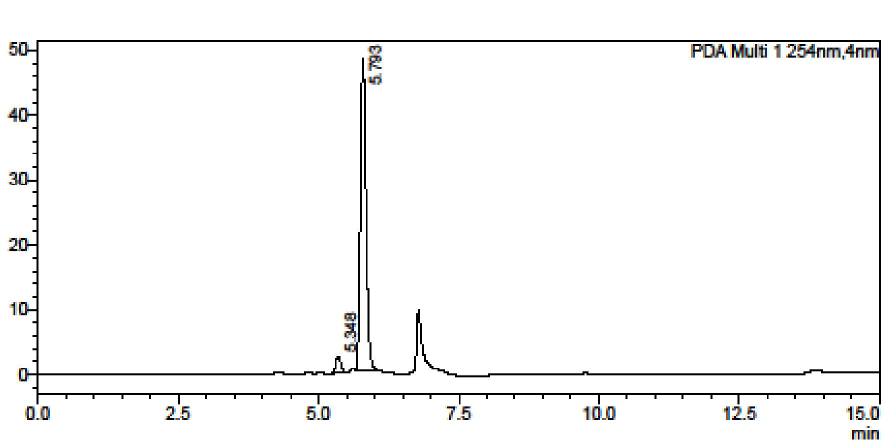

<Peak Table>

PDA Ch1 254nm

Peak#	Ret. Time	Area	Area%	Height
1	5.348	16262	4.669	2636
2	5.793	332014	95.331	47997
Total		348276	100.000	50633

C:\Andrey\CF3(octenyl)-OBn3.lcd

Supplementary figure 292. HPLC chromatogram for compound **8b,** Ligand:**(4S)-7g**

SHIMADZU LabSolutions Analysis Report

<Sample Information>

Sample Name	: CF3(octenyl)OBn rac		
Sample ID	: CF3(octenyl)OBn rac		
Data Filename	: CF3(octenyl)OBn1rac1.lcd		
Method Filename	: andrey.lcm		
Batch Filename	:		
Vial #	: 1-99	Sample Type	: Unknown
Injection Volume	: 2 uL		
Date Acquired	: 10/08/2017 15:11:10	Acquired by	: System Administrator
Date Processed	: 10/08/2017 18:04:13	Processed by	: System Administrator

<Chromatogram>

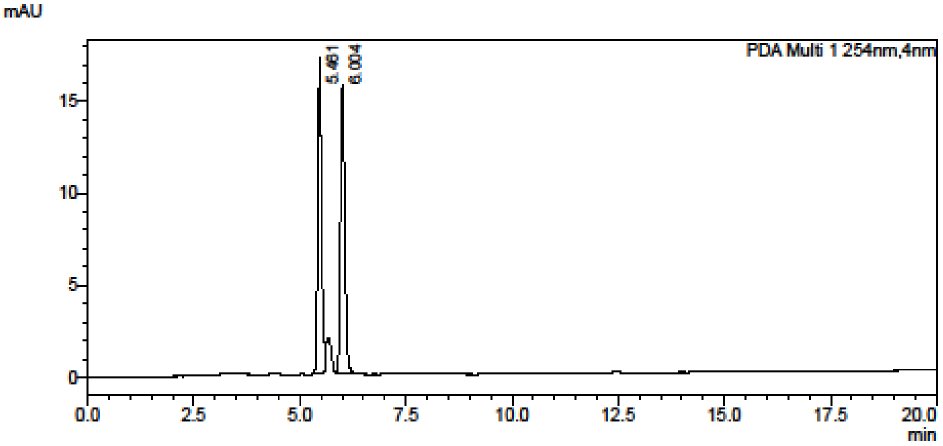

<Peak Table>

PDA Ch1 254nm

Peak#	Ret. Time	Area	Area%	Height
1	5.461	109782	49.309	17117
2	6.004	112859	50.691	15661
Total		222641	100.000	32778

C:\Andrey\CF3(octenyl)OBn1rac1.lcd

Supplementary figure 293. HPLC chromatogram for compound **8b,** racemic

09/07/2017 10:13:12 Page 1 / 1

SHIMADZU LabSolutions **Analysis Report**

<Sample Information>

Sample Name	: CF3(stryrenyl)-OBn_new		
Sample ID	: CF3(stryrenyl)-OBn_new		
Data Filename	: CF3(stryrenyl)-OBn_new1.lcd		
Method Filename	: shutdown.lcm		
Batch Filename	:		
Vial #	: 1-90	Sample Type	: Unknown
Injection Volume	: 3 uL		
Date Acquired	: 02/07/2017 11:41:30	Acquired by	: System Administrator
Date Processed	: 09/07/2017 10:12:56	Processed by	: System Administrator

<Chromatogram>

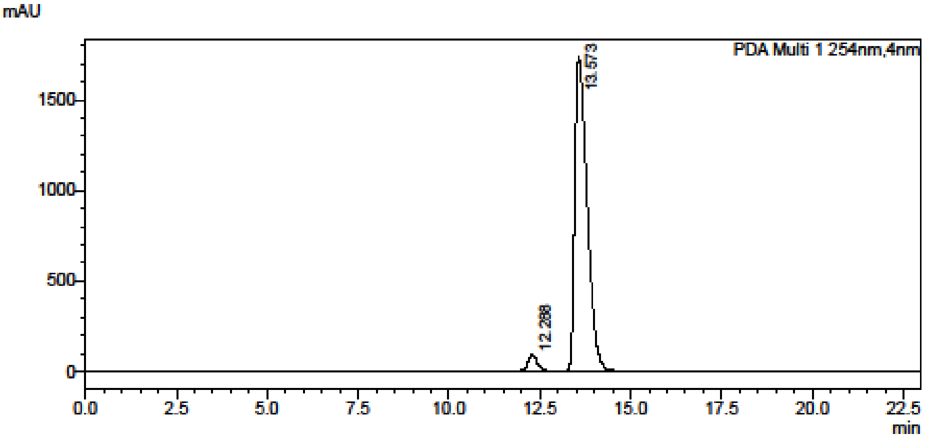

<Peak Table>

PDA Ch1 254nm

Peak#	Ret. Time	Area	Area%	Height
1	12.288	1705070	3.982	91906
2	13.573	41113424	96.018	1735969
Total		42818495	100.000	1827875

C:\Andrey\CF3(stryrenyl)-OBn_new1.lcd

Supplementary figure 294. HPLC chromatogram for compound **8c,** Ligand:**(4R)-7g**

30/06/2017 17:23:57 Page 1 / 1

SHIMADZU LabSolutions **Analysis Report**

<Sample Information>

Sample Name	: CF3(styrenyl)-OBn		
Sample ID	: CF3(styrenyl)-OBn		
Data Filename	: CF3(styrenyl)-OBn3.lcd		
Method Filename	: shutdown.lcm		
Batch Filename	:		
Vial #	: 1-86	Sample Type	: Unknown
Injection Volume	: 4 uL		
Date Acquired	: 29/06/2017 16:10:43	Acquired by	: System Administrator
Date Processed	: 30/06/2017 17:22:37	Processed by	: System Administrator

<Chromatogram>

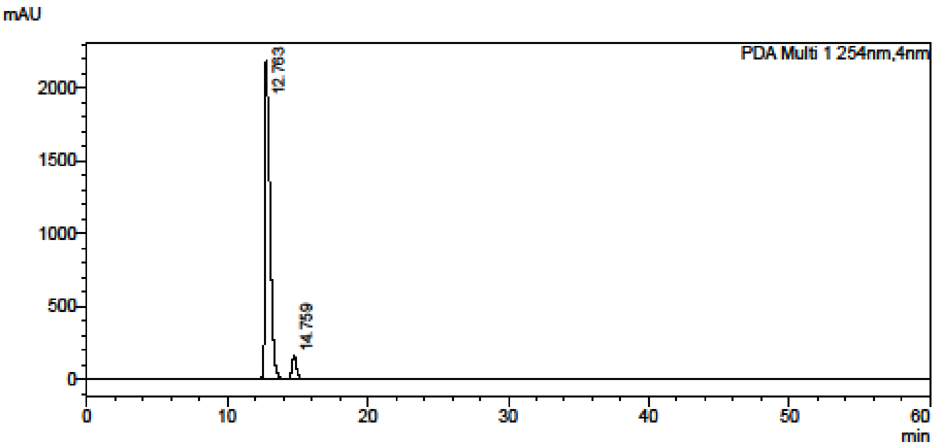

<Peak Table>

PDA Ch1 254nm

Peak#	Ret. Time	Area	Area%	Height
1	12.763	58235004	94.207	2187325
2	14.759	3580838	5.793	158236
Total		61815842	100.000	2345561

C:\Andrey\CF3(styrenyl)-OBn3.lcd

Supplementary figure 295. HPLC chromatogram for compound **8a,** Ligand:**(4S)-7g**

Analysis Report

<Sample Information>

Sample Name	: CF3(styrenyl)OBn_rac		
Sample ID	: CF3(styrenyl)OBn_rac		
Data Filename	: CF3(styrenyl)OBn_-rac1.lcd		
Method Filename	: andrey.lcm		
Batch Filename	:		
Vial #	: 1-98	Sample Type	: Unknown
Injection Volume	: 2 uL		
Date Acquired	: 10/08/2017 16:28:26	Acquired by	: System Administrator
Date Processed	: 10/08/2017 17:44:09	Processed by	: System Administrator

<Chromatogram>

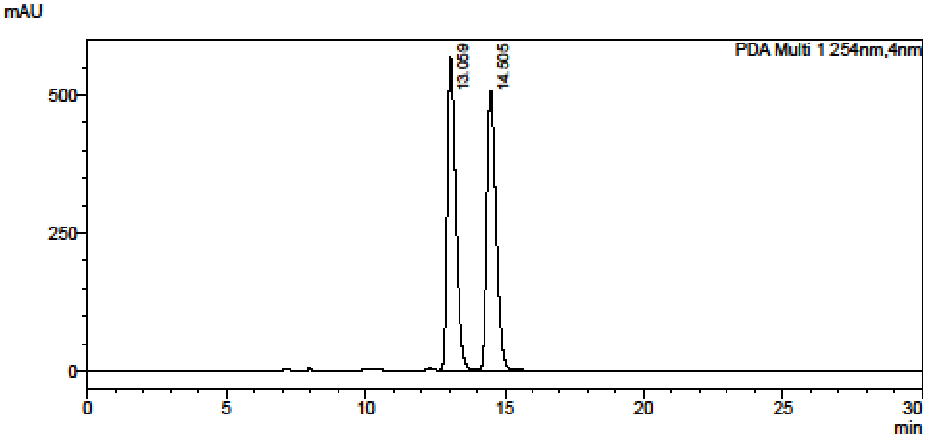

<Peak Table>

PDA Ch1 254nm

Peak#	Ret. Time	Area	Area%	Height
1	13.059	11747613	50.428	567217
2	14.505	11548344	49.572	507420
Total		23295957	100.000	1074637

C:\Andrey\CF3(styrenyl)OBn_-rac1.lcd

Supplementary figure 296. HPLC chromatogram for compound **8c,** racemic

Analysis Report

<Sample Information>

Sample Name	: CF3(styrenyl)OBnCF3_new		
Sample ID	: CF3(styrenyl)OBnCF3_new		
Data Filename	: CF3(styrenyl)OBnCF3_new7.lcd		
Method Filename	: andrey.lcm		
Batch Filename	:		
Vial #	: 1-82	Sample Type	: Unknown
Injection Volume	: 1 uL		
Date Acquired	: 21/08/2017 18:35:06	Acquired by	: System Administrator
Date Processed	: 21/08/2017 19:22:00	Processed by	: System Administrator

<Chromatogram>

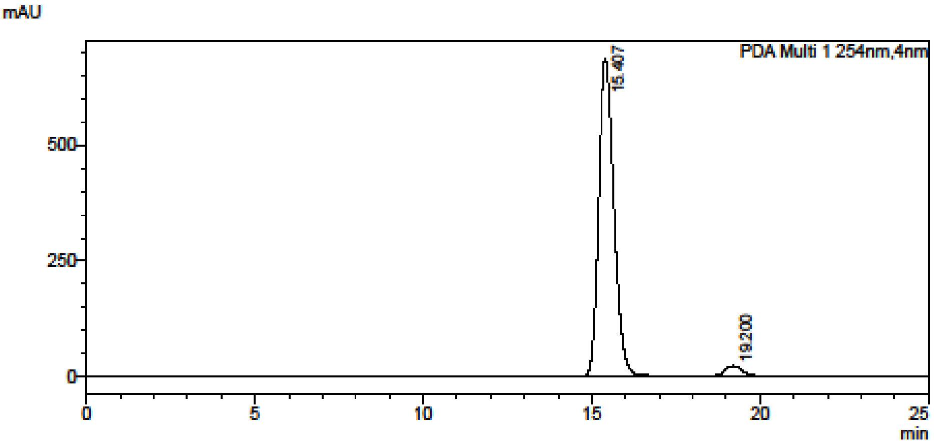

<Peak Table>

PDA Ch1 254nm

Peak#	Ret. Time	Area	Area%	Height
1	15.407	21777529	96.320	686498
2	19.200	832146	3.680	22863
Total		22609675	100.000	709360

C:\Andrey\CF3(styrenyl)OBnCF3_new7.lcd

Supplementary figure 297. HPLC chromatogram for compound **8f,** Ligand:**(4S)-7g**

SHIMADZU LabSolutions **Analysis Report**

<Sample Information>

Sample Name	: CF3(styrenyl)OBnCF3_old
Sample ID	: CF3(styrenyl)OBnCF3_old
Data Filename	: CF3(styrenyl)OBnCF3_old6.lcd
Method Filename	: andrey.lcm
Batch Filename	:
Vial #	: 1-36
Injection Volume	: 1 uL
Date Acquired	: 21/08/2017 18:08:31
Date Processed	: 21/08/2017 19:21:28
Sample Type	: Unknown
Acquired by	: System Administrator
Processed by	: System Administrator

<Chromatogram>

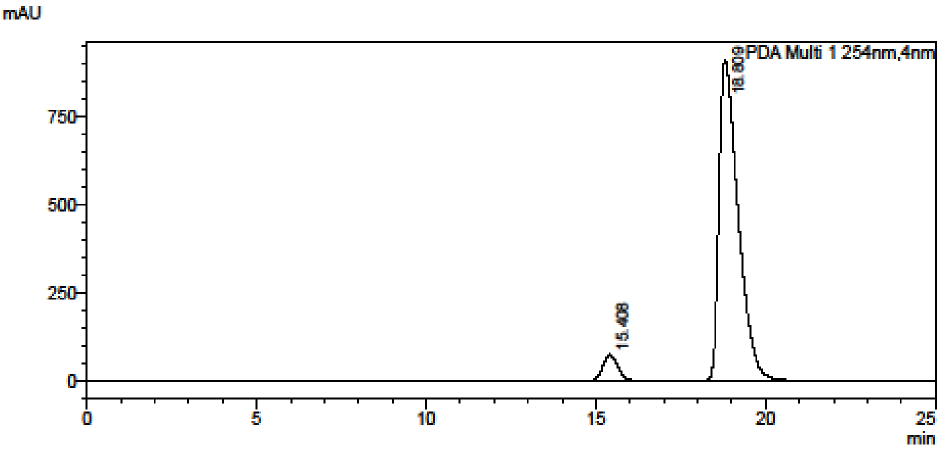

<Peak Table>

PDA Ch1 254nm

Peak#	Ret. Time	Area	Area%	Height
1	15.408	2348505	6.153	75527
2	18.809	35817287	93.847	910271
Total		38165792	100.000	985798

C:\Andrey\CF3(styrenyl)OBnCF3_old6.lcd

Supplementary figure 298. HPLC chromatogram for compound **8f,** Ligand:**(4R)-7g**

21/08/2017 19:28:20 Page 1 / 1

SHIMADZU LabSolutions

Analysis Report

<Sample Information>

Sample Name	: CF3(styrenyl)OBnCF3_rac		
Sample ID	: CF3(styrenyl)OBnCF3_rac		
Data Filename	: CF3(styrenyl)OBnCF3_rac1.lcd		
Method Filename	: andrey.lcm		
Batch Filename	:		
Vial #	: 1-14	Sample Type	: Unknown
Injection Volume	: 1 uL		
Date Acquired	: 21/08/2017 19:02:43	Acquired by	: System Administrator
Date Processed	: 21/08/2017 19:28:06	Processed by	: System Administrator

<Chromatogram>

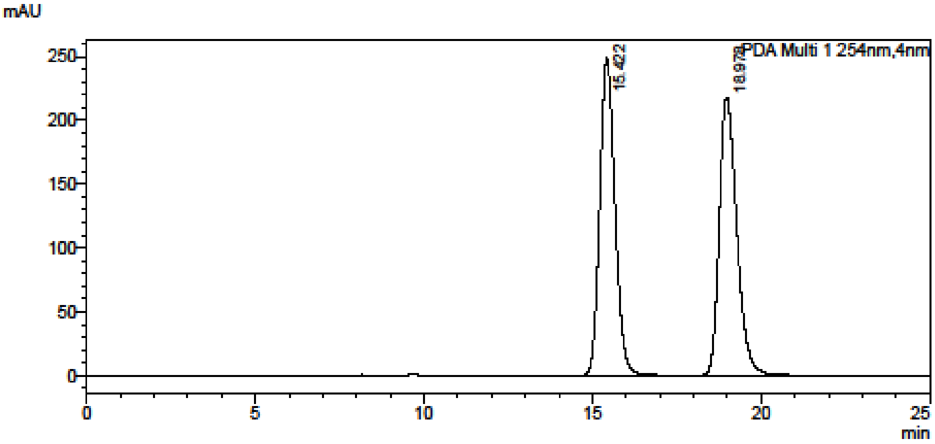

<Peak Table>

PDA Ch1 254nm

Peak#	Ret. Time	Area	Area%	Height
1	15.422	7789208	49.925	248538
2	18.978	7812543	50.075	217751
Total		15601751	100.000	466289

C:\Andrey\CF3(styrenyl)OBnCF3_rac1.lcd

Supplementary figure 299. HPLC chromatogram for compound **8f,** racemic

Analysis Report

<Sample Information>

Sample Name	: CF3(vin)O(2-indanyl)		
Sample ID	: CF3(vin)O(2-indanyl)		
Data Filename	: CF3(vin)O(2-indanyl).lcd		
Method Filename	: andrey.lcm		
Batch Filename	:		
Vial #	: 1-85	Sample Type	: Unknown
Injection Volume	: 1 uL		
Date Acquired	: 08/05/2018 11:16:34	Acquired by	: System Administrator
Date Processed	: 08/05/2018 16:58:41	Processed by	: System Administrator

<Chromatogram>

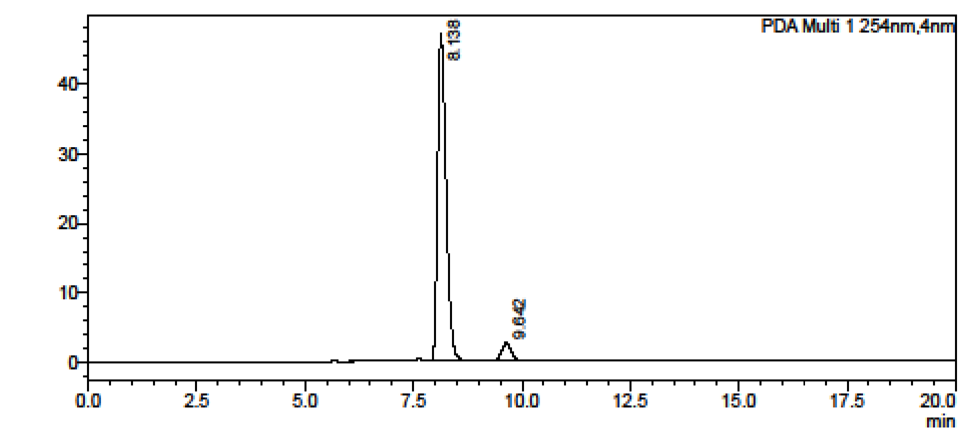

<Peak Table>

PDA Ch1 254nm

Peak#	Ret. Time	Area	Area%	Height
1	8.138	632350	94.165	46951
2	9.642	39184	5.835	2566
Total		671534	100.000	49518

C:\Andrey\CF3(vin)OBnCF4.lcd

Supplementary figure 300. HPLC chromatogram for compound **8g,** Ligand:**(4R)-7g**

LabSolutions

Analysis Report

<Sample Information>

Sample Name	: CF3(vinyll)O(2-indanyl)		
Sample ID	: CF3(vinyll)O(2-indanyl)		
Data Filename	: CF3(vinyll)O(2-indanyl)1.lcd		
Method Filename	: andrey.lcm		
Batch Filename	:		
Vial #	: 1-69	Sample Type	: Unknown
Injection Volume	: 2 uL		
Date Acquired	: 30/04/2018 16:24:02	Acquired by	: System Administrator
Date Processed	: 08/05/2018 17:03:29	Processed by	: System Administrator

<Chromatogram>

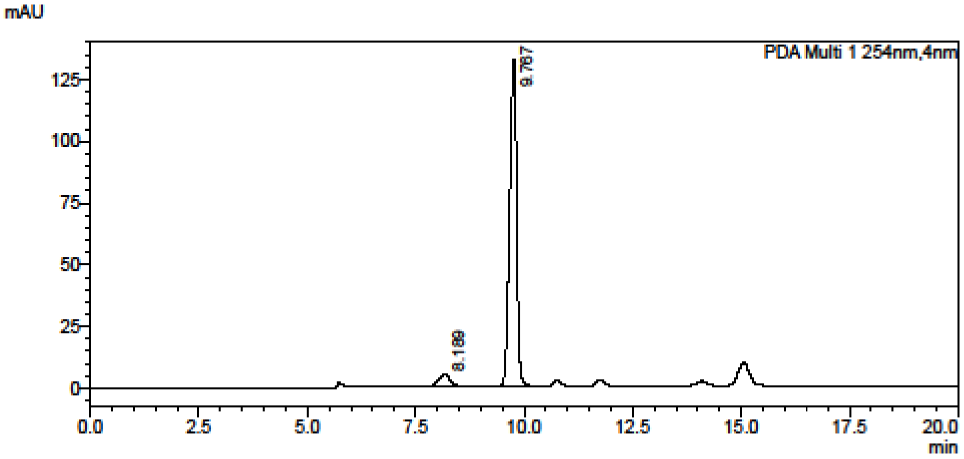

<Peak Table>

PDA Ch1 254nm

Peak#	Ret. Time	Area	Area%	Height
1	8.189	83470	5.308	4459
2	9.767	1488965	94.692	131889
Total		1572435	100.000	136347

C:\Andrey\CF3(vinyll)O(2-indanyl)1.lcd

Supplementary figure 301. HPLC chromatogram for compound **8g,** Ligand:**(4S)-7g**

SHIMADZU LabSolutions Analysis Report

<Sample Information>

Sample Name	: CF3(vinyll)O(2-Indanyl)		
Sample ID	: CF3(vinyll)O(2-Indanyl)		
Data Filename	: CF3(vinyll)O(2-Indanyl)__9.lcd		
Method Filename	: andrey.lcm		
Batch Filename	:		
Vial #	: 1-85	Sample Type	: Unknown
Injection Volume	: 1 uL		
Date Acquired	: 14/05/2018 14:16:48	Acquired by	: System Administrator
Date Processed	: 18/05/2018 16:19:50	Processed by	: System Administrator

<Chromatogram>

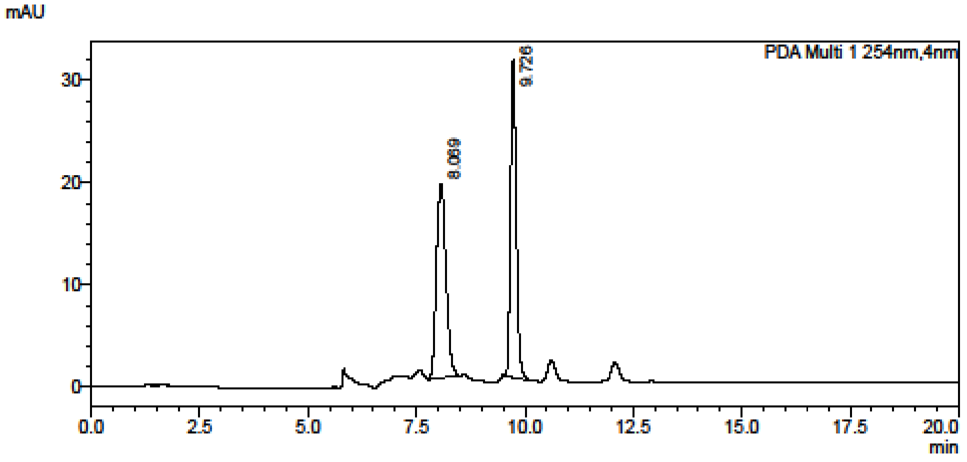

<Peak Table>

PDA Ch1 254nm

Peak#	Ret. Time	Area	Area%	Height
1	8.069	289109	50.381	19048
2	9.726	284741	49.619	30998
Total		573850	100.000	50046

C:\Andrey\CF3(vinyll)O(2-Indanyl)__9.lcd

Supplementary figure 302. HPLC chromatogram for compound **8f,** racemic

Analysis Report

<Sample Information>

Sample Name	: CF3-(Ph)OH, from Bn-CF3		
Sample ID	: CF3-(Ph)OH, from Bn-CF3		
Data Filename	: CF3-(Ph)OH, from Bn-CF3.lcd		
Method Filename	: new.lcm		
Batch Filename	:		
Vial #	: 1-8	Sample Type	: Unknown
Injection Volume	: 10 uL		
Date Acquired	: 28/01/2017 20:11:01	Acquired by	: System Administrator
Date Processed	: 22/08/2017 15:27:37	Processed by	: System Administrator

<Chromatogram>

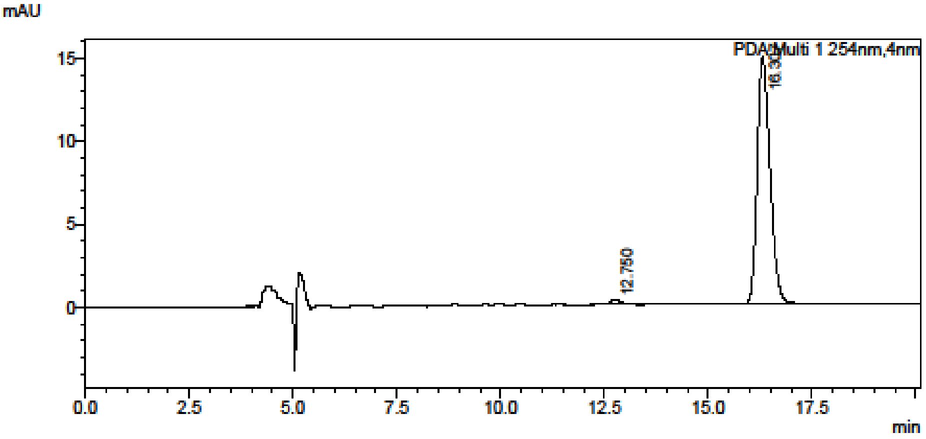

<Peak Table>

PDA Ch1 254nm

Peak#	Ret. Time	Area	Area%	Height
1	12.750	4825	1.510	279
2	16.307	314764	98.490	14822
Total		319589	100.000	15101

C:\Andrey\CF3-(Ph)OH, from Bn-CF3.lcd

Supplementary figure 303. HPLC chromatogram for compound **9a,** from **6a** with ligand **(4S,5R)-7a**

SHIMADZU LabSolutions **Analysis Report**

<Sample Information>

Sample Name	: R-CF3-(Ph)OH, ref
Sample ID	: R-CF3-(Ph)OH, ref
Data Filename	: R-CF3-(Ph)OH, ref2.lcd
Method Filename	: new.lcm
Batch Filename	:
Vial #	: 1-7
Injection Volume	: 10 uL
Date Acquired	: 28/01/2017 19:51:10
Date Processed	: 22/08/2017 15:29:41
Sample Type	: Unknown
Acquired by	: System Administrator
Processed by	: System Administrator

<Chromatogram>

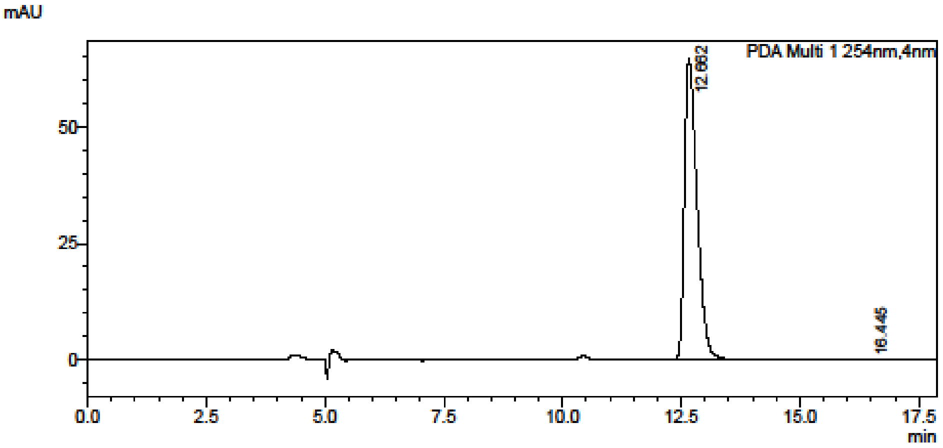

<Peak Table>

PDA Ch1 254nm

Peak#	Ret. Time	Area	Area%	Height
1	12.662	1189678	99.872	64446
2	16.445	1519	0.128	78
Total		1191197	100.000	64524

C:\Andrey\R-CF3-(Ph)OH, ref2.lcd

Supplementary figure 304. HPLC chromatogram for compound**:** commercial (R)-**9a**

SHIMADZU LabSolutions **Analysis Report**

<Sample Information>

Sample Name	: CF3(OH)-styrenyl		
Sample ID	: CF3(OH)-styrenyl		
Data Filename	: CF3(OH)-styrenyl2.lcd		
Method Filename	: shutdown.lcm		
Batch Filename	:		
Vial #	: 1-102	Sample Type	: Unknown
Injection Volume	: 2 uL		
Date Acquired	: 02/07/2017 16:50:55	Acquired by	: System Administrator
Date Processed	: 22/08/2017 15:34:56	Processed by	: System Administrator

<Chromatogram>

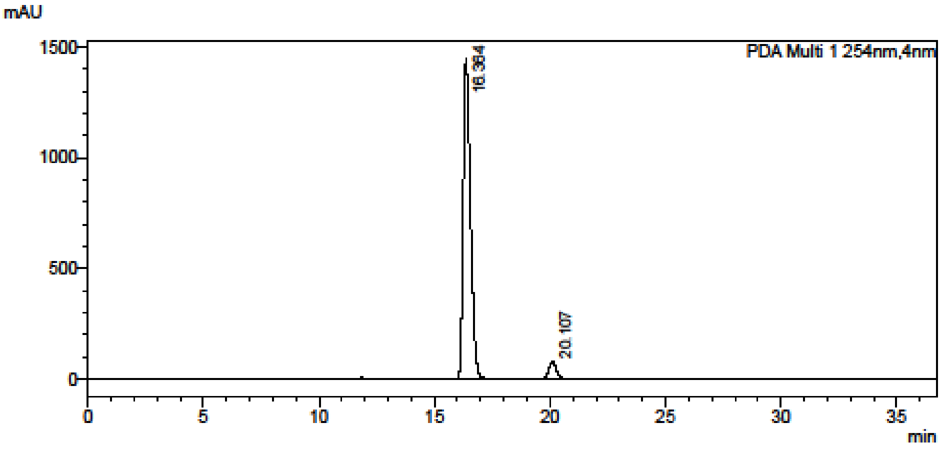

<Peak Table>

PDA Ch1 254nm

Peak#	Ret. Time	Area	Area%	Height
1	16.364	32477253	94.575	1443248
2	20.107	1862981	5.425	76595
Total		34340235	100.000	1519843

C:\Andrey\CF3(OH)-styrenyl2.lcd

Supplementary figure 305. HPLC chromatogram for compound**: 9b,** from **8c** with ligand **(4R)-7g**

Analysis Report

<Sample Information>

Sample Name	: CF3(OH)CH2CH2Ph		
Sample ID	: CF3(OH)CH2CH2Ph		
Data Filename	: CF3(OH)CH2CH2Ph7.lcd		
Method Filename	: andrey.lcm		
Batch Filename	:		
Vial #	: 1-32	Sample Type	: Unknown
Injection Volume	: 3 uL		
Date Acquired	: 20/11/2017 09:44:55	Acquired by	: System Administrator
Date Processed	: 31/12/2017 15:24:30	Processed by	: System Administrator

<Chromatogram>

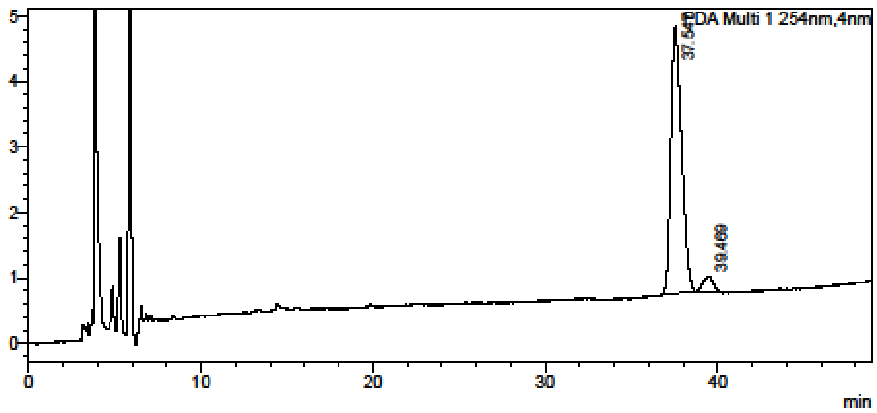

<Peak Table>

PDA Ch1 254nm

Peak#	Ret. Time	Area	Area%	Height
1	37.547	171147	94.762	4082
2	39.469	9460	5.238	248
Total		180606	100.000	4330

C:\Andrey\CF3(OH)CH2CH2Ph7.lcd

Supplementary figure 306. HPLC chromatogram for compound **9c,** from **8f** with ligand **(4R)-7g**

Analysis Report

<Sample Information>

Sample Name	: CF3(p-MeOPh)-OCH2CH2OBu		
Sample ID	: CF3(p-MeOPh)-OCH2CH2OBu		
Data Filename	: CF3(p-MeOPh)-OCH2CH2OBu14.lcd		
Method Filename	: andrey.lcm		
Batch Filename	:		
Vial #	: 1-67	Sample Type	: Unknown
Injection Volume	: 2 uL		
Date Acquired	: 11/07/2017 11:21:20	Acquired by	: System Administrator
Date Processed	: 21/08/2017 16:26:00	Processed by	: System Administrator

<Chromatogram>

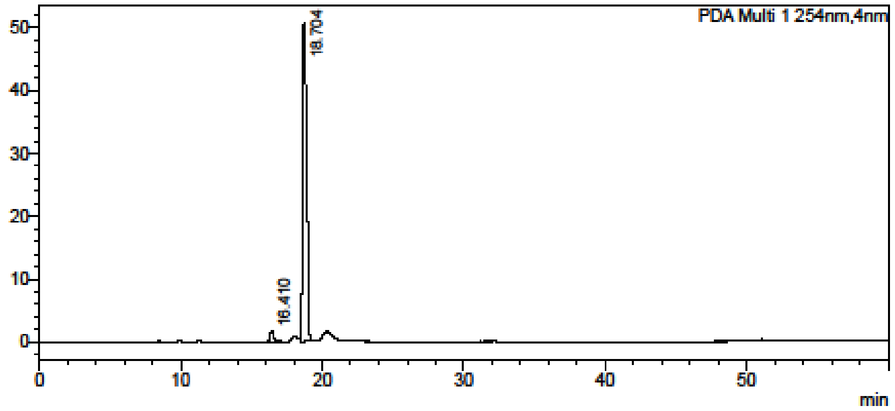

<Peak Table>

PDA Ch1 254nm

Peak#	Ret. Time	Area	Area%	Height
1	16.410	33137	3.314	1847
2	18.704	966714	96.686	50535
Total		999851	100.000	52382

C:\Andrey\CF3(p-MeOPh)-OCH2CH2OBu14.lcd

Supplementary figure 307. HPLC chromatogram for compound **11a,** Ligand:**(4S,5R)-7a**

Analysis Report

<Sample Information>

Sample Name	: CF3(p-MeOPh)-OCH2CH2OBu
Sample ID	: CF3(p-MeOPh)-OCH2CH2OBu
Data Filename	: CF3(p-MeOPh)-OCH2CH2OBu13.lcd
Method Filename	: andrey.lcm
Batch Filename	:
Vial #	: 1-66
Sample Type	: Unknown
Injection Volume	: 2 uL
Date Acquired	: 11/07/2017 10:20:43
Acquired by	: System Administrator
Date Processed	: 21/08/2017 16:29:10
Processed by	: System Administrator

<Chromatogram>

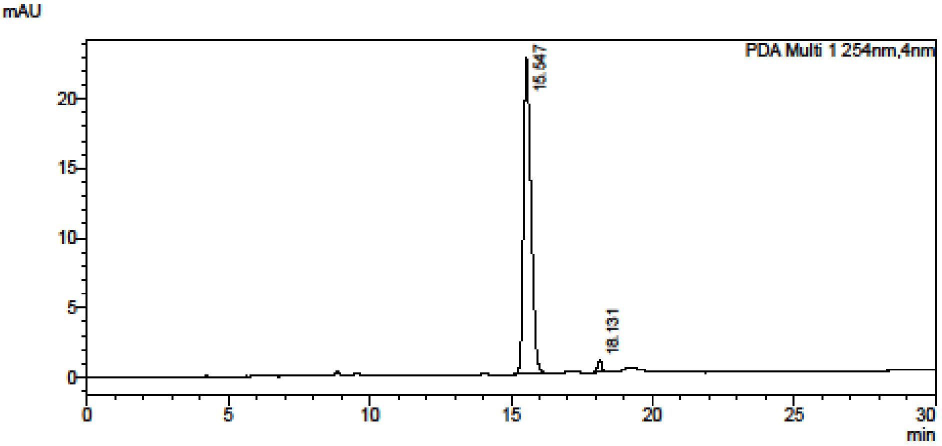

<Peak Table>

PDA Ch1 254nm

Peak#	Ret. Time	Area	Area%	Height
1	15.547	426781	97.935	22662
2	18.131	8998	2.065	856
Total		435780	100.000	23518

C:\Andrey\CF3(p-MeOPh)-OCH2CH2OBu13.lcd

Supplementary figure 308. HPLC chromatogram for compound **11a,** Ligand:**(4R,5S)-7a**

Analysis Report

<Sample Information>

Sample Name	: CF3(p-MeOPh)-OCH2CH2OBu		
Sample ID	: CF3(p-MeOPh)-OCH2CH2OBu		
Data Filename	: CF3(p-MeOPh)-OCH2CH2OBu18.lcd		
Method Filename	: andrey.lcm		
Batch Filename	:		
Vial #	: 1-76	Sample Type	: Unknown
Injection Volume	: 2 uL		
Date Acquired	: 11/07/2017 15:46:49	Acquired by	: System Administrator
Date Processed	: 13/08/2017 16:05:24	Processed by	: System Administrator

<Chromatogram>

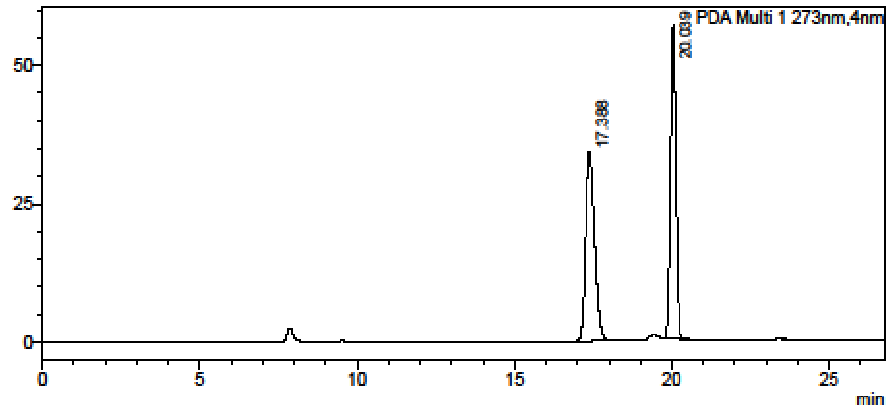

<Peak Table>

PDA Ch1 273nm

Peak#	Ret. Time	Area	Area%	Height
1	17.388	688475	50.455	34190
2	20.039	676060	49.545	56718
Total		1364534	100.000	90908

C:\Andrey\CF3(p-MeOPh)-OCH2CH2OBu18.lcd

Supplementary figure 309. HPLC chromatogram for compound **11a,** racemic

Analysis Report

<Sample Information>

Sample Name : CF3(p-MeOPh)-O(6-EtOhexanoyl)
Sample ID : CF3(p-MeOPh)-O(6-EtOhexanoyl)
Data Filename : CF3(p-MeOPh)-O(6-EtOhexanoyl)2.lcd
Method Filename : andrey.lcm
Batch Filename :
Vial # : 1-65
Sample Type : Unknown
Injection Volume : 2 uL
Date Acquired : 09/07/2017 20:42:28
Acquired by : System Administrator
Date Processed : 10/07/2017 17:48:33
Processed by : System Administrator

<Chromatogram>

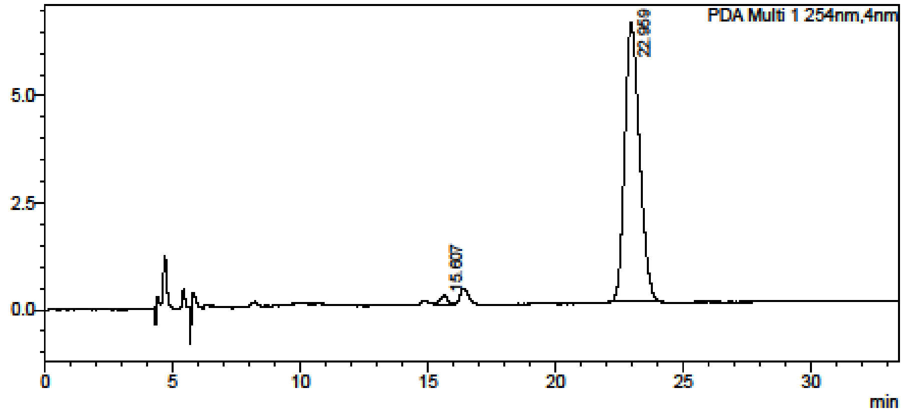

<Peak Table>

PDA Ch1 254nm

Peak#	Ret. Time	Area	Area%	Height
1	15.607	4300	1.597	203
2	22.959	264907	98.403	6491
Total		269207	100.000	6694

Supplementary figure 310. HPLC chromatogram for compound **11b,** Ligand:**(4S,5R)-7a**

Analysis Report

<Sample Information>

Sample Name : CF3(p-MeO)-O(6-EtOhexanoyl),Me(4,5diPh)_BOX
Sample ID : CF3(p-MeO)-O(6-EtOhexanoyl),Me(
Data Filename : CF3(p-MeO)-O(6-EtOhexanoyl),Me(4,5diPh)_BOX2.lcd
Method Filename : andrey.lcm
Batch Filename :
Vial # : 1-49　　Sample Type : Unknown
Injection Volume : 5 uL
Date Acquired : 21/05/2017 15:35:33　　Acquired by : System Administrator
Date Processed : 10/07/2017 17:49:33　　Processed by : System Administrator

<Chromatogram>

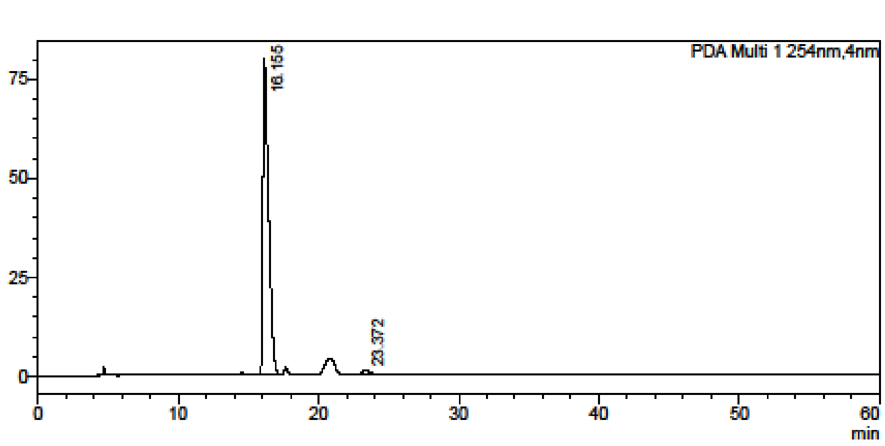

<Peak Table>

PDA Ch1 254nm

Peak#	Ret. Time	Area	Area%	Height
1	16.155	2201271	98.053	79787
2	23.372	43720	1.947	1080
Total		2244992	100.000	80867

C:\Andrey\CF3(p-MeO)-O(6-EtOhexanoyl),Me(4,5diPh)_BOX2.lcd

Supplementary figure 311. HPLC chromatogram for compound **11b,** Ligand:**(4R,5S)-7a**

SHIMADZU LabSolutions

Analysis Report

<Sample Information>

Sample Name	: CF3(p-MeOPh)-O(CH2)5COOEt_rac
Sample ID	: CF3(p-MeOPh)-O(CH2)5COOEt_rac
Data Filename	: CF3(p-MeOPh)-O(CH2)5COOEt_rac2.lcd
Method Filename	: andrey.lcm
Batch Filename	:
Vial #	: 1-78
Sample Type	: Unknown
Injection Volume	: 2 uL
Date Acquired	: 12/07/2017 14:48:52
Acquired by	: System Administrator
Date Processed	: 13/08/2017 16:02:46
Processed by	: System Administrator

<Chromatogram>

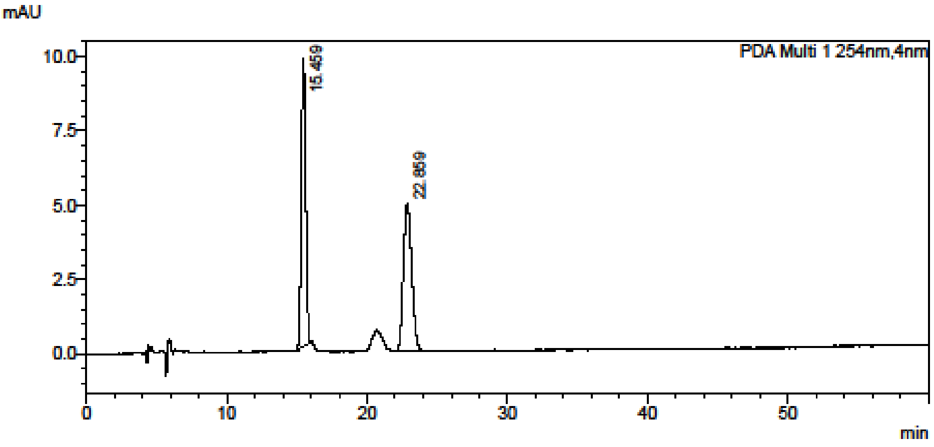

<Peak Table>

PDA Ch1 254nm

Peak#	Ret. Time	Area	Area%	Height
1	15.459	205310	50.589	9664
2	22.859	200527	49.411	4932
Total		405837	100.000	14595

C:\Andrey\CF3(p-MeOPh)-O(CH2)5COOEt_rac2.lcd

Supplementary figure 312. HPLC chromatogram for compound **11b,** racemic

Analysis Report

<Sample Information>

Sample Name	: CF3(p-MeOPh)-(CH2)3NHBoc
Sample ID	: CF3(p-MeOPh)-(CH2)3NHBoc
Data Filename	: CF3(p-MeOPh)-(CH2)3NHBoc3.lcd
Method Filename	: andrey.lcm
Batch Filename	:
Vial #	: 1-81
Sample Type	: Unknown
Injection Volume	: 2 uL
Date Acquired	: 13/07/2017 17:31:27
Acquired by	: System Administrator
Date Processed	: 13/07/2017 21:49:05
Processed by	: System Administrator

<Chromatogram>

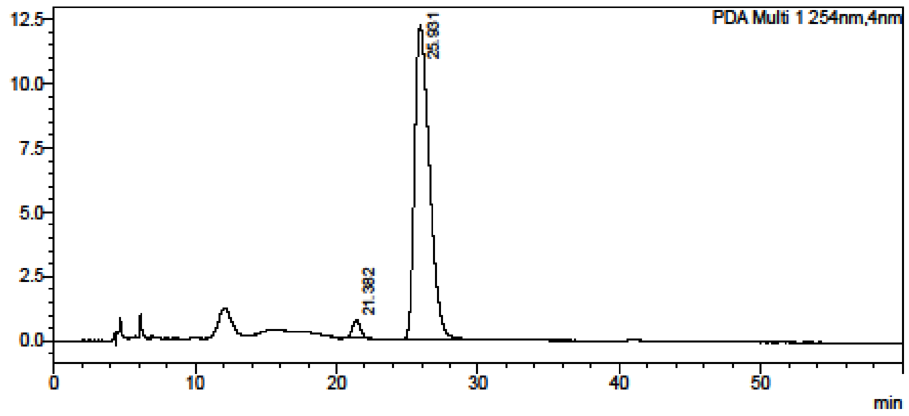

<Peak Table>

PDA Ch1 254nm

Peak#	Ret. Time	Area	Area%	Height
1	21.382	28674	3.123	702
2	25.931	889632	96.877	12219
Total		918306	100.000	12921

Supplementary figure 313. HPLC chromatogram for compound **11c,** Ligand:**(4S,5R)-7a**

SHIMADZU LabSolutions

Analysis Report

<Sample Information>

Sample Name	: CF3(p-MeOPh)-(CH2)3NHBoc
Sample ID	: CF3(p-MeOPh)-(CH2)3NHBoc
Data Filename	: CF3(p-MeOPh)-(CH2)3NHBoc2.lcd
Method Filename	: andrey.lcm
Batch Filename	:
Vial #	: 1-80
Sample Type	: Unknown
Injection Volume	: 2 uL
Date Acquired	: 13/07/2017 16:32:47
Acquired by	: System Administrator
Date Processed	: 21/08/2017 16:54:44
Processed by	: System Administrator

<Chromatogram>

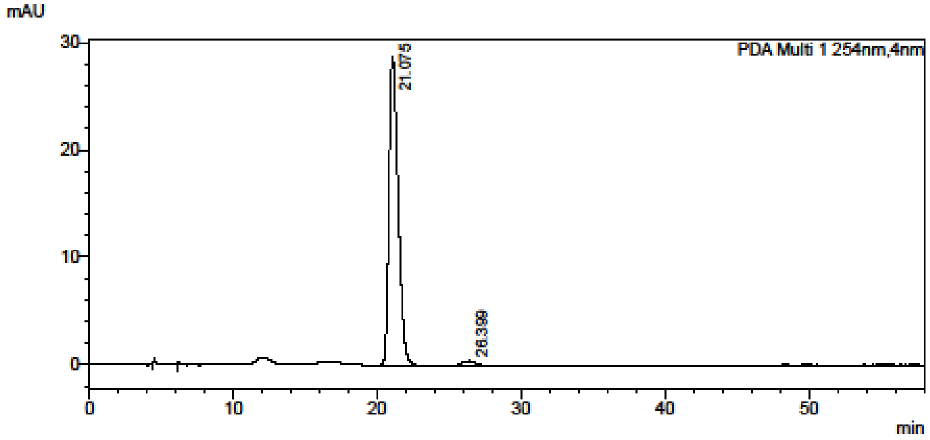

<Peak Table>

PDA Ch1 254nm

Peak#	Ret. Time	Area	Area%	Height
1	21.075	1267304	97.507	28763
2	26.399	32402	2.493	459
Total		1299706	100.000	29222

C:\Andrey\CF3(p-MeOPh)-(CH2)3NHBoc2.lcd

Supplementary figure 314. HPLC chromatogram for compound **11c,** Ligand:**(4R,5S)-7a**

SHIMADZU LabSolutions

Analysis Report

<Sample Information>

Sample Name	: CF3(pMeOPh)O(CH2)3NHBoc_rac		
Sample ID	: CF3(pMeOPh)O(CH2)3NHBoc_rac		
Data Filename	: CF3(pMeOPh)O(CH2)3NHBoc_rac1.lcd		
Method Filename	: andrey.lcm		
Batch Filename	:		
Vial #	: 1-96	Sample Type	: Unknown
Injection Volume	: 3 uL		
Date Acquired	: 10/08/2017 17:32:55	Acquired by	: System Administrator
Date Processed	: 10/08/2017 18:11:29	Processed by	: System Administrator

<Chromatogram>

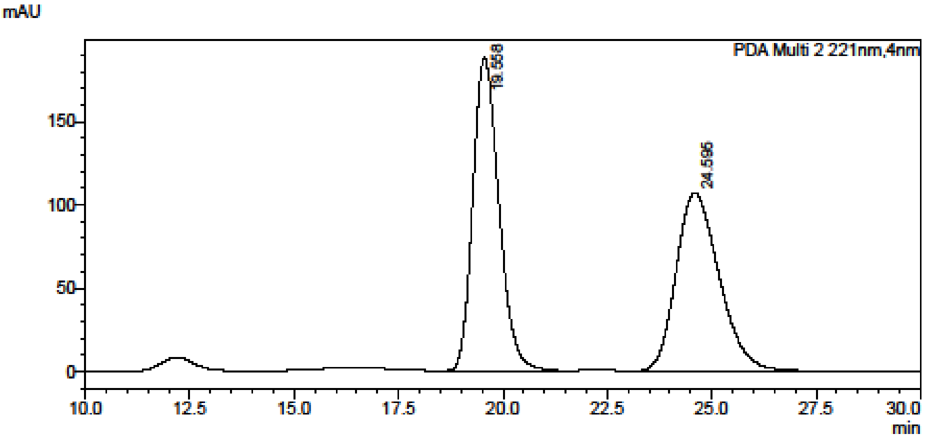

<Peak Table>

PDA Ch2 221nm

Peak#	Ret. Time	Area	Area%	Height
1	19.558	8195126	51.424	188162
2	24.595	7741165	48.576	107180
Total		15936291	100.000	295342

C:\Andrey\CF3(pMeOPh)O(CH2)3NHBoc_rac1.lcd

Supplementary figure 315. HPLC chromatogram for compound: **11c,** racemic

SHIMADZU LabSolutions **Analysis Report**

<Sample Information>

Sample Name	: CF3(p-MeOPh)-dec_old		
Sample ID	: CF3(p-MeOPh)-dec_old		
Data Filename	: CF3(p-MeOPh)-dec_old3.lcd		
Method Filename	: andrey.lcm		
Batch Filename	:		
Vial #	: 1-79	Sample Type	: Unknown
Injection Volume	: 2 uL		
Date Acquired	: 12/07/2017 19:14:39	Acquired by	: System Administrator
Date Processed	: 21/08/2017 16:58:12	Processed by	: System Administrator

<Chromatogram>

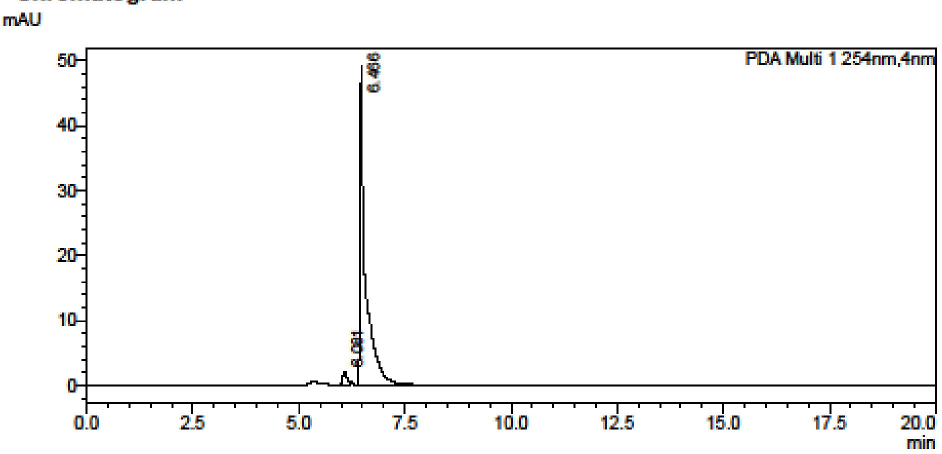

<Peak Table>

PDA Ch1 254nm

Peak#	Ret. Time	Area	Area%	Height
1	6.081	13670	2.988	2042
2	6.466	443841	97.012	49039
Total		457510	100.000	51081

C:\Andrey\CF3(p-MeOPh)-dec_old3.lcd

Supplementary figure 316. HPLC chromatogram for compound **11d,** Ligand:**(4S,5R)-7a**

Analysis Report

<Sample Information>

Sample Name	: CF3(p-MeOPh)-ODec Me(4.5DiPh)BOX		
Sample ID	: CF3(p-MeOPh)-ODec Me(4.5DiPh)BO		
Data Filename	: CF3(p-MeOPh)-ODec Me(4.5DiPh)BOX13.lcd		
Method Filename	: andrey.lcm		
Batch Filename	:		
Vial #	: 1-58	Sample Type	: Unknown
Injection Volume	: 2 uL		
Date Acquired	: 12/06/2017 19:02:08	Acquired by	: System Administrator
Date Processed	: 21/08/2017 16:59:43	Processed by	: System Administrator

<Chromatogram>

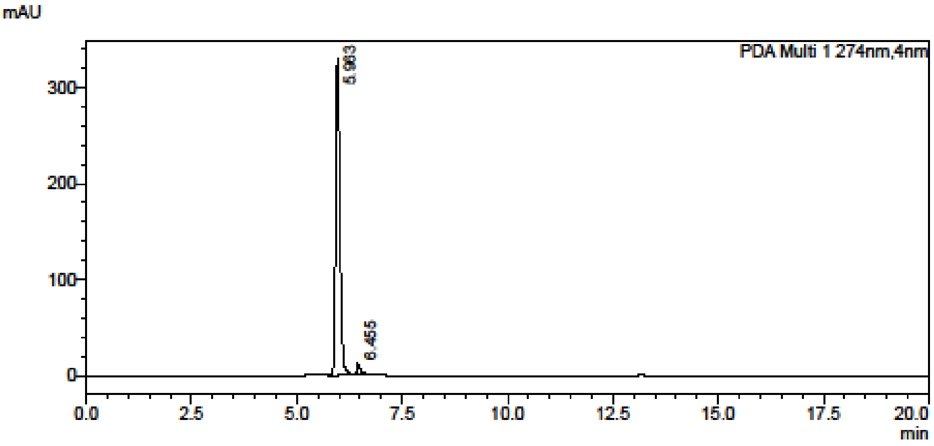

<Peak Table>

PDA Ch1 274nm

Peak#	Ret. Time	Area	Area%	Height
1	5.963	2343179	97.032	329134
2	6.455	71684	2.968	11700
Total		2414863	100.000	340834

C:\Andrey\CF3(p-MeOPh)-ODec Me(4.5DiPh)BOX13.lcd

Supplementary figure 317. HPLC chromatogram for compound **11d,** Ligand:**(4R,5S)-7a**

SHIMADZU LabSolutions **Analysis Report**

<Sample Information>

Sample Name	: CF3(pMeOPh)ODec		
Sample ID	: CF3(pMeOPh)ODec		
Data Filename	: CF3(pMeOPh)ODec_rac1.lcd		
Method Filename	: andrey.lcm		
Batch Filename	:		
Vial #	: 1-97	Sample Type	: Unknown
Injection Volume	: 2 uL		
Date Acquired	: 10/08/2017 15:45:53	Acquired by	: System Administrator
Date Processed	: 10/08/2017 18:06:03	Processed by	: System Administrator

<Chromatogram>

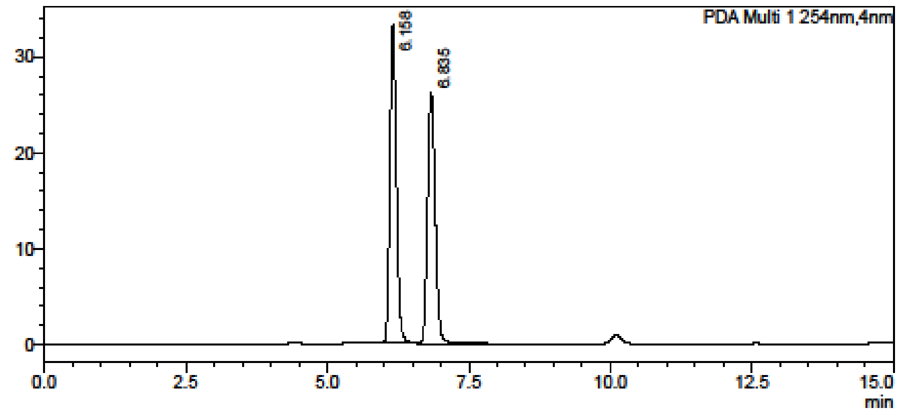

<Peak Table>

PDA Ch1 254nm

Peak#	Ret. Time	Area	Area%	Height
1	6.158	261092	51.904	33242
2	6.835	241935	48.096	26225
Total		503027	100.000	59467

C:\Andrey\CF3(pMeOPh)ODec_rac1.lcd

Supplementary figure 318. HPLC chromatogram for compound **11d,** racemic

Analysis Report

<Sample Information>

Sample Name	: CF3(pMeOPh)O(2-indanlyl)		
Sample ID	: CF3(pMeOPh)O(2-indanlyl)		
Data Filename	: CF3(pMeOPh)O(2-indanlyl)_r1.lcd		
Method Filename	: Cholest.lcm		
Batch Filename	:		
Vial #	: 1-97	Sample Type	: Unknown
Injection Volume	: 1 uL		
Date Acquired	: 19/05/2018 19:15:15	Acquired by	: System Administrator
Date Processed	: 19/05/2018 19:43:57	Processed by	: System Administrator

<Chromatogram>

mAU

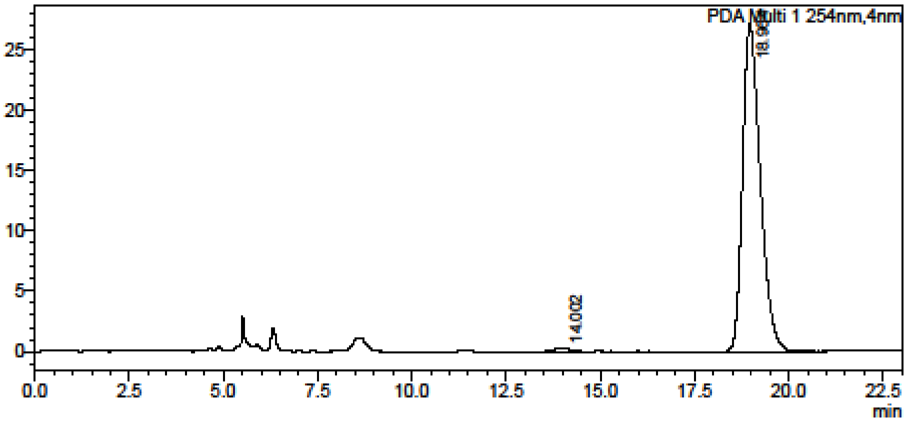

<Peak Table>

PDA Ch1 254nm

Peak#	Ret. Time	Area	Area%	Height
1	14.002	8237	0.903	314
2	18.964	904264	99.097	27093
Total		912501	100.000	27406

C:\Andrey\CF3(pMeOPh)O(2-indanlyl)_r1.lcd

Supplementary figure 319. HPLC chromatogram for compound **11e,** Ligand:**(4R,5S)-7a**

LabSolutions **Analysis Report**

<Sample Information>

Sample Name	: CF3(pMeOPh)O(2-indanlyl)		
Sample ID	: CF3(pMeOPh)O(2-indanlyl)		
Data Filename	: CF3(pMeOPh)O(2-indanlyl)_s1.lcd		
Method Filename	: Cholest.lcm		
Batch Filename	:		
Vial #	: 1-95	Sample Type	: Unknown
Injection Volume	: 1 uL		
Date Acquired	: 19/05/2018 18:21:04	Acquired by	: System Administrator
Date Processed	: 19/05/2018 19:12:12	Processed by	: System Administrator

<Chromatogram>

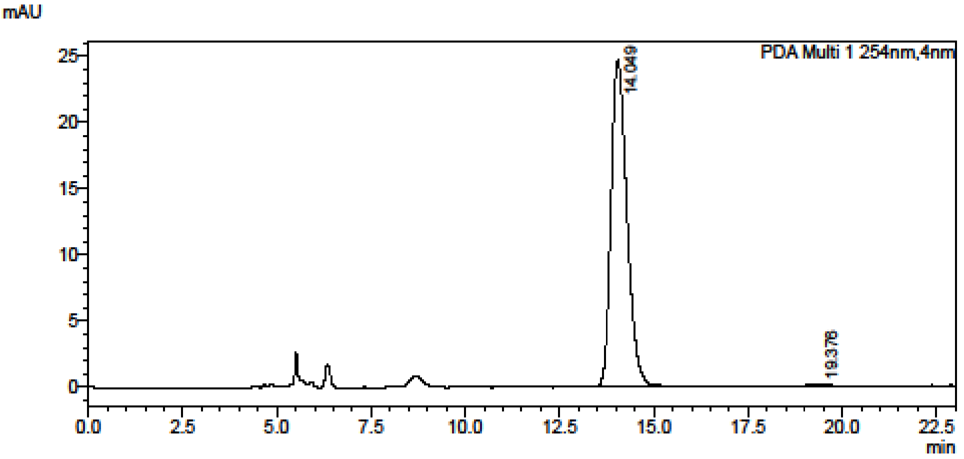

<Peak Table>

PDA Ch1 254nm

Peak#	Ret. Time	Area	Area%	Height
1	14.049	726111	99.351	24651
2	19.376	4741	0.649	162
Total		730851	100.000	24813

C:\Andrey\CF3(pMeOPh)O(2-indanlyl)_s1.lcd

Supplementary figure 320. HPLC chromatogram for compound **11e,** Ligand:**(4S,5R)-7a**

Analysis Report

<Sample Information>

Sample Name	: CF3(pMeOPh)O(2-indanlyl)		
Sample ID	: CF3(pMeOPh)O(2-indanlyl)		
Data Filename	: CF3(pMeOPh)O(2-indanlyl)_rac1.lcd		
Method Filename	: Cholest.lcm		
Batch Filename	:		
Vial #	: 1-96	Sample Type	: Unknown
Injection Volume	: 1 uL		
Date Acquired	: 19/05/2018 18:48:04	Acquired by	: System Administrator
Date Processed	: 19/05/2018 19:38:34	Processed by	: System Administrator

<Chromatogram>

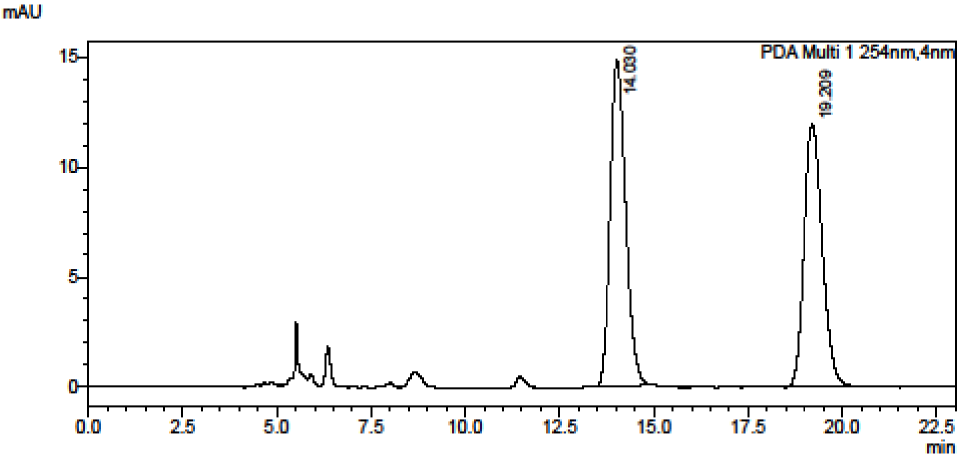

<Peak Table>

PDA Ch1 254nm

Peak#	Ret. Time	Area	Area%	Height
1	14.030	427500	51.495	14839
2	19.209	402672	48.505	11953
Total		830172	100.000	26792

C:\Andrey\CF3(pMeOPh)O(2-indanlyl)_rac1.lcd

Supplementary figure 321. HPLC chromatogram for compound **11e,** racemic

SHIMADZU LabSolutions

Analysis Report

<Sample Information>

Sample Name : CF3(pMeOPh)O(Cholesterol)
Sample ID : CF3(pMeOPh)O(Cholesterol)
Data Filename : CF3(pMeOPh)O(Cholesterol)_r7.lcd
Method Filename : andrey.lcm
Batch Filename :
Vial # : 1-104
Sample Type : Unknown
Injection Volume : 1 uL
Date Acquired : 16/05/2018 18:39:11
Acquired by : System Administrator
Date Processed : 18/05/2018 16:27:53
Processed by : System Administrator

<Chromatogram>

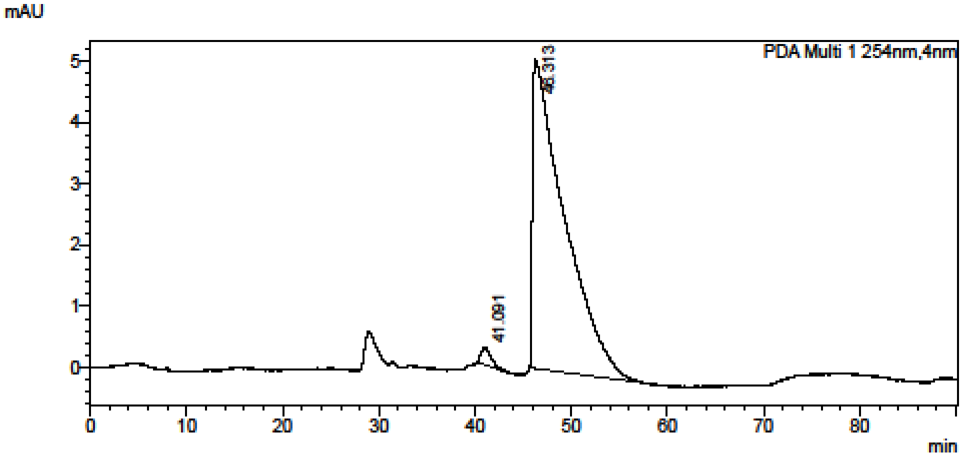

<Peak Table>

PDA Ch1 254nm

Peak#	Ret. Time	Area	Area%	Height
1	41.091	20443	1.733	281
2	46.313	1159302	98.267	5044
Total		1179745	100.000	5326

Supplementary figure 322. HPLC chromatogram for compound **11f,** Ligand:**(4R,5S)-7a**

Analysis Report

<Sample Information>

Sample Name	: CF3(p-MeOPh)O(Cholesterol)_(S)		
Sample ID	: CF3(p-MeOPh)O(Cholesterol)_(S)		
Data Filename	: CF3(p-MeOPh)O(Cholesterol)_(S)_1.lcd		
Method Filename	: andrey.lcm		
Batch Filename	: 1.lcb		
Vial #	: 1-103	Sample Type	: Unknown
Injection Volume	: 1 uL		
Date Acquired	: 16/05/2018 20:14:20	Acquired by	: System Administrator
Date Processed	: 18/05/2018 16:24:18	Processed by	: System Administrator

<Chromatogram>

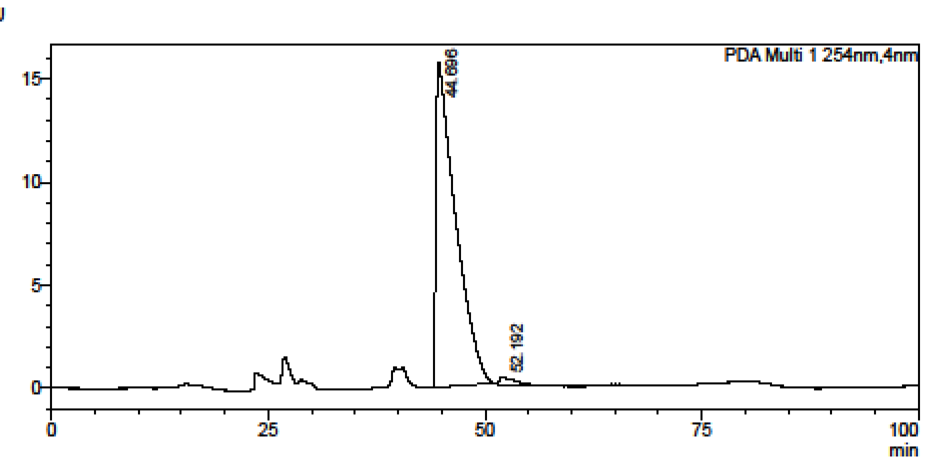

<Peak Table>

PDA Ch1 254nm

Peak#	Ret. Time	Area	Area%	Height
1	44.696	2470279	98.129	15704
2	52.192	47091	1.871	335
Total		2517369	100.000	16038

C:\Andrey\CF3(p-MeOPh)O(Cholesterol)_(S)_1.lcd

Supplementary figure 323. HPLC chromatogram for compound **11f,** Ligand:**(4S,5R)-7a**

Analysis Report

<Sample Information>

Sample Name	: CF3(p-MeOPh)O(Cholesterol)_(rac)		
Sample ID	: CF3(p-MeOPh)O(Cholesterol)_(rac		
Data Filename	: CF3(p-MeOPh)O(Cholesterol)_(rac)_1.lcd		
Method Filename	: andrey.lcm		
Batch Filename	: 1.lcb		
Vial #	: 1-102	Sample Type	: Unknown
Injection Volume	: 1 uL		
Date Acquired	: 16/05/2018 21:54:46	Acquired by	: System Administrator
Date Processed	: 18/05/2018 16:25:55	Processed by	: System Administrator

<Chromatogram>

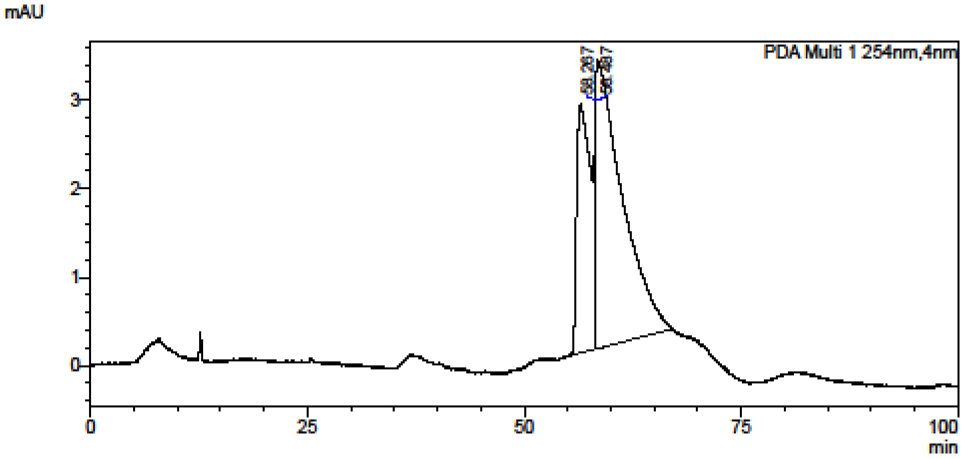

<Peak Table>

PDA Ch1 254nm

Peak#	Ret. Time	Area	Area%	Height
1	58.267	343325	33.520	3081
2	58.487	680929	66.480	3257
Total		1024254	100.000	6339

C:\Andrey\CF3(p-MeOPh)O(Cholesterol)_(rac)_1.lcd

Supplementary figure 324. HPLC chromatogram for compound **11f,**: **racemic**

Supplementary references

1. Handy C. J., Lam Y.-F., DeShong P. On the Synthesis and NMR Analysis of Tetrabutylammonium Triphenyldifluorosilicate. *The Journal of Organic Chemistry* 2000, **65**(11)**:** 3542-3543.

2. Wettergren J., Ankner T., Hilmersson G. Selective [small alpha]-defluorination of polyfluorinated esters and amides using SmI2/Et3N/H2O. *Chemical Communications* 2010, **46**(40)**:** 7596-7598.

3. Montiel-Smith S., Meza-Reyes S., Viñas-Bravo O., Fernández-Herrera M. A., Martínez-Pascual R., Sandoval-Ramírez J.*, et al.* In situ preparation of mixed anhydrides containing the trifluoroacetyl moiety. Application to the esterification of cholesterol and phenol. *Arkivoc* 2005, **2005**(6).

4. Lin J.-H., Xiao J.-C. The Asymmetric Friedel–Crafts Reaction of Indoles with Fluoroalkylated Nitroalkenes Catalyzed by Chiral Phosphoric Acid. *European Journal of Organic Chemistry* 2011, **2011**(24)**:** 4536-4539.

5. Bose A. K., Lal B. A facile replacement of hydroxyl by halogen with inversion. *Tetrahedron Letters* 1973, **14**(40)**:** 3937-3940.

6. Murata M., Ishikura M., Nagata M., Watanabe S., Masuda Y. Rhodium(I)-Catalyzed Silylation of Aryl Halides with Triethoxysilane: Practical Synthetic Route to Aryltriethoxysilanes. *Org. Lett.* 2002, **4**(11)**:** 1843-1845.

7. Tacke R., Mahner K., Strohmann C., Forth B., Mutschler E., Friebe T.*, et al.* Cyclohexy (4-fluorophenyl)(3-piperidinopropyl)silanol ((itp)-fluoro-hexahydro-sila-difenidol, p-F-HSSiD) and derivatives: synthesis and antimuscarinic properties. *Journal of Organometallic Chemistry* 1991, **417**(3)**:** 339-353.

8. Manoso A. S., Ahn C., Soheili A., Handy C. J., Correia R., Seganish W. M.*, et al.* Improved Synthesis of Aryltrialkoxysilanes via Treatment of Aryl Grignard or Lithium Reagents with Tetraalkyl Orthosilicates. *The Journal of Organic Chemistry* 2004, **69**(24)**:** 8305-8314.

9. Krasovskiy A., Knochel P. A LiCl-mediated Br/Mg exchange reaction for the preparation of functionalized aryl- and heteroarylmagnesium compounds from organic bromides. *Angew. Chem. Int. Ed. Engl.* 2004, **43**(25)**:** 3333-3336.

10. Chauhan M., Hauck B. J., Keller L. P., Boudjouk P. Hydrosilylation of alkynes catalyzed by platinum on carbon. *Journal of Organometallic Chemistry* 2002, **645**(1)**:** 1-13.

11. Corriu R. J. P., Lancelle-Beltran E., Mehdi A., Reye C., Brandes S., Guilard R. Ordered mesoporous hybrid materials containing cobalt(ii) Schiff base complex. *Journal of Materials Chemistry* 2002, **12**(5)**:** 1355-1362.

12. Xu Q., Zhou H., Geng X., Chen P. Nonenzymatic kinetic resolution of racemic 2,2,2-trifluoro-1-aryl ethanol via enantioselective acylation. *Tetrahedron* 2009, **65**(11)**:** 2232-2238.

13. Fuji K., Kawabata T., Fujita E. Hard acid and soft nucleophile system. IV. Removal of benzyl protecting group with boron trifluoride etherate and dimethyl sulflide. *Chemical & Pharmaceutical Bulletin* 1980, **28**(12)**:** 3662-3664.

14. Song J. J., Tan Z., Reeves J. T., Gallou F., Yee N. K., Senanayake C. H. N-Heterocyclic Carbene Catalyzed Trifluoromethylation of Carbonyl Compounds. *Organic Letters* 2005, **7**(11)**:** 2193-2196.
